# Genome-wide identification, evolution and expression analysis of RING finger protein genes in *Brassica rapa*

**DOI:** 10.1038/srep40690

**Published:** 2017-01-17

**Authors:** Intikhab Alam, Yan-Qing Yang, Yong Wang, Mei-Lan Zhu, Heng-Bo Wang, Boulos Chalhoub, Yun-Hai Lu

**Affiliations:** 1 Key Laboratory of Genetics, Breeding and Comprehensive Utilization of Crops, Ministry of Education, College of Crop Science, Fujian Agriculture and Forestry University, Fuzhou, 350002, China; 2 Unité de Recherche en Génomique Végétale (Institut National de la Recherche Agronomique, Centre National de la Recherche Scientifique, Université d’Evry Val d’Essonnes), Organization and Evolution of Plant Genomes, 91057 Evry cedex, France

## Abstract

More and more RING finger genes were found to be implicated in various important biological processes. In the present study, a total of 731 RING domains in 715 predicted proteins were identified in *Brassica rapa* genome (AA, 2n = 20), which were further divided into eight types: RING-H2 (371), RING-HCa (215), RING-HCb (47), RING-v (44), RING-C2 (38), RING-D (10), RING-S/T (5) and RING-G (1). The 715 RING finger proteins were further classified into 51 groups according to the presence of additional domains. 700 RING finger protein genes were mapped to the 10 chromosomes of *B. rapa* with a range of 47 to 111 genes for each chromosome. 667 RING finger protein genes were expressed in at least one of the six tissues examined, indicating their involvement in various physiological and developmental processes in *B. rapa*. Hierarchical clustering analysis of RNA-seq data divided them into seven major groups, one of which includes 231 members preferentially expressed in leaf, and constitutes then a panel of gene candidates for studying the genetic and molecular mechanisms of leafy head traits in *Brassica* crops. Our results lay the foundation for further studies on the classification, evolution and putative functions of RING finger protein genes in *Brassica* species.

The process of protein degradation is an important post-translational regulatory mechanism that mediates cell responses to intracellular signals and variation of environmental conditions in all eukaryotes^1^. A central major player in this process is the ubiquitin-26S proteasome system (UPS)^1^,^2^. In this system, the proteins destined for degradation are first modified by covalent attachment of multiple ubiquitin (Ub) molecules. Ub is a 76-amino acid protein that is ubiquitously expressed and highly conserved in all eukaryotes. The ubiquitination includes three steps: (1) Ub is activated in an ATP-depend interaction by the Ub-activating enzyme E1 to form an E1-Ub thioester linked intermediate; (2) the activated Ub is transferred to the Ub-conjugating enzyme E2 by interaction of E1-Ub with E2 to form a thioester linked E2-Ub intermediate; (3) Ub is attached, by covalence, to the substrate protein by the Ub-ligase enzyme E3, which interacts with both the E2-Ub and the target protein. The repetition of these three steps attaches then multiple Ub molecules to the substrate, which are thereafter recognized and degraded by the 26S proteasome, while the Ub molecules are recycled by the same pathway. The specificity of ubiquitination is mainly determined by a large number of E3 that recruits specific target protein(s). The level of specificity is further increased by the different possible E2-E3 combinations, thus allowing not only for the attachment of different types of Ub-conjugates but also for the specific regulation of a large number of target proteins. In *Arabidopsis thaliana*, about 6% of proteome are predicted to be UPS-associated^3^. There are two members of E1, 37 predicted members of E2, and more than 1400 predicted E3 Ub-ligases which constitute a large and diverse group and are mainly responsible for the substrate specificity^2^,^3^.

According to domain organization and the ability to carry thioester-linked Ub, E3 ligases can be divided into 4 types: HECT, RING, U-box, and RBR^4^. RING-type E3 ligases are characterized by the presence of a RING domain, which was first identified in a protein encoded by the *Really Interesting New Gene*^5^,^6^ and subsequently found in some key regulatory proteins^7^,^8^. RING domains were defined as a Cys-rich domain of 40–60 amino acids with spatially conserved 8 metal ligands containing Cys or His residues in 4 pairs which can chelate two Zn^2+^ atoms and form a cross-brace structure as a platform for binding of E2s^8^,^9^,^10^. In contrast with the known DNA-binding zinc finger domains, the RING domains function as a protein-protein interaction domain^9^,^10^, and were found to be essential for catalyzing E3 ligase activity of RING-containing proteins^11^.

According to the amino acid residue (Cys or His) at metal ligand position 5, RING domains were basically divided into the two canonical types RING-H2 and RING-HC^12^,^13^. In *A. thaliana*, genome-wide analysis of proteomic sequences had allowed for the identification of 477 RING domains in 469 predicted proteins^10^,^14^, and were further divided into two canonical types including RING-H2 (241) and RING-HC (186), and 5 modified types including RING-v (25), RING-C2 (10), RING-D (10), RING-S/T (4) and RING-G (1). The majority of these RING-containing proteins were shown to be active in *in vitro* ubiquitination essays^14^. In rice, a total of 488 RING domains in 488 predicted proteins were identified from the whole proteome, and divided into 5 types: RING-H2 (281), RING-HC (119), RING-V (23), RING-C2 (2) and non-classified (63)^15^. Eight of ten tested rice RING-containing proteins showed E3 ligase activity^16^. In apple, 688 RING domains in 663 predicted proteins were identified and further divided into 9 RING types: RING-H2 (367), RING-HC (208), RING-v (35), RING-C2 (10), RING-D (1), RING-S/T (11), RING-G (2), RING-mH2 (10) and RING-mHC (44)^17^. In *Ostreococus tauri*, the smallest free-living photosynthetic eukaryote, 65 RING domains in 65 predicted proteins were identified and further divided into 8 RING types: RING-H2 (25), RING-HC (28), RING-v (7), RING-C2 (1), C3HCHC2 (1), C2HC5 (1), C3GC3S (1) and C2SHC4 (1)^18^. Twenty-nine of the 65 RING-containing proteins in *O. tauri* share different degree of homology with those in *A. thaliana*, indicating they might play a conserved function and be necessary for the basic surviving of free-living eukaryotes^18^.

Recently, more and more RING-containing protein genes were found to be implicated in various biological processes, including growth and development, hormone signaling, environmental perception, and stress response, etc. For example, In *A. thaliana, BRH1* is involved in brassinosteroid-responsive^19^, *RIE1* in seed development^20^, *ATL2/ATL9* in defense response^21^,^22^, *XERICO* in drought tolerance^23^, *KEG* in growth and development^24^, *PEX2/PEX10/PEX12* in peroxisome formation^25^,^26^, *NLA* in adaptability to nitrogen limitation^27^, *SHA1* in shoot apical meristem maintenance^28^, *SDIR1* in stress-responsive abscisic acid signaling^29^, *DRIP1*/*DRIP2* is in drought response^30^*, RHA2a/RHA2b* in ABA-mediated stress signaling^31^,^32^, *CNI1* in the carbon/nitrogen response for growth phase transition^33^, *SIS3* in sugar response^34^, *XBAT32* in lateral root production by regulating the ethylene biosynthesis^35^, *AtAIRP1* in ABA-dependent response to drought stress^36^, *DAL1/DAL2* in regulation of programmed cell death^37^, *DAF* in anther dehiscence^38^, *AtAIRP3* in high-salt and drought stress responses^39^, *RSL1* in seed longevity^40^, *AtRING1A* in flowering^41^, *COP1* in photomorphogenesis^42^, *NERF* in drought resistance^43^, *STRF1* in salt-stress response^44^; in *B. rapa, BrRZFP1* is involved in cold, salt and dehydration stress^45^; in *B. napus, Bnclg1A-1D* is involved in cleistogamy trait formation^46^.

The genus *Brassica* includes a number of important crops, providing human nutrition in the form of oil, vegetables, condiments, dietary fiber, and vitamin C 47. Among the *Brassica* species, *B. rapa* (n = 10, AA) is one of the diploid progenitor species which contributes the ‘A’ genome to the allopolyploid oilseed crops, *B. napus* (n = 19, AACC) and *B. juncea* (n = 18, AABB)^48^. Comparing to other *Brassica* species, *B. rapa* has a relatively small genome (about 529 Mbp) 49 and then can be used as a model plant for genomic and evolutionary study in *Brassica* species. The recent sequencing of *B. rapa* genome^50^ offers an excellent opportunity for genome-wide identification, evolution and functional analysis of the RING-containing protein genes in this species. In present study, we first identified all the putative RING finger protein genes from the actual version of *B. rapa* genome database BRAD (http://brassicadb.org/brad/index.php). Then, we analyzed the RING domain type, additional domains, chromosomal location and expression pattern for each identified RING finger gene. We also examined the syntenic relationship of these RING finger genes between *B. rapa* and *A. thaliana*. Our study lays a foundation for further evolutionary and functional characterization of RING finger genes among *Brassica* species, and provides useful information for understanding the evolution of this gene family in all higher plants.

## Results

### Genome-wide identification of RING finger proteins in *B. rapa*

In order to identify all the RING finger proteins in *B. rapa,* two search strategies were used in the current study: first, we used the previous 469 RING proteins from *A. thaliana*^14^ as BLASTp queries to perform multiple searches against the latest whole predicted proteome of *B. rapa*; second, each type of representative *A. thaliana* RING domain was used as a query to BLASTp against the same database of *B. rapa* genome. The retrieved non-redundant putative protein sequences were then subjected to SMART database for domain analysis and subsequently to manual inspections of the presence or not of the eight conserved metal ligands. Consequently, we identified a total of 715 predicted *B. rapa* RING finger proteins containing 731 RING domains: 699 predicted proteins contain single RING domain, while 16 other proteins contain double RING domains. For 19 of 731 identified RING domains, one of the eight metal ligands was modified, in each case, by a new residue instead of the Cys or His (Table S1). According to the amino acid residues at eight metal ligand positions and the distance between them, and also taking into account the classification of the corresponding ortholog (if exists) in *A. thaliana* as well as the phylogenetic analysis result of this study for those with one of the eight metal ligands modified, the 731 RING domains from 715 RING proteins were classified into eight RING types: RING-H2 (371), RING-HCa (215), RING-HCb (47), RING-v (44), RING-C2 (38), RING-D (10), RING-S/T (5) and RING-G (1). The characteristic structure of each RING type with the 8 metal ligands coordinating two zinc atoms in a cross-braced manner is illustrated in Table 1. All these eight RING types were previously described in *A. thaliana*^14^. We also identified 11 proteins that were detected as RING domain containing by SMART but were classified as incomplete RING domain containing due to the modification or absence of two or more metal ligands (Table S2).

Of the 731 RING domains identified in *B. rapa*, RING-H2 represents the first most common type with 371 domains (50.8%), while RING-HC represents the second most common RING type with 262 domains (35.8%), RING-H2 and RING-HC types account for 86.6% of the total RING domains in *B. rapa* (Table 1). The 262 RING-HC domains can be further divided into two sub-types RING-HCa (215) and RING-HCb (47) based on the spacing between ml7-ml8^17^. The third common RING type is RING-v with 44 (6.0%) domains, which differs from RING-H2 in that it has a Cys residue at ml4 instead of His and a larger spacing of 7 amino acids instead of 2 between ml4-ml5. It can be distinguished from PHD domain by differed spacing features both between ml3-ml4 (1 amino acid for RING-v contrasting 2–4 for PHD domain) and ml4-ml5 (7 amino acid for RING-v contrasting 4–5 for PHD domain)^10^. The forth common RING type is RING-C2 with 38 (5.2%) domains, which differs from RING-HC in that it has a Cys residue instead of His at ml4 and a larger spacing of 4 or 5 amino acids instead of 2 or 3 between ml4-ml5. *B. rapa* has 10 RING-D domains which differ from RING-HC by an Asp residue at ml5 or ml6 instead of a Cys, 5 RING-S/T domains which differ from RING-HC by a Ser or Thr residue at one or both ml2 or ml6 instead of a Cys, and 1 RING-G domains which differ from RING-HC by a Gly residu at ml5 instead of a Cys (Table 1).

### Spacing conservation between metal ligands in *B. rapa* RING domains

**Figure 1:**
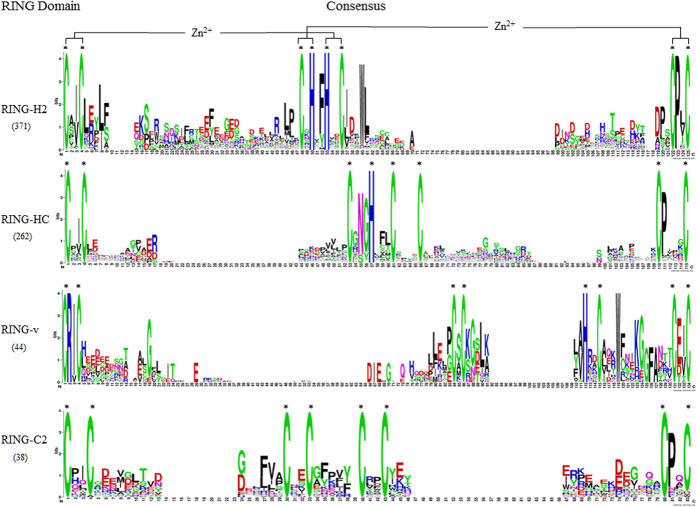
Sequence logo of the overrepresented motif found in *B. rapa* RING-H2, RING-HC, RING-v and RING-C2 domains, respectively. The asterisked letters of Cys and/or His indicate conserved metal ligands and zinc-coordinating amino acid pairs are shown. The figures were created by on-line WebLogo tool (http://weblogo.berkeley.edu/logo.cgi). The height of the letters is proportional to their frequency of the corresponding amino acid at that position.

Previous studies showed that the RING domains form a cross-brace structure, in which metal ligand pairs ml1-ml2 and ml5-ml6 chelate one zinc atom, and ml3-ml4 and ml7-ml8 chelate another one^13^ (Fig. 1). Such a structure requires that the spacing between ml1-ml2, ml3-ml6, and ml7-ml8 should be conserved, while the spacing between ml2-ml3 and ml6-ml7 might be varied to a certain degree. Tables 1, 2, 3 and 4 summarized the spacing variation between the different metal ligands of each of the 8 identified RING types in *B. rapa*. We can observe that all the 731 *B. rapa* putative RING domains (100%) had two amino acids between ml1-ml2, while 99.5% (727/731) had two amino acids between ml5-ml6, 93.2% (681/731) had 1 amino acids between ml3-ml4, 93.6% (684/731) had two amino acids between ml7-ml8, and 80.0% (584/731) had two amino acids between ml4-ml5 (Table 2). The spacing between ml2-ml3 is ranged from 9 to 77 residues with the highest frequency around 10–16 (Table 3), while the spacing between ml6-ml7 is ranged from 4 to 64 residues with the highest frequency around 6–16 (Table 4).

Interestingly, the RING domains with the same metal ligands tend to have the same spacing between metal ligands. The RING-H2 domains preferred a spacing of 14 (125/371) or 15 (181/371) residues between ml2-ml3 and 10 (287/371) or 11 (32/371) between ml6-ml7, while the RING-HCa preferred a spacing of 11 (142/215) or 10 (34/215) between ml2-ml3. The RING-HCb domains were differed from the rest by a spacing of 4 (43/47) or 3 (4/47) instead of 2 residues between ml7-ml8. The RING-v domains were characterized by a unique spacing of 7 (44/44) residues between ml4-ml5 and preferred a spacing of 12 (11/44) or 13 (10/44) residues between ml2-ml3, and 12 (38/44) or 15 (5/44) between ml6-ml7. The RING-C2 domains preferred a spacing of 4 (28/38) or 5 (8/38) residues between ml4-ml5, 15 (14/38) or 13 (9/38) between ml2-ml3, and 11 (15/38) or 16 (11/38) between ml6-ml7. So, in addition to sharing amino acid conservation, the RING domains of same type also tend to share the size of the inter-metal ligand region. As the spacing between ml4-ml5, ml2-ml3 and ml6-ml7 determined the distance between the two zinc-binding sites^10^,^13^, the spacing variations observed in these three metal ligand intervals among the 731 RING domains may reflect the 3-D structural diversity of RING domains in *B. rapa*. These main features of conserved spacing between metal ligands observed among *B. rapa* RING domains confirm the previous observations on the RING domains of *A. thaliana*^14^, apple^17^, rice^15^ and *O. tauri*^18^.

### Conservation of other residues in *B. rapa* RING domains

The previous studies^14^,^15^,^17^,^18^ showed there exist other conserved amino acid residues in addition to the conserved 8 metal ligands in different types of RING domains. In order to inspect these conserved residues in *B. rapa* RING domains, sequence alignments of the 4 major RING types, RING-H2, RING-HC, RING-v and RING-C2, were respectively performed (Figs S1–S4), and sequence logos of the over-represented residues found in each of the 4 major RING types were presented in Fig. 1. We can observed the following features that were previously described in other plant species: an Ile or Val precedes ml2 in the majority of RING-H2, RING-HC and RING-v; more than 80% of all RING-H2 domains have a Phe or Tyr residue in front of ml5; RING-v domain has an Ala or Val in front of ml5; a Trp residue is usually found at the fourth position after ml6 for RING-H2 and RING-v; a Pro residue is usually found right after ml7 in RING-H2, RING-HC and RING-C2 but not in RING-v; a Glu or Asp followed by an Ile or Val is usually found between ml7-ml8 in RING-v; an Arg is always present just after ml1 in RING-v; the motif of C-x3-[W]-x3-[KG]-x6-C is usually found between ml6-ml7 in RING-v. In addition to these previously described features, we observed a few new conserved residues in *B. rapa* RING domains: in RING H2, a Leu residue at the first and fourth, and a Glu or Arg at the second position following ml2, an Ile following ml6, a Leu at the fifth position following the ml6; in RING-HC, an Asn and an Gly precede the ml4 when the spacing between ml3-ml4 is 3 residues; in RING-v, a Pro, Gly or Glu precedes ml3, a Ser between ml3-ml4, a Lys at the first, a Gly at the second and a Leu at the fourth position after the ml4, an Ala or Val precedes ml5, etc.

### Phylogenetic analysis of the *B. rapa* RING domains

In order to classify the 731 RING domains identified in *B. rapa*, a multiple sequence alignment of all identified RING domains was first conducted using the ClustalW program and edited manually with BioEdit software to align correctly the 8 metal ligand positions (Fig. S5), from which a phylogenetic tree was then generated (Fig. S6). The results showed that the domains of similar RING type tend to be clustered together but no large clade was observed within each RING type. The phylogenetic relationship between the four main RING types (RING-H2, RING-HC, RING-v and RING-C2) cannot be unambiguously determined by the tree because of the low bootstrap values for the relevant tree nodes. The RING-HCb domains, although clustered into smaller groups, cannot be clearly separated from RING-HCa. Curiously, three RING-HCa domains (Bra006081-HCa, Bra012331-HCa and Bra016082-HCa) were closely associated with RING-H2 members and separated into the RING-H2 group, whereas no RING-H2 domain was clustered into RING-HC. Of the 10 members of RING-D, 5 were clustered together and placed between the RING-HC and RING-H2 groups, while 2 were separated into RING-HC and 3 other ones into RING-H2. All the five RING-S/T members were grouped into the RING-HC group. The unique RING-G member was associated with RING-D domains and placed between the RING-HC and RING-H2 groups. Finally, the tree showed a large number of small clades containing 2–5 members (one with 10 members) indicating the duplication of these RING domain containing genes in *B. rapa* genome.

### Additional domains in the RING protein genes

To better classify the *B. rapa* RING containing proteins, we examined the full length sequence of all the identified 715 RING domain containing proteins by SMART and identified 62 other previously identified domains associated with the RING domain. According to the domain presence and organization, we grouped and sub-grouped the similar proteins due to sharing of same features, and further classified the 715 *B. rapa* RING proteins into 51 groups (Table S3). The first largest group includes 234 members containing no additional domain outside the RING domain. The second largest group includes 200 members containing each one or more trans-membrane domains. The third largest group includes 29 members containing each one or more coiled-coil domains. The remaining groups or sub-groups include generally 1–19 members containing one or more additional known domains. Some of these additional domains were predicted to be protein binding domains which may participate in substrate recognition of E3 ligase, such as the coiled-coil domain, Vwaint, Ankyrin repeats, TPR, BRCT, CRA, SPRY and WD40, other ones were predicted to be associated with ubiquitination, such as CUE, ZNF_UBP, SINA, GIDE, RWD and Ufd2P_corr. A few additional domains, such as ZNF_C2H2, ZNF_C3H1, RRM, HIRAN, HILICc, DEXDc and BRAP2, were predicted to be nucleic acid-binding. Six types of Zinc finger (zinc ion binding function) were found to be associated with RING domain: ZNF_C2H2, ZNF_C3H1, ZNF_CHY, ZNF_RBZ, ZNF_UBP and ZNF_ZZ. The previously identified domains such as Hemathrine, Pro-CA, Pep3_Vps18, Zinc_Ribbon_6, Zinc_Ribbon_9, S4, EFH, SPRY, FBD, PA, IBR, DUF1232, DUF3675, DUF1117 and DUF269, were also found to be associated with RING domains. One domain (FBD) associating with the RING domain was shown to be specific to *Brassica* species, while most of these additional domains are simultaneously found in various plant species such as *A. thaliana, B. rapa, Oryza sativa, Vitis vinifera, Malus domestica, Zea mays*, etc., suggesting that their function might be conserved between these species (Table S3).

### Syntenic relationships between RING finger genes of *B. rapa* and *A. thaliana*

*B. rapa* was a paleohexaploidy with three subgenomes which share the same diploid ancestor of the model species *A. thaliana*^50^. According to the degree of biased gene fractionation (gene losing), the three subgenomes of *B. rapa* can be classified as the least fractionized (LF), the moderately fractionized (MF1) and the most fractionized (MF2) subgenomes^50^,^51^. For each *B. rapa* RING finger gene, we identified its syntenic paralogs on three subgenomes of *B. rapa* as well as its corresponding orthologs in *A. thaliana* from the database BRAD by Search Syntenic Gene function. By a similar way, we also used the previously identified 469 *A. thaliana* RING finger genes^14^ to identify their corresponding *B. rapa* orthologs from the same database. The collected data were summarized in Table S4. 85.9% (614/715) of *B. rapa* RING finger genes found their orthologs in *A. thaliana*, while 14.1% (101/715) didn’t found their orthologs in *A. thaliana*. On the other hand, this analysis allowed us to obtain a revised list of 502 RING finger genes in *A. thaliana* (Table S4). 18.5% (93/502) of *A. thaliana* RING finger genes didn’t found their orthologs in *B. rapa*. Analysis showed that the 614 *B. rapa* RING finger genes as well as their corresponding *A. thaliana* orthologs were derived from 24 blocks of all the 7 ancestral chromosomes of translocation Proto-Calepineae Karyotype (tPCK) of *Brassica* species^51^,^52^,^53^,^54^. Among the 715 *B. rapa* RING finger genes, 330 (46.2%) were located on LF subgenome, 223 (31.2%) were located on MF1 subgenome and 162 (22.6%) were located on MF2 subgenome. In 50 cases, the three copies were well conserved on the three subgenomes, while in 146 cases, only two of the three copies were present, and in 294 cases, only one copy was present. A total of 57 *B. rapa* as well as 58 *A. thaliana* RING finger genes were involved in tandem repeat. In 16 cases, the expected RING domains were not detected in the corresponding *A. thaliana* orthologs while their *B. rapa* counterparts were characterized as RING domain containing proteins; and in 57 cases, the corresponding *A. thaliana* orthologs were RING domain containing proteins but their *B. rapa* counterparts were characterized no RING domain containing (Table S4).

### Chromosomal location of RING finger genes on *B. rapa* genome

**Figure 2:**
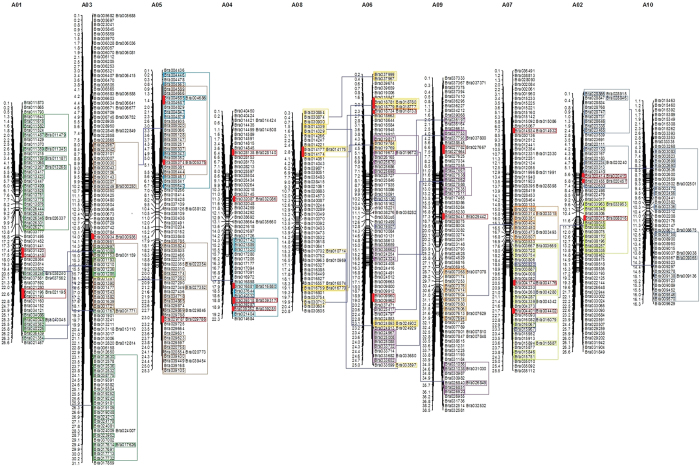
Distribution of 700 *RING* finger protein genes on 10 chromosomes of *B. rapa*. The 700 *BrRING* genes unevenly located on each conserved collinear blocks of the chromosomes. Chromosome number (A01-A10) is indicated at the *top* of each chromosome. Gene name is indicated on the *right side* of each chromosome. The physical position (Mb) of each mapped gene is indicated on the *left side* of each chromosome. The genes located on duplicated chromosomal segments are framed by same colors and connected by *blue lines* between the two relevant chromosomes. The tandem repeated genes are marked by red color on the different chromosomes.

To localize each of the 715 identified RING protein genes on *B. rapa* genome, we firstly retrieved their chromosome location data from BRAD database. In the currently released *B. rapa* genomic sequences, 700 RING finger genes were mapped to 10 chromosomes while the remaining 15 RING genes were not mapped to specific chromosome due to their localization on isolated scaffolds (Table S1). Our chromosomal mapping results showed that these RING finger genes were distributed across all the 10 chromosomes of *B. rapa* with a variable intensities (Fig. 2): 113 RING genes were detected on chromosome A03, 84 on chromosome A09, 80 on chromosome A07, 75 on chromosome A05, 72 on chromosome A06, 63 on chromosome A02, 62 on chromosome A01, 53 on chromosome A04, 49 on chromosome A08, and 49 on chromosome A10. Our gene duplication analysis showed that both the segmental duplication and tandem repeats have contributed to the expansion of the *B. rapa* RING finger gene family (Table S4, Fig. 2).

### Expression analysis of *B. rapa* RING finger genes in different tissues

**Figure 3:**
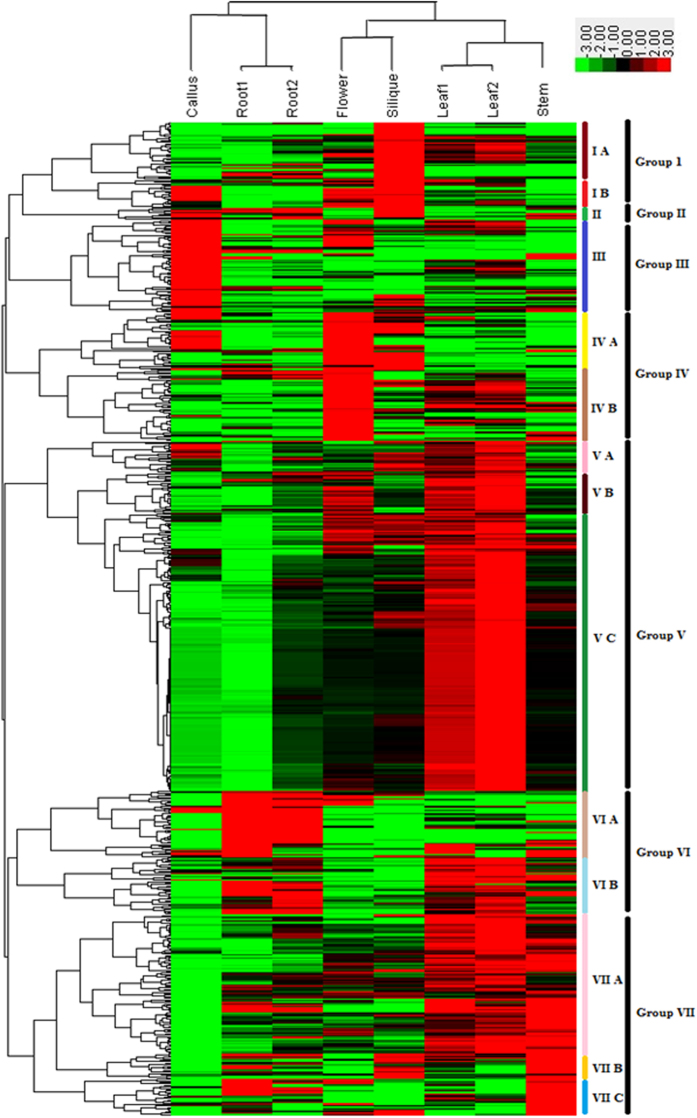
Expression profile of 667 *B. rapa* RING finger genes in different tissues revealed by RNA-seq data. The 667 genes were divided into groups (I-VII) and subgroups. The *scale* representing the relative signal values is shown above. The tissue types are indicated on the *top*. The individual gene names as well as their RING types are indicated in the Fig. S7.

To investigate the expression diversity and evolutionary fate of the RING finger genes in *B. rapa*, we used a *B. rapa* RNA-seq transcriptomic dataset downloaded from GEO database (GSE43245) to retrieve the expression patterns of each *B. rapa* RING finger gene in six major organs or tissues (callus, root, stem, leaf, flower and silique). The expression data of 673 *B. rapa* RING finger genes were identified from the dataset, of which 2 RING-H2 (Bra030136 and Bra034259) and 4 RING-HCb (Bra001543, Bra005642, Bra011996 and Bra027352) showed a value of zero for all the tested tissues and were excluded from the analysis. The remaining 667 RING finger genes were then classified into seven groups (I-VII) based on the hierarchical clustering of their expression patterns (Fig. 3, Fig. S7). The group I includes 58 genes which were all preferentially (>2-folds higher) expressed in silique and could be further divided into two subgroups: I-A (39 genes), and I-B (19 genes). The group II includes 8 genes of which 87.5% were preferentially expressed in silique, 75% in root, 62% in callus and 62% in stem. The group III includes 63 RING genes of which 100% were preferentially expressed in callus, 31% in leaf, 30% in flower, 22% in stem, 19% in silique, and 14% in root. The group IV includes 87 genes which were all preferentially expressed in flower and can be further divided into two subgroups: IV-A (39 genes) and IV-B (48 genes). The group V includes 231 RING finger genes which were all preferentially expressed in leaf and can be further divided into three subgroups V-A (21 genes), V-B (28 genes) and V-C (182 genes). The group VI includes 84 genes of which more than 80% were preferentially expressed in root and can be further divided into two subgroups: VI-A (45 genes) and VI-B (39 genes). The group VII includes 136 RING genes of which more than 95% were preferentially expressed in stem and can be further divided into three subgroups: VII-A (94 genes), VII-B (17 genes) and VII-C (25 genes).

To determine if there is a correlation between the structure categories of RING domains and expression patterns of the 667 RING finger genes in *B. rapa*, we calculated the percentages of genes per the total genes of each RING type in each expression group (Table 5). We can observe that 34.8% of RING-H2, 28.2% of RING-HCa, 52.2% of RING-HCb, 44.7% of RING-v, 34.4% of RING-C2 and 83.3% of RING-D genes shared similar expression patterns and were classified in the expression group V, while 15.9% of RING-H2, 23.8% of RING-HCa and 53.1% of RING-C2 genes shared expression patterns of group VII.

## Discussion

*B. rapa* is a mesopolyploid crop that has undergone the whole genome triplication (WGT) event since its divergence from *A. thaliana*^50^,^55^. About 500 RING finger genes were identified in the *Arabidopsis* genome (Table S4); therefore, up to 1500 RING finger genes could be produced by the WGT event in the *B. rapa* genome. However, only 715 RING finger genes were identified in the *B. rapa* genome (Table S1), suggesting that more than 50% of duplicated RING finger genes were either lost or fixed by nonfunctionalization (silencing) after WGT^56^,^57^,^58^. Similar results were also observed for other gene families in *Brassica* species^58^,^59^. In fact, our analysis of syntenic relationships between RING finger genes of *B. rapa* and *A. thaliana* showed that, only in 50 cases, the triplicated copies were well retained on all the three subgenomes (LF, MF1 and MF2) of *B. rapa*, while in 146 cases, only two of the three triplicated copies were retained, and in 294 cases, only one of the three triplicated copies was retained in *B. rapa* genome. On the other hand, 93 *A. thaliana* RING finger genes didn’t find their corresponding orthologs in the *B. rapa* genome, while 101 *B. rapa* RING finger genes didn’t find their orthologs in the *A. thaliana* genome, indicating that there were RING finger gene losses/gains in both species during the evolution of their genome. We also found that, in 57 cases, the corresponding *A. thaliana* orthologs were RING domain containing but their *B. rapa* counterparts were no RING domain containing; while in 16 cases, the corresponding *A. thaliana* orthologs were no RING domain containing but their *B. rapa* counterparts were RING domain containing. In addition, we also identified a few *B. rapa* proteins contain modified (Table S1) or incomplete (Table S2) RING domains. These findings imply the diversity and evolution dynamics of RING finger gene family among the Brassicaceae species. These species-specific RING finger genes may serve as targets for studying the phylogenetics and character/trait evolution in the Brassicaceae.

Our study of the 715 RING finger proteins in *B. rapa* confirms the common features previously observed in other species^10^,^14^,^15^,^17^,^18^: similar proportions of the main RING types, conserved spacing between metal ligands and presence of other conserved residues in addition to the eight metal ligands, etc. Interestingly, the numbers of RING-D and RING-G domains were identical between *A. thaliana* and *B. rapa*, but the number of RING-C2 domains is tri or quatri-plicated (from 10 to 38) in the *B. rapa* genome (Table 1). Analysis of additional domains allowed to divided the 715 RING proteins into 51 groups (Table S3), compared with the 30 groups identified in *A. thaliana*^17^. This increased number of groups implies that the *B. rapa* RING finger protein family comprise the proteins with more diversified functions than those of *A. thaliana*.

Our analysis on RNA-seq data showed that at least 94.7% (667) of the 715 identified RING finger genes expressed as RNA in *B. rapa*, and that the majority of them expressed preferentially in one or few specific tissues: 58 genes preferentially expressed in silique, 8 preferentially expressed in silique, root, callus and stem, 63 preferentially expressed in callus, 87 preferentially expressed in flower, 231 preferentially expressed in leaf, 84 preferentially expressed in root, and 136 preferentially expressed in stem (Fig. S7). These results are indicative of functional diversification of the RING finger gene family and their involvement in all the stages of plant growth and development in *B. rapa*. Leaf heads of Chinese cabbage (*B. rapa*) and cabbage (*Brassica oleracea*) are important vegetables that supply mineral nutrients, crude fiber and vitamins in the human diet. The leaf-related traits, such as head size, head shape, head weight and heading time, contribute to yield and quality^60^. Our study showed that more than 30% of *B. rapa* RING finger protein genes expressed preferentially in leaf, suggesting that they are probably involved in the growth and leafy head formation in *B. rapa*. They constitute a panel of gene candidates for studying the genetic and molecular mechanisms of leafy head traits in different *Brassica* species. Further studies on the responses of these *B. rapa* RING finger genes to abiotic stresses (such as salinity, drought, cold, etc.) or phytohormone treatments (such as ABA, IAA, GA3, etc.) will provide more insights about the functions and regulation mechanisms of these RING finger genes in plant growth and development.

In conclusion, a total of 715 RING finger protein genes were identified in *B. rapa* genome. The classification of these genes by RING domain type, additional domain and expression pattern, etc., provides valuable information for further studies on the biological functions of each RING finger protein gene in *B. rapa*. Our study will serve as a useful reference for comparative analyses of RING finger protein gene family in *Brassica* species and help to select the appropriate candidate genes for further functional characterizations, genetic engineering and genetic improvement of *Brassica* crops.

## Materials and Methods

### Identification of RING finger proteins in *B. rapa*

We identified the RING finger proteins in *B. rapa* using two different approaches. First, all 469 known RING-containing proteins in *A. thaliana*^17^ were retrieved from the TAIR database (http://www.arabidopsis.org/) and used as queries to BLASTp against the latest version of the whole *B. rapa* genome annotation data deposited at the Brassica Database (BRAD, ver. 1.5, http://brassicadb.org/brad/). Second, each type of representative *A. thaliana* RING domains was used as queries to BLASTp against the same database in order to fully identify the RING finger proteins. In both cases, the retrieved irredundant sequences were submitted to SMART database (http://smart.embl-heidelberg.de/) with chosen option of Pfam domains to confirm the presence of RING domains, combined by manual inspection of each protein sequence based on the conservation of eight metal ligands (His or Cys) and the residue number between two neighboring metal ligands. We determined the RING type for each identified *B. rapa* RING domain, according to the specific amino acid residues at different metal ligand positions and distances between metal ligands, and also taking into account the classification of the corresponding ortholog (if exists) in *A. thaliana* as well as the phylogenetic analysis result of this study for those with one of the eight metal ligands modified. Those proteins that were predicted as RING domain containing by SMART but lacked two or more metal ligands, were classified as incomplete RING domain containing.

### Multiple sequence alignment and phylogenetic analysis

The RING domain sequences were first extracted from the identified irredundant RING finger protein sequences then aligned using ClustalW program and edited manually with BioEdit software to align correctly the 8 metal ligand (ml) positions. For the alignment of the total identified *B. rapa* RING domains, after a primary alignment followed by manual editions, the internal sequences of ml2-ml3 and ml7-ml8 were independently extracted from each RING domain, aligned separately by using the same ClustalW program in BioEdit, and the resulted sub-alignments were re-inserted into the appropriate intervals of the initial alignment. Based on this improved alignment of the total *B. rapa* RING domains, a phylogenetic tree was generated with MEGA6.06 using the Neighbor–Joining (NJ) algorithm. Bootstrap analysis with 1,000 replicates was used to evaluate the significance of the nodes. Pair wise gap deletion mode was used to ensure that the divergent domains could contribute to the topology of the NJ tree.

### Identification of additional domains in RING domain containing *B. rapa* proteins

The RING finger protein sequences were submitted to SMART and occasionally to Interpro (http://www.ebi.ac.uk/interpro/) databases to detect any additional known domains. According to the presence or not and organization of specific additional domain(s), the RING finger proteins were manually divided into different groups. For each group, the representative sequences were BLASTp against the NCBI databases to identify the orthologous proteins with a conserved architecture of RING + additional domain in other species.

### Syntenic relationships between the RING finger protein genes of *B. rapa* and *A. thaliana*

For each identified *B. rapa* RING finger protein gene, we used the Search Syntenic Gene function of the BRAD database^51^ to determine its *A. thaliana* ortholog (if existed). On the other hand, for each *A. thaliana* RING finger protein gene, we used the same function to determine its *B. rapa* ortholog(s) (if existed). In each query, the information about the localization on tPCK (Translocation Proto-Calepineae Karyotype) chromosomes and ancestral chromosome block, the corresponding ortholog(s) in *A. thaliana*, LF (the least fractioned subgenome), MF1 (the medium fractionated subgenome) and MF2 (the most fractionated subgenome)^51^,^52^,^53^,^54^ as well as the eventual tandem repeated gene(s) were recorded.

### Chromosome location of the *B. rapa* RING finger protein genes

The chromosome location data (start-stop) of each identified RING finger protein gene were retrieved from BRAD database by using the Search Gene Sequence function. The Genes who were assigned to unassembled genomic scaffolds (no chromosomal location information) were not included in the analysis. The genes were mapped to the chromosomes by using the software Map Chart 2.3 v and the physical location values (median values). The tandem repeated genes as well as the segmental duplicated genes that were revealed by the analysis of syntenic relationships between the RING finger protein genes of *B. rapa* and *A. thaliana* were indicated on the map by lines of different colors manually drawn.

### Expression analysis of RING domain-containing genes in different tissues of *B. rapa*

The RNA-seq data of gene expression of six tissues (callus, root, stem, leaf, flower, and silique) of the *B. rapa* ssp. *pekinensis* line Chiifu-401–42 was retrieved from the Gene Expression Omnibus (GEO) database of NCBI (http://www.ncbi.nlm.nih.gov/geo/) using the accession number GSE43245. The expression profiles (FPKM values) of *B. rapa* RING finger protein genes were extracted from the data set and clustered using Cluster software (Version 3.0, http://rana.lbl.gov/EisenSoftware.htm) with uncentered Pearson’s correlation distances and the complete linkage method for hierarchical clustering. The Java Tree view software (Version1.1.5r2, http://jtreeview.sourceforge.net/) was used for constructing and viewing the clustering tree of *B. rapa* RING finger protein genes.

## Additional Information

**How to cite this article**: Alam, I. *et al*. Genome-wide identification, evolution and expression analysis of RING finger protein genes in *Brassica rapa. Sci. Rep.* **7**, 40690; doi: 10.1038/srep40690 (2017).

**Publisher's note:** Springer Nature remains neutral with regard to jurisdictional claims in published maps and institutional affiliations.

## Supplementary Material

Supplementary Figures

Supplementary Tables

## Figures and Tables

**Table 1:** The types and characteristics of RING domains in *B. rapa*.

RING domain		Consensus														
Type	No.	ml1		ml2		ml3		ml4		ml5		ml6		ml7		ml8
RING-H2	371	C	x_2_	C	x_11-43_	C	x_1_	H	x_2_	H	x_2_	C	x_6-64_	C	x_2_	C
RING-HCa	215	C	x_2_	C	x_10-45_	C	x_1,3_	H	x_2,3_	C	x_2-4_	C	x_6-43_	C	x_2_	C
RING-HCb	47	C	x_2_	C	x_8-16_	C	x_1_	H	x_2_	C	x_2_	C	x_5-30_	C	x_3,4_	C
RING-v	44	C	x_2_	C	x_11-77_	C	x_1_	C	x_7_	H	x_2_	C	x_12-30_	C	x_2_	C
RING-C2	38	C	x_2_	C	x_10-16_	C	x_1,2_	C	x _2-6_	C	x_2_	C	x_10-36_	C	x_2_	C
RING-D	10	C	x_2_	C	x_12-15_	C	x_1_	H	x_2_	C/D	x_2_	C/D	x_10_	C	x_2_	C
RING-S/T	5	C	x_2_	C/S	x_12-16_	C	x_1_	H	x_2,3_	C	x_2_	S/T	x_4-14_	C	x_2_	C
RING-G	1	C	x_2_	C	x_16_	C	x_1_	H	x_2_	G	x_2_	C	x_13_	C	x_2_	C

The eight conserved metal ligands (ml) in canonical and modified RING domains are shadowed in bleu. X_(n)_, number of amino acids between metal ligands.

**Table 2:** Spacing variation between metal ligand (ml) pairs ml1-ml2, ml3-ml4, ml4-ml5, ml5-ml6 and ml7-ml8 in the different types of *B. rapa* RING domains.

ml pairs		ml1-x_n_-ml2	ml3-x_n_-ml4			ml4-x_n_-ml5						ml5-x_n_-ml6			ml7-x_n_-ml8		
No. of amino acid (n)		2	1	2	3	2	3	4	5	6	7	2	3	4	2	3	4
No. of RING domains	RING-H2	371	371			371						371			371		
	RING-HCa	215	191		24	151	64					211	1	3	215		
	RING-HCb	47	47			47						47				4	43
	RING-v	44	44								44	44			44		
	RING-C2	38	12	26		1		28	8	1		38			38		
	RING-D	10	10			10						10			10		
	RING-S/T	5	5			3	2					5			5		
	RING-G	1	1			1						1			1		
Total No.		731	681	26	24	584	66	28	8	1	44	727	1	3	684	4	43

X_(n)_, number of amino acids between metal ligands.

**Table 3:** Spacing variation in the loop between metal ligand (ml) ml2-ml3 in the different types of *B. rapa* RING domains.

ml pair		ml2-x_n_-ml3																								
No. of amino acids (n)		9	10	11	12	13	14	15	16	17	18	20	21	22	24	25	28	29	30	42	43	48	56	64	71	77
No. of RING domains	RING-H2		1	4	1	2	125	181	27	3	6	3	2	1		2	7	3	2		1					
	RING-HCa	1	34	142	14		3	9	2	1		6	1	1								1				
	RING-HCb			5	2	15	11	11	3																	
	RING-v			3	11	10	6	2	2		3				1	1				1			1	1	1	1
	RING-C2	1	6	2	1	9	2	14	2							1										
	RING-D				2		5	3																		
	RING-S/T				1		3	1																		
	RING-G								1																	
Total No.		2	41	156	32	36	155	221	37	4	9	9	3	2	1	4	7	3	2	1	1	1	1	1	1	1

X_(n)_, number of amino acids between metal ligands.

**Table 4:** Spacing variation in the loop between metal ligand (ml) ml6-ml7 in the different types of *B. rapa* RING domains.

ml pair		ml6-x_n_-ml7																												
No. of amino acids (n)		4	6	7	8	10	11	12	13	14	15	16	17	18	19	20	21	22	23	24	25	26	27	29	30	31	36	43	54	64
No. of RING domains	RING-H2			3	3	287	32	13	4	15	2	1		2			2		1	2					1				1	2
	RING-HCa	1	39	28	2	56	18	19	7	1	4	3		1	1	3	12	1	4	2	3	1		3	1		4	1		
	RING-HCb			1		2	4			2		15	5	7	8	1		1							1					
	RING-v							38			5														1					
	RING-C2					5	15	1	1		2	11											1			1	1			
	RING-D					10																								
	RING-S/T	1		1						2		1																		
	RING-G								1																					
Total No.		2	39	33	5	360	69	71	13	20	13	31	5	10	9	4	14	2	5	4	3	1	1	3	4	1	5	1	1	2

X_(n)_, number of amino acids between metal ligands.

**Table 5:** Expression diversity of different types of RING finger protein genes in *B. rapa.*

Expression group^a^	No. of genes	RING-H2	RING-HCa	RING-HCb	RING-v	RING-C2	RING-D	RING-S/T	RING-G
I	58	30 (8.8)^b^	20 (9.7)	1 (2.5)	6 (15.8)	1 (3.1)	0 (0.0)	0 (0.0)	0 (0.0)
II	8	4 (1.2)	3 (1.5)	1 (2.5)	0 (0.0)	0 (0.0)	0 (0.0)	0 (0.0)	0 (0.0)
III	63	41 (12.1)	16 (7.8)	1 (2.5)	3 (7.9)	1 (3.1)	1 (16.7)	0 (0.0)	0 (0.0)
IV	87	39 (11.5)	37 (18.0)	3 (7.5)	4 (10.5)	2 (6.3)	0 (0.0)	2 (40.0)	0 (0.0)
V	231	118 (34.8)	58 (28.2)	21 (52.5)	17 (44.7)	11 (34.4)	5 (83.3)	1 (20.0)	0 (0.0)
VI	84	53 (15.6)	23 (11.2)	7 (17.5)	1 (2.6)	0 (0.0)	0 (0.0)	0 (0.0)	0 (0.0)
VII	136	54 (15.9)	49 (23.8)	6 (15.0)	7 (18.4)	17 (53.1)	0 (0.0)	2 (40.0)	1 (100.0)
Total No.	667	339	206	40	38	32	6	5	1

^a^The expression groups were defined based on the hierarchical clustering of the expression patterns of 667 RING finger protein genes as shown in Fig. 3 and Fig. S7;

^b^values in parenthesis indicate the percentages of genes per total genes of each RING type in each expression group.
