# Supplementary material for: Genome-wide identification, evolution and expression analysis of RING finger protein genes in Brassica rapa
Source: Sci Rep. 2017 Jan 17;7:40690. doi: 10.1038/srep40690 (PMC5240574; doi:10.1038/srep40690)
Supplement: Supplementary Figures [file srep40690-s1.pdf]

Supplementary Information

Genome-wide identification, evolution and expression analysis of RING finger protein genes in *Brassica rapa*

Intikhab Alam, Yan-Qing Yang, Yong Wang, Mei-Lan Zhu, Heng-Bo Wang, Boulos Chalhoub, Yun-Hai Lu*

Figure S1 Multiple sequence alignment of 371 RING-H2 domains of *B. rapa*.

```
                       *  *                                           * *  *  *                                                                 *  *
Bra000007-H2           CTVCL----------------------SVFGDGE------EIRRLSACKHAFHVSCIETWLK-------------------------------------------DHP-N-----------CPIC
Bra000143-H2           CAVCK----------------------ENFELKS-----AARE--MPCNHIYHPDCILPWL--------------------------------------------AIRNS-----------CPVC
Bra000207-H2           CAVCK----------------------DEFELGT-----EAKQ--MPCNHIYHSDCIVPWL--------------------------------------------VQHNS-----------CPVC
Bra000350-H2           CAICR----------------------EDFVIGE-----SARR--LPCDHLYHHDCITPWL--------------------------------------------TSHNT-----------CPLC
Bra000673-H2           CAICL----------------------NEFDDEE-----TL-RWMPPCSHTFHASCIDVWLS--------------------------------------------SWST-----------CPVC
Bra000683-H2           CAICI----------------------EDYRIGD------KLRILP-CNHKFHVGCVDLWLGQ-------------------------------------------RRSF-----------CPVC
Bra000742-H2           CTVCL----------------------SDFESDD-----NI-RQLPNCRHVFHDHCLDRWI------------------------------------------VDCRKMT-----------CPIC
Bra000951-H2           CSICY----------------------EDLKPV-----AENLQSISACGHVFHELCLQQWFEYCPS---------------------------------------TNKRN-----------CPIC
Bra001080-H2           CSVCL----------------------SEFQEEE-----RL-RMLPKCSHAFHVACIDTWLK--------------------------------------------SHSN-----------CPLC
Bra001153-H2           CSICL----------------------DTVAKDGD-----RAWANLQCGHQFHLDCIGSAFNA------------------------------------------KGVMQ-----------CPNC
Bra001381-H2           CPVCQ----------------------DRFEVGS-----VARK--LPCKHIYHSECIIPWL--------------------------------------------IQRNT-----------CPVC
Bra001390-H2           CCICL----------------------GGLEEGE-----KMKV-LPMCRHCYHCECVDRWL--------------------------------------------MTESS-----------CPLC
Bra001582-H2           CTICQ----------------------ETYKNRD------KIAMLD-CKHEYHPACLEKWLV--------------------------------------------IKNI-----------CPIC
Bra001639-H2           CAVCL----------------------SEFEETE-----SG-RVLPGCKHTFHVDCIDMWFH--------------------------------------------SHST-----------CPLC
Bra001721-H2           CVICL----------------------SDFVAGE-----QI-RLLPKCNHGFHVRCIDKWLT--------------------------------------------QQMT-----------CPKC
Bra001767-H2           CAVCM----------------------DEFEDGG------DVKQMP-CKHVFHQDCLMPWL--------------------------------------------ELHNS-----------CPVC
Bra002061-H2           CAICL----------------------TEFADGE-----EI-RVLPLCGHSFHVACIDKWLV--------------------------------------------SRSS-----------CPSC
Bra002084-H2           CSVCL----------------------QDFQLGE------TVRSLPHCHHMFHLPCIDKWL--------------------------------------------LRHGS-----------CPMC
Bra002147-H2           CGVCKKLLS-----QKSPWCSHKILRSGDMPAAG----------VFPCHHVYHVECL-------------------------------------------------DKVTPTSQTR--DPSCPAC
Bra002176-H2           CPICYEYLF------------------DSLKDTT----------VMKCGHTMHFECY-------------------------------------------------HEMLKRDKFC-----CPIC
Bra002336-H2           CAVCL----------------------GDLEDGD-----EV-RELRNCSHVFHRECIDRWLDYECCGG------------------------------DDNDGEEDNHRT-----------CPLC
Bra002382-H2           CSICL----------------------EPFCDSD-------PSTLTSCKHEFHLQCILEWCQ--------------------------------------------RSSQ-----------CPMC
Bra002441-H2           CPVCFEYLF------------------DSTRDIT----------VLRCGHAMHLECT-------------------------------------------------KDMGLHNRYT-----CPLC
Bra002456-H2           CAICL----------------------TAMKAGQGH-----AIFTAECSHSFHFHCITTNVQ------------------------------------------HGN-QF-----------CPVC
Bra002559-H2           CAVCT----------------------EVFEAET-----EARE--MPCKHIFHDDCITPWL--------------------------------------------SIRNS-----------CPVC
Bra002636-H2           CVICL----------------------GLWEVGD-----FG-RKLRFCGHGFHVECIDMWLS--------------------------------------------SHSS-----------CPLC
Bra002690-H2           CDLCGRDLA-----SDPERPNVSL---RSLQEVC----------VLDCGHVYHFKCL-------------------------------------------------KGTTLDLDNRSTNPSCIFC
Bra002823-H2           CSVCL----------------------DEFEKGV----EEEAKEMP-CKHKFHVKCIVPWL--------------------------------------------ELHSS-----------CPVC
Bra003083-H2           CAICL----------------------SEYEPNE------TLRTIPLCQHCFHADCIDEWLK--------------------------------------------LNGT-----------CPVC
Bra003351-H2           CTVCL----------------------EDFEP--K--E-T--VMVTPCKHMFHEECIVPWL--------------------------------------------KSKGQ-----------CPLC
Bra003404-H2           CAVCL----------------------SRFEPDD-----QL-RLLPLCCHAFHADCIDIWLV--------------------------------------------SNQT-----------CPLC
Bra003450-H2           CAVCL----------------------YEFEGEE-----EI-RWLRNCRHIFHRSCLDRW-------------------------------------------MDHDQKT-----------CPLC
Bra003507-H2           CSVCL----------------------SEFEEED-----EG-RVLPKCGHVFHVDCIDTWFR--------------------------------------------SRSS-----------CPLC
Bra003667-H2           CSLCE----------------------KEYPKEG-----EIVRKTR-CNHIFHGTCISRYLL--------------------------------------------RIPH-----------CPFC
Bra003720-H2           CVICL----------------------TEFAAGD-----EL-RVLPQCGHGFHVSCIDTWLE--------------------------------------------SHSS-----------CPSC
Bra003754-H2           CGACSKF-------EKSSVAN------FERQIAA----------VLACGHVYHAECL-------------------------------------------------ETMTTEIEKY--DLACHVC
Bra003830-H2           CSICQ----------------------EEYEGKD------EVGKLR-CGHRYHIHCAKQWLL--------------------------------------------RKNS-----------CPVC
Bra003887-H2           CPVCL----------------------NEFEDDE-----TL-RLLPKCCHVFHPGCVDAWLR--------------------------------------------SHAT-----------CPLC
Bra004008-H2           CPTCF----------------------YEYAEDN-------PKTVLQCGHIFHLACIYEWME--------------------------------------------RSAA-----------CPFC
Bra004280-H2           CCICL----------------------SSYEDGT------ELVTLP-CNHHFHSTCIEKWLK--------------------------------------------MNAT-----------CPLC
Bra004287-H2           CAICK----------------------EEFEVGE-----EGKE--LKCLHLYHTSCIVSWL--------------------------------------------NIHNT-----------CPIC
Bra004342-H2           CCICQ----------------------EGFFLG------GYAT-ITSCSHVFHSSCITDWIN--------------------------------------------KSTK-----------CPLC
Bra004436-H2           CSICL----------------------ELVVDDGS-----RSSAKLQCGHQFHLDCIGSAFNM------------------------------------------KGTMQ-----------CPNC
Bra004446-H2           CSVCL----------------------SEFEEED-----QG-RLLPKCGHAFHVDCIDTWFR--------------------------------------------SSST-----------CPLC
Bra004563-H2           CSICL----------------------CEYREAE------MLRMMPECKHYFHLCCLDAWLK--------------------------------------------LNGS-----------CPVC
Bra004589-H2           CPVCK----------------------EEFELGA-----EAKQ--MPCNHVYHSDCIVPWL--------------------------------------------VQHNS-----------CPVC
Bra004685-H2           CAVCL----------------------TLLEEKD-----TA-RMLPNCKHVFHMTCVDTWLT--------------------------------------------THST-----------CPIC
Bra004686-H2           CAVCL----------------------AFLEEKD-----TA-RMLLNCKHVFHMACVDTWLA--------------------------------------------THST-----------CPIC
Bra004687-H2           CAVCL----------------------SLLEEQD-----TA-RELPSCKHVFHVDCVDKWLT--------------------------------------------ACST-----------CPVC
Bra004847-H2           CSICL----------------------QDAAEGE-----KMRR-LTACSHCFHAGCIDPWL--------------------------------------------EKRST-----------CPLC
Bra004935-H2           CCICL----------------------CDYEDGT------ELRELS-CRHHFHEACIDKWLR--------------------------------------------INAT-----------CPLC
Bra005020-H2           CAVCK----------------------ETFILDS-----AARE--MPCNHIYHPDCILPWL--------------------------------------------AIRNS-----------CPVC
Bra005066-H2           CSICL----------------------TKMKEGGGH-----AIFTAECSHSFHFHCIASNVK------------------------------------------HGN-QV-----------CPVC
Bra005307-H2           CPICL----------------------GDYKGND------LLRQLPDCNHFFHLKCVDMWLRI--------------------------------------------NPT-----------CPVC
Bra005343-H2           CAICL----------------------SEFSDED-----TV-RLITVCRHGFHSICIDLWFE--------------------------------------------SHKT-----------CPVC
Bra005374-H2           CAVCL----------------------CEFEDEE-----TL-RLMPPCCHVFHADCVDVWLS--------------------------------------------ERST-----------CPLC
Bra005375-H2           CVVCL----------------------CEFQDND-----RL-SLMPNCCHVYHADCVSVWLS--------------------------------------------DHST-----------CPLC
Bra005859-H2           CSICF----------------------EDLSDV-----DEESIELHDCSHVFHKVCLFQWIW--------------------------------------------SKSS-----------CPLC
Bra005970-H2           CAVCK----------------------DEMGIGS-----RGVVLQLPCNHKYHGECIVPWL--------------------------------------------ETRNT-----------CPVC
Bra006070-H2           CSICQ----------------------EEYVDGD------EVGTMP-CEHMYHVSCVQQWLR--------------------------------------------MKNW-----------CPIC
Bra006321-H2           CPTCL----------------------EEYTSEN-------PKIVTKCCHHYHLSCIYEWME--------------------------------------------RSQD-----------CPVC
Bra006415-H2           CSVCL----------------------SEFEEDE-----SL-RLLPKCNHAFHLPCIDTWLR--------------------------------------------SHSN-----------CPLC
Bra006470-H2           CPICFEYLF------------------DSLKETT----------VMKCGHTMHCECY-------------------------------------------------HEMIKRDKFC-----CPIC
Bra006583-H2           CAVCL----------------------GDLEDGD-----EV-RELRNCSHVFHRECIDRWLDYEC---------------------------------DDNDGEEDNHRT-----------CPLC
Bra006603-H2           CSICL----------------------EAFCESE-------PSTLTSCKHEYHLQCILEWCQ--------------------------------------------RSSQ-----------CPMC
Bra006641-H2           CPVCFEYLF------------------DSTRDIT----------VLRCGHTMHLQCT-------------------------------------------------KDMGLHNRYT-----CPVC
Bra006709-H2           CAVCT----------------------EVFEGET-----EARE--MPCKHIFHEDCIVPWL--------------------------------------------SIRNS-----------CPVC
Bra006752-H2           CVICL----------------------GLWEVGE-----FG-RKLRTCGHGFHVECIDMWLS--------------------------------------------SHST-----------CPLC
Bra006916-H2           CSICL----------------------MDYEDED------AVTQLPRCNHLFHVHCIEPWLLR-------------------------------------------GSLT-----------CPLC
Bra007174-H2           CSVCL----------------------EQVTVGE------VVRTLP-CLHQFHAVCIDPWLRQ-------------------------------------------QG-T-----------CPVC
Bra007253-H2           CPVCK----------------------EEFELKS-----EAKQ--MPCKHMYHSDCIVPWL--------------------------------------------VQHNT-----------CPVC
Bra007414-H2           CSVCL----------------------EDFEP--K--E-T--VMLTPCKHMFHEECIVPWL--------------------------------------------KSKGQ-----------CPVC
Bra007504-H2           CAVCK----------------------EDFSVGE-----SAKR--LPCSHIYHSDCIVPWL--------------------------------------------SDHNS-----------CPLC
Bra007515-H2           CAVCL----------------------SKFEPED-----QL-RLLPLCCHAFHADCIDTWLV--------------------------------------------SNQT-----------CPLC
Bra007580-H2           CSICL----------------------CAYEDGA------ELRELP-CRHHFHSVCVDKWLR--------------------------------------------INAT-----------CPLC
Bra007606-H2           CAVCL----------------------YEFEGEQ-----EI-RWLRNCRHIFHRSCLDRW-------------------------------------------MDHDQKT-----------CPLC
Bra007613-H2           CSICL----------------------CEYMEEE------MVRMMPECKHYFHVSCLDAWLK--------------------------------------------LNGS-----------CPVC
Bra007629-H2           CSICL----------------------SEYATKE------TVRFLPECEHCFHAECIDAWLK--------------------------------------------LHSS-----------CPVC
Bra007694-H2           CSVCL----------------------SEFEEED-----EG-RVLPKCGHVFHVDCIDTWFR--------------------------------------------SRSS-----------CPLC
Bra007789-H2           CAICL----------------------EGLYK-----N-DETFDLPYCSHRFHSTCVGEWLQS--------------------------------------------HNS-----------CPLC
```

```
Bra007847-H2    CAICL----------------------DDFES-------KTMSMFPGCFHQFHRECLLEWIERSR----------------------------------------RGHGS-----------CPLC
Bra007848-H2    CAICL----------------------EDMSE--S--G-QAFCQPPGCVHVFHEDCFVKWLGR--------------------------------------------HDS-----------CPLC
Bra008015-H2    CPICL----------------------NEFEDEE-----TL-RLIPKCCHVFHPDCIDAWFQ--------------------------------------------SHAT-----------CPLC
Bra008016-H2    CPVCL----------------------NEFEDAE-----RL-RLIPKCCHVFHPGCIDAWLQ--------------------------------------------SHPT-----------CPLC
Bra008018-H2    CPVCL----------------------NEFEEDE-----SL-RLLPKCNHAFHISCIDTWLR--------------------------------------------SHTN-----------CPLC
Bra008028-H2    CAICL----------------------SDLVKGD-----KA-RLLPKCNHSFHVECIDMWFQ--------------------------------------------SHST-----------CPIC
Bra008075-H2    CVVCL----------------------SELAEGD-----KA-KLLPSCKHWFHAHCIDAWLE--------------------------------------------SHAT-----------CPIC
Bra008257-H2    CAICL----------------------TEFAAGD-----EL-RVLPQCGHGFHVSCIDTWLG--------------------------------------------SHSS-----------CPSC
Bra008462-H2    CPVCL----------------------ERLDQDTGG------ILTTMCNHSFHCSCISNWPDS----------------------------------------S-----------------CPVC
Bra008475-H2    CCICL----------------------ARYGDDE------EVRELP-CLHVFHVDCVDKWLK--------------------------------------------INAT-----------CPLC
Bra008518-H2    CRICQ----------------------DEFEGGE-----QV-RSLRNCAHVFHKTCIDRW-------------------------------------------IHDDKMT-----------CPLC
Bra008671-H2    CAICM----------------------GDFVVKE-----KVTR--LPCWHYYHGECIVPWL--------------------------------------------EMKNT-----------CPVC
Bra008675-H2    CPTCL----------------------EEYTSEN-------PKIVTKCSHHFHLGCIYEWME--------------------------------------------RSED-----------CPVC
Bra009015-H2    CSICQ----------------------EEYVDGD------EVGTMP-CEHMYHVSCVQQWLR--------------------------------------------MKNW-----------CPIC
Bra009037-H2    CSVCL----------------------SEFEEGE-----GV-RLLPKCSHAFHVSCIDTWLL--------------------------------------------SHKN-----------CPLC
Bra009136-H2    CAVCL----------------------ARFEPTE-----VL-RLLPKCKHAFHVECVDTWLD--------------------------------------------AHST-----------CPLC
Bra009297-H2    CSICL----------------------CDYVARD------PVRCIPECNHCFHADCVDEWLRT--------------------------------------------SAT-----------CPLC
Bra009323-H2    CAVCK----------------------DEMDVGT-----EAVG--LPCDHKYHTECIVPWL--------------------------------------------KTRNT-----------CPVC
Bra009570-H2    CVICK----------------------EEMSEGR------DVCEMP-CQHLFHWKCILPWL--------------------------------------------SKRNT-----------CPFC
Bra009663-H2    CSVCL----------------------QDFQVGE------TVRSLPHCHHMFHLPCIDKWL--------------------------------------------RGHAS-----------CPLC
Bra009800-H2    CSICQ----------------------EEYVDGD------EVGTLP-CQHMYHVSCAQQWLR--------------------------------------------MKNW-----------CPIC
Bra009912-H2    CPDC------------------------KLPGDDCP------LIWGACNHSFHLHCILKWVNS----------------------------------------QTSQ-AH-----------CPMC
Bra009960-H2    CAICL----------------------NEFQDDE-----TL-RLLPKCDHVFHPHCIGAWLQ--------------------------------------------GHVT-----------CPVC
Bra009962-H2    CAICL----------------------TEFGDDE-----TL-RFLPKCDHVFHAHCIGAWLQ--------------------------------------------GHVT-----------CPVC
Bra010289-H2    CVVCI----------------------EGFRQGQ------WCRKLPGCGHVFHRKCVDFWL--------------------------------------------VKVGT-----------CPIC
Bra010349-H2    CSVCL----------------------SKFESVE-----IL-RLLPKCRHAFHIGCIDQWLE--------------------------------------------QHAT-----------CPLC
Bra010370-H2    CVICM----------------------TAINLRQR----TSDFMVTPCEHFFHTGCLQR--------------------------------------------WMDIKME-----------CPTC
Bra010473-H2    CAICR----------------------EPM-----------KAKRLHCNHLFHLGCLRSWLDQGL----------------------------------------NDVYS-----------CPTC
Bra010532-H2    CAICL----------------------MEFSDGE-----EI-RILPLCRHAFHVACIDKWLT--------------------------------------------SRSS-----------CPSC
Bra010641-H2    CAICL----------------------NELEDRE-----TV-RLLPVCNHLFHVDCIDAWLY--------------------------------------------SHAT-----------CPVC
Bra010702-H2    CGACSRPLS-----EKSLWSSQKMFMTNELSVSA----------ILACGHVYHGECL-------------------------------------------------EKMTPEIDKF--DPSCPIC
Bra010752-H2    CSICL----------------------ESFTPQD-------PATVTSCKHEYHLQCIIEWSQ--------------------------------------------RSKE-----------CPIC
Bra010969-H2    CCVCL----------------------CGFKEEE------EVSELVSCKHFFHRACLDNWFG-------------------------------------------NNHTT-----------CPLC
Bra011068-H2    CSVCL----------------------SKFESVE-----IL-RLLPKCRHAFHVGCIDQWLE--------------------------------------------QHAT-----------CPLC
Bra011187-H2    CVVCI----------------------DGFRQGQ------WCRRLPGCGHVFHRKCVDFWL--------------------------------------------VKVGT-----------CPIC
Bra011189-H2    CAVCL----------------------SEFETED-----KL-RLLPKCSHAFHMDCIDTWLL--------------------------------------------SHST-----------CPLC
Bra011215-H2    CLVCL----------------------DYIEMNT------TGKQMP-CQHKFHSYCLLYWL--------------------------------------------QLHSS-----------CPVC
Bra011359-H2    CCICL----------------------AKYANNE------ELRELP-CSHFFHKECVDKWLK--------------------------------------------INAS-----------CPLC
Bra011618-H2    CAICL----------------------TEFTDGE-----EI-RILPLCNHAFHLACIDKWLT--------------------------------------------SRSS-----------CPSC
Bra011643-H2    CSVCL----------------------QDFQLGE------TVRSLPQCHHMFHLPCIDNWL--------------------------------------------LRHGS-----------CPMC
Bra011873-H2    CGACSRPLS-----EKSMWSSQKIFMTNELSVSA----------ILACGHVYHGECL-------------------------------------------------EQMTPEIDKF--DPSCPIC
Bra011991-H2    CIICL----------------------SKFEEGE-----TV-KVIPHCGHLFHVDCVDTWLN--------------------------------------------SHVT-----------CPLC
Bra012055-H2    CAICL----------------------EDYRFGD------SLRLLP-CQHAFHLSCIDSWLTK-------------------------------------------WGTS-----------CPVC
Bra012134-H2    CVICL----------------------DELKCND------EASTLA-CGHDFHYECIKNWLM--------------------------------------------VKNK-----------CPLC
Bra012251-H2    CAICL----------------------TEFSNGD-----EL-RVLPQCGHGFHVSCIDTWLG--------------------------------------------SHSS-----------CPSC
Bra012330-H2    CPVCL----------------------NGFEDDE-----TL-RLIPQCCHVFHTCCIDAWLR--------------------------------------------SHVT-----------CPLC
Bra012441-H2    CVICL----------------------EEWKAGE------TVKEMP-CKHRFHGGCVEKWL--------------------------------------------GLHGS-----------CPVC
Bra012630-H2    CAICL----------------------LEFDEDH-----VL-RLLTTCYHVFHQECIDLWFE--------------------------------------------SHKT-----------CPVC
Bra012814-H2    CSICL----------------------VDYEAED------AVTHLPRCNHLFHINCIEPWLLS-------------------------------------------GHLT-----------CPLC
Bra013006-H2    CAVCR----------------------EEMSVGN-----EVAE--LPCRHKYHGECIVPWL--------------------------------------------GIRNT-----------CPVC
Bra013110-H2    CTICL----------------------EPLVNTRDD---RRTFVTLRCGHKFHLDCIGSAFNA------------------------------------------KGFMQ-----------CPNC
Bra013118-H2    CCQCQ----------------------AAFDE-SG----GHQLTRLGCLHAIHTSCLVS---------------------------------------LIKSLPPHTAPPG--------YVCPTC
Bra013211-H2    CSVCL----------------------TKFEEGS------EVNKL-KCGHLFHKTCVEKWID-------------------------------------------YWNIT-----------CPLC
Bra013267-H2    CSICL----------------------GEFNEEE-----SL-RLLPNCNHIFHVVCIDRWLT--------------------------------------------SHSN-----------CPLC
Bra013268-H2    CAICL----------------------LEFDGDH-----VL-RLLTTCYHVFHQECIDLWFE--------------------------------------------SHKT-----------CPVC
Bra013751-H2    CCVCL----------------------GEFELKE------ELVEMPSCKHIFHLDCIHLWL--------------------------------------------YSHTT-----------CPLC
Bra013876-H2    CAICR----------------------EPMA----------KAKKLHCNHLFHLGCLRSWLDQGL----------------------------------------NEVYS-----------CPTC
Bra014043-H2    CCVCQ----------------------EEYKEGE------EMGVLE-CGHDFHSQCIKEWLK--------------------------------------------RKNL-----------CPIC
Bra014174-H2    CVICL----------------------SDFVCGE-----KL-RLLPKCNHGFHVRCIDKWLK--------------------------------------------QHLT-----------CPKC
Bra014175-H2    CVICL----------------------SDFVCGE-----KL-RLLPKCNHGFHVRCIDKWLQ--------------------------------------------QHLT-----------CPKC
Bra014177-H2    CVICL----------------------SEFVSEE-----RV-KLLPTCHHGFHVRCIDKWLS--------------------------------------------SHSS-----------CPTC
Bra014221-H2    CSICL----------------------ESFTNGD------MLISLP-CTHSFHSSCLNPWLIA-------------------------------------------CG-D-----------CPYC
Bra014266-H2    CAVCK----------------------EVFSLGN-----ETTQ--LPCLHLYHPHCIVPWL--------------------------------------------GARNS-----------CPLC
Bra014339-H2    CIVCL----------------------STLKSGE------EVRKL-GCRHVFHKQCLEGWLQ-------------------------------------------HLNFK-----------CPLC
Bra014424-H2    CSICL----------------------SEYASSE------TVGCLLICEHCFHVECIDTWLQ--------------------------------------------LRSS-----------CPIC
Bra014435-H2    CAVCL----------------------YEFEGDQ-----EI-RRLRNCRHIFHRSCLDRW-------------------------------------------MDHDQKT-----------CPLC
Bra014499-H2    CPLCLYPLFP-----------------EDGES--D--Q-MPFMKLMSCFHCFHCECIIRWWNWLHTEKEADSVN------------------------GNSGSVDKSLGN-----------CPVC
Bra014508-H2    CAVCL----------------------QEAEEGD-----KMRR-LTICRHCFHADCIDPWLG-------------------------------------------EMSST-----------CPLC
Bra014684-H2    CPVCK----------------------EEFELKS-----EAKQ--MPCKHVYHSDCIVPWL--------------------------------------------VQHNT-----------CPVC
Bra015142-H2    CSVCL----------------------SNFEEDS------EINKL-KCGHLFHKTCLEKWID-------------------------------------------YWNIT-----------CPLC
Bra015160-H2    CTACT----------------------FTLDIP------AVHFM---CMHSFHQRCLG-----------------------------------------------DNETE-----------CPEC
Bra015221-H2    CCICQ----------------------EEYAEGD------DLGTLE-CGHEFHKDCIKQWVM--------------------------------------------LKNL-----------CPIC
Bra015225-H2    CAICL----------------------DQFKTGE------TLVHLP-CTHKFHSICLLPWLDT-------------------------------------------NA-Y-----------CPYC
Bra015233-H2    CSICI----------------------DDLSKT-----RENIIELPQCLHVFHQDCLFEWLR--------------------------------------------RQNS-----------CPLC
Bra015308-H2    CSVCL----------------------NEFQEDE-----KL-RIIPNCCHVFHIDCIDIWLQ--------------------------------------------SNAN-----------CPLC
Bra015349-H2    CVICL----------------------ETPTIGD------TIRHLP-CFHKFHKDCIDPWLGR-------------------------------------------SK-A-----------CPVC
Bra015751-H2    CAICL----------------------TEFAAGD-----EL-RVLPQCGHGFHVACIDTWLG--------------------------------------------SHSS-----------CPSC
Bra015846-H2    CGACSKLLT-----ERSSVAN------FELPIAA----------VLACGHVYHAECL-------------------------------------------------ETMTTEIEKY--DPACPIC
Bra015894-H2    CPICHEYIF------------------TSSSPVK----------ALPCGHLMHSKCF-------------------------------------------------KDYT-CSHYT-----CPVC
Bra015913-H2    CTICL----------------------QDIAVGE------ITRGLPRCDHTFHLVCVDKWL--------------------------------------------IRHGS-----------CPIC
Bra015967-H2    CSICQ----------------------EDYEAKD------EVGKLR-CGHRYHISCVKQWLL--------------------------------------------RKNC-----------CPVC
Bra016067-H2    CAICL----------------------SALVKGD-----KA-RLLPKCSHSFHVECIDMWFQ--------------------------------------------SHST-----------CPIC
Bra016079-H2    CPVCL----------------------NEFQEDE-----SL-RLLPKCNHAFHISCIDTWLR--------------------------------------------SHTN-----------CPLC
Bra016093-H2    CAICL----------------------EDYSVGD------KLRLLP-CRHKFHAVCVDSWLTS-------------------------------------------WRTF-----------CPVC
Bra016449-H2    CAICL----------------------TEFTAGD-----EL-RVLPQCGHGFHLSCIDTWLG--------------------------------------------SHSS-----------CPSC
Bra016574-H2    CSICQ----------------------DEYEREC------QVGKLE-CGHSFHVQCVKQWLS--------------------------------------------RKNA-----------CPVC
Bra016759-H2    CCICL----------------------SAYEDGT------ELRELP-CGHHFHCSCVDKWLY--------------------------------------------INAT-----------CPLC
Bra016770-H2    CGVCKRKI---LTMTGDFRMAQSYFSAGPLAPFY----------VFPCGHSFHAQCLITHVTSCAHEEQAEHILDLQKQLTLLGSEPPRNINGNRSDEPITSTTTADKLRSELDDA-IASECPFC
Bra016880-H2    CAVCL----------------------SVFEEQD-----TA-RELPNCRHVFHVDCIDTWLT--------------------------------------------TCST-----------CPVC
Bra016881-H2    CAVCL----------------------SLLEEKD-----TA-RMLPNCKHVFHVTCIDTWLT--------------------------------------------TCST-----------CPVC
Bra017177-H2    CTVCK----------------------SVLADGE------EIRQLSACKHEFHVSCIEEWLQ-------------------------------------------TRS-N-----------CPNC
Bra017205-H2    CAICL----------------------AEYKEGE------ELGELKGCGHDYHGGCIKKWLS--------------------------------------------LKNS-----------CPIC
Bra017280-H2    CSICL----------------------GDYKGNH------LLRQLPDCNHLFHLKCIDTWLRL--------------------------------------------NPT-----------CPVC
Bra017320-H2    CAICL----------------------AEFSDED-----TV-RLITVCRHGFHSTCIDSWFE--------------------------------------------SHKT-----------CPVC
Bra017465-H2    CIVCL----------------------STLKTGE------QVRKL-DCRHVFHKQCLEGWIQ-------------------------------------------HLNFN-----------CPLC
Bra017535-H2    CAICL----------------------LEFEEEH-----VLLRLLTTCYHVFHQECIDRWLE--------------------------------------------SNKT-----------CPVC
Bra017713-H2    CAICL----------------------TEFSDGE-----EI-RILPLCSHAFHVACIDKWLT--------------------------------------------SRSS-----------CPSC
Bra017732-H2    CSVCL----------------------QDFQLGE------TVRSLPHCHHMFHLPCIDNWL--------------------------------------------LRHGS-----------CPMC
Bra017859-H2    CGICL----------------------QSAKAGR-----GTAIFTAECSHTFHFPCVASRAG-----------------------------------------DLTLLAA-----------CPVC
Bra018091-H2    CLICL----------------------EEFQIGH------EVRGLP-CAHNFHVECIDQWLR--------------------------------------------LNVK-----------CPRC
```

```
Bra018644-H2    CCMCF----------------------DPVS--IR----GDTVVVFFCCHAYHMTCLMDAAFSDSNIKTAAKGSSGYGYGYDNGVEEE----------DTEDEEEDDSNDGDRSGR-SRLRCILC
Bra018663-H2    CAICL----------------------DEIVKGDGK-----AIFTAECSHTFHFDCITTNVK------------------------------------------HGN-KI-----------CPLC
Bra018777-H2    CVICL----------------------SDFVSGE-----KL-RLLPNCNHGFHVRCIDKWLQ--------------------------------------------QHLT-----------CPSC
Bra018779-H2    CVICL----------------------SDFVSGE-----KL-RLLPKCNHGFHVHCIDKWLQ--------------------------------------------QHLT-----------CPSC
Bra018780-H2    CVICL----------------------SDFVSGE-----KL-RLLPKCNHGFHVCCIDKWLQ--------------------------------------------QHLT-----------CPKC
Bra018781-H2    CAICL----------------------SEFVSEE-----RV-KLLPTCHHGFHVRCIDKWLS--------------------------------------------SHSS-----------CPTC
Bra019006-H2    CAICL----------------------SGYVVNE-----ECRVF-PVCRHMYHAVCIDAWL--------------------------------------------KNHLT-----------CPTC
Bra019109-H2    CSVCL----------------------DEFDKGC------EAKEMP-CKHKFHIRCIVPWL--------------------------------------------ELHSS-----------CPVC
Bra019181-H2    CAICR----------------------EPMA----------KAKRLHCNHLFHLGCLRSWLDQGL----------------------------------------NEVYS-----------CPTC
Bra019252-H2    CCVCL----------------------GEFELKE------ELVEMPSCKHIFHLDCIHLWL--------------------------------------------YSHTT-----------CPLC
Bra019304-H2    CPICL----------------------EEYETEN-------PRLLTKCRHDFHLACILEWME--------------------------------------------RSES-----------CPVC
Bra019382-H2    CAVCT----------------------EVFEAGA-----EGRE--MPCKHIFHGDCIVPWL--------------------------------------------SIRNS-----------CPVC
Bra019499-H2    CVVCQ----------------------MEFEEGE------SLVVLRLCNHPYHSECITKWLQ--------------------------------------------TKKV-----------CPIC
Bra019672-H2    CPICL----------------------DEWSEGD------VAAEMP-CKHRFHSKCVEEWL--------------------------------------------GRQAT-----------CPLC
Bra019673-H2    CGLCNRYIS-----QKPPCGSRSIVRNQDMPVTG----------VLPCQHVFHAECL-------------------------------------------------DQSTPKAHRN--DPPCPVC
Bra019769-H2    CGVCKRKI---LTMTGDFRMAQGYSSSGPLAPFY----------VFPCGHSFHAQCLITHVTSCAHEEQAEHILDLQKQLTLLGSETRRDMNGNRSDEPITSTTTADKLRSELDDA-IASECPFC
Bra019784-H2    CCICL----------------------CAYEDGS------ELRELP-CGHHFHCSCVDKWLY--------------------------------------------INAT-----------CPLC
Bra019945-H2    CSICL----------------------EDLSCG---GGPGGVPSRMPCSHVFHIRCLLKWFS--------------------------------------------RKST-----------CPMC
Bra020133-H2    CAVCL----------------------GDLEDGD-----EV-RELRNCSHVFHRECIDRWLDYECCGG------------------------------DGNEGEEDNHRT-----------CPLC
Bra020167-H2    -SICL----------------------EVFCDSD-------PSTLTSCKHEYHLQCILEWCQ--------------------------------------------RSSQ-----------CPMC
Bra020183-H2    CVICT----------------------EEMFLA------EEATSMPCCSHVFHSSCIEKWFQ--------------------------------------------VGNK-----------CPLC
Bra020234-H2    CAICL----------------------TAMKAGQGH-----AIFTAECSHSFHFHCITTNVK------------------------------------------HGN-QI-----------CPVC
Bra020313-H2    CAVCT----------------------EVFEAET-----EARE--MPCKHIFHEDCIVPWL--------------------------------------------SIRNS-----------CPVC
Bra020410-H2    CDLCDRDLA-----SDPERPNASL---RSLQEAC----------VLACGHVYHFKCL-------------------------------------------------RGTTLDLDN----PSCILC
Bra020507-H2    CPICFEYLF------------------ESRNDVT----------VLPCGHTIHQKCL-------------------------------------------------EEMREHYQYA-----CPLC
Bra020554-H2    CPDC------------------------KLPGDDCP------LIWGACNHSFHLHCILKWVNS----------------------------------------QTSQ-AH-----------CPMC
Bra020591-H2    CAICL----------------------NEFEDDE-----TL-RLLPKCDHVFHPHCIDAWLK--------------------------------------------SHVT-----------CPVC
Bra021061-H2    CIICL----------------------SEFKDGD-----TL-RVLDRCKHGFHVHCIQQWFS--------------------------------------------SNRS-----------CPTC
Bra021160-H2    CIICR----------------------EEMT----------TAKKLICGHLFHVHCLRSWLER--------------------------------------------QQT-----------CPTC
Bra021193-H2    CAVCL----------------------SEFEEGE-----SG-RVLPGCKHAFHVECIDMWFH--------------------------------------------SHST-----------CPLC
Bra021195-H2    CAVCL----------------------SEFEEGE-----SG-RVLPGCKHAFHVECIDMWFH--------------------------------------------SHST-----------CPLC
Bra021196-H2    CPVCL----------------------SEFEEGE-----SG-RVLPGCKHAFHVECIDMWFH--------------------------------------------SHST-----------CPLC
Bra021280-H2    CPICL----------------------EHLNGG------LGYIRLRNCMHKFHSRCIDKWLF--------------------------------------------KCAR-----------CPLC
Bra021354-H2    CSVCL----------------------CEFQENE-----RL-RLLPECNHAFHVACIDTWLK--------------------------------------------SHSN-----------CPLC
Bra021404-H2    CSVCL----------------------SEFEEED-----QG-RILPKCGHAFHVDCIDTWFR--------------------------------------------SRST-----------CPLC
Bra021467-H2    CAVCK----------------------DEMVLEE-----EVKR--LPCRHLYHGECITPWL--------------------------------------------GIRNT-----------CPVC
Bra021618-H2    CTVCL----------------------FDFAYDD-----KV-RQLPNCRHVFHKRCMDRWI------------------------------------------VDCRKIT-----------CPIC
Bra021697-H2    CAICH----------------------NELGASG-------ELNTLVCNHSYHHECIIGWVK--------------------------------------------MNLT-----------CPVC
Bra022125-H2    CGICL----------------------EELNPRG-----EIYFDMPNCSHQFHDICISRWLR--------------------------------------------RSKT-----------CPLC
Bra022144-H2    CAICL----------------------SEFEQGE-----SI-QVLEKCHHGFHVKCIHKWLS--------------------------------------------SRSS-----------CPTC
Bra022174-H2    CAVCL----------------------SEFEEGE-----SG-RVLPGCKHTFHVDCIDMWFH--------------------------------------------SHST-----------CPLC
Bra022310-H2    CPICCEFLF------------------TSSEAVR----------ALPCGHYMHSACF-------------------------------------------------QAYT-CSHYT-----CPIC
Bra022354-H2    CVICL----------------------SDFVAGE-----RI-RLLPKCNHGFHVRCIDKWLT--------------------------------------------QHIT-----------CPKC
Bra022355-H2    CAICL----------------------SEFVSGE-----RV-KLLPKCHHGFHVRCIDKWLS--------------------------------------------SHSS-----------CPTC
Bra022370-H2    CAVCL----------------------LEFEEGD-----YV-RTLPLCFHAFHLECIDEWLR--------------------------------------------SHPN-----------CPLC
Bra022466-H2    CVICR----------------------LDYEDDD------DLILLP-CKHSYHSECINNWLK--------------------------------------------INKS-----------CPVC
Bra022498-H2    CIICR----------------------EEMT----------SAKKLVCGHLFHVHCLRSWLER--------------------------------------------QNT-----------CPTC
Bra022630-H2    CSICL----------------------SEYEPKE------ILKTIPPCQHCFHADCIDEWLK--------------------------------------------LNGT-----------CPVC
Bra022849-H2    CPICC----------------------KEFGTEG-------DINSLNCEHSYHHHCILDWVK--------------------------------------------KSLT-----------CPYC
Bra023041-H2    CPICL----------------------GDYKGNH------LLRQLPDCKHLFHLKCIDTWLRL--------------------------------------------NPT-----------CPVC
Bra023042-H2    CHICL----------------------GDFKGNH------MLRQLPDCNHLFHLKCVDTWLRQ--------------------------------------------NPT-----------CPVC
Bra023101-H2    CAICL----------------------EEYVEGE------ELGELKGCGHDYHGGCIKKWLS--------------------------------------------MKNS-----------CPIC
Bra023261-H2    CSICL----------------------LEFMDDD-----TM-RLISTCNHVFHTICIDLWFE--------------------------------------------SHKT-----------CPVC
Bra023539-H2    CAICK----------------------DEIVVEE-----KVKR--LPCRHYYHKECIVPWL--------------------------------------------GIRNT-----------CPVC
Bra023814-H2    CSACL----------------------NEFHVNE-----TV-RVIPHCFHLFHVDCVD---P--------------------------------------------KNVS-----------CPLC
Bra023832-H2    CIVCF----------------------GNYSQHN------NLC-ILTCGHSFHFACIDQWI--------------------------------------------RRDIS-----------CPIC
Bra024007-H2    CSICQ----------------------EEYTIGD------EVGRLH-CEHAYHLKCVQEWLR--------------------------------------------MKSW-----------CPIC
Bra024009-H2    CSICQ----------------------EEYTIGD------EVGRLH-CEHTYHLKCVQEWLR--------------------------------------------MKSW-----------CPIC
Bra024081-H2    CAVCL----------------------CEFETED-----KL-RLLPKCSHAFHVDCIDTWLL--------------------------------------------SHST-----------CPLC
Bra024178-H2    CSVCL----------------------SKFESVE-----IL-RLLPKCRHAFHIGCIDQWLE--------------------------------------------EHAT-----------CPLC
Bra024408-H2    CGICL----------------------QRLNSGQIN---STAIFTAECSHSFHLSCAVKLDG----------------------------------------------KR-----------CPFC
Bra024491-H2    CSICL----------------------QDYQRGE------IVRILPSCRHMFHLPCIDSWF--------------------------------------------LEHRS-----------CPLC
Bra024512-H2    CAICL----------------------SDFADGE-----EI-RVLPLCGHSFHVECIDKWLV--------------------------------------------SRSS-----------CPSC
Bra024529-H2    CAICL----------------------EDYSVGE------KLRVLP-CCHKFHATCVDVWLTS-------------------------------------------WRTF-----------CPVC
Bra024628-H2    CAVCL----------------------CEFSEDD-----KL-RLLPICSHAFHIDCIDTWLL--------------------------------------------SNST-----------CPLC
Bra024853-H2    CCICL----------------------SSYEDGA------ELHTLP-CNHHFHFNCIVKWLK--------------------------------------------MKAT-----------CPLC
Bra024902-H2    CIVCL----------------------STLKTGE------EVRKL-DCRHVFHKHCLEGWLQ-------------------------------------------HLNFN-----------CPLC
Bra024913-H2    CAICL----------------------EPLSHCDDNSP-SEATFTGQCSHSFHFSCIASNVR------------------------------------------HGS-VT-----------CPVC
Bra024929-H2    CAICL----------------------SEFEPGE-----SI-HVLEKCHHGFHVKCIHKWLS--------------------------------------------SHSS-----------CPTC
Bra025008-H2    CAICL----------------------LEFEEEH-----ILLRLLTTCYHVFHQECIDRWLD--------------------------------------------SNKT-----------CPVC
Bra025085-H2    CYICL----------------------VEYEEGD------TIRTLP-CHHEFHRTCVDKWLKE-------------------------------------------IHRV-----------CPLC
Bra025417-H2    CAICR----------------------EELTANE-----RLSE--LPCSHCYHKDCISNWL--------------------------------------------SNRNT-----------CPLC
Bra025504-H2    CPICL----------------------EEYDMDN-------PKLLAKCEHHFHLACILEWME--------------------------------------------RSET-----------CPVC
Bra025597-H2    CAVCL----------------------CEFTEKD-----KL-RLLPTCSHAFHLNCIDTWLQ--------------------------------------------SNST-----------CPLC
Bra025698-H2    CSICHEYIF------------------NSNSPVK----------ALPCGHVMHSTCF-------------------------------------------------QEYT-CFHYT-----CPIC
Bra025846-H2    CAICL----------------------TEFSAGD-----EL-RVMPQCGHGFHLSCIDTWLG--------------------------------------------SHSS-----------CPSC
Bra026165-H2    CVVCL----------------------SELHEGE------EVRKL-ECGHVFHKPCLEGWLH-------------------------------------------HLHFT-----------CPLC
Bra026337-H2    CVVCL----------------------FELADGD-----KA-KFLPSCKHWFHARCIDPWLE--------------------------------------------SHAT-----------CPIC
Bra026420-H2    CCICL----------------------AKYKDKE------EVRKLP-CSHRFHVKCVDQWLR--------------------------------------------IISS-----------CPLC
Bra026444-H2    CSVCL----------------------DEFEKGS------EAKEMP-CKHKFHVGCIVPWL--------------------------------------------ELHSS-----------CPVC
Bra026849-H2    CSICL----------------------EEWSEGD------VAAEMP-CKHEFHSKCVEEWL--------------------------------------------GRHAT-----------CPLC
Bra026851-H2    CGLCNRYIS-----QKSPWGSRSIMRNRDMPVTG----------VLPCQHVFHAECL-------------------------------------------------DQSTPKTHGN--DPPCPVC
Bra026955-H2    CCICL----------------------SAYEDGT------ELRELP-CGHHFHCSCVDKWLY--------------------------------------------INAT-----------CPLC
Bra027034-H2    CAICH----------------------NELGASG-------DLNTLVCNHSYHHQCILGWIK--------------------------------------------MNLT-----------CPVC
Bra027282-H2    CTICQ----------------------ENYKNQD------KIATLD-CRHEYHAECLKKWLV--------------------------------------------IKNI-----------CPVC
Bra027349-H2    CVVCL----------------------SNLVDGD-----KA-RVMPTCKHCFHVDCIDKWLK--------------------------------------------CNSI-----------CPVC
Bra027436-H2    CTVCL----------------------DDFEIGV------EAKEMP-CKHKFHSECLLPWL--------------------------------------------ELHSS-----------CPVC
Bra027467-H2    CCICQ----------------------EEYSEGE------DMGALE-CGHDFHSQCIKEWLK--------------------------------------------QKNL-----------CPIC
Bra027562-H2    CTICQ----------------------EPYRAGD------AVRTLP-CDHSFHSICVDQWLIS-------------------------------------------DHGD-----------CPLC
Bra027625-H2    CCICL----------------------SAYADET------ELRELP-CGHHFHCSCVDKWLY--------------------------------------------INAT-----------CPLC
Bra027664-H2    CAVCL----------------------YDFENDD-----EI-RRLTNCRHIFHKECLDRWI------------------------------------------MDYSQMT-----------CPLC
Bra027782-H2    CAVCL----------------------YDFENHD-----EI-RRLTNCRHIFHKGCLDRWM------------------------------------------MDYNQIT-----------CPLC
Bra028004-H2    CSVCL----------------------GDYQADD------KLQQIPACGHTFHMDCIDLWL--------------------------------------------TSHTS-----------CPLC
Bra028075-H2    CAICL----------------------NEFDDGE-----EL-RLMPACSHAFHAPCIDVWLS--------------------------------------------SRST-----------CPVC
Bra028093-H2    CPVCL----------------------CEFEADD-----KL-RLLPKCSHAFHVDCIDTWLL--------------------------------------------SHST-----------CPLC
Bra028138-H2    CSICI----------------------EDLSKS-----QQSIIEMPNCLHMFHQNCLFEWLG--------------------------------------------RKNS-----------CPLC
Bra028140-H2    CSICI----------------------EDFSVS-----HENIIWMPQCKHVFHQGCLFEWLS--------------------------------------------RQNS-----------CPLC
Bra028173-H2    CTVCM----------------------EDFVPGG-----EATE--LPCKHIYHKNCITPWL--------------------------------------------RLHNS-----------CPIC
Bra028213-H2    CPTCL----------------------DDYTPEN-------PKIITKCSHHFHLSCIYEWME--------------------------------------------RSET-----------CPVC
Bra028453-H2    CAVCL----------------------CEFTERD-----KL-RLLPMCSHAFHLTCIDTWLQ--------------------------------------------SNST-----------CPLC
```

```
Bra028464-H2     CAICL----------------------EDLLVGS------AASTLP-CQHHFHTGCILEWLKN------------------------------------------SR-F-----------CPTC
Bra028731-H2     CSICL----------------------ADYKKTD------MIRVLPDCNHLFHDKCVDPWLKL-------------------------------------------HPT-----------CPVC
Bra028866-H2     CVICK----------------------EEMGEGR------DVCEMP-CQHLFHWKCILPWL-------------------------------------------SKRNT-----------CPFC
Bra028915-H2     CAICL----------------------GEFADGE-----KV-RVLPPCNHSFHMSCIDTWLV-------------------------------------------SHSS-----------CPNC
Bra028935-H2     CSVCL----------------------DEFERGV------EAKEMP-CKHKFHVKCIVPWL-------------------------------------------EIHSS-----------CPVC
Bra028950-H2     CCICL----------------------AKYKDKE------EVRKLP-CSHKFHLKCVDQWLR-------------------------------------------IISC-----------CPLC
Bra029441-H2     CSICL----------------------DNLVS-----DKHGVSTRMTCSHVFHEKCLLVWFQ-------------------------------------------RKNT-----------CPLC
Bra029442-H2     CSICL----------------------DNLVVSGRSNSKRGIPTRMTCSHVFHDGCLLEWLQ-------------------------------------------RKNT-----------CPLC
Bra029869-H2     CPVCQ----------------------DRFEMGS-----SARK--MPCRHIYHSECIVPWL-------------------------------------------FQHNS-----------CPVC
Bra030136-H2     CAVCK----------------------DAMVMSE-----IGKK--LPCGHFYHDNCILPWL-------------------------------------------ETRNS-----------CPVC
Bra030218-H2     CGICS----------------------QSVKPGQ-----GTAIFTAECSHTFHFPCVTSRAAA---------------------------------------DHNRLVT-----------CPVC
Bra030408-H2     CSICL----------------------EEYEDDH-----EIMRLNK-CGHIFHHFCMDSWLA-------------------------------------------RHRS-----------CPNC
Bra030712-H2     CAICL----------------------DEIRKEDGK-----AIFTAECSHSFHFDCITSNVK-----------------------------------------HGN-RI-----------CPLC
Bra030854-H2     CSVCL----------------------DEFEIGV------EAKEMP-CEHKFHGECLLPWL-------------------------------------------ELHSS-----------CPVC
Bra030982-H2     CPICW----------------------EEFGTEL-------DINSLSCNHTYHHHCISNWVE-------------------------------------------KTLT-----------CPYC
Bra030987-H2     CSICL----------------------SPFKPED-----RL-RHLPLCSHAFHLDCISTWLI-------------------------------------------SNKT-----------CPLC
Bra031056-H2     CPICHEYIF------------------TSNSPAK----------ALPCGHVMHSACF------------------------------------------------QEYT-CSHYT-----CPIC
Bra031193-H2     CGACSRPLS-----QKSLLSSQKIFATNELSVAA----------ILACGHVYHSECL------------------------------------------------EQMTPEADKF--DPQCPVC
Bra031441-H2     CSACM----------------------NEFHVNE-----TV-RVIPHCFHLFHIDCVD---P-------------------------------------------KNVN-----------CPLC
Bra031849-H2     CGICL----------------------QRVHSDQIN---STAIFTAECSHSFHLSCAVRLQD---------------------------------------------KR-----------CPFC
Bra032086-H2     CAICL----------------------EDMSEEES--E-QVLCQPPECLHMFHEDCLTNWLDR-------------------------------------------HDS-----------CPLC
Bra032514-H2     CSVCL----------------------NEFQEDE-----KL-RIIPNCCHVFHIDCIDIWLQ-------------------------------------------GNAN-----------CPLC
Bra033193-H2     CSVCL----------------------GDYQANE------KLQQIPPCGHTFHMDCIDLWL-------------------------------------------TLHTT-----------CPLC
Bra033599-H2     CAACARPLS-----EKSLWSRQNIFMTNELSVSA----------VLACGHVYHGDCL------------------------------------------------EQMTPEVDKF--DPSCPIC
Bra033652-H2     CCICQ----------------------EEYTEGD------DMGTLE-CGHEFHSQCIKEWLK-------------------------------------------QKNL-----------CPIC
Bra033680-H2     CSVCL----------------------NEFQEEE-----KL-RIIPHCSHLFHIDCIDIWLQ-------------------------------------------NNAN-----------CPLC
Bra033777-H2     CLICL----------------------EEFHIGH------EVRGLP-CAHNFHVECIDQWLR-------------------------------------------LNVK-----------CPRC
Bra033953-H2     CAICK------------------*---EEFEVGE-----EGKE--LKCLHLYHPSCIVSWL-------------------------------------------NIHNT-----------CPIC
Bra033963-H2     CCICL----------------------SSYEDGI------ELVTLP-CNHHFHSTCIVKWLK-------------------------------------------MNAT-----------CPLC
Bra034152-H2     CLICL----------------------GDFVEGE-----KV-RVLPKCNHGFHVRCIDTWLL-------------------------------------------SRDS-----------CPTC
Bra034259-H2     CAICL----------------------EDLWT-----N--KQLKLPHCSHQFHFRCISKWLQS-------------------------------------------RNS-----------CPLC
Bra034573-H2     CSVCL----------------------SEFEEEE-----TL-RLLPKCRHAFHVPCIDTWLR-------------------------------------------SHTN-----------CPVC
Bra034865-H2     CCICL----------------------GGLEEGE-----KMKV-LPLCRHCYHCECVDRWL-------------------------------------------MTESS-----------CPLC
Bra035010-H2     CPVCL----------------------ENIESCQ------SARLVPGCNHGFHQLCADTWL-------------------------------------------SNHTV-----------CPVC
Bra035230-H2     CTVCL----------------------SDFESDD-----DI-RQLPNCRHVFHHRCLDRWI-----------------------------------------VDCCKVT-----------CPVC
Bra035284-H2     CSICL----------------------DNLYGS---SSIHGSATLMNCSHVFHERCLSDWLQ-------------------------------------------QKNT-----------CPMC
Bra035483-H2     CSICL----------------------IDFMDDD-----TM-RLISTCNHFFHTICIDLWFE-------------------------------------------SHKT-----------CPVC
Bra035660-H2     CVICL----------------------EDFEVND-----VV-RILVGCKHVFHVECIDSWC-------------------------------------------FYKLT-----------CPVC
Bra035785-H2     CSICL----------------------QDWEEGQ------VGRKLERCGHTFHMKCIDEWF-------------------------------------------LRQVS-----------CPIC
Bra035835-H2     CAVCM----------------------DEFEDGG------DVKQVP-CKHVFHQDCLMPWL-------------------------------------------ELHNS-----------CPVC
Bra036197-H2     CAICR----------------------EKLAPSE-----RLSE--LPCRHYYHKDCISSWL-------------------------------------------TNRNT-----------CPLC
Bra036491-H2     CVICM----------------------ATIDLRRR----TNDCMVTPCEHLFHSGCLQR-------------------------------------------WMDIKME-----------CPTC
Bra036631-H2     CAICL----------------------NEFEDDE-----TL-CLLPKCDHVFHPHCIGAWLE-------------------------------------------GHVT-----------CPVC
Bra036816-H2     CSVCL----------------------MRMEDED------AIKSLP-CSHEFHSLCVDTWFDV-----------------------------------------SRKIC-----------CPLC
Bra037002-H2     CSVCL----------------------SEFEEEE-----ML-RLLPKCRHAFHLSCIDTWLR-------------------------------------------SHTN-----------CPLC
Bra037109-H2     CSVCL----------------------DDFEVGV------EAKQMP-CKHNFHADCLLPWL-------------------------------------------ELHSS-----------CPVC
Bra037333-H2     CPICF----------------------EDYEADN-------PKIKTNCEHDFHLSCILEWME-------------------------------------------KSDG-----------CPIC
Bra037367-H2     CSICY----------------------EDLKPA-----AENLQSISACGHVFHELCLQQWFEYCPS--------------------------------------TNKRN-----------CPIC
Bra037410-H2     CCICN----------------------CPLTKTFS----TLRVRVFNCGHATHLQCEVS------------------------------------------ESETSSSG-----------CPVC
Bra037561-H2     CAICL----------------------SEFVSGE-----RV-KLLPKCHHGFHVRCIDKWLS-------------------------------------------SHSS-----------CPTC
Bra037562-H2     CVICL----------------------SDFVAGE-----QV-RLLPKCNHGFHVRCIDKWLK-------------------------------------------QHVT-----------CPKC
Bra037592-H2     CPICCEFLF------------------TSSETVR----------ALPCGHYMHSACF------------------------------------------------QAYT-CSHYT-----CPIC
Bra037644-H2     CSICL----------------------QDAAEGE-----KMRR-LTACGHCFHAECIDPWL-------------------------------------------EKRST-----------CPQC
Bra037800-H2     CAVCK----------------------DGMVIGE-----TVKK--LPCGHCYHGNCILPWL-------------------------------------------GTRNS-----------CPVC
Bra037999-H2     CCVCL----------------------DDFEMGT------VGKQMP-CKHIFHSDCLLPWL-------------------------------------------QLHSS-----------CPVC
Bra038051-H2     CAVCL----------------------SEFEESE-----PG-RVLPNCKHAFHVECIDMWFL-------------------------------------------SHSS-----------CPLC
Bra038109-H2     CTICQ----------------------ESFKNEE------KIATLD-CGHEYHAGCLEKWLV-------------------------------------------VKNV-----------CPIC
Bra038199-H2     CAVCL----------------------GDLEDGD-----EV-RELRNCSHVFHRECIDRWLDYEC--------------------------------DDNDGEEDNHRT-----------CPLC
Bra038240-H2     CVICR----------------------LDYEDDD------DLILLP-CKHSYHSECINNWLK-------------------------------------------INKI-----------CPVC
Bra038246-H2     CAVCM----------------------DEFEDGG------DVKQMP-CKHVFHQDCLMPWL-------------------------------------------ELHNS-----------CPVC
Bra038386-H2     CAVCL----------------------NEYKEDE-----ML-RLILPCGHVFHVHCVDIWFS-------------------------------------------YRST-----------CPIC
Bra038545-H2     CPVCL----------------------CEFEAED-----KL-RLLPKCSHAFHVDCIDTWLL-------------------------------------------SHST-----------CPLC
Bra038813-H2     CVVCL----------------------DGFRQGQ------WCRNLPGCGHVFHRKCVDTWL-------------------------------------------LKAAS-----------CPIC
Bra039168-H2     CPTCL----------------------EEYTSEN-------PKIVTKCSHHFHLGCIYEWME-------------------------------------------RSEN-----------CPVC
Bra039281-H2     CPICL----------------------SEYASKE------TVRCIPECEHCFHIECIDAWLK-------------------------------------------LHGS-----------CPLC
Bra039282-H2     CPICL----------------------SEYASKE------TIRFMPECDHCFHVECIDVWLK-------------------------------------------IHGS-----------CPLC
Bra039287-H2     CTICL----------------------CEYKEAE------MLRMMPECKHYFHLCCLDAWLK-------------------------------------------LNGS-----------CPVC
Bra039317-H2     CCVCL----------------------CEYEDGT------ELRELW-CRHHFHEACIDKWLR-------------------------------------------INAT-----------CPLC
Bra039318-H2     CCVCL----------------------CEYEDGT------ELRELW-CRHHFHEACIDKWLR-------------------------------------------INAT-----------CPLC
Bra039430-H2     CSICL----------------------DTVAKDGD-----RAWANLQCGHLFHLDCIGSAFNA-----------------------------------------KGVMQ-----------CPNC
Bra039454-H2     CAICL----------------------NEFEDDE-----TL-RLVPKCDHVFHPHCIGAWLE-------------------------------------------AHVT-----------CPVC
Bra039652-H2     CSICL----------------------ESFTNGD------MLISLP-CTHSFHSSCLNPWLKA------------------------------------------CG-D-----------CPYC
Bra039659-H2     CTICQ----------------------ESFKNEE------KIATLD-CGHEYHAECLEKWLI-------------------------------------------VKNV-----------CPIC
Bra039822-H2     CCQCQ----------------------STFDE-GG----GLQVTRLGCLHAIHTSCLVS--------------------------------------LIKSFPPHTAPAG--------YVCPSC
Bra039842-H2     CCICQ----------------------EEYAEGD------DLGTLE-CGHEFHKDCIKQWVM-------------------------------------------LKNL-----------CPIC
Bra040087-H2     CCICL----------------------AKYANNE------ELRELP-CSHFFHKECVDKWLK-------------------------------------------INAS-----------CPLC
Bra040450-H2     CVICQ----------------------LKYKIGE------KQMNLP-CKHVYHSECISKWLS-------------------------------------------INKI-----------CPVC
Bra040498-H2     CGICL----------------------NSVKTGQ-----GTAKYTAECSHVFHFPCVTDYVS----------------------------------------KHGKLV------------CPVC
Bra040540-H2     CSICI----------------------ETVTKEGD-----RAWAKLHCGHEFHLDCIGSAFNT-----------------------------------------KGVMQ-----------CPNC
Bra040564-H2     CAVCL----------------------SHLVDGD-----KA-RVLPGCNHGFHVDCIDMWFQ-------------------------------------------SHST-----------CPLC
Bra040642-H2     CAICL----------------------NEFEDDE-----TL-RLLPKCDHVFHPHCIDAWLE-------------------------------------------AHVT-----------CPVC
Bra040855-H2     CPVCL----------------------NEFEDDE-----TL-RLLPQCCHVFHPGCIDAWLR-------------------------------------------SHAT-----------CPLC
Bra040966-H2     CCICL----------------------CEYEDGV------ELRELP-CNHHFHCTCIDKWLH-------------------------------------------INSR-----------CPLC
Bra008196-H2     SSTCSNDLATGSHYASSEGDWSLNTFSELGGVFSKGQMMSSVAAVLACGHVYHAECLETVTTE----------------------------------------IEKYDPA----------CPIC
Bra002312-H2     CAIC--------------RNHIMDLCIECQANQASATSEECTVAWGVCNHAFHFHCISRWL--------------------------------------------KTRQV----------CPLD
Bra006565-H2     CAIC--------------RNHIMDLCIECQANQASATSEECTVAWGVCNHAFHFHCISRWL--------------------------------------------KTRQV----------CPLD
Bra020111-H2     CAIC--------------RNHIMDLCIECQANQASATSEECTVAWGVCNHAFHFHCISRWL--------------------------------------------KTRQV----------CPLD
Bra006585-H2     CCIR------------------------KENLVIG--DKMQE--LP-CKHTFHPPCLKPWL--------------------------------------------DEHNS----------CPIC
Bra016327-H2     CAVC------------------------LCEFSED--DKLRL--LPVCSHAFHIDCIDTFEFFAAGDDGER---------------------------------EPAENDIR--------CGKR
Bra027290-H2     CWIC---------------------REFVSDNAPD--DRSQDFKCTQCAHKYHGVCLQERS--------------------------------------------KRREPFP--------TYFC
Bra010204-H2     CSVC------------------------LGDYQAD--DKLQQ--ILSGGHTFHMDCIDLWL-------------------------------------------TSHST-----------CPLC
```

Figure S2 Multiple sequence alignment of 262 RING-HC domains of *B. rapa*.

```
                 *  *                                                *   *   *    *                                          *    *
Bra000043-HCa    CAICFDARR-------------------------------------DCFFLPCG--HCVSCY--QCGTKIA--------------------------------EAAGSCPV--C
Bra000106-HCa    CAICLETVFN-------------------------------------PYALKCG--HIF-CK--ACACSAASVMIFQG---------------------VKAAPQCSKCPI--C
Bra000249-HCa    CPVCTNLMY--------------------------------------PPINQCPNGHTL-CS--SCKQQVQN-----------------------------------TCPT--C
Bra000250-HCa    CYICMD-------------------------------------LSKEPVVTNCG--HLY-CW--PCLYRWLEVSVAK------------------------------ECPV--C
```

```
Bra000425-HCa    CPICIEPLS-------------------------------------IPIFQCDNGHLA-CS--SCCPKLKN------------------------------------KCPS--C
Bra000480a-HCa   CCICFEQV-------------------------------------CTIEVKDCG-HQM-CA--QCTLALCCHNKPNPTTS-------------------TVTPP---VCPF--C
Bra000480b-HCa   CTKCQNMIELSEGCA--------------------------------TVQCRCG-HSF-CY--RCG------------------------------------ANAG-CCPH--C
Bra000646-HCa    CPVCLEIPH------------------------------------HSVVLLCSSYHKG-CRPYMCATGNRFSNCLEQYKNAYAKDEK-------------SGKPAELLCPL--C
Bra000729-HCa    CGICMDIIV------------------------------------DRGLLDCCQ-HWF-CF--ECIDNWSS---------------------------------IMNLCPL--C
Bra000784-HCa    CRVCMGRKKGAA-------------------------------------FIPCG-HTF-CR--VCSREVWLNRG---------------------------------SCPL--C
Bra000997-HCa    CPICLSNPK--------------------------------------NMAFGCG-HQT-CC--ECGPDLK-------------------------------------VCPI--C
Bra001159-HCa    CGICLSEEDMRR---------------------------------LKGTLDCCS-HYF-CF--TCIMEWSK---------------------------------VESRCPL--C
Bra001236-HCa    CCVCQVRIKGAT-------------------------------------FTSCG-HTF-CK--LCSKELMAQKG---------------------------------HCPV--C
Bra001285-HCa    CSVCLDRVLSKATPGER----------------------------KFGLLTECH-HPF-CI--QCIRNWRSSAPVSGMD----------------------VNSTLRACPI--C
Bra001477-HCa    CRSCGKGEA-------------------------------------SVLLLPCR-HMCLCS--VCGSS-------------------------------------VYTCPV--C
Bra001901-HCa    CPICNNLYDRA------------------------------------TTISECL-HTFYCK--RCIEDKLVVENL-------------------------------KACPV--C
Bra002208-HCa    CVICLTEPK-------------------------------------DTAVMPCR-HLCLCS--DCAKELRFQSN---------------------------------KCPI--C
Bra002699-HCa    CAVCLERK-------------------------------------CTVSADGCG-HEF-CT--NCALYLSTTTNTSSKTT-------------------SQATPGSVPCPL--C
Bra003133-HCa    CVICLTEPR-------------------------------------DTTVLPCR-HMCMCS--GCAKLLRFQTN---------------------------------LCPI--C
Bra003180-HCa    CMLCQSLLF------------------------------------DSTRCVPCT-HVF-CK--VCLAR---------------------------------FKD----CPL--C
Bra003314-HCa    CNICLD-------------------------------------LSKEPVLTCCG-HLY-CW--PCLFQWLQISEAK-------------------------------ECPV--C
Bra003315-HCa    CPVCTNLMY-------------------------------------PPIHQCPNGHTL-CS--NCKARVQN------------------------------------TCPT--C
Bra003473-HCa    CPVCTNSMY-------------------------------------PPIHQCHNGHTL-CS--TCKNRVHN------------------------------------RCPT--C
Bra003493-HCa    CAVCADNLEW-------------------------------------VAYGSCG-HREVCS--TCVVRLRFVLCD-------------------------------PRCCI--C
Bra003533-HCa    CPICQVDPATP------------------------------------FIALPCQ-HRY-CY--YCIRTRCASAAS-------------------------------FRCLR--C
Bra003669-HCa    CPVCLESPH------------------------------------NAVLLLCSSYHQG-CRPYMCATSSRFANCLDQYRKSYGNSEQ-----------------AQLLCPL--C
Bra003806-HCa    CPICWEPFNVFENV---------------------------------PYVLWCG-HTI-CK--DCLLALHRAVVIKSSGF-------------------PFHLPFFVTCPW--C
Bra004082a-HCa   CKICRQVMTL-------------------------------------PVTTPCA-HNF-CK--ACLEDKFAGQTLVRERSRGGRT--------------LRAQKNVMNCPC--C
Bra004082b-HCa   CSFCIQLPER-------------------------------------PVTTPCG-HNF-CL--KCFE-KWIG-------------------------------QGKRTCAK--C
Bra004174-HCa    CPVCFQALT-------------------------------------QHVFQCDNGHIA-CS--SCCRELRN------------------------------------KCPA--C
Bra004176-HCa    CPTCLEPLR-------------------------------------GPIYQCHNGHIT-CS--PCLPRVYK------------------------------------HCPV--C
Bra004260-HCa    CPICLVIPK--------------------------------------NMAFNCG-HQT-CS--DCGEDLH-------------------------------------VCPI--C
Bra004339-HCa    CRVCFEDPI-------------------------------------NVVLLPCR-HHVLCS--TCCEK-------------------------------------CKKCPI--C
Bra004373-HCa    CPICFESFNIVENV---------------------------------PYVLWCG-HTL-CQ--NCVFALQPAVLRLSS-Q-------------------DIKVPFFVSCPW--C
Bra004401-HCa    CPVCCHALT-------------------------------------NPIFQCDNGHIA-CS--SCCTNLRN------------------------------------KCPY--C
Bra004402-HCa    CPVCCHALT-------------------------------------RPVFQCDNGHIA-CS--SCCTNLRS------------------------------------KCPS--C
Bra004478-HCa    CAVCAENLEW-------------------------------------VAYGFCG-HREVCS--TCVVRLRFILGD-------------------------------RRCCI--C
Bra004666-HCa    CYVCLD-------------------------------------LSKEPVVTNCG-HLF-CW--SCLYRWLQVSEAK-------------------------------ECPV--C
Bra004829-HCa    CNICLE-------------------------------------KAEDPILTCCG-HLF-CW--GCFYQLPFIYLNIK------------------------------ECPV--C
Bra004879-HCa    CKACND-------------------------------------RSKEVVITKCF-HLF-CN--PCVQKIIGTRQ--------------------------------RKCPT--C
Bra005128-HCa    CAICFDAKR-------------------------------------DCFFLPCG-HCVACY--QCGTKIT---------------------------------EAAGSCPI--C
Bra005251-HCa    CSICLNVWHD------------------------------------VVTAAPCL-HNF-CN--GCFS-EWMRS---------------------------EARHQHVLCPQ--C
Bra005352-HCa    CLICMKDEV-------------------------------------SVVFLPCA-HQVVCA--SCSESFM--------------------------------GGDKATCPC--C
Bra005381-HCa    CCVCNASQV-------------------------------------ETLLYRCG-HMCTCL--RCANELQCNGG---------------------------------KCPI--C
Bra005541-HCa    CPICMQVIKD-------------------------------------AFLTACG-HSF-CY--MCIITHLRN--K-------------------------------SDCPC--C
Bra005682-HCa    CGICLEIRNK-------------------------------------VVLPTCN-HSM-CI--NCYRDWRLRSQS---------------------------------CPF--C
Bra005685-HCa    CAICFDAPR-------------------------------------DCFFLSCG-HCVACF--QCGTRIA---------------------------------ETSGFCPV--C
Bra006081-HCa    CAICQEKMH-------------------------------------TPILLRCK-HMF-CE--DCVSEWFER------------------------------ER---TCPL--C
Bra006112b-HCa   CEHAIEFSATG------------------------------TGSSYDVSCLCS--HSF-CW--NCTQDAHRPVDCDRVSQWIRKNS-------VEAENTKWILDNSKPCPK--C
Bra006205-HCa    CSVCHTRYNED---------------------------------ERVPLLLQCG-HGF-CK--DCLSKMFSSSS----------------------------DTSL-ACPR--C
Bra006253-HCa    CPICLSSPK-------------------------------------DMAFG-CG-HQT-CC--ECGPDLE-------------------------------------MCPI--C
Bra006505-HCa    CAICLNAIPLQ----------------------------------ETAMVKGCE-HTY-CV--TCILRWASCKESPT-------------------------------CPQ--C
Bra006634-HCa    CPICLEAFED-------------------------------------AVLTPCA-HRL-CR--ECLLASWRNSSS-------------------------------GLCPV--C
Bra006745-HCa    CGICMEMNSL-------------------------------------AVLPNCT-HSL-CI--KCYRDWRGRSQS---------------------------------CPF--C
Bra007068-HCa    CMLCQALLY------------------------------------DSSRCVPCT-HVF-CK--VCVAR---------------------------------FKD----CPL--C
Bra007376-HCa    CPVCTNLMY-------------------------------------PPIHQCPNGHTL-CS--NCKARVQN------------------------------------TCPT--C
Bra007725-HCa    CVICLDRKC-------------------------------------DAVFLECG-HMCCCL--TCSLSLELQGK---------------------------------RCPL--C
Bra007810a-HCa   CQICLQVMNF-------------------------------------PVTTPCA-HNF-CK--GCLEDKFAGQTRMVERSRGGRT--------------LRAMKNIMKCPC--C
Bra007810b-HCa   CSFCLQLLER-------------------------------------PVTTPCG-HNF-CL--KCFV-KWTE-------------------------------QGKRECIK--C
Bra008335-HCa    CSVCLESPH------------------------------------NAVLLLCSSYHK-GCRPYMCATSTRFANCLDQYRKSHGSEQ------------------LPLLCPL--C
Bra008536-HCa    CSICRN-------------------------------------PFLNPVVTNCN-HYF-CN--LCALM--HHEKNT-------------------------------NCFV--C
Bra008758-HCa    CPICLSNPK--------------------------------------DMAFGCG-HQT-CC--ECGPDLQ-------------------------------------MCPI--C
Bra008900-HCa    CAICLDTIPLQ----------------------------------ETAMVKGCE-HAY-CV--TCILRWASYKEIHT-------------------------------CPQ--C
Bra009089-HCa    CPICISPPRD-------------------------------------IIITRCA-HIF-CR--SCILQTLQRTKP--------------------------------SCPL--C
Bra009278-HCa    CAVCLERT-------------------------------------CTVAAEGCE-HQL-CV--RCALYLCSSSNVPSVT---------------------VDPPGSIPCPL--C
Bra009452-HCa    CVCCDNHI--------------------------------------DALLYRCG-HMCTCS--QCANELVRNGG---------------------------------KCPL--C
Bra009546-HCa    CVICLSEPR-------------------------------------DVLVLPCR-HMCMCV--GCAKELRFQTN---------------------------------LCPV--C
Bra009629-HCa    CVRCDFPIA------------------------------------IYGRLNPCD-HAF-CL--ECAR-------------------------------------SDSICYL--C
Bra010392-HCa    CPLCTFSMY-------------------------------------PPIHQCHNGHTL-CS--TCKVRVHN------------------------------------RCPT--C
Bra010403-HCa    CNICLD-------------------------------------TAHDPVVTLCG-HLF-CW--PCIYKWLHVKLSPVSIDHHH-----------------------NTCPV--C
Bra010714-HCa    CKVCFESPT-------------------------------------AAIPLPCR-HFCLCK--SCSLA-------------------------------------CSECPI--C
Bra010963-HCa    CGICLEPCTK-------------------------------------MVLPNCC-HAM-CI--KCYRNWNTKSES---------------------------------CPF--C
Bra011479-HCa    CPICFGSFTV-------------------------------------PCRGDCG-HWY-CG--SCILQYWN----------------------------YAAVSRPCKCPM--C
Bra011582-HCa    CLNCGSDGET------------------------------------RVLFLPCR-HLCCCT--ECEAG-------------------------------------LVVCPI--C
Bra011923-HCa    CCICFEQA-------------------------------------CTIEVKDCG-HQM-CA--QCTLALCCHNKPNPTTS-------------------TVTPP---VCPF--C
Bra011996b-HCa   CGKCQHMIELSQG-------------------------------CIRIKCRCG--HEF-CY--QCGAE-------------------------------------ARRCPH--G
Bra012331-HCa    CSICHGNFSA-------------------------------------PCQANCS-HWF-CG--NCIMLVWR----------------------------HGSTLRPCKCPL--C
Bra012354-HCa    CPICCEPLK-------------------------------------IPIFQCENGHLA-CS--QCCEKVKK------------------------------------ICPS--C
Bra012512-HCa    CGICLEPCTK-------------------------------------MVLPNCC-HAM-CI--KCYRNWNTKSES---------------------------------CPF--C
Bra012532-HCa    CKRCGEREA-------------------------------------SVLVLPCR-HLCLCT--VCGSA--L----------------------------------LQTCPV--C
Bra012650-HCa    CSICRRNSA-------------------------------------TVMALPCR-HLVLCK--GCDGGGD-----------------------------------VRVCPI--C
Bra013017-HCa    CKICLIKEV-------------------------------------DITIVPCG-H-VLCR--ECSA-------------------------------------SVARCPF--C
Bra013241a-HCa   CCVCLELLYK-------------------------------------PIVLSCG-HLS-CF--WCVHKSMNELRE-------------------------------SHCPI--C
Bra013241b-HCa   CSACKELLVR-------------------------------------PVVLNCG-HVY-CE--GCVVDMF-QEGEK------------------------------IKCQE--C
Bra013423-HCa    CKRCGEREA-------------------------------------SVLVLPCR-HLCLCT--VCGSA--L----------------------------------LRTCPV--C
Bra013447-HCa    CCVCSEMPV-------------------------------------ESLLYRCG-HMCTCL--KCAHELQWSSK---------------------------------KCPI--C
Bra013499-HCa    CADCHLAIKLVSKVYEREIVVRDRSRFHHFKDGSCSCQDYCLSLFDSAASLSCN-HV-FCN--ECIVKSMKV---------------------------------DATCPV--C
Bra013775-HCa    CVICFEERR-------------------------------------NCFFVPCG-HSATCR--GCAEKIMS--------------------------------EENKVCPI--C
Bra014051-HCa    CRFCGVREV-------------------------------------CMLLLPCK-HMCLCK--ECERK-------------------------------------LSSCPL--C
Bra014247-HCa    CVICEDASS-------------------------------------EAACVPCG-HVAGCV--SCLKEIK---------------------------------NKK--CPI--C
Bra014421-HCa    CPVCTNSMY-------------------------------------PPIHQCQNGHTL-CS--TCKNRVHN------------------------------------RCPT--C
Bra014589-HCa    CPVCTNLMY-------------------------------------PPIHQCPNGHTL-CS--NCKARVQN------------------------------------TCPT--C
Bra014591-HCa    CNICLD-------------------------------------VSKEPVLTCCG-HLY-CW--LCLFQWLNISEAK-------------------------------ECPV--C
Bra014932-HCa    CLICMDAPS-------------------------------------DGICVPCG-HVAGCM--SCLTQIK---------------------------------SHASECPI--C
Bra014934-HCa    CSICLDAPS-------------------------------------EAVCVPCG-HVAGCM--SCLTEIK---------------------------------AKKWGCPV--C
Bra015086a-HCa   CCVCLELLYK-------------------------------------PIVLSCG-HLS-CF--WCVHRSMSGLRV-------------------------------SHCPI--C
Bra015086b-HCa   CSACKELLVR-------------------------------------PVVLNCG-HVY-CE--GCVVDMMDQESEK------------------------------IKCKE--C
Bra015092-HCa    CCVCMVRSKGAA-------------------------------------FIPCG-HTF-CR--LCSRELWVQRG---------------------------------NCPL--C
Bra015262-HCa    CPICLGIIRKT------------------------------------RTFMECL-HRF-CQ--ECIDKSMRFGN--------------------------------HECPA--C
Bra015389-HCa    CGLCHESAED-------------------------------------SVVTSCS-HVF-CK--ACLIDFSASLGK-------------------------------VSCPT--C
Bra015392-HCa    CGLCHEPAED-------------------------------------NVVTSCA-HVF-CK--ACLIDFSASLRK-------------------------------VSCPT--C
Bra015877-HCa    CNICLE-------------------------------------MAREPIVTLCG-HLF-CW--PCLYKWIHFHSQSK------------------------------HCPV--C
Bra015952-HCa    CRICFEREI-------------------------------------SVVLLPCR-HRVLCR--NCSEK-------------------------------------CKKCPF--C
Bra016082-HCa    CSICHSHFTA-------------------------------------PCQANCS-HWF-CG--NCIMLVWR----------------------------HGSTLQPCKCPL--C
```

```
Bra016385-HCa      CCVCLERVLSKPTPAER----------------------------KFGILTECD--HAF-CI--GCIRNWRSSSPSTGMD----------------------VNSTLRACPI--C
Bra016557-HCa      CRVCFEREI-------------------------------------SLVLLPCR--HRVLCR--LCSDK-------------------------------------CKKCPA--C
Bra016898-HCa      CLACTNLMY--------------------------------------PPIFQCPNGHTL-CS--TCKLKVHN------------------------------------TCPT--C
Bra017091-HCa      CPICLENLTHR----------------------------------RAAVIPSCR--HGY-CL--GCIRKWSGLKRS---------------------------------CPL--C
Bra017094-HCa      CAICLETVFN-------------------------------------PYALKCG--HIY-CK--ACACSSASVMIFQG----------------------VKAAPQCSKCPI--C
Bra017511-HCa      CRICRRRAA-------------------------------------TVLALPCR--HLVLCK--GCDG--S-----------------------------------VRVCPL--C
Bra017614-HCa      CPICFSSFTV-------------------------------------PCRGNCG--HWY-CG--SCILQYWN----------------------------YAAVSKPCKCPM--C
Bra017691-HCa      CLNCGLNGET------------------------------------RVLFLPCR--HLCCCT--GCEDG-------------------------------------LVLCPI--C
Bra017935-HCa      CPVCLQSYDGESTL---------------------------------PRVLSCG--HTA-CE--ECLTNI------------------------------PKKFPDTIRCPA--C
Bra018162-HCa      CGICMEMNSM-------------------------------------VVLPNCT--HSL-CI--KCYRDWHGRSES---------------------------------CPF--C
Bra018221-HCa      CEYAIELH-EDLLEDDDLDFG---------------------------VVCLCG--HTF-CW--SCTLESHRPVTCNDASVWFS------STLD-TLKSNAWLLENTKRCPN--C
Bra018266-HCa      CPICCVPLT--------------------------------------SNIFQCDNGHIA-CP--TCCNKLRN------------------------------------KCPS--C
Bra018428-HCa      CKMCHGKEV-------------------------------------SVLVVPCR--HLSLCK--ECDV--F-----------------------------------TGFCPV--C
Bra018847-HCa      CRACND-------------------------------------PPEAPVITLCG--HIF-CY--QCVSEYITGDE--------------------------------NSCPV--C
Bra019048-HCa      CNICLD-------------------------------------TALDPVVTLCG--HLF-CW--PCIYKWLHVQLSSLSTDHHRR----------------------NNCPV--C
Bra019242-HCa      CVICFEERR-------------------------------------NCFFVPCG--HSATCK--GCALRIMS--------------------------------EENKVCPI--C
Bra019391-HCa      CCVCMGRKRGAA-------------------------------------FIPCG--HTF-CR--VCSRELWLNRG---------------------------------SCPL--C
Bra019817-HCa      CGICLETCTK-------------------------------------MVLPNCC--HSM-CI--KCYRNWNLKSQS---------------------------------CPF--C
Bra019910-HCa      CRMCNAREV-------------------------------------SVLLVPCR--HLSLCK--ECDV--F-----------------------------------TGVCPV--C
Bra020414-HCa      CAVCLERK-------------------------------------CTVAADGCG--HEF-CT--NCALYLSTTNITSSKT--------------------SQATPGSVPCPL--C
Bra020415-HCa      CAVCLERKCTVAA-------------------------------------DGCG--HEF-CT--NWELYLSTTNRTSSKT--------------------PQATPGSDPCPL--C
Bra020415-HCa      CAVCLERK-------------------------------------CTVAADGCG--HEF-CT--NWELYLSTTNRTSSKT--------------------PQATPGSDPCPL--C
Bra020684-HCa      CPICMCPFTE-------------------------------------EMSTKCG--HIF-CK--GCIKM---------------------------------AISKQGKCPT--C
Bra020773-HCa      CVICMTEAK-------------------------------------DTAVLPCR--HLCMCS--DCAKELRLQSN---------------------------------KCPI--C
Bra020883-HCa      CPICLSLFN-------------------------------------SAVSLTCN--HVF-CN--ACIVK---------------------------------SMKVDATCPV--C
Bra021742b-HCa     CPRCKRHIE---------------------------------------KNQGCN--HM-TCSA-PCR---------------------------------------FQFCWI--C
Bra021889-HCa      CPVCLGLIPKNA---------------------------------VIKVLPNCK--HMF-YE--ECIARWL---------------------------------ESHTTCPV--C
Bra022858-HCa      CPVCCHALT--------------------------------------NPIFQCDNGHIA-CS--ACCINMRN------------------------------------KCPS--C
Bra023148-HCa      CCVCD-ETQV------------------------------------EAVLYMCG--HMCTCL--KCANELHWSGG---------------------------------KCPI--C
Bra023274-HCa      CIICMKDEV-------------------------------------SVVFLPCA--HQVVCG--SCSDNFFSSNN---------------------------GGGGKVTCPC--C
Bra023282-HCa      CKGCRTREA-------------------------------------TVVMLPCR--HLSICP--ECDR--T-----------------------------------ALACPL--C
Bra023465-HCa      CPICLSNPK-------------------------------------DMAFG-CG--HQT-CC--ECGPDLQ-------------------------------------VCPI--C
Bra024213-HCa      CNICLD-------------------------------------QVRDPVVTLCG--HLF-CW--PCIYKWTHCTNNTRQRVDQYDKKE------------------SSKCPV--C
Bra024254-HCa      CPICLTKPK-------------------------------------DMAFS-CG--HTT-CK--ECGVVVK-------------------------------------TCPM--C
Bra024967-HCa      CRICWRRSA-------------------------------------TVLALPCR--HLILCK--GCDG--S-----------------------------------VRVCPL--C
Bra025434-HCa      CPICYEPLT--------------------------------------VPIFQCDNGHLA-CS--SCCPKLSN------------------------------------KCHA--C
Bra025676-HCa      CTVCLKLLY-------------------------------------EPATTPCG--HTF-CR--SCLFQ---------------------------------SMDRGNKCPL--C
Bra025727-HCa      CNICFD-------------------------------------LAQDPIVTLCG--HLF-CW--PCLYKWLHLHSTSS------------------------------ECPV--C
Bra026271-HCa      CNICLD-------------------------------------QVRDPVVTLCG--HLF-CW--PCIYKWTYSTNNTRRRVDQYDKKE------------------SPKCPV--C
Bra026301-HCa      CPVCTCSMY--------------------------------------PPIHQCHNGHTL-CS--TCKVRVHN------------------------------------RCPT--C
Bra026346-HCa      CNICLD-------------------------------------TAHDPVVTLCG--HLF-CW--PCIYKWLHVQLTSVSIDQHH-----------------------SNCPV--C
Bra026920-HCa      CGICLETCTK-------------------------------------MVLPNCC--HSM-CI--KCYRNWNLKSQS---------------------------------CPF--C
Bra027538-HCa      CPICLGIIKKT------------------------------------RTVMECL--HRF-CR--ECIDKSMRLGN--------------------------------KECPA--C
Bra027588-HCa      CKRCGERTA-------------------------------------SVLVLPCR--HLCLCT--VCGSA--L----------------------------------LQACPV--C
Bra027667-HCa      CVICLEHNY-------------------------------------NAVFVPCG--HMCCCT--AC--SSHLTS-----------------------------------CPL--C
Bra028153-HCa      CPICFEAFT--------------------------------------IPIFQCENGHLA-CS--SCCPKISN------------------------------------KCPT--C
Bra028364-HCa      CKRCGERTA-------------------------------------SVLVLPCR--HLCLCT--VCGSA--L----------------------------------LQSCPV--C
Bra028395-HCa      CPICCEPLK--------------------------------------APIFQCDNGHLA-CS--VCCTKVRN------------------------------------ICPS--C
Bra028419-HCa      CVICVDASA-------------------------------------EVACVPCG--HVAGCI--SCLKEIK---------------------------------NKKLGCPI--C
Bra028646-HCa      CPDCDDYAIKRHDEVPSEDF---------------------------DVVCLCG--HIF-CW--SCQLESHRPVTCNNASFWLN-------ELLYKSRNLARTAKRIKHCPE--C
Bra028846-HCa      CVVCLSELR-------------------------------------DTIVLPCR--HMCMCS--GCAKALRFQKN---------------------------------ECPV--C
Bra029623-HCa      CCVCHVYIKGAT-------------------------------------FAPCG--HTF-CK--LCSKELMAQKG---------------------------------HCPV--C
Bra029631-HCa      CPICMCPFTE-------------------------------------EVSTKCG--HIF-CK--KCIKL---------------------------------AVSVQAKCPT--C
Bra029788-HCa      CVICLSEPR-------------------------------------DTTVLPCR--HMCMCS--GCAKVLRFQTN---------------------------------RCPI--C
Bra029846-HCa      CCVCCDTDI-------------------------------------DALLYRCG--HMCTCS--NCGNELVRTGG---------------------------------KCPL--C
Bra030604-HCa      CGLCHEPAED-------------------------------------NVVTSCE--HVF-CK--ACLIDFAASLGE-------------------------------VSCPT--C
Bra030874-HCa      CGVCFD-------------------------------------RPKEVVIMKCF--HLF-CK--QCIQRSLEIRH--------------------------------RKCPG--C
Bra030900-HCa      CVICVTRRR-------------------------------------IPAFIPCG--HVVCCR--YCALTVER--------------------------------GLNPKCPV--C
Bra031030-HCa      CRVCFEKEI-------------------------------------SLVLLPCR--HRVLCR--ICSDK-------------------------------------CTKCPI--C
Bra031038-HCa      CTVCLKLLY-------------------------------------DPATTPCG--HTF-CR--SCLFQ---------------------------------SMDRGNKCPL--C
Bra031184-HCa      CKVCFESPT-------------------------------------ATILLPCR--HFCLCK--CCSLA-------------------------------------CSECPI--C
Bra031225-HCa      CCICYAGDAN-------------------------------------AVLVPCS--HRS-CY--GCI-TRHL-----------------------------------LNCQR--C
Bra031418-HCa      CPSCKVTLTNTM---------------------------------SLVALSSCG--HVF-CK--KCGEK---------------------------------FMPVDKVCLV--C
Bra031420-HCa      CPSCKVTLTNTM---------------------------------SLVALSSCG--HVF-CK--KCGEK---------------------------------FMPVDKVCLV--C
Bra031495-HCa      CKSCNVKEV-------------------------------------SVLLVPCR--HLSLCK--DCDV--F-----------------------------------TGFCPV--C
Bra031942-HCa      CPICLTNPK--------------------------------------DMAFSCG--HTT-CK--ECGVVVK-------------------------------------ICPM--C
Bra032048-HCa      CPICFHPLT--------------------------------------IPIFQCDNGHIV-CS--TCCEKLSQ------------------------------------KCAT--C
Bra032145-HCa      CNICFE-------------------------------------LAQDPIVTLCG--HLF-CW--PCLYRWLHHHSHSQ------------------------------ECPV--C
Bra032532-HCa      CPLCLKLLN------------------------------------QPVLLP-CD--HIF-CD--SCIHE---------------------------------SSQVESGCPV--C
Bra032581-HCa      CSICLDTVFD-------------------------------------PISLTCG--HIY-CY--MCACSAASVNVVDG----------------------LKAADPSEKCPL--C
Bra032793-HCa      CPICLVKTK--------------------------------------NMAFNCG--HQT-CD--ECGEAIQ-------------------------------------TCPI--C
Bra032944-HCa      CPICLEYPLC-------------------------------------PQITSCG--HIF-CF--PCVLQYLLIGEDNHKAECF------------------------KRCPL--C
Bra033002-HCa      CAICLEICFE-------------------------------------PSTTTCG--HSF-CK--KCLRSAAD-----------------------------KCG---RKCPK--C
Bra033065-HCa      CFWCNKSML-------------------------------------LWANFPCR--HKLWCS--SCKHK------------------------------IAESAGDHHRCVI--C
Bra033093-HCa      CPICWESFNIVENV---------------------------------PYVLWCG--HTM-CK--NCILGLQWAIVKLPT-H-------------------PVQLPLFISCPW--C
Bra033268-HCa      CFICRE-------------------------------------SFVDPVVTKCK--HYF-CE--HCALK--HHTKNK-------------------------------KCFV--C
Bra033353-HCa      CSICLDTVFD-------------------------------------PISLTCG--HIY-CY--MCACSAASVNVVDG----------------------LKTADPSEKCPL--C
Bra033393-HCa      CVMCLSEEM-------------------------------------SVIFLPCA--HQVLCF--TCNQLHE--------------------------------KEGMKDCPS--C
Bra033597-HCa      CKVCFESPT-------------------------------------AAFLPPCR--HFCLCK--SCSLA-------------------------------------CSECPI--C
Bra033682-HCa      CPICLESADD-------------------------------------PILTPCA--HRM-CR--ECLLTSWRSTSC-------------------------------GLCPI--C
Bra033732-HCa      CPICLGIIKKT------------------------------------RTVMECL--HRF-CR--ECIDKSMRLGN--------------------------------NECPA--C
Bra033848-HCa      CPICLTIRKD--------------------------------------VAFGCG--LMI-CR--DCGSR-------------------------------------ISNCPI--C
Bra034017-HCa      CVICWTGFS------------------------------------STRGILPCG--HRF-CY--SCIQQWVDRLVS---------------------------ERKKTTCPL--C
Bra034086-HCa      CVICLSEPS-------------------------------------DTTVLPCR--HMCMCS--GCAKVLRFQTN---------------------------------RCPI--C
Bra034121-HCa      CVCCDADI--------------------------------------DALLYRCG--HMCTCS--KCGYELVRTGG---------------------------------KCPL--C
Bra034212-HCa      CNICLD-------------------------------------SVQEPVVTLCG--HLF-CW--PCIHKWLHVQTDEHQRQHK-------------------------QCPV--C
Bra034501-HCa      CCVCMGREKGAA-------------------------------------FIPCG--HTY-CR--VCSREIWMNRG---------------------------------SCPL--C
Bra034718-HCa      CRGCGKGEA-------------------------------------SVLLLPCR--HMCLCT--VCGSS-------------------------------------VNTCPV--C
Bra035092-HCa      CRSCGEEES-------------------------------------CVLVLPCR--HLCLCG--VCGSS-------------------------------------VHTCPI--C
Bra035112-HCa      CPICLTNRK--------------------------------------DVAFGCG--HMT-CG--ECGSRIS-------------------------------------NCPI--C
Bra035664-HCa      CCICFEQV-------------------------------------CTIEVKDCG--HQM-CA--QCTLALCCHNKPNLTTS-------------------TVNPP---VCPF--C
Bra036164-HCa      CPICMCPFTE-------------------------------------EMSTKCG--HIF-CK--GCIKM---------------------------------AISRQNKCPT--C
Bra036227-HCa      CVMCLSEEM-------------------------------------SVIFLPCA--HQVLCS--KCNQLHE--------------------------------KEAMDDCPS--C
Bra036439-HCa      CPFCEL-------------------------------------PFLDPVVTTCD--HYF-CN--LCARK--HHKKDP-------------------------------TCFV--C
Bra037471-HCa      CPICMCPFTE-------------------------------------ETSTKCG--HIF-CK--GCIKT---------------------------------AISRQAKCPT--C
Bra037967-HCa      CVICVTRRR-------------------------------------VPAFIPCG--HVVCCR--QCASTVER--------------------------------EVNPKCPV--C
Bra038276-HCa      CEYAIELH-EDLLEDDDLDFG---------------------------VVCLCG--HTF-CW--SCTLESHRPVTCKEASVWSS------STLD-TLKSNAWLLENTKRCPN--C
Bra038348-HCa      CKVCFENPV-------------------------------------NVVLLPCK--HYVLCS--TCCKK-------------------------------------CKTCPI--C
Bra038415-HCa      CCVCLERVLSKPTPAER----------------------------KFGLLTECD--HAF-CI--GCIRNWRSSSPSTGMD----------------------VNSTLRACPI--C
Bra038515a-HCa     CQICRKVMSS-------------------------------------PVTTPCA--HNF-CK--ECLTGKFAGITQVRQRSRGGRT--------------LRAQKNVMKCPC--C
```

```
Bra038515b-HCa  CSICLQLPER-------------------------------------PVTTPCG--HNF-CL--KCFQ-KWVAG------------------------------KRKPTCGT--C
Bra038659-HCa   CPGCSYFIEFHKGTDA--------------------------EEYGLNVVCLCG--HNF-CC--RCSLETHRPVTCNNASDWLSRDLQKLSEASDKSLSFLWIEDNTETCPH--C
Bra039105-HCa   CPICLSNPK--------------------------------------NMAFGCG--HQT-CC--ECGPGLK-------------------------------------VCPI--C
Bra039354-HCa   CRSCGKGEA-------------------------------------SVLLLPCR--HMCLCS--VCGSS-------------------------------------INTCPI--C
Bra039449-HCa   CPICLGPIL------------------------------------QESYLDTCF--HKF-CF--RCIKQWIKVVSSKVSK-----------------------PLSSVKCPL--C
Bra039533-HCa   CRRCGERRA-------------------------------------SVLVLPCR--HLCLCT--VCGSA--L----------------------------------LQACPV--C
Bra039871-HCa   CCVCDETQV-------------------------------------EAVLYRCG--HMCTCL--KCANELHWSGG---------------------------------KCPI--C
Bra040048-HCa   CSVCLDRVLSKATPGER----------------------------KFGLLTECH--HPF-CI--QCIRNWRSSAPVSGMD----------------------VNSTLRACPI--C
Bra040255-HCa   CRFCGVGEV-------------------------------------CMLLLPCK--HMCLCK--ECERN-------------------------------------LSSCPL--C
Bra040332-HCa   CKACND-------------------------------------RSKEVVITKCF--HLF-CN--PCVLKITGTRQ--------------------------------RKCPT--C
Bra040362-HCa   CCVCQVNIKGAT-------------------------------------FTPCG--HTF-CK--LCSKELSAQKG---------------------------------HCPV--C
Bra040424-HCa   CVVCLERKC-------------------------------------DAAFVPCG--HMCCCL--TC--ALKLLGK---------------------------------PCPL--C
Bra000504a-HCb  CSICIDDNIS---------------------------------AIQMFSVDICG--HRF-CS--ECVKRYIEAKLLEGNR---------------------------LTCPHDGC
Bra001543-HCb   CMICMDEKSP---------------------------------SDIFRGTTSCT--HYY-CT--ECTVRYVMTKIEGNIA--------------------------MIKCPDVDC
Bra001771-HCb   CNICND-------------------------------------PPENPVISLCG--HVF-CY--QCVSEHINGDE--------------------------------NVCPVRRC
Bra001960-HCb   CDICVEDVE----------------------------------GHHHMTRMDCG--HCF-CN--NCWAAHFTVKINEGQ-SK------------------------RIRCMAYKC
Bra002501a-HCb  CLICFNDID----------------------------------PERMFSIGKCS--HRF-CF--QCVKQHVEVKLLHGMI---------------------------PNCPHDKC
Bra002501b-HCb  CGKCQHMIELSQGCN--------------------------------HITCRCG--HEF-CY--NCGGG---------------------------------WNKKTGTCVKQ-C
Bra002502a-HCb  CLICFNDID----------------------------------PERMFSIGKCS--HRF-CF--QCVKQHVEVKLLHGMI---------------------------PNCPHDKC
Bra002502b-HCb  CGKCQHMIELSQGCN--------------------------------HITCRCG--HEF-CY--NCGGG---------------------------------WNKITGTCAKQ-C
Bra005642-HCb   CGICFESYL-----------------------------------RKEIATVSCG--HPY-CK--TCWTSYITEKINNGPGCL------------------------MIKCPEPSC
Bra006057-HCb   CPICLCEVDDG------------------------------------YSLEGCS--HLF-CK--ACLLEQLEASMRNFD-------------------------AFPILCSHTDC
Bra006112a-HCb  CGICFDYFC-----------------------------------RIDIVPIMCG--HAF-CS--TCWTSYISIAINDGPGCL------------------------MQKCPEPSC
Bra006671-HCb   CLICYNETD----------------------------------IERMFSVAKCR--HRF-CF--HCSKQHVEVKLLHGTV---------------------------PNCPHDGC
Bra007078-HCb   CPACYAHVSP---------------------------------RHKLEVGSGCF--HRI-CF--TCIRDCVSSQLARGD---------------------------TLLCPYPGC
Bra007801-HCb   CAICLEETD----------------------------------AGRMFSTEQCL--HRH-CF--SCVKQYVEVKLLSGIV---------------------------PTCLGEGC
Bra009038-HCb   CPICLSEVEDG------------------------------------YSLEGCS--HLF-CK--ACLLEQLEASMRNFD-------------------------AFPIICSHIDC
Bra010152-HCb   CCICFSESAG-----------------------------------FDFVKLPCE--HFF-CV--KCMKTYTDIHVSEGTVN-------------------------KLQCPDSKC
Bra011524-HCb   CDVCLEDVV----------------------------------AEQ-MKRMDCG--HCF-CN--DCWTEHFTVQINEGQ-SK------------------------RIRCMAHKC
Bra011996-HCb   CAICLDEDIN---------------------------------ADQMFSVDKCG--HWF-CS--ECVKRHIEVKLLEQGF---------------------------VRCPQHRC
Bra012536-HCb   CSICCEDRR-----------------------------------AEMMLTLKCT--HKS-CS--YCMKTYVQGRVESSEV--------------------------PIRCPQVQC
Bra013417-HCb   CSICCEDRL-----------------------------------SEMMLTLKCT--HKF-CS--HCMKTYVEGKVNSSEV--------------------------PIRCPQLQC
Bra014815-HCb   CKRCGEWCR-------------------------------------LPVITPCR--HLL-CL--DCVS------------------------------------LDSERCTFPGC
Bra015067-HCb   CGICFDTDFN---------------------------------AEQMFCVASCG--HEF-CV--ECVKRHIEVRVFEGDVH------------------------IRCPCY-YYC
Bra015460-HCb   CGICFKLLSL-----------------------------------ERSASVSCG--HRV-CK--FCWRSHINKSINKIADVDWYG---------------------TLKCPYDIC
Bra015463-HCb   CGICFEPFQ-----------------------------------REEVVSLACG--HPF-CS--TCWTGYISTTINDGPGCL------------------------MLKCPEPSC
Bra018584-HCb   CPACYTHVSR---------------------------------PHKLEVRSGCF--HLI-CF--TCIRDCVSSQLARGD---------------------------TVLCPYPGC
Bra020455a-HCb  CIICLDDVD----------------------------------SDLMFYVERCG--HRF-CI--NCVKQHINVKLVDGKI---------------------------PNCPHHGC
Bra020457-HCb   CKICLDDVD----------------------------------SDLMFYVERCG--HRF-CI--NCVKQHINVKLVDGKI---------------------------PNCPHHGC
Bra021726-HCb   CGICFDPYP-----------------------------------PEKIASVSCG--HPF-CT--TCWTGYISTTINDGPGCL------------------------MLRCPDPSC
Bra021742a-HCb  CGICFESYF-----------------------------------QKDIATVSCG--HPY-CM--TCWNGYITAKITGGPGCL------------------------MVTCPEPSC
Bra022479-HCb   CGICFETYL-----------------------------------SDKLHAAACG--HPF-CD--SCWEGYISTAINDGPG-----------------------------CLMLRC
Bra022845-HCb   CGICFDSYP-----------------------------------PEKFVSVSCG--HPF-CT--TCWTGYITTTINDGPGCL------------------------MLRCPDPSC
Bra022865a-HCb  CGICFDSYT-----------------------------------QKEVTTLSCG--HPY-CN--VCWTGYITTTINDGPG-----------------------------CLTVKC
Bra023338-HCb   CGICFDKFR-----------------------------------RKKIVPVVCG--HTF-CS--TCWNGYITTAINDGSGCL------------------------MQKCPEPSC
Bra026648a-HCb  CAICLDDDVN---------------------------------ADQMFTVNKCG--HRF-CS--ECVKRHIEVRLLEGSV---------------------------MTCPQFRC
Bra026648b-HCb  CGKCQHMIEL--------------------------------SKGCVLVVCRCG--HKF-CY--RCGANARSCTHGLGHMFPPQPQ-------------ELESPPPAPPCWAQ-C
Bra027352-HCb   CMICMDEKPS---------------------------------SDMFRGSVTCT--HAY-CT--QCTIRYVASKIKENSA--------------------------RIKCPDVEC
Bra029725-HCb   CMICMDEKSP-----------------------------------SDMFRGNCT--HSY-CT--ECTVRYVETKIGENVA--------------------------GIKCPDVDC
Bra031452-HCb   CGICND-------------------------------------SPEDAVVSVCG--HVF-CK--QCFYECFTGDD--------------------------------NLCPIANC
Bra033024-HCb   CDICMEDDL----------------------------------PSHAMTGMECG--HSF-CN--DCWKEHFTIKINEGQ-SK------------------------RIACMAHKC
Bra034292-HCb   CPICFDDDLE---------------------------------AHQMFSVALCC--HQF-CF--ECVRRYINVGLMEGRA---------------------------FGCPHFKC
Bra035824-HCb   CNICDD-------------------------------------PPENPVVTLCG--HVF-CY--QCVSEHITGDE--------------------------------NVCPVRRC
Bra037371-HCb   CPACYAHVSP---------------------------------RHKLEVRSGCF--HRI-CF--TCIRDCVSSQLARGD---------------------------SVLCPYPGC
Bra037375-HCb   CVICYEDVPA---------------------------------DTKFTV-SGCF--HRI-CF--ECMRNYITHSLRHRS---------------------------RLICPNTGC
Bra038285a-HCb  CVICYEGIT----------------------------------VDKMFSVDGCF--HRF-CF--SCMKQHVEIKLLGGKT---------------------------ATCPSDGC
Bra038306-HCb   CLICMDEKSP---------------------------------SDIFRGTTSCT--HSY-CT--ECTVRYVTTKVEENAARA------------------------RIKCPDMNC
Bra038573-HCb   CGICND-------------------------------------SPEDAVVSVCG--HVF-CK--QCFYECLTGDN--------------------------------NICPNVNC
Bra039068-HCb   CEICMDDDL----------------------------------QSYTMTRMDCG--HCF-CN--NCWKEHFTVKINEGM-SK------------------------RITCMAHKC
```

Figure S3 Multiple sequence alignment of 44 RING-v domains of *B. rapa*.

```
            *  *                                                                               * *                         *  *               *  *
Bra000030-V CRICHLGVVETSG----GGA-----------------------------------------------------------IELGCSCKEDLAGAHRQCAGTWFKIKGDKLVAHRQCAETWFKIKG---DKICEIC
Bra001193-V CRICL-DVLEEGNT-----------------------------------------------------------------LKMECSCKGDLR------------------LVHEACAIKWFSTKG---TRTCDVC
Bra001210-V CRICRSAEEPGNA------------------------------------------------------------------LMHPCSCRGSIK------------------YVHHDCLLIWLNRRG---YKQCEVC
Bra002470-V CRICLVDLCEGGET-----------------------------------------------------------------FKMECSCKGELA------------------LAHKDCALKWFTIKG---NKTCEVC
Bra002600-V CRICHLPLETTNKADEECEDSDEQEEQGEEEDEE-------------------------------------EEEYYGLPLQLGCSCKGDLG------------------VAHSKCAETWFKIKG---NMTCEIC
Bra005147-V CRICHLGVVETSG----GGA-----------------------------------------------------------MELGCSCKEDLA------------------IAHRQCAETWFKIKG---DKICEIC
Bra005444-V CRICHVGSDQTPDR--VSGKTV----------------------------------------------------VSLELIQIGCKCKNELA------------------LAHFHCAEAWFKLRG---NSVCEIC
Bra005697-V CRICHLGLESSRR---ECGD----------------------------------------------------------PMVLGCSCKDDLG------------------YVHKQCAETWFKIKG---DKTCEIC
Bra009138-V CRICHMTLDATNL---ESGV----------------------------------------------------------GIELGCSCKDDLA------------------AAHKHCAETWFKIKG---NKICEVC
Bra009367-V CRVCQCAESDRRGD-AALGFLGITPPVSEPRRSSNAKKQETTDHKTGGFIELISPDGEVFVCANEDIEMGAWQ-HRDTLLELGCSCKNDLA------------------LVHYACALKWFVNHG---STVCEIC
Bra010084-V CRICQDED-SVKN------------------------------------------------------------------LECPCSCSGSLK------------------YAHRKCVQRWCNEKG---DTTCEIC
Bra011370-V CRICQSPEEPNNP------------------------------------------------------------------LRHPCACRGSLK------------------YVHTDCIFLWINRRR---SKHCEIC
Bra011487-V CRICRNPGEADNP------------------------------------------------------------------LRYPCACSGSIK------------------FVHQDCLLQWLNHSN---ARQCEVC
Bra014329-V CRICHDED-LDCN------------------------------------------------------------------METPCSCSGSLK------------------YAHRRCVQRWCNEKG---DTTCEIC
Bra014845-V CRICKSEVG--YG----QGL-----------------------------------------------------------IELGCSCKGDLA------------------FSHRQCAETWFKLKG---NQVCEIC
Bra017626-V CRICRNPGDADNP------------------------------------------------------------------LRYPCACSGSIK------------------FVHEDCLLQWLSFSK---ARHCEVC
Bra018136-V CRICQEED-ITNN------------------------------------------------------------------LETPCACNGSLK------------------YAHRKCVQRWCNEKG---DIICEIC
Bra018414-V CRICLENDSDLLG----------------------------------------------------------------DELISPCMCKGTQQ------------------FVHRSCLDHWRSVKEGFAFSHCTTC
Bra019507-V CRICQEED-TIQN------------------------------------------------------------------LEAPCACNGSLK------------------YAHRKCVQRWCNEKG---DITCEIC
Bra020240-V CRICLVDLCEGGET-----------------------------------------------------------------LKMECSCKGELA------------------LAHKDCALKWFTIKG---NKTCEVC
Bra020758-V CRICL-DECEEGNT-----------------------------------------------------------------LKMECSCKGDLR------------------LVHEACAIKWFSTKG---TRTCDVC
Bra022967-V CRICHFGSDQTPDR--VSGKS-----------------------------------------------------VSVDLIEIGCKCKNELG------------------LAHFHCAEAWFKLRG---NSVCEIC
Bra023691-V CRICL-DVCEEGNT-----------------------------------------------------------------LKMECSCKGDLR------------------LVHEHCAIKWFSTKG---TRICDVC
Bra024797-V CRICQDEC-PIKT------------------------------------------------------------------LESPCACSGSLK------------------YAHRKCVQRWCNEKG---NIICEIC
Bra024893-V CRICHDED-LDSN------------------------------------------------------------------METPCSCSGSLK------------------YAHRRCVQRWCNEKG---NTNCEIC
Bra026840-V CRICQEES-AIKN------------------------------------------------------------------LESPCSCNGSLK------------------YAHRKCVQRWCNEKG---NTICEIC
Bra028398-V CRICHDED-EDSN------------------------------------------------------------------MDTPCSCSGTLK------------------FAHHQCIQRWCNEKG---DTLCEIC
Bra028747-V CRICHMSLDSVNL---ESGV----------------------------------------------------------PIELGCSCKNDLA------------------AAHKHCAETWFKIKG---NKICEVC
Bra028847-V CRICMVELGEDSEA-----------------------------------------------------------------FKMECMCKGELA------------------LSHKACTIKWFTIKG---NITCDVC
Bra029202-V CRVCHSAESDKRGD-AALGFLGIT------LKTNADET-----VDDN--VSKST---------ETDIEMGILQQHQDPLLELGCSCKNELA------------------LVHYACALKWFLNHG---STVCEIC
Bra029259-V CRICQEED-SVKN------------------------------------------------------------------LESPCSCSGSLK------------------YAHRKCVQRWCNEKG---DTICEIC
Bra029597-V CRICRSPEEPGNP------------------------------------------------------------------LRYPCLCRGSIK------------------FVHQDCLRTWLIRRG---NNKCEVC
Bra029786-V CRICLVELGEDSEA-----------------------------------------------------------------FKMECMCRGELA------------------LAHKECTIKWFTIKG---NRTCDVC
Bra029787-V CRICMVELGEDSEA-----------------------------------------------------------------FKMECMCRGELA------------------LAHKECTIKWFTIKG---NRTCDVC
Bra030264-V CRICLETD----G----------------------------------------------------------------RDFIAPCKCKGTSK------------------YVHRDCLDHWRAIKEGFAFAHCTTC
Bra030491-V CRICLDVG----G----------------------------------------------------------------EDLIGPCNCKGTQK------------------YVHRSCLDNWRSTKEGFAFSHCTEC
Bra030505-V CRICHEEE-PESY------------------------------------------------------------------FEAPCSCSGTLK------------------FAHRDCIQRWCDEKG---NTICEIC
Bra031231-V CRICLESD----G----------------------------------------------------------------RDFIAPCKCKGTSK------------------YVHRDCLDHWRAIKEGFAFAHCTTC
Bra031736-V CRICLENDCELLG----------------------------------------------------------------DELISPCMCKGTQQ------------------LVHRSCLDHWRSVKEGFAFSHCTTC
```

```
Bra033335-V   CRICHEEEEAESY------------------------------------------------------------------FEAPCSCSGTVK------------------FAHRDCIQRWCDEKG---NTICEIC
Bra036295-V   CRICHEEE-AESF------------------------------------------------------------------FEVPCACSGTVK------------------FAHRDCIQRWCNEKG---NTTCEIC
Bra037753-V   CRVCHSTESDKRGD-AALGLLEITPPVPEARKSNADEA----EAEQKSCIVKSN----------IDIELGIQQQHQGALIELGCSCKNELA------------------LVHYACALKWFLNHG---STVCEIC
Bra038662-V   CRVCHSVESDRRGD-TALGFLGITPPIPEARKSNADDVSKDTEAEQKSSIVKSNG------VNPIDIEMGIQQ-HQDALLELGCSCKNELA------------------LVHYACALKWFLNHG---STVCEIC
Bra040226-V   CRICL-DVLEEGNT-----------------------------------------------------------------LKMECSCKGDLR------------------LVHEACAIKWFSTKG---TRTCDVC
```

Figure S4 Multiple sequence alignment of 38 RING-C2 domains of *B. rapa*.

```
                *  *                         *  *      *  *                                    *  *
Bra000934-C2    CHQCKN-LTDK-------------VNLVFCSKCTKKRY-CYDCIKK--------------------WYPETTSEEVQAACPFC
Bra000936-C2    CHQCKN-LTDK-------------VDLVFCSKCIKKRY-CYDCIKR--------------------WYPERTPEEVRDACPFC
Bra004233-C2    CYICRYRQEQP----------------LDCVECGFVL--CFRCATLPHKLRYKHD-----EHLLVFSYKEYADDDELYWCEIC
Bra005845-C2    CQICSDNVGKTVD----------GDRFVACDVCGFPV--CRPCYEF--------------------ERK-----DGNQSCPQC
Bra006036-C2    CQICGDEIELSDD----------GESFVACNECAFPV--CRTCYEY--------------------ERR-----EGNQSCPQC
Bra006407-C2    CEICGDQIGLTVE----------GDLFVACNECGFPA--CRPCYEY--------------------ERR-----EGSQNCPQC
Bra006677-C2    CPLCAEEMDLT------------DQQLKPCK-CGYQI--CVWCWHH--------------------IVDMAEKDQIEGRCPAC
Bra008170-C2    CPLCAEKMDAT------------DLLFEQCASCEYKM--CLFCYNN--------------------INE------STRVCPGC
Bra009365-C2    CPHCQSRVEKDSDNY---------LRIVTCV-CSYAF--CWRCLR-----------------------PEEDHRGGLDYCFEV
Bra010131-C2    CPICYEDLDLT------------DSSFLPCP-CGFRL--CLFCHKT--------------------ICD------GDGRCPGC
Bra011345-C2    CQICGDHAGLTET----------GDLFVACNECAFPV--CRPCYEY--------------------ERK-----DGTQCCPHC
Bra011429-C2    CPVCYECDAQFTV----------FNRRHHCRLCGRVF--CAKCAAN---------SIPSPSDEAKDSHEESSSDRRIRVCNYC
Bra011865-C2    CQICGDDIELSVN----------GELFVACNECAFPV--CRPCYEY--------------------ERR-----EGNQACPQC
Bra011940-C2    CPLCAEEMDLT------------DQHFKPCK-CGYQI--CVWCWHR--------------------IIEMAEKDKTEGRCPAC
Bra012578-C2    CNTCGEEIGVKPN----------GEFFVACHECSFPI--CKACLEY--------------------EFK-----EGRRICLRC
Bra013525-C2    CHWCGT--RGS-------------EDLISCLSCEKEFF-CVDCIEK--------------------RNKGS-KEEVEKKCPVC
Bra015887-C2    CPICSEGMDAT------------DLSFLPCP-CGFRL--CLFCHKQ--------------------INE------NDGRCPAC
Bra016801-C2    CHQCFK--GER-------------RFLFICTFCEEKLY-CFPCIKK--------------------WYPHLSHDDVIEKCPFC
Bra018086-C2    CPICYEDLDLT------------DSNFLPCP-CGFRL--CLFCHKT--------------------ICD------GDGRCPGC
Bra020455b-C2   CPKCYHMVGRSYG-----------CNRITCR-CGNAF--CYKCGY-------------------------LWNRGIHGDCNQD
Bra022865b-C2   CIVAIDFASGSRS----------EHYDVSCL-CSYEF--CWNCGEDAHRPVDCDTVSKWISKNTDESENTNWILANTKPCPNC
Bra023952-C2    CQICGDNAGLTET----------GDLFVACNECAFPV--CRPCYEY--------------------DRK-----DGTQCCPQC
Bra024324-C2    CKICRDEIELTVD----------GEPFVACNECAFPV--CRPCYEY--------------------ERR-----EGNQACPQC
Bra027055-C2    CHQCLK--GER-------------ITLLVCSECEETMY-CLQCIRK--------------------WYPHLSEDDIVDKCPFC
Bra028768-C2    CQICSDNVGKTVD----------GDRFVACDICGFPV--CRPCYEY--------------------ERK-----HGNQSCPQC
Bra029238-C2    CPICYEDLDST------------DASFFPCP-CGFKL--CLFCHKT--------------------IYD------GDGRCPGC
Bra029664-C2    CHQCQR--NDN-------------GEVVRCQNCCDRKRYCHKCLET--------------------WYPRIPHEDIAKKCPFC
Bra029874-C2    CQICTENVGRAEN----------GYPFVACDVCSFSV--CRPCYEY--------------------ERR-----YGNQSCPQC
Bra030754-C2    CHHCLR--KDR-------------ERIISCLKCNQRAF-CDACITT--------------------QYSDIPLEEVEKVCPAC
Bra031904-C2    CEICRDEIESTVD----------GEPFVACNECAFPV--CRPCYEY--------------------ERR-----EGNQACPQC
Bra033714-C2    CRICRDEVKDGDN----------GQTFVACHVCAFPV--CKPCYEY--------------------ERS-----NGNKCCPQC
Bra035692-C2    CPLCAEEMDLT------------DQHLNPCQ-CGYQI--CVWCWHQ--------------------IIEMAEKDKTEGRCPAC
Bra037394-C2    CHQCKIMMSSK-------------TDLVFCSICVNKRY-CKDCIKR--------------------WYPERTPEGVKAACPFC
Bra037793-C2    CEICRDEVELTVD----------GEPFVACNECAFPV--CRPCYEY--------------------ERR-----EGNQACPQC
Bra038282-C2    CPLCAEEMDLT------------DQQLNPCK-CGYQI--CVWCWHH--------------------IIDMAEKDQSEGRCPAC
Bra038285b-C2   CIRCNNMVELAFG-----------CYHITCR-CGYEF--CYTCGAEWK------------------------NKKATCACPIW
Bra038775-C2    CHWCGT--RGF-------------EDLISCLSCGREFF-CVDCIEK--------------------RNKGS-KEEVGKKCPVC
Bra040045-C2    CKTCGKKYHKNCVKSWAQHRDLFHWSSWSCPSCRV----CEVCRRT--------------------GDP-----NKFVFCKRC
```

Figure S5 Sequence alignment of 731 RING domains in 715 putative proteins of *B. rapa*.

```
              *  *                                                                             *   *       *    *                                                                *    *
Bra000007-H2  CTVC------------------------------------------LSVFGDG------EEIRR--LSA------------CK--HAF-----HVS--C---------------IET--------WL---KDH-------PN---------------------CPI--C 42
Bra000143-H2  CAVC------------------------------------------KENFELK------SAARE--MP-------------CN--HIY-----HPD--C---------------ILP--------WL---AIR-------NS---------------------CPV--C 41
Bra000207-H2  CAVC------------------------------------------KDEFELG------TEAKQ--MP-------------CN--HIY-----HSD--C---------------IVP--------WL---VQH-------NS---------------------CPV--C 41
Bra000350-H2  CAIC------------------------------------------REDFVIG------ESARR--LP-------------CD--HLY-----HHD--C---------------ITP--------WL---TSH-------NT---------------------CPL--C 41
Bra000673-H2  CAIC------------------------------------------LNEFDDE------ETLRW--MPP------------CS--HTF-----HAS--C---------------IDV--------WL---SSW-------ST---------------------CPV--C 42
Bra000683-H2  CAIC------------------------------------------IEDYRIG------DKLRI--LP-------------CN--HKF-----HVG--C---------------VDL--------WL---GQR-------RSF--------------------CPV--C 42
Bra000742-H2  CTVC------------------------------------------LSDFESD------DNIRQ--LPN------------CR--HVF-----HDH--C-----------------------LDRWIV--DCR-------KMT--------------------CPI--C 44
Bra000951-H2  CSIC---------------------------------------YEDLK-PVAEN--------LQ--SISA-----------CG--HVF-----HEL--C---------------LQ--------QWFE--YCP-------STNKRN-----------------CPI--C 48
Bra001080-H2  CSVC------------------------------------------LSEFQEE------ERLRM--LPK------------CS--HAF-----HVA--C---------------IDT--------WL---KSH-------SN---------------------CPL--C 42
Bra001153-H2  CSIC------------------------------------------LDTVAKDGD----RAWAN--LQ-------------CG--HQF-----HLD--C-------------------------------IG--------SAFNAKGVMQ-------------CPN--C 45
Bra001381-H2  CPVC------------------------------------------QDRFEVG------SVARK--LP-------------CK--HIY-----HSE--C---------------IIP--------WL---IQR-------NT---------------------CPV--C 41
Bra001390-H2  CCIC------------------------------------------LGGLEEG------EKMKV--LPM------------CR--HCY-----HCE--C---------------VDR--------WL---MTE-------SS---------------------CPL--C 42
Bra001582-H2  CTIC------------------------------------------QETYKNR------DKIAM--LD-------------CK--HEY-----HPA--C---------------LE--------KWLV--IKN-------I----------------------CPI--C 41
Bra001639-H2  CAVC------------------------------------------LSEFEET------ESGRV--LPG------------CK--HTF-----HVD--C---------------IDM--------WF---HSH-------ST---------------------CPL--C 42
Bra001721-H2  CVIC------------------------------------------LSDFVAG------EQIRL--LPK------------CN--HGF-----HVR--C---------------IDK--------WL---TQQ-------MT---------------------CPK--C 42
Bra001767-H2  CAVC------------------------------------------MDEFEDG------GDVKQ--MP-------------CK--HVF-----HQD--C---------------LMP--------WL---ELH-------NS---------------------CPV--C 41
Bra002061-H2  CAIC------------------------------------------LTEFADG------EEIRV--LPL------------CG--HSF-----HVA--C---------------IDK--------WL---VSR-------SS---------------------CPS--C 42
Bra002084-H2  CSVC------------------------------------------LQDFQLG------ETVRS--LPH------------CH--HMF-----HLP--C---------------IDK--------WL---LRH-------GS---------------------CPM--C 42
Bra002147-H2  CGVC-------------------------KKLLSQKSPWCSHKILRSGDMPAAGV----FP--------------------CH--HVY-----HVE--C---------------------------LDKVTPT------SQTRDPS-----------------CPA--C 59
Bra002176-H2  CPIC------------------------------------------YE-YLFDSL-------KD--TTVMK----------CG--HTM-----HFE--C---------------------------YHEMLK-------RDKFC-------------------CPI--C 43
Bra002312-H2  CAIC------------------------RNHIMDLCIECQANQASATSEECTVAW-------GV-----------------CN--HAF-----HFH--C---------------ISR--------WL---KTR-------QV---------------------CPL--D 56
Bra002336-H2  CAVC------------------------------------------LGDLEDG------DEVRE--LRN------------CS--HVF-----HRE--C---------------IDRWLDYECCGGDD-NDGEED---NHRT---------------------CPL--C 56
Bra002382-H2  CSIC------------------------------------------LEPFCDSDP-------ST--LTS------------CK--HEF-----HLQ--C---------------IL--------EWC---QRS-------SQ---------------------CPM--C 41
Bra002441-H2  CPVC------------------------------------------FE-YLFDST-------RD--ITVLR----------CG--HAM-----HLE--C--------------TKDM--------GL---HNRYT----------------------------CPL--C 43
Bra002456-H2  CAIC------------------------------------------LTAMKAGQ------GHAI--FTAE-----------CS--HSF-----HFH--C---------------ITTN----------VQHGN-------QF---------------------CPV--C 44
Bra002559-H2  CAVC------------------------------------------TEVFEAE------TEARE--MP-------------CK--HIF-----HDD--C---------------ITP--------WL---SIR-------NS---------------------CPV--C 41
Bra002636-H2  CVIC------------------------------------------LGLWEVG------DFGRK--LRF------------CG--HGF-----HVE--C---------------IDM--------WL---SSH-------SS---------------------CPL--C 42
Bra002690-H2  CDLC---------------------------------------GRDLASDPERP-------NVS--LRSLQEVCVLD----CG--HVY-----HFK--C---------------LKG-------TTLD-LDNRS---TNPS----------------------CIF--C 58
Bra002823-H2  CSVC------------------------------------------LDEFEKGVE----EEAKE--MP-------------CK--HKF-----HVK--C---------------IVP--------WL---ELH-------SS---------------------CPV--C 43
Bra003083-H2  CAIC------------------------------------------LSEYEPN------ETLRT--IPL------------CQ--HCF-----HAD--C---------------IDE--------WL---KLN-------GT---------------------CPV--C 42
Bra003351-H2  CTVC------------------------------------------LEDFEPK------ETVMV--TP-------------CK--HMF-----HEE--C---------------IVP--------WL---KSK-------GQ---------------------CPL--C 41
Bra003404-H2  CAVC------------------------------------------LSRFEPD------DQLRL--LPL------------CC--HAF-----HAD--C---------------IDI--------WL---VSN-------QT---------------------CPL--C 42
Bra003450-H2  CAVC------------------------------------------LYEFEGE------EEIRW--LRN------------CR--HIF-----HRS--C---------------LD--------RWMD--HDQ-------KT---------------------CPL--C 43
Bra003507-H2  CSVC------------------------------------------LSEFEEE------DEGRV--LPK------------CG--HVF-----HVD--C---------------IDT--------WF---RSR-------SS---------------------CPL--C 42
Bra003667-H2  CSLC------------------------------------------EKEYPKE------GEIVR--KTR------------CN--HIF-----HGT--C----------------IS-------RYL----LR-------IPH--------------------CPF--C 42
Bra003720-H2  CVIC------------------------------------------LTEFAAG------DELRV--LPQ------------CG--HGF-----HVS--C---------------IDT--------WL---ESH-------SS---------------------CPS--C 42
Bra003754-H2  CGAC--------------------------------SKFEKSSVANFERQIAA---------VL--A--------------CG--HVY-----HAE--C---------------------------LETMTTE------IEKYDLA-----------------CHV--C 51
Bra003830-H2  CSIC------------------------------------------QEEYEGK------DEVGK--LR-------------CG--HRY-----HIH--C---------------AK--------QWLL--RKN-------S----------------------CPV--C 41
Bra003887-H2  CPVC------------------------------------------LNEFEDD------ETLRL--LPK------------CC--HVF-----HPG--C---------------VDA--------WL---RSH-------AT---------------------CPL--C 42
Bra004008-H2  CPTC----------------------------------------FYEYAEDNPKT----VLQ-------------------CG--HIF-----HLA--C---------------IY--------EWM---ERS-------AA---------------------CPF--C 41
Bra004280-H2  CCIC------------------------------------------LSSYEDG------TELVT--LP-------------CN--HHF-----HST--C---------------IEK--------WL---KMN-------AT---------------------CPL--C 41
Bra004287-H2  CAIC------------------------------------------KEEFEVG------EEGKE--LK-------------CL--HLY-----HTS--C---------------IVS--------WL---NIH-------NT---------------------CPI--C 41
Bra004342-H2  CCIC------------------------------------------QEGFFLGGY-------AT--ITS------------CS--HVF-----HSS--C---------------ITD--------WI---NKS-------TK---------------------CPL--C 41
Bra004436-H2  CSIC------------------------------------------LELVVDDGS----RSSAK--LQ-------------CG--HQF-----HLD--C-------------------------------IG--------SAFNMKGTMQ-------------CPN--C 45
Bra004446-H2  CSVC------------------------------------------LSEFEEE------DQGRL--LPK------------CG--HAF-----HVD--C---------------IDT--------WF---RSS-------ST---------------------CPL--C 42
Bra004563-H2  CSIC------------------------------------------LCEYREA------EMLRM--MPE------------CK--HYF-----HLC--C---------------LDA--------WL---KLN-------GS---------------------CPV--C 42
Bra004589-H2  CPVC------------------------------------------KEEFELG------AEAKQ--MP-------------CN--HVY-----HSD--C---------------IVP--------WL---VQH-------NS---------------------CPV--C 41
Bra004685-H2  CAVC------------------------------------------LTLLEEK------DTARM--LPN------------CK--HVF-----HMT--C---------------VDT--------WL---TTH-------ST---------------------CPI--C 42
Bra004686-H2  CAVC------------------------------------------LAFLEEK------DTARM--LLN------------CK--HVF-----HMA--C---------------VDT--------WL---ATH-------ST---------------------CPI--C 42
Bra004687-H2  CAVC------------------------------------------LSLLEEQ------DTARE--LPS------------CK--HVF-----HVD--C---------------VDK--------WL---TAC-------ST---------------------CPV--C 42
Bra004847-H2  CSIC------------------------------------------LQDAAEG------EKMRR--LTA------------CS--HCF-----HAG--C---------------IDP--------WL---EKR-------ST---------------------CPL--C 42
Bra004935-H2  CCIC------------------------------------------LCDYEDG------TELRE--LS-------------CR--HHF-----HEA--C---------------IDK--------WL---RIN-------AT---------------------CPL--C 41
Bra005020-H2  CAVC------------------------------------------KETFILD------SAARE--MP-------------CN--HIY-----HPD--C---------------ILP--------WL---AIR-------NS---------------------CPV--C 41
Bra005066-H2  CSIC------------------------------------------LTKMKEGG------GHAI--FTAE-----------CS--HSF-----HFH--C---------------IASN----------VKHGN-------QV---------------------CPV--C 44
```

```
Bra005307-H2  CPIC------------------------------------------LGDYKGN------DLLRQ--LPD------------CN--HFF-----HLK--C---------------VDM--------WL---RIN-------PT---------------------CPV--C 42
Bra005343-H2  CAIC------------------------------------------LSEFSDE------DTVRL--ITV------------CR--HGF-----HSI--C---------------IDL--------WF---ESH-------KT---------------------CPV--C 42
Bra005374-H2  CAVC------------------------------------------LCEFEDE------ETLRL--MPP------------CC--HVF-----HAD--C---------------VDV--------WL---SER-------ST---------------------CPL--C 42
Bra005375-H2  CVVC------------------------------------------LCEFQDN------DRLSL--MPN------------CC--HVY-----HAD--C---------------VSV--------WL---SDH-------ST---------------------CPL--C 42
Bra005859-H2  CSIC---------------------------------------FEDLS-DVDEE--------SI--ELHD-----------CS--HVF-----HKV--C---------------LF--------QWIW--SKS-------S----------------------CPL--C 43
Bra005970-H2  CAVC------------------------------------------KDEMGIG------SRGVVLQLP-------------CN--HKY-----HGE--C---------------IVP--------WL---ETR-------NT---------------------CPV--C 43
Bra006070-H2  CSIC------------------------------------------QEEYVDG------DEVGT--MP-------------CE--HMY-----HVS--C---------------VQ--------QWLR--MKN-------W----------------------CPI--C 41
Bra006321-H2  CPTC----------------------------------------LEEYTSENPKI----VTK-------------------CC--HHY-----HLS--C---------------IY--------EWM---ERS-------QD---------------------CPV--C 41
Bra006415-H2  CSVC------------------------------------------LSEFEED------ESLRL--LPK------------CN--HAF-----HLP--C---------------IDT--------WL---RSH-------SN---------------------CPL--C 42
Bra006470-H2  CPIC------------------------------------------FE-YLFDSL-------KE--TTVMK----------CG--HTM-----HCE--C---------------------------YHEMIK-------RDKFC-------------------CPI--C 43
Bra006565-H2  CAIC------------------------RNHIMDLCIECQANQASATSEECTVAW-------GV-----------------CN--HAF-----HFH--C---------------ISR--------WL---KTR-------QV---------------------CPL--D 56
Bra006583-H2  CAVC------------------------------------------LGDLEDG------DEVRE--LRN------------CS--HVF-----HRE--C--------------IDRWLDYEC---DD--NDGEED---NHRT---------------------CPL--C 53
Bra006585-H2  CCIR------------------------------------------KENLVIG------DKMQE--LP-------------CK--HTF-----HPP--C---------------LKP--------WL---DEH-------NS---------------------CPI--C 41
Bra006603-H2  CSIC------------------------------------------LEAFCESEP-------ST--LTS------------CK--HEY-----HLQ--C---------------IL--------EWC---QRS-------SQ---------------------CPM--C 41
Bra006641-H2  CPVC------------------------------------------FE-YLFDST-------RD--ITVLR----------CG--HTM-----HLQ--C--------------TKDM--------GL---HNRYT----------------------------CPV--C 43
Bra006709-H2  CAVC------------------------------------------TEVFEGE------TEARE--MP-------------CK--HIF-----HED--C---------------IVP--------WL---SIR-------NS---------------------CPV--C 41
Bra006752-H2  CVIC------------------------------------------LGLWEVG------EFGRK--LRT------------CG--HGF-----HVE--C---------------IDM--------WL---SSH-------ST---------------------CPL--C 42
Bra006916-H2  CSIC------------------------------------------LMDYEDE------DAVTQ--LPR------------CN--HLF-----HVH--C---------------IEP--------WL---LR--------GSLT-------------------CPL--C 43
Bra007174-H2  CSVC------------------------------------------LEQVTVG------EVVRT--LP-------------CL--HQF-----HAV--C---------------IDP--------WL---RQQ-------GT---------------------CPV--C 41
Bra007253-H2  CPVC------------------------------------------KEEFELK------SEAKQ--MP-------------CK--HMY-----HSD--C---------------IVP--------WL---VQH-------NT---------------------CPV--C 41
Bra007414-H2  CSVC------------------------------------------LEDFEPK------ETVML--TP-------------CK--HMF-----HEE--C---------------IVP--------WL---KSK-------GQ---------------------CPV--C 41
Bra007504-H2  CAVC------------------------------------------KEDFSVG------ESAKR--LP-------------CS--HIY-----HSD--C---------------IVP--------WL---SDH-------NS---------------------CPL--C 41
Bra007515-H2  CAVC------------------------------------------LSKFEPE------DQLRL--LPL------------CC--HAF-----HAD--C---------------IDT--------WL---VSN-------QT---------------------CPL--C 42
Bra007580-H2  CSIC------------------------------------------LCAYEDG------AELRE--LP-------------CR--HHF-----HSV--C---------------VDK--------WL---RIN-------AT---------------------CPL--C 41
Bra007606-H2  CAVC------------------------------------------LYEFEGE------QEIRW--LRN------------CR--HIF-----HRS--C---------------LD--------RWMD--HDQ-------KT---------------------CPL--C 43
Bra007613-H2  CSIC------------------------------------------LCEYMEE------EMVRM--MPE------------CK--HYF-----HVS--C---------------LDA--------WL---KLN-------GS---------------------CPV--C 42
Bra007629-H2  CSIC------------------------------------------LSEYATK------ETVRF--LPE------------CE--HCF-----HAE--C---------------IDA--------WL---KLH-------SS---------------------CPV--C 42
Bra007694-H2  CSVC------------------------------------------LSEFEEE------DEGRV--LPK------------CG--HVF-----HVD--C---------------IDT--------WF---RSR-------SS---------------------CPL--C 42
Bra007789-H2  CAIC------------------------------------------LEGLYKNDE-------TF--DLPY-----------CS--HRF-----HST--C-------------------------------VG--------EWLQSHNS---------------CPL--C 42
Bra007847-H2  CAIC------------------------------------------LDDFESK-------TMSM--FPG------------CF--HQF-----HRE--C---------------LL--------EWI---ERS-------RRGHGS-----------------CPL--C 45
Bra007848-H2  CAIC------------------------------------------LEDMSES------GQAFC--QPPG-----------CV--HVF-----HED--C---------------FVK--------WL---GRH-------DS---------------------CPL--C 43
Bra008015-H2  CPIC------------------------------------------LNEFEDE------ETLRL--IPK------------CC--HVF-----HPD--C---------------IDA--------WF---QSH-------AT---------------------CPL--C 42
Bra008016-H2  CPVC------------------------------------------LNEFEDA------ERLRL--IPK------------CC--HVF-----HPG--C---------------IDA--------WL---QSH-------PT---------------------CPL--C 42
Bra008018-H2  CPVC------------------------------------------LNEFEED------ESLRL--LPK------------CN--HAF-----HIS--C---------------IDT--------WL---RSH-------TN---------------------CPL--C 42
Bra008028-H2  CAIC------------------------------------------LSDLVKG------DKARL--LPK------------CN--HSF-----HVE--C---------------IDM--------WF---QSH-------ST---------------------CPI--C 42
Bra008075-H2  CVVC------------------------------------------LSELAEG------DKAKL--LPS------------CK--HWF-----HAH--C---------------IDA--------WL---ESH-------AT---------------------CPI--C 42
Bra008196-H2  SSTC------------------------SNDLATGSHYASSEGDWSLNTFSELGG-VFSKGQMMSSVAAVLA---------CG--HVY-----HAE--C---------------------------LETVTTE------IEKYDPA-----------------CPI--C 74
Bra008257-H2  CAIC------------------------------------------LTEFAAG------DELRV--LPQ------------CG--HGF-----HVS--C---------------IDT--------WL---GSH-------SS---------------------CPS--C 42
Bra008462-H2  CPVC------------------------------------------LERLDQ-----DTGGILT-TM--------------CN--HSF-----HCS--C---------------ISN--------WP---DSS------------------------------CPV--C 40
Bra008475-H2  CCIC------------------------------------------LARYGDD------EEVRE--LP-------------CL--HVF-----HVD--C---------------VDK--------WL---KIN-------AT---------------------CPL--C 41
Bra008518-H2  CRIC------------------------------------------QDEFEGG------EQVRS--LRN------------CA--HVF-----HKT--C-----------------------IDRWIH--DDK--------MT--------------------CPL--C 43
Bra008671-H2  CAIC------------------------------------------MGDFVVK------EKVTR--LP-------------CW--HYY-----HGE--C---------------IVP--------WL---EMK-------NT---------------------CPV--C 41
Bra008675-H2  CPTC----------------------------------------LEEYTSENPKI----VTK-------------------CS--HHF-----HLG--C---------------IY--------EWM---ERS-------ED---------------------CPV--C 41
Bra009015-H2  CSIC------------------------------------------QEEYVDG------DEVGT--MP-------------CE--HMY-----HVS--C---------------VQ--------QWLR--MKN-------W----------------------CPI--C 41
Bra009037-H2  CSVC------------------------------------------LSEFEEG------EGVRL--LPK------------CS--HAF-----HVS--C---------------IDT--------WL---LSH-------KN---------------------CPL--C 42
Bra009136-H2  CAVC------------------------------------------LARFEPT------EVLRL--LPK------------CK--HAF-----HVE--C---------------VDT--------WL---DAH-------ST---------------------CPL--C 42
Bra009297-H2  CSIC------------------------------------------LCDYVAR------DPVRC--IPE------------CN--HCF-----HAD--C------------------------------VDEWLR---TSAT---------------------CPL--C 42
Bra009323-H2  CAVC------------------------------------------KDEMDVG------TEAVG--LP-------------CD--HKY-----HTE--C---------------IVP--------WL---KTR-------NT---------------------CPV--C 41
Bra009570-H2  CVIC------------------------------------------KEEMSEG------RDVCE--MP-------------CQ--HLF-----HWK--C---------------ILP--------WL---SKR-------NT---------------------CPF--C 41
Bra009663-H2  CSVC------------------------------------------LQDFQVG------ETVRS--LPH------------CH--HMF-----HLP--C---------------IDK--------WL---RGH-------AS---------------------CPL--C 42
Bra009800-H2  CSIC------------------------------------------QEEYVDG------DEVGT--LP-------------CQ--HMY-----HVS--C---------------AQ--------QWLR--MKN-------W----------------------CPI--C 41
Bra009912-H2  CPDC--------------------------------------------------------NAME-----------------CN--HSF-----HLH--C---------------ILK--------WV---NSQ-------TSQAH------------------CPM--C 34
Bra009960-H2  CAIC------------------------------------------LNEFQDD------ETLRL--LPK------------CD--HVF-----HPH--C---------------IGA--------WL---QGH-------VT---------------------CPV--C 42
Bra009962-H2  CAIC------------------------------------------LTEFGDD------ETLRF--LPK------------CD--HVF-----HAH--C---------------IGA--------WL---QGH-------VT---------------------CPV--C 42
Bra010204-H2  CSVC------------------------------------------LGDYQAD------DKLQQ--ILS------------GG--HTF-----HMD--C---------------IDL--------WL---TSH-------ST---------------------CPL--C 42
Bra010289-H2  CVVC------------------------------------------IEGFRQG------QWCRK--LPG------------CG--HVF-----HRK--C---------------VDF--------WL---VKV-------GT---------------------CPI--C 42
Bra010349-H2  CSVC------------------------------------------LSKFESV------EILRL--LPK------------CR--HAF-----HIG--C---------------IDQ--------WL---EQH-------AT---------------------CPL--C 42
Bra010370-H2  CVIC---------------------------------------MTAINLRQRTSD-------FM--VTP------------CE--HFF-----HTG--C---------------LQ--------RWMD--IKM-------E----------------------CPT--C 44
Bra010473-H2  CAIC------------------------------------------REPM-KA------KRLH------------------CN--HLF-----HLG--C---------------LRS--------WL---DQGLN---DVYS---------------------CPT--C 41
Bra010532-H2  CAIC------------------------------------------LMEFSDG------EEIRI--LPL------------CR--HAF-----HVA--C---------------IDK--------WL---TSR-------SS---------------------CPS--C 42
Bra010641-H2  CAIC------------------------------------------LNELEDR------ETVRL--LPV------------CN--HLF-----HVD--C---------------IDA--------WL---YSH-------AT---------------------CPV--C 42
Bra010702-H2  CGAC------------------------SRPLSEKSLWSSQKMFMTNELSVSA---------IL--A--------------CG--HVY-----HGE--C---------------------------LEKMTPE------IDKFDPS-----------------CPI--C 59
Bra010752-H2  CSIC------------------------------------------LESFTPQDP-------AT--VTS------------CK--HEY-----HLQ--C---------------II--------EWS---QRS-------KE---------------------CPI--C 41
Bra010969-H2  CCVC------------------------------------------LCGFKEE------EEVSE--LVS------------CK--HFF-----HRA--C---------------LD--------NWFG--NNH-------TT---------------------CPL--C 43
Bra011068-H2  CSVC------------------------------------------LSKFESV------EILRL--LPK------------CR--HAF-----HVG--C---------------IDQ--------WL---EQH-------AT---------------------CPL--C 42
Bra011187-H2  CVVC------------------------------------------IDGFRQG------QWCRR--LPG------------CG--HVF-----HRK--C---------------VDF--------WL---VKV-------GT---------------------CPI--C 42
Bra011189-H2  CAVC------------------------------------------LSEFETE------DKLRL--LPK------------CS--HAF-----HMD--C---------------IDT--------WL---LSH-------ST---------------------CPL--C 42
Bra011215-H2  CLVC------------------------------------------LDYIEMN------TTGKQ--MP-------------CQ--HKF-----HSY--C---------------LLY--------WL---QLH-------SS---------------------CPV--C 41
Bra011359-H2  CCIC------------------------------------------LAKYANN------EELRE--LP-------------CS--HFF-----HKE--C---------------VDK--------WL---KIN-------AS---------------------CPL--C 41
Bra011618-H2  CAIC------------------------------------------LTEFTDG------EEIRI--LPL------------CN--HAF-----HLA--C---------------IDK--------WL---TSR-------SS---------------------CPS--C 42
Bra011643-H2  CSVC------------------------------------------LQDFQLG------ETVRS--LPQ------------CH--HMF-----HLP--C---------------IDN--------WL---LRH-------GS---------------------CPM--C 42
Bra011873-H2  CGAC------------------------SRPLSEKSMWSSQKIFMTNELSVSA---------IL--A--------------CG--HVY-----HGE--C---------------------------LEQMTPE------IDKFDPS-----------------CPI--C 59
Bra011991-H2  CIIC------------------------------------------LSKFEEG------ETVKV--IPH------------CG--HLF-----HVD--C---------------VDT--------WL---NSH-------VT---------------------CPL--C 42
Bra012055-H2  CAIC------------------------------------------LEDYRFG------DSLRL--LP-------------CQ--HAF-----HLS--C---------------IDS--------WL---TKW-------GTS--------------------CPV--C 42
Bra012134-H2  CVIC------------------------------------------LDELKCNDE-------AS--TLA------------CG--HDF-----HYE--C---------------IK--------NWLM--VKN-------K----------------------CPL--C 41
Bra012251-H2  CAIC------------------------------------------LTEFSNG------DELRV--LPQ------------CG--HGF-----HVS--C---------------IDT--------WL---GSH-------SS---------------------CPS--C 42
Bra012330-H2  CPVC------------------------------------------LNGFEDD------ETLRL--IPQ------------CC--HVF-----HTC--C---------------IDA--------WL---RSH-------VT---------------------CPL--C 42
Bra012441-H2  CVIC------------------------------------------LEEWKAG------ETVKE--MP-------------CK--HRF-----HGG--C---------------VEK--------WL---GLH-------GS---------------------CPV--C 41
Bra012630-H2  CAIC-----------------------------------------LLEFDEDHV------L-RL--LTT------------CY--HVF-----HQE--C---------------IDL--------WF---ESH-------KT---------------------CPV--C 42
Bra012814-H2  CSIC------------------------------------------LVDYEAE------DAVTH--LPR------------CN--HLF-----HIN--C---------------IEP--------WL---LS--------GHLT-------------------CPL--C 43
Bra013006-H2  CAVC------------------------------------------REEMSVG------NEVAE--LP-------------CR--HKY-----HGE--C---------------IVP--------WL---GIR-------NT---------------------CPV--C 41
Bra013110-H2  CTIC------------------------------------------LEPLVNTRD----DRRTFVTLR-------------CG--HKF-----HLD--C-------------------------------IG--------SAFNAKGFMQ-------------CPN--C 47
Bra013118-H2  CCQC----------------------------------------QAAFDESG-------GHQLT-RLG-------------CL--HAI-----HTS--C----------------LV-------SLIK--SLP-------PHTAPPGYV--------------CPT--C 51
Bra013211-H2  CSVC------------------------------------------LTKFEE-------GSEVN-KLK-------------CG--HLF-----HKT--C-----------------------VEKWID--YWN--------IT--------------------CPL--C 42
Bra013267-H2  CSIC------------------------------------------LGEFNEE------ESLRL--LPN------------CN--HIF-----HVV--C---------------IDR--------WL---TSH-------SN---------------------CPL--C 42
Bra013268-H2  CAIC-----------------------------------------LLEFDGDHV------L-RL--LTT------------CY--HVF-----HQE--C---------------IDL--------WF---ESH-------KT---------------------CPV--C 42
Bra013751-H2  CCVC------------------------------------------LGEFELK------EELVE--MPS------------CK--HIF-----HLD--C---------------IHL--------WL---YSH-------TT---------------------CPL--C 42
Bra013876-H2  CAIC------------------------------------------REPMAKA------KKLH------------------CN--HLF-----HLG--C---------------LRS--------WL---DQGLN---EVYS---------------------CPT--C 42
Bra014043-H2  CCVC------------------------------------------QEEYKEG------EEMGV--LE-------------CG--HDF-----HSQ--C---------------IK--------EWLK--RKN-------L----------------------CPI--C 41
Bra014174-H2  CVIC------------------------------------------LSDFVCG------EKLRL--LPK------------CN--HGF-----HVR--C---------------IDK--------WL---KQH-------LT---------------------CPK--C 42
Bra014175-H2  CVIC------------------------------------------LSDFVCG------EKLRL--LPK------------CN--HGF-----HVR--C---------------IDK--------WL---QQH-------LT---------------------CPK--C 42
Bra014177-H2  CVIC------------------------------------------LSEFVSE------ERVKL--LPT------------CH--HGF-----HVR--C---------------IDK--------WL---SSH-------SS---------------------CPT--C 42
Bra014221-H2  CSIC------------------------------------------LESFTNG------DMLIS--LP-------------CT--HSF-----HSS--C---------------LN--------PWLI--ACG-------D----------------------CPY--C 41
Bra014266-H2  CAVC------------------------------------------KEVFSLG------NETTQ--LP-------------CL--HLY-----HPH--C---------------IVP--------WL---GAR-------NS---------------------CPL--C 41
Bra014339-H2  CIVC------------------------------------------LSTLKSG------EEVRK--LG-------------CR--HVF-----HKQ--C---------------LE--------GWL---QHL-------NFK--------------------CPL--C 42
Bra014424-H2  CSIC------------------------------------------LSEYASS------ETVGC--LLI------------CE--HCF-----HVE--C---------------IDT--------WL---QLR-------SS---------------------CPI--C 42
Bra014435-H2  CAVC------------------------------------------LYEFEGD------QEIRR--LRN------------CR--HIF-----HRS--C---------------LD--------RWMD--HDQ-------KT---------------------CPL--C 43
Bra014499-H2  CPLC-----------------------------------LYPLFPE-DGESDQMP-----FMKL--MS-------------CF--HCF-----HCE--C---------------IIRWW-----NWL---HTE-------KEADSVNGNSGSVDKSLGN----CPV--C 68
Bra014508-H2  CAVC------------------------------------------LQEAEEG------DKMRR--LTI------------CR--HCF-----HAD--C---------------IDP--------WL---GEM-------SST--------------------CPL--C 43
Bra014684-H2  CPVC------------------------------------------KEEFELK------SEAKQ--MP-------------CK--HVY-----HSD--C---------------IVP--------WL---VQH-------NT---------------------CPV--C 41
Bra015142-H2  CSVC------------------------------------------LSNFEE-------DSEIN-KLK-------------CG--HLF-----HKT--C-----------------------LEKWID--YWN--------IT--------------------CPL--C 42
Bra015160-H2  CTAC------------------------------------------TFTLDIP--------AVH--FM-------------CM--HSF-----HQR--C---------------------------LG--DNE-------TE---------------------CPE--C 36
Bra015221-H2  CCIC------------------------------------------QEEYAEG------DDLGT--LE-------------CG--HEF-----HKD--C---------------IK--------QWVM--LKN-------L----------------------CPI--C 41
Bra015225-H2  CAIC------------------------------------------LDQFKTG------ETLVH--LP-------------CT--HKF-----HSI--C---------------LL--------PWLD--TNA-------Y----------------------CPY--C 41
Bra015233-H2  CSIC---------------------------------------IDDLS-KTREN--------II--ELPQ-----------CL--HVF-----HQD--C---------------LF--------EWLR--RQN-------S----------------------CPL--C 43
Bra015308-H2  CSVC------------------------------------------LNEFQED------EKLRI--IPN------------CC--HVF-----HID--C---------------IDI--------WL---QSN-------AN---------------------CPL--C 42
Bra015349-H2  CVIC------------------------------------------LETPTIG------DTIRH--LP-------------CF--HKF-----HKD--C---------------IDP--------WL---GRS-------KA---------------------CPV--C 41
Bra015751-H2  CAIC------------------------------------------LTEFAAG------DELRV--LPQ------------CG--HGF-----HVA--C---------------IDT--------WL---GSH-------SS---------------------CPS--C 42
Bra015846-H2  CGAC------------------------------SKLLTERSSVANFELPIAA---------VL--A--------------CG--HVY-----HAE--C---------------------------LETMTTE------IEKYDPA-----------------CPI--C 53
Bra015894-H2  CPIC--------------------------------------HEYIFTSSSPV---------KA--LP-------------CG--HLM-----HSK--C-----------------------FKDYT---CS--------HYT--------------------CPV--C 42
Bra015913-H2  CTIC------------------------------------------LQDIAVG------EITRG--LPR------------CD--HTF-----HLV--C---------------VDK--------WL---IRH-------GS---------------------CPI--C 42
Bra015967-H2  CSIC------------------------------------------QEDYEAK------DEVGK--LR-------------CG--HRY-----HIS--C---------------VK--------QWLL--RKN-------C----------------------CPV--C 41
Bra016067-H2  CAIC------------------------------------------LSALVKG------DKARL--LPK------------CS--HSF-----HVE--C---------------IDM--------WF---QSH-------ST---------------------CPI--C 42
Bra016079-H2  CPVC------------------------------------------LNEFQED------ESLRL--LPK------------CN--HAF-----HIS--C---------------IDT--------WL---RSH-------TN---------------------CPL--C 42
Bra016093-H2  CAIC------------------------------------------LEDYSVG------DKLRL--LP-------------CR--HKF-----HAV--C---------------VD--------SWL---TSW-------RTF--------------------CPV--C 42
Bra016327-H2  CAVC------------------------------------------LCEFSED------DKLRL--LPV------------CS--HAF-----HID--C---------------IDT------FEFFAAGDDGER-----EPAENDIR---------------CGK--R 55
Bra016449-H2  CAIC------------------------------------------LTEFTAG------DELRV--LPQ------------CG--HGF-----HLS--C---------------IDT--------WL---GSH-------SS---------------------CPS--C 42
Bra016574-H2  CSIC------------------------------------------QDEYERE------CQVGK--LE-------------CG--HSF-----HVQ--C---------------VK--------QWLS--RKN-------A----------------------CPV--C 41
Bra016759-H2  CCIC------------------------------------------LSAYEDG------TELRE--LP-------------CG--HHF-----HCS--C---------------VDK--------WL---YIN-------AT---------------------CPL--C 41
Bra016770-H2  CGVC------------------------------------------KRKILTMTG----DFRMAQSYFSAGPLAPFYVFP-CG--HSF-----HAQ--CLITHVTSCAHEEQAEHILDLQKQLTLLGSEPPRNINGNRSDEPITSTTTADKLRSELDDAIASECPF--C 111
```

```
Bra016880-H2  CAVC------------------------------------------LSVFEEQ------DTARE--LPN------------CR--HVF-----HVD--C---------------IDT--------WL---TTC-------ST---------------------CPV--C 42
Bra016881-H2  CAVC------------------------------------------LSLLEEK------DTARM--LPN------------CK--HVF-----HVT--C---------------IDT--------WL---TTC-------ST---------------------CPV--C 42
Bra017177-H2  CTVC------------------------------------------KSVLADG------EEIRQ--LSA------------CK--HEF-----HVS--C---------------IEE--------WL---QTR-------SN---------------------CPN--C 42
Bra017205-H2  CAIC------------------------------------------LAEYKEG------EELGE--LKG------------CG--HDY-----HGG--C---------------IKK--------WL---SLK-------NS---------------------CPI--C 42
Bra017280-H2  CSIC------------------------------------------LGDYKGN------HLLRQ--LPD------------CN--HLF-----HLK--C---------------IDT--------WL---RLN-------PT---------------------CPV--C 42
Bra017320-H2  CAIC------------------------------------------LAEFSDE------DTVRL--ITV------------CR--HGF-----HST--C---------------IDS--------WF---ESH-------KT---------------------CPV--C 42
Bra017465-H2  CIVC------------------------------------------LSTLKTG------EQVRK--LD-------------CR--HVF-----HKQ--C---------------LE--------GWI---QHL-------NFN--------------------CPL--C 42
Bra017535-H2  CAIC-----------------------------------------LLEFEEEHV------LLRL--LTT------------CY--HVF-----HQE--C---------------IDR--------WL---ESN-------KT---------------------CPV--C 43
Bra017713-H2  CAIC------------------------------------------LTEFSDG------EEIRI--LPL------------CS--HAF-----HVA--C---------------IDK--------WL---TSR-------SS---------------------CPS--C 42
Bra017732-H2  CSVC------------------------------------------LQDFQLG------ETVRS--LPH------------CH--HMF-----HLP--C---------------IDN--------WL---LRH-------GS---------------------CPM--C 42
Bra017859-H2  CGIC------------------------------------------LQSAKAGR------GTAI--FTAE-----------CS--HTF-----HFP--C----------------VA-------SRAG--DLT-------LLAA-------------------CPV--C 46
Bra018091-H2  CLIC------------------------------------------LEEFQIG------HEVRG--LP-------------CA--HNF-----HVE--C---------------IDQ--------WL---RLN-------VK---------------------CPR--C 41
Bra018644-H2  CCMC------------------------------------------FDPVSIRG-----DTVVV--FF-------------CC--HAY-----HMT--CLMDAAFSDSN-----IKTAAKGSSGYGYGYDNGVEEEDTEDEEEDDSNDGDRSGRSRLR-----CIL--C 86
Bra018663-H2  CAIC------------------------------------------LDEIVKGD------GKAI--FTAE-----------CS--HTF-----HFD--C---------------ITTN----------VKHGN-------KI---------------------CPL--C 44
Bra018777-H2  CVIC------------------------------------------LSDFVSG------EKLRL--LPN------------CN--HGF-----HVR--C---------------IDK--------WL---QQH-------LT---------------------CPS--C 42
Bra018779-H2  CVIC------------------------------------------LSDFVSG------EKLRL--LPK------------CN--HGF-----HVH--C---------------IDK--------WL---QQH-------LT---------------------CPS--C 42
Bra018780-H2  CVIC------------------------------------------LSDFVSG------EKLRL--LPK------------CN--HGF-----HVC--C---------------IDK--------WL---QQH-------LT---------------------CPK--C 42
Bra018781-H2  CAIC------------------------------------------LSEFVSE------ERVKL--LPT------------CH--HGF-----HVR--C---------------IDK--------WL---SSH-------SS---------------------CPT--C 42
Bra019006-H2  CAIC------------------------------------------LSGYVVN------EECRV--FPV------------CR--HMY-----HAV--C---------------IDA--------WL---KNH-------LT---------------------CPT--C 42
Bra019109-H2  CSVC------------------------------------------LDEFDKG------CEAKE--MP-------------CK--HKF-----HIR--C---------------IVP--------WL---ELH-------SS---------------------CPV--C 41
Bra019181-H2  CAIC------------------------------------------REPMAKA------KRLH------------------CN--HLF-----HLG--C---------------LRS--------WL---DQGLN---EVYS---------------------CPT--C 42
Bra019252-H2  CCVC------------------------------------------LGEFELK------EELVE--MPS------------CK--HIF-----HLD--C---------------IHL--------WL---YSH-------TT---------------------CPL--C 42
Bra019304-H2  CPIC----------------------------------------LEEYETENPRL----LTK-------------------CR--HDF-----HLA--C---------------IL--------EWM---ERS-------ES---------------------CPV--C 41
Bra019382-H2  CAVC------------------------------------------TEVFEAG------AEGRE--MP-------------CK--HIF-----HGD--C---------------IVP--------WL---SIR-------NS---------------------CPV--C 41
Bra019499-H2  CVVC------------------------------------------QMEFEEG------ESLVV--LRL------------CN--HPY-----HSE--C---------------ITK--------WL---QTK-------KV---------------------CPI--C 42
Bra019672-H2  CPIC------------------------------------------LDEWSEG------DVAAE--MP-------------CK--HRF-----HSK--C------------------------------VEEWLG---RQAT---------------------CPL--C 41
Bra019673-H2  CGLC-------------------------NRYISQKPPCGSRSIVRNQDMPVTGV----LP--------------------CQ--HVF-----HAE--C---------------------------LDQSTPK------AHRNDPP-----------------CPV--C 59
Bra019769-H2  CGVC------------------------------------------KRKILTMTG----DFRMAQGYSSSGPLAPFYVFP-CG--HSF-----HAQ--CLITHVTSCAHEEQAEHILDLQKQLTLLGSETRRDMNGNRSDEPITSTTTADKLRSELDDAIASECPF--C 111
Bra019784-H2  CCIC------------------------------------------LCAYEDG------SELRE--LP-------------CG--HHF-----HCS--C---------------VDK--------WL---YIN-------AT---------------------CPL--C 41
Bra019945-H2  CSIC------------------------------------------LEDLSCGGG----PGGVPSRMP-------------CS--HVF-----HIR--C---------------LL--------KWFS--RKS-------T----------------------CPM--C 45
Bra020111-H2  CAIC------------------------RNHIMDLCIECQANQASATSEECTVAW-------GV-----------------CN--HAF-----HFH--C---------------ISR--------WL---KTR-------QV---------------------CPL--D 56
Bra020133-H2  CAVC------------------------------------------LGDLEDG------DEVRE--LRN------------CS--HVF-----HRE--C--------------IDRWLDYECCGGDG--NEGEED---NHRT---------------------CPL--C 56
Bra020167-H2  CSIC------------------------------------------LEVFCDSDP-------ST--LTS------------CK--HEY-----HLQ--C---------------IL--------EWC---QRS-------SQ---------------------CPM--C 41
Bra020183-H2  CVIC-----------------------------------------TEEMFLAE------E-ATS--MPC------------CS--HVF-----HSS--C---------------IEK--------WF---QVG-------NK---------------------CPL--C 42
Bra020234-H2  CAIC------------------------------------------LTAMKAGQ------GHAI--FTAE-----------CS--HSF-----HFH--C---------------ITTN----------VKHGN-------QI---------------------CPV--C 44
Bra020313-H2  CAVC------------------------------------------TEVFEAE------TEARE--MP-------------CK--HIF-----HED--C---------------IVP--------WL---SIR-------NS---------------------CPV--C 41
Bra020410-H2  CDLC---------------------------------------DRDLASDPERP-------NAS--LRSLQEACVLA----CG--HVY-----HFK--C---------------LRG-------TTLD--LDN-------PS---------------------CIL--C 54
Bra020507-H2  CPIC------------------------------------------FE-YLFESR-------ND--VTVLP----------CG--HTI-----HQK--C--------------LEEM--------RE---HYQYA----------------------------CPL--C 43
Bra020554-H2  CPDC--------------------------------------------------------NAME-----------------CN--HSF-----HLH--C---------------ILK--------WV---NSQ-------TSQAH------------------CPM--C 34
Bra020591-H2  CAIC------------------------------------------LNEFEDD------ETLRL--LPK------------CD--HVF-----HPH--C---------------IDA--------WL---KSH-------VT---------------------CPV--C 42
Bra021061-H2  CIIC------------------------------------------LSEFKDG------DTLRV--LDR------------CK--HGF-----HVH--C--------------------IQ---QWFS--SNR-------S----------------------CPT--C 42
Bra021160-H2  CIIC------------------------------------------REEMTTA------KKLI------------------CG--HLF-----HVH--C---------------LRS--------WL---ERQ-------QT---------------------CPT--C 38
Bra021193-H2  CAVC------------------------------------------LSEFEEG------ESGRV--LPG------------CK--HAF-----HVE--C---------------IDM--------WF---HSH-------ST---------------------CPL--C 42
Bra021195-H2  CAVC------------------------------------------LSEFEEG------ESGRV--LPG------------CK--HAF-----HVE--C---------------IDM--------WF---HSH-------ST---------------------CPL--C 42
Bra021196-H2  CPVC------------------------------------------LSEFEEG------ESGRV--LPG------------CK--HAF-----HVE--C---------------IDM--------WF---HSH-------ST---------------------CPL--C 42
Bra021280-H2  CPIC---------------------------------------LEHLN--GGLG--------YI--RLRN-----------CM--HKF-----HSR--C---------------IDK--------WL---FKC-------AR---------------------CPL--C 42
Bra021354-H2  CSVC------------------------------------------LCEFQEN------ERLRL--LPE------------CN--HAF-----HVA--C---------------IDT--------WL---KSH-------SN---------------------CPL--C 42
Bra021404-H2  CSVC------------------------------------------LSEFEEE------DQGRI--LPK------------CG--HAF-----HVD--C---------------IDT--------WF---RSR-------ST---------------------CPL--C 42
Bra021467-H2  CAVC------------------------------------------KDEMVLE------EEVKR--LP-------------CR--HLY-----HGE--C---------------ITP--------WL---GIR-------NT---------------------CPV--C 41
Bra021618-H2  CTVC------------------------------------------LFDFAYD------DKVRQ--LPN------------CR--HVF-----HKR--C-----------------------MDRWIV--DCR-------KIT--------------------CPI--C 44
Bra021697-H2  CAIC------------------------------------------HNELGA-------SGELN-TLV-------------CN--HSY-----HHE--C---------------IIG--------WV---KMN-------LT---------------------CPV--C 41
Bra022125-H2  CGIC---------------------------------------LEELNPRGEI---------YF--DMPN-----------CS--HQF-----HDI--C------------------------------ISRWLR---RSKT---------------------CPL--C 43
Bra022144-H2  CAIC------------------------------------------LSEFEQG------ESIQV--LEK------------CH--HGF-----HVK--C---------------IHK--------WL---SSR-------SS---------------------CPT--C 42
Bra022174-H2  CAVC------------------------------------------LSEFEEG------ESGRV--LPG------------CK--HTF-----HVD--C---------------IDM--------WF---HSH-------ST---------------------CPL--C 42
Bra022310-H2  CPIC--------------------------------------CEFLFTSSEAV---------RA--LP-------------CG--HYM-----HSA--C------------------------FQAYT--CS--------HYT--------------------CPI--C 42
Bra022354-H2  CVIC------------------------------------------LSDFVAG------ERIRL--LPK------------CN--HGF-----HVR--C---------------IDK--------WL---TQH-------IT---------------------CPK--C 42
Bra022355-H2  CAIC------------------------------------------LSEFVSG------ERVKL--LPK------------CH--HGF-----HVR--C---------------IDK--------WL---SSH-------SS---------------------CPT--C 42
Bra022370-H2  CAVC------------------------------------------LLEFEEG------DYVRT--LPL------------CF--HAF-----HLE--C---------------IDE--------WL---RSH-------PN---------------------CPL--C 42
Bra022466-H2  CVIC------------------------------------------RLDYEDD------DDLIL--LP-------------CK--HSY-----HSE--C---------------INN--------WL---KIN-------KS---------------------CPV--C 41
Bra022498-H2  CIIC------------------------------------------REEMTSA------KKLV------------------CG--HLF-----HVH--C---------------LRS--------WL---ERQ-------NT---------------------CPT--C 38
Bra022630-H2  CSIC------------------------------------------LSEYEPK------EILKT--IPP------------CQ--HCF-----HAD--C---------------IDE--------WL---KLN-------GT---------------------CPV--C 42
Bra022849-H2  CPIC------------------------------------------CKEFGT-------EGDIN-SLN-------------CE--HSY-----HHH--C---------------ILD--------WV---KKS-------LT---------------------CPY--C 41
Bra023041-H2  CPIC------------------------------------------LGDYKGN------HLLRQ--LPD------------CK--HLF-----HLK--C---------------IDT--------WL---RLN-------PT---------------------CPV--C 42
Bra023042-H2  CHIC------------------------------------------LGDFKGN------HMLRQ--LPD------------CN--HLF-----HLK--C---------------VDT--------WL---RQN-------PT---------------------CPV--C 42
Bra023101-H2  CAIC------------------------------------------LEEYVEG------EELGE--LKG------------CG--HDY-----HGG--C---------------IKK--------WL---SMK-------NS---------------------CPI--C 42
Bra023261-H2  CSIC------------------------------------------LLEFMDD------DTMRL--IST------------CN--HVF-----HTI--C---------------IDL--------WF---ESH-------KT---------------------CPV--C 42
Bra023539-H2  CAIC------------------------------------------KDEIVVE------EKVKR--LP-------------CR--HYY-----HKE--C---------------IVP--------WL---GIR-------NT---------------------CPV--C 41
Bra023814-H2  CSAC------------------------------------------LNEFHVN------ETVRV--IPH------------CF--HLF-----HVD--C-----------------------------VDPKN-------VS---------------------CPL--C 39
Bra023832-H2  CIVC------------------------------------------FGNYSQH------NNLCI--LT-------------CG--HSF-----HFA--C-----------------------IDQWIR--RD--------IS---------------------CPI--C 41
Bra024007-H2  CSIC------------------------------------------QEEYTIG------DEVGR--LH-------------CE--HAY-----HLK--C---------------VQ--------EWLR--MKS-------W----------------------CPI--C 41
Bra024009-H2  CSIC------------------------------------------QEEYTIG------DEVGR--LH-------------CE--HTY-----HLK--C---------------VQ--------EWLR--MKS-------W----------------------CPI--C 41
Bra024081-H2  CAVC------------------------------------------LCEFETE------DKLRL--LPK------------CS--HAF-----HVD--C---------------IDT--------WL---LSH-------ST---------------------CPL--C 42
Bra024178-H2  CSVC------------------------------------------LSKFESV------EILRL--LPK------------CR--HAF-----HIG--C---------------IDQ--------WL---EEH-------AT---------------------CPL--C 42
Bra024408-H2  CGIC------------------------------------------LQRLNSGQI----NSTAI--FTAE-----------CS--HSF-----HLS--C-------------------------AVKL--DGK-------R----------------------CPF--C 43
Bra024491-H2  CSIC------------------------------------------LQDYQRG------EIVRI--LPS------------CR--HMF-----HLP--C---------------ID--------SWF---LEH-------RS---------------------CPL--C 42
Bra024512-H2  CAIC------------------------------------------LSDFADG------EEIRV--LPL------------CG--HSF-----HVE--C---------------IDK--------WL---VSR-------SS---------------------CPS--C 42
Bra024529-H2  CAIC------------------------------------------LEDYSVG------EKLRV--LP-------------CC--HKF-----HAT--C---------------VD--------VWL---TSW-------RTF--------------------CPV--C 42
Bra024628-H2  CAVC------------------------------------------LCEFSED------DKLRL--LPI------------CS--HAF-----HID--C---------------IDT--------WL---LSN-------ST---------------------CPL--C 42
Bra024853-H2  CCIC------------------------------------------LSSYEDG------AELHT--LP-------------CN--HHF-----HFN--C---------------IVK--------WL---KMK-------AT---------------------CPL--C 41
Bra024902-H2  CIVC------------------------------------------LSTLKTG------EEVRK--LD-------------CR--HVF-----HKH--C---------------LE--------GWL---QHL-------NFN--------------------CPL--C 42
Bra024913-H2  CAIC---------------------------------LEPLSHCDDNS-PSEAT--------FT--GQ-------------CS--HSF-----HFS--C---------------IASN----------VRHGS-------VT---------------------CPV--C 48
Bra024929-H2  CAIC------------------------------------------LSEFEPG------ESIHV--LEK------------CH--HGF-----HVK--C---------------IHK--------WL---SSH-------SS---------------------CPT--C 42
Bra025008-H2  CAIC-----------------------------------------LLEFEEEHI------LLRL--LTT------------CY--HVF-----HQE--C---------------IDR--------WL---DSN-------KT---------------------CPV--C 43
Bra025085-H2  CYIC------------------------------------------LVEYEEG------DTIRT--LP-------------CH--HEF-----HRT--C---------------VDK--------WL---KEI-------HRV--------------------CPL--C 42
Bra025417-H2  CAIC------------------------------------------REELTAN------ERLSE--LP-------------CS--HCY-----HKD--C---------------ISN--------WL---SNR-------NT---------------------CPL--C 41
Bra025504-H2  CPIC----------------------------------------LEEYDMDNPKL----LAK-------------------CE--HHF-----HLA--C---------------IL--------EWM---ERS-------ET---------------------CPV--C 41
Bra025597-H2  CAVC------------------------------------------LCEFTEK------DKLRL--LPT------------CS--HAF-----HLN--C---------------IDT--------WL---QSN-------ST---------------------CPL--C 42
Bra025698-H2  CSIC--------------------------------------HEYIFNSNSPV---------KA--LP-------------CG--HVM-----HST--C------------------------FQEYT--CF--------HYT--------------------CPI--C 42
Bra025846-H2  CAIC------------------------------------------LTEFSAG------DELRV--MPQ------------CG--HGF-----HLS--C---------------IDT--------WL---GSH-------SS---------------------CPS--C 42
Bra026165-H2  CVVC------------------------------------------LSELHEG------EEVRK--LE-------------CG--HVF-----HKP--C---------------LE--------GWL---HHL-------HFT--------------------CPL--C 42
Bra026337-H2  CVVC------------------------------------------LFELADG------DKAKF--LPS------------CK--HWF-----HAR--C---------------IDP--------WL---ESH-------AT---------------------CPI--C 42
Bra026420-H2  CCIC------------------------------------------LAKYKDK------EEVRK--LP-------------CS--HRF-----HVK--C---------------VDQ--------WL---RII-------SS---------------------CPL--C 41
Bra026444-H2  CSVC------------------------------------------LDEFEKG------SEAKE--MP-------------CK--HKF-----HVG--C---------------IVP--------WL---ELH-------SS---------------------CPV--C 41
Bra026849-H2  CSIC------------------------------------------LEEWSEG------DVAAE--MP-------------CK--HEF-----HSK--C------------------------------VEEWLG---RHAT---------------------CPL--C 41
Bra026851-H2  CGLC-------------------------NRYISQKSPWGSRSIMRNRDMPVTGV----LP--------------------CQ--HVF-----HAE--C---------------------------LDQSTPK------THGNDPP-----------------CPV--C 59
Bra026955-H2  CCIC------------------------------------------LSAYEDG------TELRE--LP-------------CG--HHF-----HCS--C---------------VDK--------WL---YIN-------AT---------------------CPL--C 41
Bra027034-H2  CAIC------------------------------------------HNELGA-------SGDLN-TLV-------------CN--HSY-----HHQ--C---------------ILG--------WI---KMN-------LT---------------------CPV--C 41
Bra027282-H2  CTIC------------------------------------------QENYKNQ------DKIAT--LD-------------CR--HEY-----HAE--C---------------LK--------KWLV--IKN-------I----------------------CPV--C 41
Bra027290-H2  CWIC---------------------------------------REFVSDNAPD------DRSQDFKCTQ------------CA--HKY-----HGV--C---------------LQE--------RS---KRR-------EPFPE------------------TYF--C 50
Bra027349-H2  CVVC------------------------------------------LSNLVDG------DKARV--MPT------------CK--HCF-----HVD--C---------------IDK--------WL---KCN-------SI---------------------CPV--C 42
Bra027436-H2  CTVC------------------------------------------LDDFEIG------VEAKE--MP-------------CK--HKF-----HSE--C---------------LLP--------WL---ELH-------SS---------------------CPV--C 41
Bra027467-H2  CCIC------------------------------------------QEEYSEG------EDMGA--LE-------------CG--HDF-----HSQ--C---------------IK--------EWLK--QKN-------L----------------------CPI--C 41
Bra027562-H2  CTIC------------------------------------------QEPYRAG------DAVRT--LP-------------CD--HSF-----HSI--C---------------VDQ--------WL-----I-------SDHGD------------------CPL--C 42
Bra027625-H2  CCIC------------------------------------------LSAYADE------TELRE--LP-------------CG--HHF-----HCS--C---------------VDK--------WL---YIN-------AT---------------------CPL--C 41
Bra027664-H2  CAVC------------------------------------------LYDFEND------DEIRR--LTN------------CR--HIF-----HKE--C-----------------------LDRWIM--DYS-------QMT--------------------CPL--C 44
Bra027782-H2  CAVC------------------------------------------LYDFENH------DEIRR--LTN------------CR--HIF-----HKG--C-----------------------LDRWMM--DYN-------QIT--------------------CPL--C 44
Bra028004-H2  CSVC------------------------------------------LGDYQAD------DKLQQ--IPA------------CG--HTF-----HMD--C---------------IDL--------WL---TSH-------TS---------------------CPL--C 42
Bra028075-H2  CAIC------------------------------------------LNEFDDG------EELRL--MPA------------CS--HAF-----HAP--C---------------IDV--------WL---SSR-------ST---------------------CPV--C 42
Bra028093-H2  CPVC------------------------------------------LCEFEAD------DKLRL--LPK------------CS--HAF-----HVD--C---------------IDT--------WL---LSH-------ST---------------------CPL--C 42
Bra028138-H2  CSIC---------------------------------------IEDLS-KSQQS--------II--EMPN-----------CL--HMF-----HQN--C---------------LF--------EWLG--RKN-------S----------------------CPL--C 43
Bra028140-H2  CSIC---------------------------------------IEDFS-VSHEN--------II--WMPQ-----------CK--HVF-----HQG--C---------------LF--------EWLS--RQN-------S----------------------CPL--C 43
Bra028173-H2  CTVC------------------------------------------MEDFVPG------GEATE--LP-------------CK--HIY-----HKN--C---------------ITP--------WL---RLH-------NS---------------------CPI--C 41
Bra028213-H2  CPTC----------------------------------------LDDYTPENPKI----ITK-------------------CS--HHF-----HLS--C---------------IY--------EWM---ERS-------ET---------------------CPV--C 41
Bra028453-H2  CAVC------------------------------------------LCEFTER------DKLRL--LPM------------CS--HAF-----HLT--C---------------IDT--------WL---QSN-------ST---------------------CPL--C 42
Bra028464-H2  CAIC------------------------------------------LEDLLVGSA-------AS--TLP------------CQ--HHF-----HTG--C---------------IL--------EWL---KNS-------RF---------------------CPT--C 41
Bra028731-H2  CSIC------------------------------------------LADYKKT------DMIRV--LPD------------CN--HLF-----HDK--C---------------VDP--------WL---KLH-------PT---------------------CPV--C 42
Bra028866-H2  CVIC------------------------------------------KEEMGEG------RDVCE--MP-------------CQ--HLF-----HWK--C---------------ILP--------WL---SKR-------NT---------------------CPF--C 41
Bra028915-H2  CAIC------------------------------------------LGEFADG------EKVRV--LPP------------CN--HSF-----HMS--C---------------IDT--------WL---VSH-------SS---------------------CPN--C 42
Bra028935-H2  CSVC------------------------------------------LDEFERG------VEAKE--MP-------------CK--HKF-----HVK--C---------------IVP--------WL---EIH-------SS---------------------CPV--C 41
Bra028950-H2  CCIC------------------------------------------LAKYKDK------EEVRK--LP-------------CS--HKF-----HLK--C---------------VDQ--------WL---RII-------SC---------------------CPL--C 41
```

```
Bra029441-H2   CSIC------------------------------------------LDNLVS-----DKHGVST-RMT-------------CS--HVF-----HEK--C---------------LL--------VWFQ--RKN-------T----------------------CPL--C 43
Bra029442-H2   CSIC------------------------------------------LDNLVVSGRSNSKRGIPT-RMT-------------CS--HVF-----HDG--C---------------LL--------EWLQ--RKN-------T----------------------CPL--C 48
Bra029869-H2   CPVC------------------------------------------QDRFEMG------SSARK--MP-------------CR--HIY-----HSE--C---------------IVP--------WL---FQH-------NS---------------------CPV--C 41
Bra030136-H2   CAVC------------------------------------------KDAMVMS------EIGKK--LP-------------CG--HFY-----HDN--C---------------ILP--------WL---ETR-------NS---------------------CPV--C 41
Bra030218-H2   CGIC------------------------------------------SQSVKPGQ------GTAI--FTAE-----------CS--HTF-----HFP--C--------------VTSR------------AAADHN---RLVT---------------------CPV--C 47
Bra030408-H2   CSIC------------------------------------------LEEYEDD------HEIMR--LNK------------CG--HIF-----HHF--C---------------MD--------SWL---ARH-------RS---------------------CPN--C 42
Bra030712-H2   CAIC------------------------------------------LDEIRKED------GKAI--FTAE-----------CS--HSF-----HFD--C---------------ITSN----------VKHGN-------RI---------------------CPL--C 44
Bra030854-H2   CSVC------------------------------------------LDEFEIG------VEAKE--MP-------------CE--HKF-----HGE--C---------------LLP--------WL---ELH-------SS---------------------CPV--C 41
Bra030982-H2   CPIC------------------------------------------WEEFGT-------ELDIN-SLS-------------CN--HTY-----HHH--C---------------ISN--------WV---EKT-------LT---------------------CPY--C 41
Bra030987-H2   CSIC------------------------------------------LSPFKPE------DRLRH--LPL------------CS--HAF-----HLD--C---------------IST--------WL---ISN-------KT---------------------CPL--C 42
Bra031056-H2   CPIC--------------------------------------HEYIFTSNSPA---------KA--LP-------------CG--HVM-----HSA--C-----------------------FQEYT---CS--------HYT--------------------CPI--C 42
Bra031193-H2   CGAC------------------------SRPLSQKSLLSSQKIFATNELSVAA---------IL--A--------------CG--HVY-----HSE--C---------------------------LEQMTPE------ADKFDPQ-----------------CPV--C 59
Bra031441-H2   CSAC------------------------------------------MNEFHVN------ETVRV--IPH------------CF--HLF-----HID--C-----------------------------VDPKN-------VN---------------------CPL--C 39
Bra031849-H2   CGIC------------------------------------------LQRVHSDQI----NSTAI--FTAE-----------CS--HSF-----HLS--C-------------------------AVRL--QDK-------R----------------------CPF--C 43
Bra032086-H2   CAIC------------------------------------------LEDMSEEES----EQVLC--QPPE-----------CL--HMF-----HED--C---------------LTN--------WL---DRH-------DS---------------------CPL--C 45
Bra032514-H2   CSVC------------------------------------------LNEFQED------EKLRI--IPN------------CC--HVF-----HID--C---------------IDI--------WL---QGN-------AN---------------------CPL--C 42
Bra033193-H2   CSVC------------------------------------------LGDYQAN------EKLQQ--IPP------------CG--HTF-----HMD--C---------------IDL--------WL---TLH-------TT---------------------CPL--C 42
Bra033599-H2   CAAC------------------------ARPLSEKSLWSRQNIFMTNELSVSA---------VL--A--------------CG--HVY-----HGD--C---------------------------LEQMTPE------VDKFDPS-----------------CPI--C 59
Bra033652-H2   CCIC------------------------------------------QEEYTEG------DDMGT--LE-------------CG--HEF-----HSQ--C---------------IK--------EWLK--QKN-------L----------------------CPI--C 41
Bra033680-H2   CSVC------------------------------------------LNEFQEE------EKLRI--IPH------------CS--HLF-----HID--C---------------IDI--------WL---QNN-------AN---------------------CPL--C 42
Bra033777-H2   CLIC------------------------------------------LEEFHIG------HEVRG--LP-------------CA--HNF-----HVE--C---------------IDQ--------WL---RLN-------VK---------------------CPR--C 41
Bra033953-H2   CAIC------------------------------------------KEEFEVG------EEGKE--LK-------------CL--HLY-----HPS--C---------------IVS--------WL---NIH-------NT---------------------CPI--C 41
Bra033963-H2   CCIC------------------------------------------LSSYEDG------IELVT--LP-------------CN--HHF-----HST--C---------------IVK--------WL---KMN-------AT---------------------CPL--C 41
Bra034152-H2   CLIC------------------------------------------LGDFVEG------EKVRV--LPK------------CN--HGF-----HVR--C---------------IDT--------WL---LSR-------DS---------------------CPT--C 42
Bra034259-H2   CAIC------------------------------------------LEDLWTN-------KQLK--LPH------------CS--HQF-----HFR--C---------------ISK--------WL---QSR-------NS---------------------CPL--C 41
Bra034573-H2   CSVC------------------------------------------LSEFEEE------ETLRL--LPK------------CR--HAF-----HVP--C---------------IDT--------WL---RSH-------TN---------------------CPV--C 42
Bra034865-H2   CCIC------------------------------------------LGGLEEG------EKMKV--LPL------------CR--HCY-----HCE--C---------------VDR--------WL---MTE-------SS---------------------CPL--C 42
Bra035010-H2   CPVC------------------------------------------LENIESCQ------SARL--VPG------------CN--HGF-----HQL--C--------------------------ADTWLSNH-------TV---------------------CPV--C 42
Bra035230-H2   CTVC------------------------------------------LSDFESD------DDIRQ--LPN------------CR--HVF-----HHR--C-----------------------LDRWIV--DCC-------KVT--------------------CPV--C 44
Bra035284-H2   CSIC------------------------------------------LDNLYGS---SSIHGSAT-LMN-------------CS--HVF-----HER--C---------------LS--------DWLQ--QKN-------T----------------------CPM--C 45
Bra035483-H2   CSIC------------------------------------------LIDFMDD------DTMRL--IST------------CN--HFF-----HTI--C---------------IDL--------WF---ESH-------KT---------------------CPV--C 42
Bra035660-H2   CVIC------------------------------------------LEDFEVN------DVVRI--LVG------------CK--HVF-----HVE--C---------------IDS--------WC---FYK-------LT---------------------CPV--C 42
Bra035785-H2   CSIC------------------------------------------LQDWEEG------QVGRK--LER------------CG--HTF-----HMK--C---------------ID--------EWF---LRQ-------VS---------------------CPI--C 42
Bra035835-H2   CAVC------------------------------------------MDEFEDG------GDVKQ--VP-------------CK--HVF-----HQD--C---------------LMP--------WL---ELH-------NS---------------------CPV--C 41
Bra036197-H2   CAIC------------------------------------------REKLAPS------ERLSE--LP-------------CR--HYY-----HKD--C---------------ISS--------WL---TNR-------NT---------------------CPL--C 41
Bra036491-H2   CVIC---------------------------------------MATIDLRRRTND-------CM--VTP------------CE--HLF-----HSG--C---------------LQ--------RWMD--IKM-------E----------------------CPT--C 44
Bra036631-H2   CAIC------------------------------------------LNEFEDD------ETLCL--LPK------------CD--HVF-----HPH--C---------------IGA--------WL---EGH-------VT---------------------CPV--C 42
Bra036816-H2   CSVC------------------------------------------LMRMEDE------DAIKS--LP-------------CS--HEF-----HSL--C---------------VDT--------WF---DVS-------RKIC-------------------CPL--C 43
Bra037002-H2   CSVC------------------------------------------LSEFEEE------EMLRL--LPK------------CR--HAF-----HLS--C---------------IDT--------WL---RSH-------TN---------------------CPL--C 42
Bra037109-H2   CSVC------------------------------------------LDDFEVG------VEAKQ--MP-------------CK--HNF-----HAD--C---------------LLP--------WL---ELH-------SS---------------------CPV--C 41
Bra037333-H2   CPIC----------------------------------------FEDYEADNPKI----KTN-------------------CE--HDF-----HLS--C---------------IL--------EWM---EKS-------DG---------------------CPI--C 41
Bra037367-H2   CSIC---------------------------------------YEDLK-PAAEN--------LQ--SISA-----------CG--HVF-----HEL--C---------------LQ--------QWFE--YCP-------STNKRN-----------------CPI--C 48
Bra037410-H2   CCIC------------------------------------------NCPLTKTFS----TLRVR--VFN------------CG--HAT-----HLQ--C----------------------------EVSESE-------TSSSG------------------CPV--C 45
Bra037561-H2   CAIC------------------------------------------LSEFVSG------ERVKL--LPK------------CH--HGF-----HVR--C---------------IDK--------WL---SSH-------SS---------------------CPT--C 42
Bra037562-H2   CVIC------------------------------------------LSDFVAG------EQVRL--LPK------------CN--HGF-----HVR--C---------------IDK--------WL---KQH-------VT---------------------CPK--C 42
Bra037592-H2   CPIC--------------------------------------CEFLFTSSETV---------RA--LP-------------CG--HYM-----HSA--C-----------------------FQAYT---CS--------HYT--------------------CPI--C 42
Bra037644-H2   CSIC------------------------------------------LQDAAEG------EKMRR--LTA------------CG--HCF-----HAE--C---------------IDP--------WL---EKR-------ST---------------------CPQ--C 42
Bra037800-H2   CAVC------------------------------------------KDGMVIG------ETVKK--LP-------------CG--HCY-----HGN--C---------------ILP--------WL---GTR-------NS---------------------CPV--C 41
Bra037999-H2   CCVC------------------------------------------LDDFEMG------TVGKQ--MP-------------CK--HIF-----HSD--C---------------LLP--------WL---QLH-------SS---------------------CPV--C 41
Bra038051-H2   CAVC------------------------------------------LSEFEES------EPGRV--LPN------------CK--HAF-----HVE--C---------------IDM--------WF---LSH-------SS---------------------CPL--C 42
Bra038109-H2   CTIC------------------------------------------QESFKNE------EKIAT--LD-------------CG--HEY-----HAG--C---------------LE--------KWLV--VKN-------V----------------------CPI--C 41
Bra038199-H2   CAVC------------------------------------------LGDLEDG------DEVRE--LRN------------CS--HVF-----HRE--C--------------IDRWLDYEC---DD--NDGEED---NHRT---------------------CPL--C 53
Bra038240-H2   CVIC------------------------------------------RLDYEDD------DDLIL--LP-------------CK--HSY-----HSE--C---------------INN--------WL---KIN-------KI---------------------CPV--C 41
Bra038246-H2   CAVC------------------------------------------MDEFEDG------GDVKQ--MP-------------CK--HVF-----HQD--C---------------LMP--------WL---ELH-------NS---------------------CPV--C 41
Bra038386-H2   CAVC------------------------------------------LNEYKED------EMLRL--ILP------------CG--HVF-----HVH--C---------------VDI--------WF---SYR-------ST---------------------CPI--C 42
Bra038545-H2   CPVC------------------------------------------LCEFEAE------DKLRL--LPK------------CS--HAF-----HVD--C---------------IDT--------WL---LSH-------ST---------------------CPL--C 42
Bra038813-H2   CVVC------------------------------------------LDGFRQG------QWCRN--LPG------------CG--HVF-----HRK--C---------------VDT--------WL---LKA-------AS---------------------CPI--C 42
Bra039168-H2   CPTC----------------------------------------LEETYSENPKI----VTK-------------------CS--HHF-----HLG--C---------------IY--------EWM---ERS-------EN---------------------CPV--C 41
Bra039281-H2   CPIC------------------------------------------LSEYASK------ETVRC--IPE------------CE--HCF-----HIE--C---------------IDA--------WL---KLH-------GS---------------------CPL--C 42
Bra039282-H2   CPIC------------------------------------------LSEYASK------ETIRF--MPE------------CD--HCF-----HVE--C---------------IDV--------WL---KIH-------GS---------------------CPL--C 42
Bra039287-H2   CTIC------------------------------------------LCEYKEA------EMLRM--MPE------------CK--HYF-----HLC--C---------------LDA--------WL---KLN-------GS---------------------CPV--C 42
Bra039317-H2   CCVC------------------------------------------LCEYEDG------TELRE--LW-------------CR--HHF-----HEA--C---------------IDK--------WL---RIN-------AT---------------------CPL--C 41
Bra039318-H2   CCVC------------------------------------------LCEYEDG------TELRE--LW-------------CR--HHF-----HEA--C---------------IDK--------WL---RIN-------AT---------------------CPL--C 41
Bra039430-H2   CSIC------------------------------------------LDTVAKDGD----RAWAN--LQ-------------CG--HLF-----HLD--C-------------------------------IG--------SAFNAKGVMQ-------------CPN--C 45
Bra039454-H2   CAIC------------------------------------------LNEFEDD------ETLRL--VPK------------CD--HVF-----HPH--C---------------IGA--------WL---EAH-------VT---------------------CPV--C 42
Bra039652-H2   CSIC------------------------------------------LESFTNG------DMLIS--LP-------------CT--HSF-----HSS--C---------------LN--------PWLK--ACG-------D----------------------CPY--C 41
Bra039659-H2   CTIC------------------------------------------QESFKNE------EKIAT--LD-------------CG--HEY-----HAE--C---------------LE--------KWLI--VKN-------V----------------------CPI--C 41
Bra039822-H2   CCQC----------------------------------------QSTFDEGG-------GLQVT-RLG-------------CL--HAI-----HTS--C----------------LV-------SLIK--SFP-------PHTAPAGYV--------------CPS--C 51
Bra039842-H2   CCIC------------------------------------------QEEYAEG------DDLGT--LE-------------CG--HEF-----HKD--C---------------IK--------QWVM--LKN-------L----------------------CPI--C 41
Bra040087-H2   CCIC------------------------------------------LAKYANN------EELRE--LP-------------CS--HFF-----HKE--C---------------VDK--------WL---KIN-------AS---------------------CPL--C 41
Bra040450-H2   CVIC------------------------------------------QLKYKIG------EKQMN--LP-------------CK--HVY-----HSE--C---------------ISK--------WL---SIN-------KI---------------------CPV--C 41
Bra040498-H2   CGIC------------------------------------------LNSVKTGQ------GTAK--YTAE-----------CS--HVF-----HFP--C-----------------------VTDYVS--KHG-------KLV--------------------CPV--C 45
Bra040540-H2   CSIC------------------------------------------IETVTKEGD----RAWAK--LH-------------CG--HEF-----HLD--C-------------------------------IG--------SAFNTKGVMQ-------------CPN--C 45
Bra040564-H2   CAVC------------------------------------------LSHLVDG------DKARV--LPG------------CN--HGF-----HVD--C---------------IDM--------WF---QSH-------ST---------------------CPL--C 42
Bra040642-H2   CAIC------------------------------------------LNEFEDD------ETLRL--LPK------------CD--HVF-----HPH--C---------------IDA--------WL---EAH-------VT---------------------CPV--C 42
Bra040855-H2   CPVC------------------------------------------LNEFEDD------ETLRL--LPQ------------CC--HVF-----HPG--C---------------IDA--------WL---RSH-------AT---------------------CPL--C 42
Bra040966-H2   CCIC------------------------------------------LCEYEDG------VELRE--LP-------------CN--HHF-----HCT--C---------------IDK--------WL---HIN-------SR---------------------CPL--C 41
Bra000043-HCa  CAIC-------------------------------------------FD--ARRD----CFFLP-----------------CG--HCVS----CYQ--C----------------GT-------KIAE--AAG-------S----------------------CPV--C 39
Bra000106-HCa  CAIC------------------------------------------LETVFNP---------YA--LK-------------CG--HIF-----CKA--C-----------ACSAASV-------MIFQ--GVK-------AAPQCSK----------------CPI--C 49
Bra000249-HCa  CPVC------------------------------------------TNLMYPPIN----Q---------------------CPNGHTL-----CSS--C-----------------KQ-------QV---QNT------------------------------CPT--C 36
Bra000250-HCa  CYIC------------------------------------------MDLSKE---------PVV--TN-------------CG--HLY-----CWP--C---------------LY--------RWLE--VSV-------AKE--------------------CPV--C 40
Bra000425-HCa  CPIC------------------------------------------IEPLSIPIF----Q---------------------CDNGHLA-----CSS--C-------------------------------CPK------LKNK--------------------CPS--C 36
Bra000480-HCa  CCIC------------------------------------------FEQVCTIE-----VKD-------------------CG--HQM-----CAQ--C--------------TLA--------LCCH--NKP-------NPTTSTVTPPV------------CPF--C 49
Bra000504b-HCa CTKC------------------------------------------LEIPHHSV------V--L--L--------------CG--HSF-----CYR--C------------------------------GANAGC---CPHC---------------------CPH--C 38
Bra000646-HCa  CPVC---------------------------------------------MDII---------VD--RGLLDC---------CSSYHKG-----CRPYMC--------------------AT---GNRF--SNC-----LEQYKNAYAKDEKSGKPAELL----CPL--C 63
Bra000729-HCa  CGIC---------------------------------------------MGRKK------GAAF--IP-------------CQ--HWF-----CFE--C---------------IDN--------W----SSI-------MNL--------------------CPL--C 38
Bra000784-HCa  CRVC------------------------------------------LSNPKN----------MA--FG-------------CG--HTF-----CRV--C--------------------------SREVWLNR-------GS---------------------CPL--C 37
Bra000997-HCa  CPIC------------------------------------------LSEEDMR-------RLKG-TLDC------------CG--HQT-----CCE--C--------------------------------GP-------D-LKV------------------CPI--C 38
Bra001159-HCa  CGIC---------------------------------------------QVRIK------GATF--TS-------------CS--HYF-----CFT--C----------------------------IMEWSK-------VESR-------------------CPL--C 38
Bra001236-HCa  CCVC------------------------------------------LDRVLSKATP---GERKFGLLTE------------CG--HTF-----CKL--C--------------------------SKELMAQK-------GH---------------------CPV--C 47
Bra001285-HCa  CSVC-------------------------------------------GKGEAS--------VLL--LP-------------CH--HPF-----CIQ--C---------------IRN--------WR---SSAPV---SGMDVNSTLRA--------------CPI--C 49
Bra001477-HCa  CRSC-------------------------------------------NNLYDR--------ATT--ISE------------CR--HMCL----CSV--C--------------------------------G--------SSVYT------------------CPV--C 36
Bra001901-HCa  CPIC------------------------------------------LTEPKDT---------AV--MP-------------CL--HTFY----CKR--C-----------------------------IEDKL-------VVENLKA----------------CPV--C 41
Bra002208-HCa  CVIC------------------------------------------LERKCTVSA-------DG-----------------CR--HLCL----CSD--C--------------------AK---ELRF--QSN-------K----------------------CPI--C 39
Bra002699-HCa  CAVC------------------------------------------LTEPRDT---------TV--LP-------------CG--HEF-----CTN--C------------ALYLSTTTNTSSKTTSQATPGS-------VP---------------------CPL--C 52
Bra003133-HCa  CVIC------------------------------------------QS-LLFDST-------RC--VP-------------CR--HMCM----CSG--C--------------------AK---LLRF--QTN-------L----------------------CPI--C 40
Bra003180-HCa  CMLC------------------------------------------LDLSKE---------PVL--TC-------------CT--HVF-----CKV--C-----------------------L--AR--FKD-------------------------------CPL--C 34
Bra003314-HCa  CNIC------------------------------------------TNLMYPPIH----Q---------------------CG--HLY-----CWP--C---------------LF--------QWLQ--ISE-------AKE--------------------CPV--C 39
Bra003315-HCa  CPVC------------------------------------------TNSMYPPIH----Q---------------------CPNGHTL-----CSN--C----------------KA--------RV---QNT------------------------------CPT--C 36
Bra003473-HCa  CPVC---------------------------------------ADNLE-WVAYG--------S------------------CHNGHTL-----CST--C----------------KN--------RV---HNR------------------------------CPT--C 37
Bra003493-HCa  CAVC-------------------------------------------QVDPATP-------FIA--LP-------------CG--HREV----CST--C--------------------VV---RLRF--VLC-------DPR--------------------CCI--C 42
Bra003533-HCa  CPIC------------------------------------------LESPHNAV------L--L--L--------------CQ--HRY-----CYY--C---------------IRT--------RC---ASA-------ASFR-------------------CLR--C 40
Bra003669-HCa  CPVC------------------------------------------WEPFNVF---------EN--VPYVLW---------CSSYHQG-----CRPYMC--------------------AT---SSRF--ANC-------LDQYRKSYGNSEQAQLL------CPL--C 62
Bra003806-HCa  CPIC------------------------------------------RQVMTL---------PVT--TP-------------CG--HTI-----CKD--C------------LLALHRAVVIKSSGFP---FHL-------PFFVT------------------CPW--C 52
Bra004082a-HCa CKIC------------------------------------------IQLPER---------PVT--TP-------------CA--HNF-----CKA--C---LEDKFAGQTLVRERSRGGR---TLRA--QKN-------VMN--------------------CPC--C 57
Bra004082b-HCa CSFC------------------------------------------FQALTQHVF----Q---------------------CG--HNF-----CLK--C--------------------FE---KWIG--QGK-------RT---------------------CAK--C 38
Bra004174-HCa  CPVC---------------------------------------LEPLR--GPI---------YQ-----------------CDNGHIA-----CSS--C--------------------CR---ELRN--K--------------------------------CPA--C 36
Bra004176-HCa  CPTC------------------------------------------LVIPKN----------MA--FN-------------CHNGHIT-----CSP--C------------------------LPRVY--KH-------------------------------CPV--C 36
Bra004260-HCa  CPIC------------------------------------------FEDPINV---------VL--LP-------------CG--HQT-----CSD--C--------------------------------G--------EDLHV------------------CPI--C 34
Bra004339-HCa  CRVC------------------------------------------FESFNIV---------EN--VPYVLW---------CR--HHVL----CST--C-------------------------------CEK------CKK---------------------CPI--C 39
Bra004373-HCa  CPIC------------------------------------------CHALTNPIF----Q---------------------CG--HTL-----CQN--C--------VFALQPAVL--------RLSS--QDI-------KVPFFVS----------------CPW--C 50
Bra004401-HCa  CPVC------------------------------------------CHALTRPVF----Q---------------------CDNGHIA-----CSS--C--------------------CT---NLRN--K--------------------------------CPY--C 36
Bra004402-HCa  CPVC---------------------------------------AENLE-WVAYG--------F------------------CDNGHIA-----CSS--C--------------------CT---NLRS--K--------------------------------CPS--C 37
Bra004478-HCa  CAVC------------------------------------------LDLSKE---------PVV--TN-------------CG--HREV----CST--C--------------------VV---RLRF--ILG-------DRR--------------------CCI--C 41
Bra004666-HCa  CYVC------------------------------------------LEKAED---------PIL--TC-------------CG--HLF-----CWS--C---------------LY--------RWLQ--VSE-------AKE--------------------CPV--C 40
Bra004829-HCa  CNIC------------------------------------------NDRSKE---------VVI--TK-------------CG--HLF-----CWG--C----------------------FYQLPFI--YLN--------IKE-------------------CPV--C 41
Bra004879-HCa  CKAC-------------------------------------------FD--AKRD----CFFLP-----------------CF--HLF-----CNP--C------------------VQK---------IIGTRQ---RK-----------------------CPT--C 39
Bra005128-HCa  CAIC------------------------------------------LNVWHDV--------VTA--AP-------------CG--HCVA----CYQ--C----------------GT-------KITE--AAG-------S----------------------CPI--C 40
Bra005251-HCa  CSIC-------------------------------------------MKDEVS--------VVF--LP-------------CL--HNF-----CNG--C------------------------------FSEWMR---RSEARHQHVL---------------CPQ--C 44
Bra005352-HCa  CLIC------------------------------------------NASQVET---------LL--YR-------------CA--HQVV----CAS--C---------------------------SESFMG-------GDKAT-------------------CPC--C 40
```

```
Bra005381-HCa CCVC-------------------------------------------MQVIKD----------AF--LTA------------CG--HMCT----CLR--C---------------------AN---ELQC--NGG-------K-----------------------CPI--C 39
Bra005541-HCa CPIC-------------------------------------------LEIRNK----------VV--LPT------------CG--HSF-----CYM--C--------------------IIT---HLRN--KSD-------------------------------CPC--C 38
Bra005682-HCa CGIC--------------------------------------------FD--APRD----CFFLS-----------------CN--HSM-----CIN--C----------------YR--------DWR---LRS-------QS----------------------CPF--C 38
Bra005685-HCa CAIC--------------------------------------------QEKMHTP--------IL--LR-------------CG--HCVA----CFQ--C-----------------GT-------RIAE--TSG-------F-----------------------CPV--C 39
Bra006081-HCa CAIC----------------------------------------HTRYNEDERVP--------LL--LQ-------------CK--HMF-----CED--C-------------------------------VSEWFE---RERT----------------------CPL--C 42
Bra006112b-HCa CEHA-------------------------------------------IEFSATG-------TGSSYDVSCL-----------CS--HSF-----CWN--C------------TQDAHRPVDCDRVSQWIRKNSVEAENTKWILDNSKP------------------CPK--C 70
Bra006205-HCa CSVC-------------------------------------------LSSPKD----------MA--FG-------------CG--HGF-----CKD--C------------------L-------SKM----FS-------SSSDTSLA----------------CPR--C 41
Bra006253-HCa CPIC-----------------------------------------LNAIPLQETAM----VKG-------------------CG--HQT-----CCE--C---------------------------------GP-------D-LEM-------------------CPI--C 37
Bra006505-HCa CAIC-------------------------------------------LEAFED---------AVL--TP-------------CE--HTY-----CVT--C----------------IL--------RWAS--CK--------ESPT--------------------CPQ--C 40
Bra006634-HCa CPIC-------------------------------------------MEMNSL----------AV--LPN------------CA--HRL-----CRE--C---------------LLA--------SWR---NSS-------SGL---------------------CPV--C 40
Bra006745-HCa CGIC-------------------------------------------QA-LLYDSS-------RC--VP-------------CT--HSL-----CIK--C----------------YR--------DWR---GRS-------QS----------------------CPF--C 39
Bra007068-HCa CMLC-------------------------------------------TNLMYPPIH----Q---------------------CT--HVF-----CKV--C------------------------V--AR--FKD--------------------------------CPL--C 33
Bra007376-HCa CPVC----------------------------------------------LDRKC------DAVF--LE-------------CPNGHTL-----CSN--C-----------------KA--------RV--QNT--------------------------------CPT--C 37
Bra007725-HCa CVIC-------------------------------------------LQVMNF---------PVT--TP-------------CG--HMCC----CLT--C---------------------SL---SLEL--QGK-------R-----------------------CPL--C 39
Bra007810a-HCa CQIC-------------------------------------------LQLLER---------PVT--TP-------------CA--HNF-----CKG--C---LEDKFAGQTRMVERSRGGR----TLRA--MKN-------IMK---------------------CPC--C 57
Bra007810b-HCa CSFC-------------------------------------------LESPHNAV------L--L--L--------------CG--HNF-----CLK--C---------------------FV---KWTE--QGK-------RE----------------------CIK--C 39
Bra008335-HCa CSVC-------------------------------------------RNPFLN---------PVV--TN-------------CSSYHKG-----CRPYMC---------------------AT---STRF--ANC-------LDQYRKSHG-SEQLPLL-------CPL--C 57
Bra008536-HCa CSIC-------------------------------------------LSNPKD----------MA--FG-------------CN--HYF-----CNL--C-----------------A--------LMHH--EK--------NTN---------------------CFV--C 37
Bra008758-HCa CPIC-----------------------------------------LDTIPLQETAM----VKG-------------------CG--HQT-----CCE--C---------------------------------GP-------D-LQM-------------------CPI--C 37
Bra008900-HCa CAIC------------------------------------------ISP-PRDII--------IT--R--------------CE--HAY-----CVT--C----------------IL--------RWAS--YK--------EIHT--------------------CPQ--C 40
Bra009089-HCa CPIC-------------------------------------------LERTCTVAA-------EG-----------------CA--HIF-----CRS--C----------------IL--------QTLQ--RTK-------PS----------------------CPL--C 39
Bra009278-HCa CAVC--------------------------------------------DNHIDA---------LL--YR-------------CE--HQL-----CVR--C-------------ALYLCSSSNVPSVT--VDPPGS-------IP----------------------CPL--C 49
Bra009452-HCa CVCC-------------------------------------------LSEPRDV---------LV--LP-------------CG--HMCT----CSQ--C---------------------AN---ELVR--NGG-------K-----------------------CPL--C 39
Bra009546-HCa CVIC------------------------------------------DFPIAIYG---------RL--NP-------------CR--HMCM----CVG--C---------------------AK---ELRF--QTN-------L-----------------------CPV--C 40
Bra009629-HCa CVRC-------------------------------------------TFSMYPPIH----Q---------------------CD--HAF-----CLE--C--------------------------------ARSDS---I-------------------------CYL--C 33
Bra010392-HCa CPLC-------------------------------------------LDTAHD---------PVV--TL-------------CHNGHTL-----CST--C-----------------KV--------RV--HNR--------------------------------CPT--C 37
Bra010403-HCa CNIC-------------------------------------------FESPTAA---------IP--LP-------------CG--HLF-----CWP--C----------------IYK--------WL---HVKLS---PVSIDHHHNT----------------CPV--C 48
Bra010714-HCa CKVC-------------------------------------------LEPCTK----------MV--LPN------------CR--HFCL----CKS--C---------------------------S-----LA-------CSE---------------------CPI--C 35
Bra010963-HCa CGIC-------------------------------------------FGSFTVP---------CR--GD-------------CC--HAM-----CIK--C----------------YR--------NWN---TKS-------ES----------------------CPF--C 38
Bra011479-HCa CPIC-------------------------------------------GSDGETR--------VLF--LP-------------CG--HWY-----CGS--C-----------------------ILQYWNYAAVSR------PCK----------------------CPM--C 44
Bra011582-HCa CLNC-------------------------------------------FEQACTIE-----VKD-------------------CR--HLCC----CTE--C-------------------------------EAGL-------VV----------------------CPI--C 35
Bra011923-HCa CCIC-------------------------------------------HGNFSAP---------CQ--AN-------------CG--HQM-----CAQ--C---------------TLA--------LCCH-NKP--------NPTTSTVTPPV-------------CPF--C 49
Bra011996b-HCa CGKC-------------------------------------------QHMIELSQG----CIRIK--CR-------------CG--HEF-----CYQ--C-------------------------------GAEARR-----------------------------CPH--G 39
Bra012331-HCa CSIC-------------------------------------------CEPLKIPIF----Q---------------------CS--HWF-----CGN--C--------------------IML---VWRH--GST-------LRPCK-------------------CPL--C 42
Bra012354-HCa CPIC-------------------------------------------LEPCTK----------MV--LPN------------CENGHLA-----CSQ--C--------------------------------CEK------VKKI---------------------CPS--C 37
Bra012512-HCa CGIC--------------------------------------------GEREAS--------VLV--LP-------------CC--HAM-----CIK--C----------------YR--------NWN---TKS-------ES----------------------CPF--C 38
Bra012532-HCa CKRC-------------------------------------------RRNSATV---------MA--LP-------------CR--HLCL----CTV--C---------------------------------G--------SALLQT------------------CPV--C 36
Bra012650-HCa CSIC--------------------------------------------LIKEVD--------ITI--VP-------------CR--HLVL----CKG--C--------------------------------DGG-------GDVR-----V--------------CPI--C 37
Bra013017-HCa CKIC-------------------------------------------LELLYK---------PIV--LS-------------CG--HVL-----CRE--C---------------SASV-----------------------AR----------------------CPF--C 34
Bra013241a-HCa CCVC-------------------------------------------KELLVR---------PVV--LN-------------CG--HLS-----CFW--C--------------------------VHKS--MNE-------LRESH-------------------CPI--C 40
Bra013241b-HCa CSAC--------------------------------------------GEREAS--------VLV--LP-------------CG--HVY-----CEG--C------------------------VVDMF-QEGEK------IK-----------------------CQE--C 40
Bra013423-HCa CKRC-------------------------------------------SEMPVES---------LL--YR-------------CR--HLCL----CTV--C---------------------------------G--------SALLRT------------------CPV--C 36
Bra013447-HCa CCVC------HLAIKLVSKVYEREIVVRDRSRFHHFKDGSCSCQDYCLSL-FDSA--------AS--LS-------------CG--HMCT----CLK--C---------------------AH---ELQW--SSK-------K-----------------------CPI--C 76
Bra013499-HCa CADC--------------------------------------------FE--ERRN----CFFVP-----------------CN--HVF-----CNE--C----------------IVK--------SM---KVD-------AT----------------------CPV--C 38
Bra013775-HCa CVIC--------------------------------------------GVREVC--------MLL--LP-------------CG--HSAT----CRG--C-------------------------------AEKI-------MSEENKV-----------------CPI--C 40
Bra014051-HCa CRFC-------------------------------------------EDASSEA---------AC--VP-------------CK--HMCL----CKE--C-------------------------------ERK--------LSS---------------------CPL--C 35
Bra014247-HCa CVIC-------------------------------------------TNSMYPPIH----Q---------------------CG--HVAG----CVS--C----------------LK--------EIKN--KK--------------------------------CPI--C 36
Bra014421-HCa CPVC-------------------------------------------TNLMYPPIH----Q---------------------CQNGHTL-----CST--C-----------------KN--------RV--HNR--------------------------------CPT--C 36
Bra014589-HCa CPVC-------------------------------------------LDVSKE---------PVL--TC-------------CPNGHTL-----CSN--C-----------------KA--------RV--QNT--------------------------------CPT--C 37
Bra014591-HCa CNIC-------------------------------------------MDAPSDG---------IC--VP-------------CG--HLY-----CWL--C----------------LF--------QWLN--ISE-------AKE---------------------CPV--C 40
Bra014932-HCa CLIC-------------------------------------------LDAPSEA---------VC--VP-------------CG--HVAG----CMS--C----------------------------LT--QIK-------SHAS--E-----------------CPI--C 39
Bra014934-HCa CSIC-------------------------------------------LELLYK---------PIV--LS-------------CG--HVAG----CMS--C----------------LT--------EIKA--KKW-------G-----------------------CPV--C 39
Bra015086a-HCa CCVC-------------------------------------------KELLVR---------PVV--LN-------------CG--HLS-----CFW--C--------------------------VHRS--MSG-------LRVSH-------------------CPI--C 40
Bra015086b-HCa CSAC----------------------------------------------MVRSK------GAAF--IP-------------CG--HVY-----CEG--C------------------------VVDMMDQESEK------IK-----------------------CKE--C 41
Bra015092-HCa CCVC--------------------------------------------LGIIR-------KTRT--FME-------------CG--HTF-----CRL--C---------------------------SRELWVQR-------GN----------------------CPL--C 39
Bra015262-HCa CPIC-------------------------------------------HESAED---------SVV--TS-------------CL--HRF-----CQE--C----------------------------IDKSMR-------FGNHE--------------------CPA--C 39
Bra015389-HCa CGLC-------------------------------------------HEPAED---------NVV--TS-------------CS--HVF-----CKA--C-----------------LI-------DFSA--SLG-------KVS---------------------CPT--C 40
Bra015392-HCa CGLC-------------------------------------------LEMARE---------PIV--TL-------------CA--HVF-----CKA--C-----------------LI-------DFSA--SLR-------KVS---------------------CPT--C 40
Bra015877-HCa CNIC-------------------------------------------FEREISV---------VL--LP-------------CG--HLF-----CWP--C----------------LYK--------WI---HFH-------SQSKH-------------------CPV--C 41
Bra015952-HCa CRIC-------------------------------------------HSHFTAP---------CQ--AN-------------CR--HRVL----CRN--C--------------------------------SEK------CKK----------------------CPF--C 35
Bra016082-HCa CSIC-------------------------------------------LERVLSKPTP---AERKFGILTE------------CS--HWF-----CGN--C--------------------IML---VWRH--GST-------LQPCK-------------------CPL--C 52
Bra016385-HCa CCVC-------------------------------------------FEREISL---------VL--LP-------------CD--HAF-----CIG--C----------------IRN--------WR---SSSPS---TGMDVNSTLRA---------------CPI--C 49
Bra016557-HCa CRVC-------------------------------------------TNLMYPPIF----Q---------------------CR--HRVL----CRL--C--------------------------------SDK------CKK----------------------CPA--C 34
Bra016898-HCa CLAC-------------------------------------------LENLTHRR------A-AV--IPS------------CPNGHTL-----CST--C-----------------KL--------KV--HNT--------------------------------CPT--C 40
Bra017091-HCa CPIC-------------------------------------------LETVFNP---------YA--LK-------------CR--HGY-----CLG--C----------------IRK--------WS---GLK-------RS----------------------CPL--C 38
Bra017094-HCa CAIC-------------------------------------------RRRAATV---------LA--LP-------------CG--HIY-----CKA--C------------ACSSASV-------MIFQ--GVK-------AAPQCSK-----------------CPI--C 49
Bra017511-HCa CRIC-------------------------------------------FSSFTVP---------CR--GN-------------CR--HLVL----CKG--C--------------------------------DG--------SVR------V--------------CPL--C 35
Bra017614-HCa CPIC-------------------------------------------GLNGETR--------VLF--LP-------------CG--HWY-----CGS--C-----------------------ILQYWNYAAVSK------PCK----------------------CPM--C 44
Bra017691-HCa CLNC-------------------------------------------LQSYDGES-----TLPRV--LS-------------CR--HLCC----CTG--C-------------------------------EDGL-------VL----------------------CPI--C 39
Bra017935-HCa CPVC-------------------------------------------MEMNSM----------VV--LPN------------CG--HTA-----CEE--C----------------LT--------NIP---KKF-------PDTIR-------------------CPA--C 41
Bra018162-HCa CGIC-------------------------------------------CVPLTSNIF----Q---------------------CT--HSL-----CIK--C----------------YR--------DWH---GRS-------ES----------------------CPF--C 37
Bra018221-HCa CEYA----------------------------------IELH-EDLLEDDDLDFG-------VVC--L--------------CG--HTF-----CWSCTL---ESHRPVTCNDASVWFS------STLD--TLK-------SNAWLLENTKR-------------CPN--C 73
Bra018266-HCa CPIC--------------------------------------------HGKEVS--------VLV--VP-------------CDNGHIA-----CPT--C---------------------CN---KLRN--K---------------------------------CPS--C 37
Bra018428-HCa CKMC-------------------------------------------NDPPEA---------PVI--TL-------------CR--HLSL----CKE--C--------------------DV---------FTGF-------------------------------CPV--C 35
Bra018847-HCa CRAC-------------------------------------------LDTALD---------PVV--TL-------------CG--HIF-----CYQ--C---------------VS---EY---------ITGD-E---NS------------------------CPV--C 39
Bra019048-HCa CNIC--------------------------------------------FE--ERRN----CFFVP-----------------CG--HLF-----CWP--C----------------IYK--------WL---HVQLS---SLSTDHHRRNN---------------CPV--C 49
Bra019242-HCa CVIC----------------------------------------------MGRKR------GAAF--IP-------------CG--HSAT----CKG--C-------------------------------ALRI-------MSEENKV-----------------CPI--C 40
Bra019391-HCa CCVC-------------------------------------------LETCTK----------MV--LPN------------CG--HTF-----CRV--C---------------------------SRELWLNR-------GS----------------------CPL--C 38
Bra019817-HCa CGIC--------------------------------------------NAREVS--------VLL--VP-------------CC--HSM-----CIK--C----------------YR--------NWN---LKS-------QS----------------------CPF--C 38
Bra019910-HCa CRMC-------------------------------------------LERKCTVAA-------DG-----------------CR--HLSL----CKE--C--------------------DV---------FTGV-------------------------------CPV--C 35
Bra020414-HCa CAVC-------------------------------------------MCPFTEEMS----TK--------------------CG--HEF-----CTN--C-------------ALYLSTTNITSSKT-SQATPGS-------VP----------------------CPL--C 51
Bra020415-HCa CAVC-------------------------------------------LERKCTVAA-------DG-----------------CG--HEF-----CTN--W-------------ELYLSTTNRTSSKT-PQATPGS-------DP----------------------CPL--C 51
Bra020684-HCa CPIC-------------------------------------------MTEAKDT---------AV--LP-------------CG--HIF-----CKG--C------------------------IK----MAISK------QGK----------------------CPT--C 38
Bra020773-HCa CVIC--------------------------------------------LSL-FNSA-------VS--LT-------------CR--HLCM----CSD--C---------------------AK---ELRL--QSN-------K-----------------------CPI--C 39
Bra020883-HCa CPIC-------------------------------------------KRHIEKNQG--------------------------CN--HVF-----CNA--C----------------IVK--------SM---KVD-------AT----------------------CPV--C 36
Bra021742b-HCa CPRC-------------------------------------------CHALTNPIF----Q---------------------CN--HMT-----CSAP-C----------------------------RF--QF--------------------------------CWI--C 32
Bra021889-HCa CPVC-------------------------------------------LGLIPKNA------VIKV--LPN------------CK--HMF-----YEE--C----------------IAR--------WL---ESH-------TT----------------------CPV--C 42
Bra022858-HCa CPVC-------------------------------------------DETQVEA---------VL--YM-------------CDNGHIA-----CSA--C---------------------CI---NMRN--K---------------------------------CPS--C 37
Bra023148-HCa CCVC--------------------------------------------MKDEVS--------VVF--LP-------------CG--HMCT----CLK--C---------------------AN---ELHW--SGG-------K-----------------------CPI--C 39
Bra023274-HCa CIIC-------------------------------------------RTREATV---------VM--LP-------------CA--HQVV----CGS--C---------------------SD---NFFS--SNN-------GGGGKVT-----------------CPC--C 45
Bra023282-HCa CKGC-------------------------------------------LSNPKD----------MA--FG-------------CR--HLSI----CPE--C--------------------------------DRT-------ALA---------------------CPL--C 34
Bra023465-HCa CPIC-------------------------------------------LDQVRD---------PVV--TL-------------CG--HQT-----CCE--C---------------------------------GP-------D-LQV-------------------CPI--C 34
Bra024213-HCa CNIC-------------------------------------------LTKPKD----------MA--FS-------------CG--HLF-----CWP--C------------------------IYKWTH--CTNNTRQRVDQYDKKESSK---------------CPV--C 52
Bra024254-HCa CPIC-------------------------------------------WRRSATV---------LA--LP-------------CG--HTT-----CKE--C---------------------------------GV------VVKT---------------------CPM--C 34
Bra024967-HCa CRIC-------------------------------------------YEPLTVPIF----Q---------------------CR--HLIL----CKG--C--------------------------------DG--------SVR------V--------------CPL--C 34
Bra025434-HCa CPIC-------------------------------------------LKLLYE---------PAT--TP-------------CDNGHLA-----CSS--C--------------------------------CPK------LSNK---------------------CHA--C 37
Bra025676-HCa CTVC-------------------------------------------FDLAQD---------PIV--TL-------------CG--HTF-----CRS--C----------------------------LFQSMD-------RGNK---------------------CPL--C 38
Bra025727-HCa CNIC-------------------------------------------LDQVRD---------PVV--TL-------------CG--HLF-----CWP--C----------------LYK--------WL---HLH-------STSSE-------------------CPV--C 41
Bra026271-HCa CNIC-------------------------------------------TCSMYPPIH----Q---------------------CG--HLF-----CWP--C------------------------IYKWTY--STNNTRRRVDQYDKKESPK---------------CPV--C 52
Bra026301-HCa CPVC-------------------------------------------LDTAHD---------PVV--TL-------------CHNGHTL-----CST--C-----------------KV--------RV--HNR--------------------------------CPT--C 37
Bra026346-HCa CNIC-------------------------------------------LETCTK----------MV--LPN------------CG--HLF-----CWP--C----------------IYK--------WL---HVQLT---SVSIDQHHSN----------------CPV--C 48
Bra026920-HCa CGIC--------------------------------------------LGIIK-------KTRT--VME-------------CC--HSM-----CIK--C----------------YR--------NWN---LKS-------QS----------------------CPF--C 39
Bra027538-HCa CPIC--------------------------------------------GERTAS--------VLV--LP-------------CL--HRF-----CRE--C----------------------------IDKSMR-------LGNKE--------------------CPA--C 39
Bra027588-HCa CKRC----------------------------------------------LEHNY------NAVF--VP-------------CR--HLCL----CTV--C---------------------------------G--------SALLQA------------------CPV--C 36
Bra027667-HCa CVIC-------------------------------------------FEAFTIPIF----Q---------------------CG--HMCC----CTA--C-----------------S--------SHL---TS--------------------------------CPL--C 34
Bra028153-HCa CPIC--------------------------------------------GERTAS--------VLV--LP-------------CENGHLA-----CSS--C--------------------------------CPK------ISNK---------------------CPT--C 37
Bra028364-HCa CKRC-------------------------------------------CEPLKAPIF----Q---------------------CR--HLCL----CTV--C---------------------------------G--------SALLQS------------------CPV--C 35
Bra028395-HCa CPIC-------------------------------------------VDASAEV---------AC--VP-------------CDNGHLA-----CSV--C---------------------CT---KVRN--I---------------------------------CPS--C 37
Bra028419-HCa CVIC--------------------------------DDYAIKRHDEVPSEDFDV---------VC--L--------------CG--HVAG----CIS--C----------------LK--------EIKN--KKL-------G-----------------------CPI--C 49
Bra028646-Hca CPDC-------------------------------------------LSELRDT---------IV--LP-------------CG--HIF-----CWS--CQLESHRPVTCNNASFWLN--------ELLY--KSR-------NLARTAKRIKH-------------CPE--C 64
Bra028846-HCa CVVC----------------------------------------------HVYIK------GATF--AP-------------CR--HMCM----CSG--C---------------------AK---ALRF--QKN-------E-----------------------CPV--C 39
Bra029623-HCa CCVC-------------------------------------------MCPFTEEVS----TK--------------------CG--HTF-----CKL--C---------------------------SKELMAQK-------GH----------------------CPV--C 38
Bra029631-HCa CPIC-------------------------------------------LSEPRDT---------TV--LP-------------CG--HIF-----CKK--C------------------------IK----LAVSV------QAK----------------------CPT--C 38
Bra029788-HCa CVIC-------------------------------------------CDTDIDA---------LL--YR-------------CR--HMCM----CSG--C---------------------AK---VLRF--QTN-------R-----------------------CPI--C 39
Bra029846-HCa CCVC-------------------------------------------HEPAED---------NVV--TS-------------CG--HMCT----CSN--C---------------------GN---ELVR--TGG-------K-----------------------CPL--C 39
Bra030604-HCa CGLC-------------------------------------------FDRPKE---------VVI--MK-------------CE--HVF-----CKA--C-----------------LI-------DFAA--SLG-------EVS---------------------CPT--C 40
Bra030874-HCa CGVC----------------------------------------------VTRRR------IPAF--IP-------------CF--HLF-----CKQ--C-----------------IQ-------RSLE---IR-------HRK---------------------CPG--C 39
Bra030900-HCa CVIC-------------------------------------------FEKEISL---------VL--LP-------------CG--HVV-----CCRY-C----------------------------ALTVERG-------LNPK--------------------CPV--C 40
Bra031030-HCa CRVC-------------------------------------------LKLLYD---------PAT--TP-------------CR--HRVL----CRI--C--------------------------------SDK------CTK----------------------CPI--C 35
Bra031038-HCa CTVC-------------------------------------------FESPTAT---------IL--LP-------------CG--HTF-----CRS--C----------------------------LFQSMD-------RGNK---------------------CPL--C 38
```

```
Bra031184-HCa  CKVC---------------------------------------------YAGDA------NAVL--VP-------------CR--HFCL----CKC--C------------------------S------LA-------CSE-------------------CPI--C 35
Bra031225-HCa  CCIC------------------------------------------KVTLTNTMS----LVALS--S--------------CS--HRS-----CYG--C---------------IT-------RHL----LN-----------------------------CQR--C 39
Bra031418-HCa  CPSC------------------------------------------KVTLTNTMS----LVALS--S--------------CG--HVF-----CKK--C--------------------------GEKFMP-------VDKV-------------------CLV--C 42
Bra031420-HCa  CPSC-------------------------------------------NVKEVS--------VLL--VP-------------CG--HVF-----CKK--C--------------------------GEKFMP-------VDKV-------------------CLV--C 38
Bra031495-HCa  CKSC------------------------------------------LTNPKD----------MA--FS-------------CR--HLSL----CKD--C------------------DV---------FTGF-----------------------------CPV--C 34
Bra031942-HCa  CPIC------------------------------------------FHPLTIPIF----Q---------------------CG--HTT-----CKE--C-------------------------------GV------VVKI-------------------CPM--C 33
Bra032048-HCa  CPIC------------------------------------------FELAQD---------PIV--TL-------------CDNGHIV-----CST--C------------------------------CEK------LSQK-------------------CAT--C 37
Bra032145-HCa  CNIC------------------------------------------LKLLNQ---------PVL--LP-------------CG--HLF-----CWP--C--------------LYR--------WL---HHH-------SHSQE-----------------CPV--C 41
Bra032532-HCa  CPLC------------------------------------------LDTVFDP---------IS--LT-------------CD--HIF-----CDS--C---------------------------IHESSQ-------VESG------------------CPV--C 38
Bra032581-HCa  CSIC------------------------------------------LVKTKN----------MA--FN-------------CG--HIY-----CYM--C----------ACSAASV-------NVVD--GLK-------AADPSEK---------------CPL--C 48
Bra032793-HCa  CPIC---------------------------------------------------L---EYPLCPQITS------------CG--HQT-----CDE--C-------------------------------G--------EAIQT-----------------CPI--C 34
Bra032944-HCa  CPIC------------------------------------------LEICFEP---------ST--TT-------------CG--HIF-----CFP--C------------------------VLQYLLIG--------EDNHKAECFKR-----------CPL--C 47
Bra033002-HCa  CAIC--------------------------------------------NKSML---------LW--ANFP-----------CG--HSF-----CKK--C-------------------------LR--SAADKC---GRK---------------------CPK--C 39
Bra033065-HCa  CFWC------------------------------------------WESFNIV---------EN--VPYVLW---------CR--HKLW----CSS--C---------------KH-------KIAE--SAG-------DHHR------------------CVI--C 46
Bra033093-HCa  CPIC------------------------------------------RESFVD---------PVV--TK-------------CG--HTM-----CKN--C-------ILGLQWAIV--------KLPT--HPV-------QLPLFIS---------------CPW--C 51
Bra033268-HCa  CFIC------------------------------------------LDTVFDP---------IS--LT-------------CK--HYF-----CEH--C---------------A--------LKHH--TK--------NKK-------------------CFV--C 38
Bra033353-HCa  CSIC-------------------------------------------LSEEMS--------VIF--LP-------------CG--HIY-----CYM--C----------ACSAASV-------NVVD--GLK-------TADPSEK---------------CPL--C 49
Bra033393-HCa  CVMC------------------------------------------FESPTAA---------FL--PP-------------CA--HQVL----CFT--C----------------------------------------NQLHEKEGMKD-----------CPS--C 40
Bra033597-HCa  CKVC------------------------------------------LESADD---------PIL--TP-------------CR--HFCL----CKS--C------------------------S------LA-------CSE-------------------CPI--C 35
Bra033682-HCa  CPIC-------------------------------------------LGIIK-------KTRT--VME-------------CA--HRM-----CRE--C-------------LLT--------SWR---STS-------CGL-------------------CPI--C 41
Bra033732-HCa  CPIC---------------------------------------------WTGF---SSTRG----ILP-------------CL--HRF-----CRE--C--------------------------IDKSMR-------LGNNE------------------CPA--C 40
Bra033848-HCa  CPIC------------------------------------------LTIRKD----------VA--FG-------------CG--LMI-----CRD--C------------------------GSRI--SN------------------------------CPI--C 33
Bra034017-HCa  CVIC------------------------------------------LSEPSDT---------TV--LP-------------CG--HRF-----CYS--C---------------IQ-------QWVD--RLV-------SERKKTT---------------CPL--C 44
Bra034086-HCa  CVIC-------------------------------------------DADIDA---------LL--YR-------------CR--HMCM----CSG--C-------------------AK---VLRF--QTN-------R---------------------CPI--C 38
Bra034121-HCa  CVCC------------------------------------------LDSVQE---------PVV--TL-------------CG--HMCT----CSK--C-------------------GY---ELVR--TGG-------K---------------------CPL--C 39
Bra034212-HCa  CNIC---------------------------------------------MGREK------GAAF--IP-------------CG--HLF-----CWP--C---------IHKWLHVQ--------TD---EHQRQ---HKQ---------------------CPV--C 46
Bra034501-HCa  CCVC-------------------------------------------GKGEAS--------VLL--LP-------------CG--HTY-----CRV--C-------------------------SREIWMNR-------GS--------------------CPL--C 38
Bra034718-HCa  CRGC-------------------------------------------GEEESC--------VLV--LP-------------CR--HMCL----CTV--C-------------------------------G--------SSVNT-----------------CPV--C 35
Bra035092-HCa  CRSC------------------------------------------LTNRKD----------VA--FG-------------CR--HLCL----CGV--C-------------------------------G--------SSVHT-----------------CPI--C 34
Bra035112-HCa  CPIC------------------------------------------FEQVCTIE-----VKD-------------------CG--HMT-----CGE--C------------------------GSRI--SN------------------------------CPI--C 34
Bra035664-HCa  CCIC------------------------------------------MCPFTEEMS----TK--------------------CG--HQM-----CAQ--C-------------TLA--------LCCH--NKP-------NLTTSTVNPPV-----------CPF--C 49
Bra036164-HCa  CPIC-------------------------------------------LSEEMS--------VIF--LP-------------CG--HIF-----CKG--C----------------------IK----MAISR------QNK--------------------CPT--C 38
Bra036227-HCa  CVMC------------------------------------------ELPFLD---------PVV--TT-------------CA--HQVL----CSK--C----------------------------------------NQLHEKEAMDD-----------CPS--C 40
Bra036439-HCa  CPFC------------------------------------------MCPFTEETS----TK--------------------CD--HYF-----CNL--C---------------A--------RKHH--KK--------DPT-------------------CFV--C 38
Bra037471-HCa  CPIC---------------------------------------------VTRRR------VPAF--IP-------------CG--HIF-----CKG--C----------------------IK----TAISR------QAK--------------------CPT--C 38
Bra037967-HCa  CVIC------------------------------------------FENPVNV---------VL--LP-------------CG--HVV-----CCRQ-C--------------------------ASTVERE-------VNPK------------------CPV--C 40
Bra038276-HCa  CEYA---------------------------------IELH-EDLLEDDDLDFG-------VVC--L--------------CG--HTF-----CWSCTL---ESHRPVTCKEASVWSS-----STLD--TLK-------SNAWLLENTKR-----------CPN--C 73
Bra038348-HCa  CKVC------------------------------------------LERVLSKPTP---AERKFGLLTE------------CK--HYVL----CST--C------------------------------CKK------CKT--------------------CPI--C 44
Bra038415-HCa  CCVC------------------------------------------RKVMSS---------PVT--TP-------------CD--HAF-----CIG--C--------------IRN--------WR---SSSPS---TGMDVNSTLRA-------------CPI--C 49
Bra038515a-HCa CQIC------------------------------------------LQLPER---------PVT--TP-------------CA--HNF-----CKE--C---LTGKFAGITQVRQRSRGGR--TLRA--QKN-------VMK-------------------CPC--C 57
Bra038515b-HCa CSIC----------------------------------------SYFIEFHKG------TDAEEYGLNVVCL---------CG--HNF-----CLK--C-------------------FQ---KWVA--GKR-------KPT-------------------CGT--C 51
Bra038659-HCa  CPGC------------------------------------------LSNPKN----------MA--FG-------------CG--HNF-----CCR--C-----------SLETHRPVTCN--NASDWLSRDLQKL--SEASDKSLSFLWIEDNTET----CPH--C 70
Bra039105-HCa  CPIC-------------------------------------------GKGEAS--------VLL--LP-------------CG--HQT-----CCE--C-------------------------------GP-------G-LKV-----------------CPI--C 34
Bra039354-HCa  CRSC----------------------------------------LGPILQESY------LDT-------------------CR--HMCL----CSV--C-------------------------------G--------SSINT-----------------CPI--C 36
Bra039449-HCa  CPIC-------------------------------------------GERRAS--------VLV--LP-------------CF--HKF-----CFR--C----------------------IKQWIKVVSSK------VSKPLSSVK--------------CPL--C 48
Bra039533-HCa  CRRC------------------------------------------DETQVEA---------VL--YR-------------CR--HLCL----CTV--C-------------------------------G--------SALLQA----------------CPV--C 36
Bra039871-HCa  CCVC------------------------------------------LDRVLSKATP---GERKFGLLTE------------CG--HMCT----CLK--C-------------------AN---ELHW--SGG-------K---------------------CPI--C 48
Bra040048-HCa  CSVC-------------------------------------------GVGEVC--------MLL--LP-------------CH--HPF-----CIQ--C--------------IRN--------WR---SSAPV---SGMDVNSTLRA-------------CPI--C 49
Bra040255-HCa  CRFC------------------------------------------NDRSKE---------VVI--TK-------------CK--HMCL----CKE--C------------------------------ERN-------LSS-------------------CPL--C 35
Bra040332-HCa  CKAC---------------------------------------------QVNIK------GATF--TP-------------CF--HLF-----CNP--C-----------------VLK---------ITGTRQ---RK----------------------CPT--C 39
Bra040362-HCa  CCVC---------------------------------------------LERKC------DAAF--VP-------------CG--HTF-----CKL--C-------------------------SKELSAQK-------GH--------------------CPV--C 38
Bra040424-HCa  CVVC----------------------------------------IDDNISAIQM--------FS--VDI------------CG--HMC-----CCLT-C---------------A--------LKLL--GKP-----------------------------CPL--C 41
Bra000504a-HCb CSIC---------------------------------------MDEKS-PSD-I--------FR--GTTS-----------CG--HRF-----CSE--C----------VKRYIEAK----------LLEGN-------RLT-------------------CPHDGC 50
Bra001543-HCb  CMIC------------------------------------------NDPPEN---------PVI--SL-------------CT--HYY-----CTE--C----------TVRYVMTK----------IEGNI-------AMIK------------------CPDVDC 47
Bra001771-HCb  CNIC------------------------------------------VEDVEG-------HHHMT-RMD-------------CG--HVF-----CYQ--C-------------VS---EH---------INGD-E---NV----------------------CPVRRC 44
Bra001960-HCb  CDIC----------------------------------------FN-DIDPERM--------FS--IGK------------CG--HCF-----CNN--C--------------------WA--AHFTVKINE-------GQSKRIR---------------CMAYKC 51
Bra002501a-HCb CLIC------------------------------------------QHMIELSQG----CNHIT--CR-------------CS--HRF-----CFQ--C----------VKQHVEVK----------LLHGM-------IPN-------------------CPHDKC 51
Bra002501b-HCb CGKC----------------------------------------FN-DIDPERM--------FS--IGK------------CG--HEF-----CYN--C--------------GGGWNK---------KTGT-----------------------------CVKQ-C 42
Bra002502a-HCb CLIC------------------------------------------QHMIELSQG----CNHIT--CR-------------CS--HRF-----CFQ--C----------VKQHVEVK----------LLHGM-------IPN-------------------CPHDKC 51
Bra002502b-HCb CGKC------------------------------------------FESYLRKEI-------AT--VS-------------CG--HEF-----CYN--C--------------GGGWNK---------ITGT-----------------------------CAKQ-C 41
Bra005642-HCb  CGIC------------------------------------------LCEVDDG------YSLEG-----------------CG--HPY-----CKT--C--------------------WTSYITEKINNGP-------GCLMIK----------------CPEPSC 50
Bra006057-HCb  CPIC------------------------------------------FDYFCRI---------DI--VPIM-----------CS--HLF-----CKA--C--------------LL--------EQL---EAS-------MRNFDAFPIL------------CSHTDC 50
Bra006112a-HCb CGIC----------------------------------------YN-ETDIERM--------FS--VAK------------CG--HAF-----CST--C--------------------WTSYISIAINDGP-------GCLMQK----------------CPEPSC 52
Bra006671-HCb  CLIC-------------------------------------------YAHVSPRH----KLEVG--SG-------------CR--HRF-----CFH--C----------SKQHVEVK----------LLHGT-------VPN-------------------CPHDGC 50
Bra007078-HCb  CPAC----------------------------------------LE-ETDAGRM--------FS--TEQ------------CF--HRI-----CFT--C-------------------IRDCVSSQ--LARGDT---LL----------------------CPYPGC 49
Bra007801-HCb  CAIC------------------------------------------LSEVEDG------YSLEG-----------------CL--HRH-----CFS--C----------VKQYVEVK----------LLSGI-------VPT-------------------CLGEGC 47
Bra009038-HCb  CPIC----------------------------------------FSESAGFDF---------VK--LP-------------CS--HLF-----CKA--C--------------LL--------EQL---EAS-------MRNFDAFPII------------CSHIDC 50
Bra010152-HCb  CCIC----------------------------------------LE-DVVAEQM--------KR--MD-------------CE--HFF-----CVK--C----------MKTYTDIH----------VSEGT------VNKLQ------------------CPDSKC 50
Bra011524-HCb  CDVC----------------------------------------LDEDINADQM--------FS--VDK------------CG--HCF-----CND--C--------------------WT--EHFTVQINE-------GQSKRIR---------------CMAHKC 52
Bra011996a-HCb CAIC----------------------------------------CE-DRRAEMM--------LT--LK-------------CG--HWF-----CSE--C----------VKRHIEVK----------LLEQG-------FVR-------------------CPQHRC 48
Bra012536-HCb  CSIC----------------------------------------CE-DRLSEMM--------LT--LK-------------CT--HKS-----CSY--C----------MKTYVQGR----------VE-SS------EVPIR------------------CPQVQC 49
Bra013417-HCb  CSIC---------------------------------------------------G---EWCRLPVITP------------CT--HKF-----CSH--C----------MKTYVEGK----------VN-SS------EVPIR------------------CPQLQC 47
Bra014815-HCb  CKRC----------------------------------------FDTDFNAEQM--------FC--VAS------------CR--HLL-----CLD--C--------------------------VS---LD-------SER-------------------CTFPGC 41
Bra015067-HCb  CGIC------------------------------------------FKLLSLERS-------AS--VS-------------CG--HEF-----CVE--C----------VKRHIEVR----------VFEGD-------VHIRCP----------------CYYY-C 50
Bra015460-HCb  CGIC------------------------------------------FEPFQRE------E-VVS--LA-------------CG--HRV-----CKF--C-------------------------WRSHINKSIN---KIADVDWYGTLK------------CPYDIC 54
Bra015463-HCb  CGIC-------------------------------------------YTHVSRPH----KLEVR--SG-------------CG--HPF-----CST--C--------------------WTGYISTTINDGP-------GCLMLK----------------CPEPSC 53
Bra018584-HCb  CPAC----------------------------------------LD-DVDSDLM--------FY--VER------------CF--HLI-----CFT--C-------------------IRDCVSSQ--LARGDT---VL----------------------CPYPGC 49
Bra020455a-HCb CIIC----------------------------------------LD-DVDSDLM--------FY--VER------------CG--HRF-----CIN--C----------VKQHINVK----------LVDGK-------IPN-------------------CPHHGC 49
Bra020457-HCb  CKIC------------------------------------------FDPYPPEKI-------AS--VS-------------CG--HRF-----CIN--C----------VKQHINVK----------LVDGK-------IPN-------------------CPHHGC 48
Bra021726-HCb  CGIC------------------------------------------FETYLSD-------KLHA--AA-------------CG--HPF-----CTT--C--------------------WTGYISTTINDGP-------GCLMLR----------------CPDPSC 51
Bra021742a-HCb CGIC------------------------------------------QNMIELSEG----CATVQ--CR-------------CG--HPY-----CMT--C--------------------WNGYITAKITGGP-------GCLMVT----------------CPEPSC 54
Bra022479-HCb  CGIC------------------------------------------FDSYPPEKF-------VS--VS-------------CG--HPF-----CDS--C--------------------WEGYISTAINDGP-------G---------------------CLMLRC 46
Bra022845-HCb  CGIC------------------------------------------FDSYTQKEV-------TT--LS-------------CG--HPF-----CTT--C--------------------WTGYITTTINDGP-------GCLMLR----------------CPDPSC 51
Bra022865a-HCb CGIC------------------------------------------FDKFRRK---------KI--VPVV-----------CG--HPY-----CNV--C--------------------WTGYITTTINDGP-------G---------------------CLTVKC 46
Bra023338-HCb  CGIC----------------------------------------LDDDVNADQM--------FT--VNK------------CG--HTF-----CST--C--------------------WNGYITTAINDGS-------GCLMQK----------------CPEPSC 53
Bra026648b-HCb CGKC------------------------------------------QHMIELSKG----CVLVV--CR-------------CG--HKF-----CYR--C----------------------GAN-ARSCTH------GLGHMFPPQPQELESPPPAPP---CWAQ-C 64
Bra026648a-HCb CAIC---------------------------------------MDEKP-SSD-M--------FR--GSVT-----------CG--HRF-----CSE--C----------VKRHIEVR----------LLEGS-------VMT-------------------CPQFRC 50
Bra027352-HCb  CMIC---------------------------------------MDEKS-PSD-M--------FR--GN-------------CT--HAY-----CTQ--C----------TIRYVASK----------IKENS-------ARIK------------------CPDVEC 49
Bra029725-HCb  CMIC------------------------------------------NDSPED---------AVV--SV-------------CT--HSY-----CTE--C----------TVRYVETK----------IGENV-------AGIK------------------CPDVDC 47
Bra031452-HCb  CGIC------------------------------------------MEDDLP-------SHAMT-GME-------------CG--HVF-----CKQ--C------------------FYEC-------FTGD-D---NL----------------------CPIANC 44
Bra033024-HCb  CDIC----------------------------------------FDDDLEAHQM--------FS--VAL------------CG--HSF-----CND--C--------------------WK--EHFTIKINE-------GQSKRIA---------------CMAHKC 52
Bra034292-HCb  CPIC------------------------------------------DDPPEN---------PVV--TL-------------CC--HQF-----CFE--C----------VRRYINVG----------LMEGR-------AFG-------------------CPHFKC 46
Bra035824-HCb  CNIC-------------------------------------------YAHVSPRH----KLEVR--SG-------------CG--HVF-----CYQ--C-------------VS---EH---------ITGD-E---NV----------------------CPVRRC 45
Bra037371-HCb  CPAC----------------------------------------YE-DVPADTK--------FT--VSG------------CF--HRI-----CFT--C-------------------IRDCVSSQ--LARGDS---VL----------------------CPYPGC 49
Bra037375-HCb  CVIC----------------------------------------YE-GITVDKM--------FS--VDG------------CF--HRI-----CFE--C--------------MR--------NYITHSLRH-------RSRLI-----------------CPNTGC 49
Bra038285a-HCb CVIC---------------------------------------MDEKS-PSD-I--------FR--GTTS-----------CF--HRF-----CFS--C----------MKQHVEIK----------LLGGK-------TAT-------------------CPSDGC 50
Bra038306-HCb  CLIC------------------------------------------NDSPED---------AVV--SV-------------CT--HSY-----CTE--C----------TVRYVTTK----------VEENAA-----RARIK------------------CPDMNC 49
Bra038573-HCb  CGIC------------------------------------------MDDDLQ-------SYTMT-RMD-------------CG--HVF-----CKQ--C------------------FYEC-------LTGD-N---NI----------------------CPNVNC 44
Bra039068-HCb  CEIC------------------------------------------FESYFQKDI-------AT--VS-------------CG--HCF-----CNN--C--------------------WK--EHFTVKINE-------GMSKRIT---------------CMAHKC 50
Bra000030-V    CRIC--------------------------------------------HLGVVET----SGGGA--IELG-----------CS--CKEDLAGAHRQ--CAGTWFKIKGDKLVAHRQCAE----TWFK--IKG-------DKI-------------------CEI--C 68
Bra001193-V    CRIC-------------------------------------------LDVLE-------EGNTL-KME-------------CS--CKGDLRLVHEA--C--------------AI--------KWFS--TKG-------TRT-------------------CDV--C 47
Bra001210-V    CRIC-----------------------------------------RSAEEPGNA--------LM--HP-------------CS--CRGSIKYVHHD--C--------------LL--------IWLN--RRG-------YKQ-------------------CEV--C 47
Bra002470-V    CRIC------------------------------------------LVDLCE-------GGETF-KME-------------CS--CKGELALAHKD--C--------------AL--------KWFT--IKG-------NKT-------------------CEV--C 48
Bra002600-V    CRIC----------------HLPLETTNKADEECEDSDEQEEQGEEEDEEEEEY--------YG--LPLQLG---------CS--CKGDLGVAHSK--C--------------AE--------TWFK--IKG-------NMT-------------------CEI--C 76
Bra005147-V    CRIC--------------------------------------------HLGVVET----SGGGA-MELG------------CS--CKEDLAIAHRQ--C--------------AE--------TWFK--IKG-------DKI-------------------CEI--C 50
Bra005444-V    CRIC-----------------------------------------HVGSDQTPDR-------VS--GKTVVSLELIQIG--CK--CKNELALAHFH--C--------------AE--------AWFK--LRG-------NSV-------------------CEI--C 59
Bra005697-V    CRIC---------------------------------------HLGLESSRREC-----GDPMV--LG-------------CS--CKDDLGYVHKQ--C--------------AE--------TWFK--IKG-------DKT-------------------CEI--C 52
Bra009138-V    CRIC------------------------------------------HMTLDATNL----ESGVG--IELG-----------CS--CKDDLAAAHKH--C--------------AE--------TWFK--IKG-------NKI-------------------CEV--C 52
Bra009367-V    CRVCQCAESDRRGDAALGFLGITPPVSEPRRSSNAKKQETTDHKTGGFIELISPDGEVFVCANEDIEMGAWQHRDTLLELGCS--CKNDLALVHYA--C--------------AL--------KWFV--NHG-------STV-------------------CEI--C 111
Bra010084-V    CRIC------------------------------------------QDEDSVKN--------LE--CP-------------CS--CSGSLKYAHRK--C--------------VQ--------RWCN--EKG-------DTT-------------------CEI--C 46
Bra011370-V    CRIC-----------------------------------------QSPEEPNNP--------LR--HP-------------CA--CRGSLKYVHTD--C--------------IFL--------WI---NRR-------RSKH------------------CEI--C 47
Bra011487-V    CRIC-----------------------------------------RNPGEADNP--------LR--YP-------------CA--CSGSIKFVHQD--C--------------LL--------QWLN--HSN-------ARQ-------------------CEV--C 47
Bra014329-V    CRIC------------------------------------------HDEDLDCN--------ME--TP-------------CS--CSGSLKYAHRR--C--------------VQ--------RWCN--EKG-------DTT-------------------CEI--C 46
Bra014845-V    CRIC---------------------------------------------KSEVG-----YGQGL--IELG-----------CS--CKGDLAFSHRQ--C--------------AE--------TWFK--LKG-------NQV-------------------CEI--C 48
Bra017626-V    CRIC-----------------------------------------RNPGDADNP--------LR--YP-------------CA--CSGSIKFVHED--C--------------LL--------QWLS--FSK-------ARH-------------------CEV--C 47
Bra018136-V    CRIC------------------------------------------QEEDITNN--------LE--TP-------------CA--CNGSLKYAHRK--C--------------VQ--------RWCN--EKG-------DII-------------------CEI--C 46
Bra018414-V    CRIC------------------------------------------LENDSDL---------LG--DELISP---------CM--CKGTQQFVHRS--C--------------LD--------HWR---SVK-------EGFAFSH---------------CTT--C 52
Bra019507-V    CRIC------------------------------------------QEEDTIQN--------LE--AP-------------CA--CNGSLKYAHRK--C--------------VQ--------RWCN--EKG-------DIT-------------------CEI--C 46
Bra020240-V    CRIC------------------------------------------LVDLCE-------GGETL-KME-------------CS--CKGELALAHKD--C--------------AL--------KWFT--IKG-------NKT-------------------CEV--C 48
Bra020758-V    CRIC-------------------------------------------LDECE-------EGNTL-KME-------------CS--CKGDLRLVHEA--C--------------AI--------KWFS--TKG-------TRT-------------------CDV--C 47
```

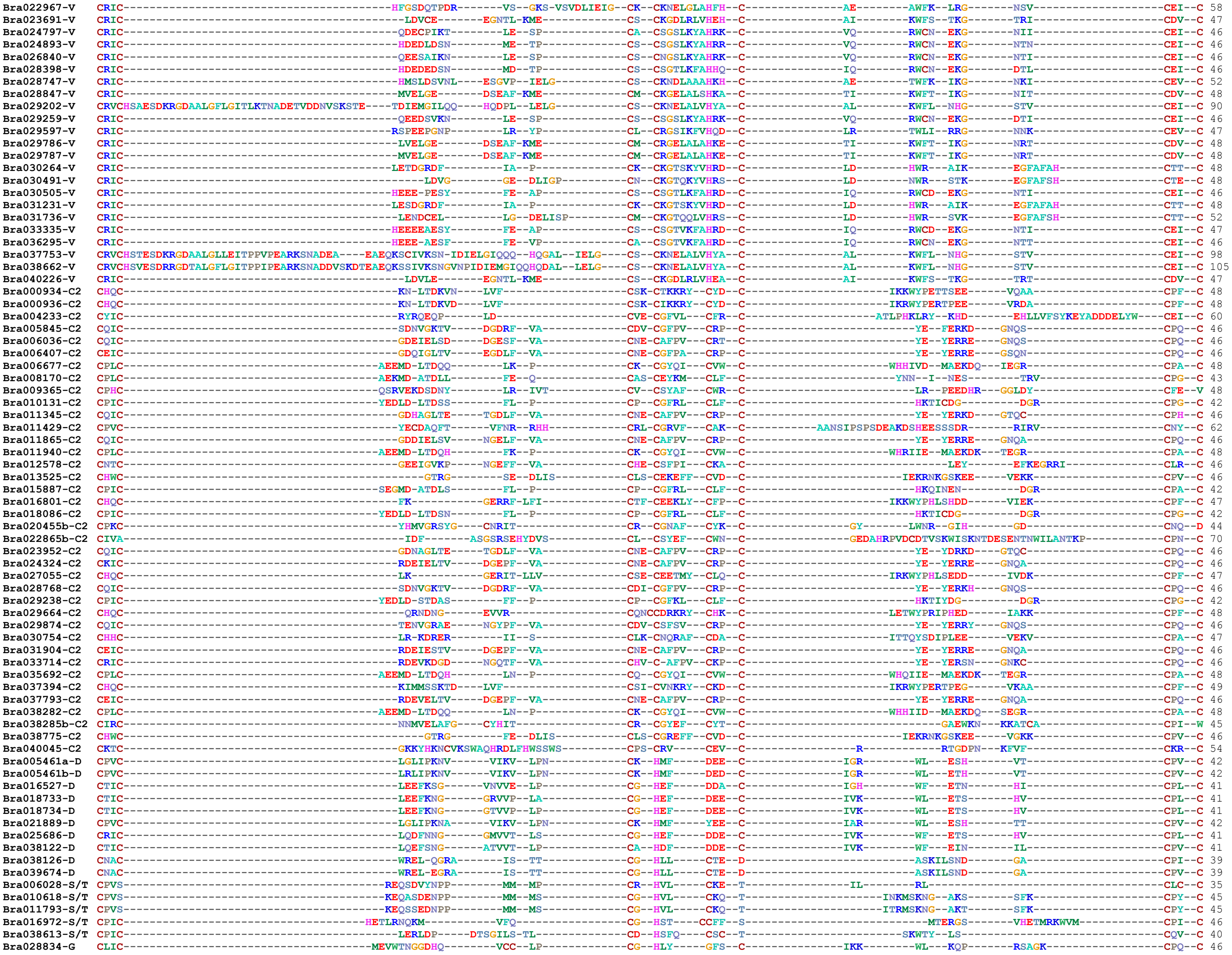

Figure S6 Phylogenetic tree based on multiple sequence alignment of 731 *B. rapa* RING domains. The percentage of replicate trees in which the associated taxa clustered together in the bootstrap test (1000 replicates) are shown next to the branches. The evolutionary distances were computed using the Poisson correction method and are in the units of the number of amino acid substitutions per site.

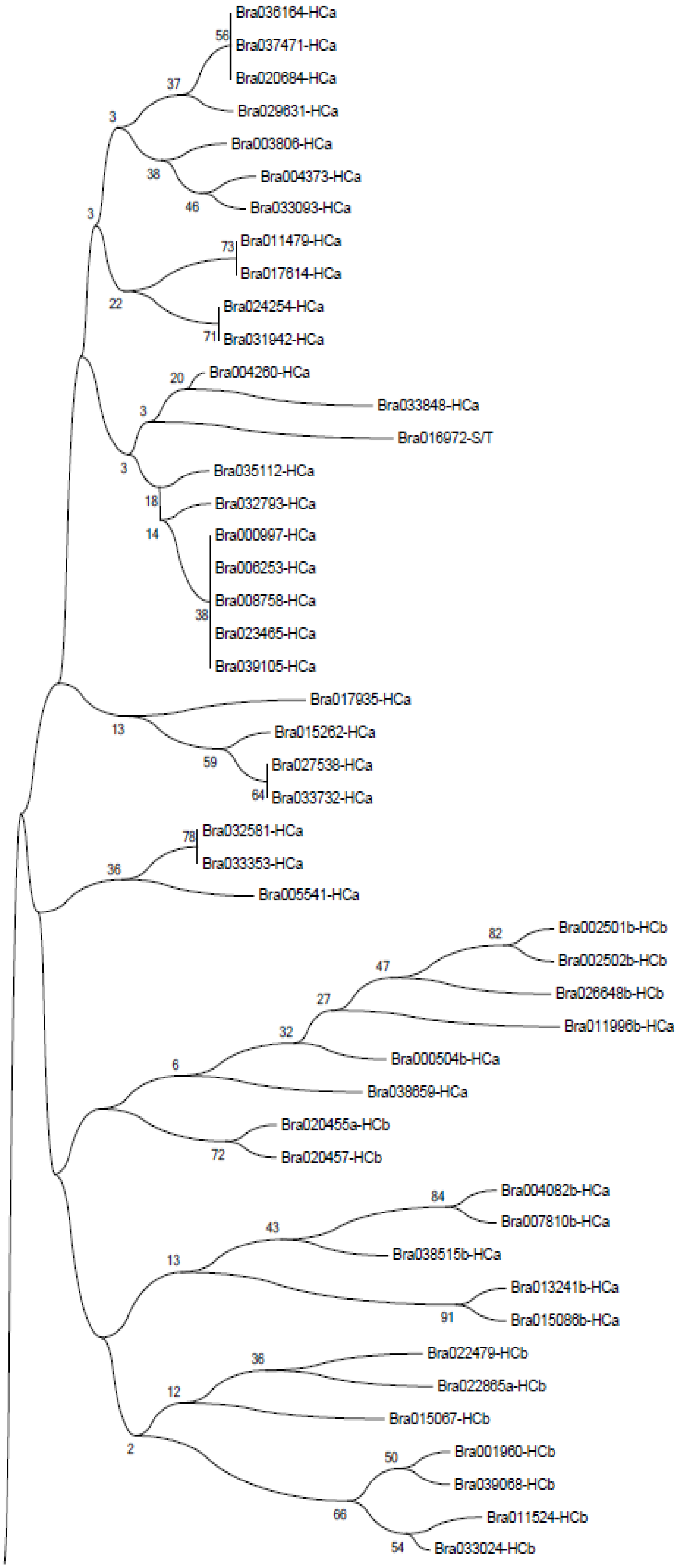
Bra036164-HCa
Bra037471-HCa
Bra020684-HCa
Bra029631-HCa
Bra003806-HCa
Bra004373-HCa
Bra033093-HCa
Bra011479-HCa
Bra017614-HCa
Bra024254-HCa
Bra031942-HCa
Bra004260-HCa
Bra033848-HCa
Bra016972-S/T
Bra035112-HCa
Bra032793-HCa
Bra000997-HCa
Bra006253-HCa
Bra008758-HCa
Bra023465-HCa
Bra039105-HCa
Bra017935-HCa
Bra015262-HCa
Bra027538-HCa
Bra033732-HCa
Bra032581-HCa
Bra033353-HCa
Bra005541-HCa
Bra002501b-HCb
Bra002502b-HCb
Bra026648b-HCb
Bra011996b-HCa
Bra000504b-HCa
Bra038659-HCa
Bra020455a-HCb
Bra020457-HCb
Bra004082b-HCa
Bra007810b-HCa
Bra038515b-HCa
Bra013241b-HCa
Bra015086b-HCa
Bra022479-HCb
Bra022865a-HCb
Bra015067-HCb
Bra001960-HCb
Bra039068-HCb
Bra011524-HCb
Bra033024-HCb

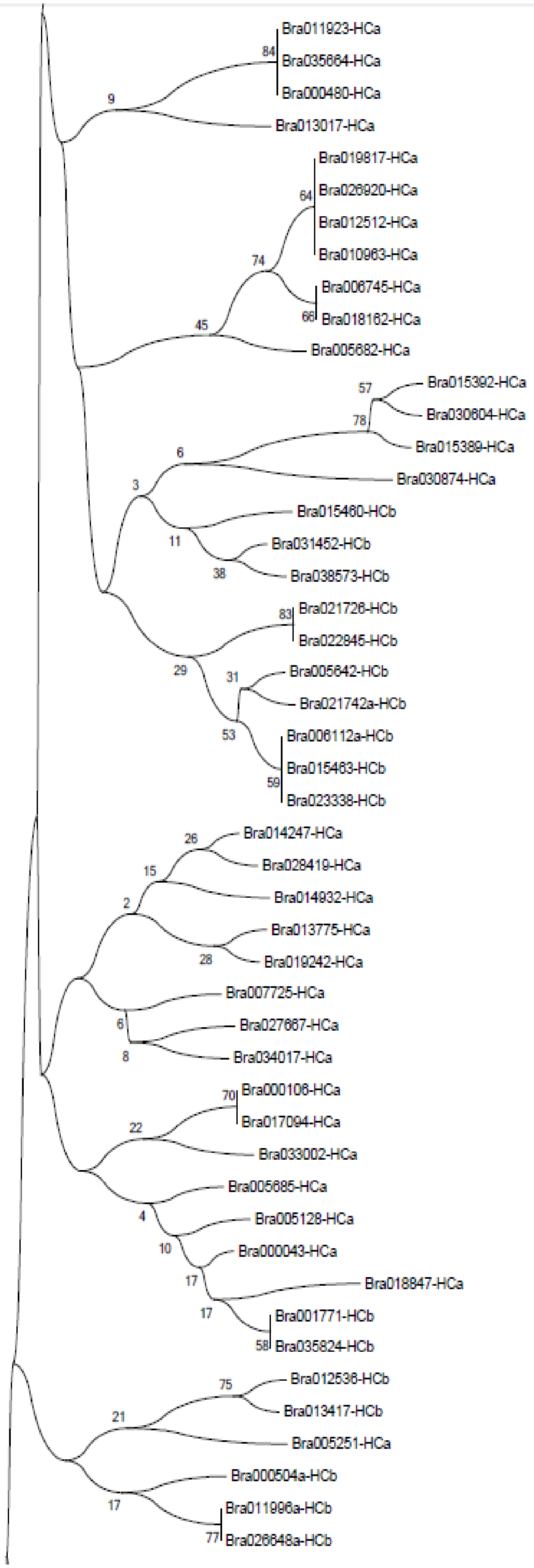
Bra011923-HCa
Bra035664-HCa
Bra000480-HCa
Bra013017-HCa
Bra019817-HCa
Bra026920-HCa
Bra012512-HCa
Bra010963-HCa
Bra006745-HCa
Bra018162-HCa
Bra005682-HCa
Bra015392-HCa
Bra030604-HCa
Bra015389-HCa
Bra030874-HCa
Bra015460-HCb
Bra031452-HCb
Bra038573-HCb
Bra021726-HCb
Bra022845-HCb
Bra005642-HCb
Bra021742a-HCb
Bra006112a-HCb
Bra015463-HCb
Bra023338-HCb
Bra014247-HCa
Bra028419-HCa
Bra014932-HCa
Bra013775-HCa
Bra019242-HCa
Bra007725-HCa
Bra027667-HCa
Bra034017-HCa
Bra000106-HCa
Bra017094-HCa
Bra033002-HCa
Bra005685-HCa
Bra005128-HCa
Bra000043-HCa
Bra018847-HCa
Bra001771-HCb
Bra035824-HCb
Bra012536-HCb
Bra013417-HCb
Bra005251-HCa
Bra000504a-HCb
Bra011996a-HCb
Bra026648a-HCb

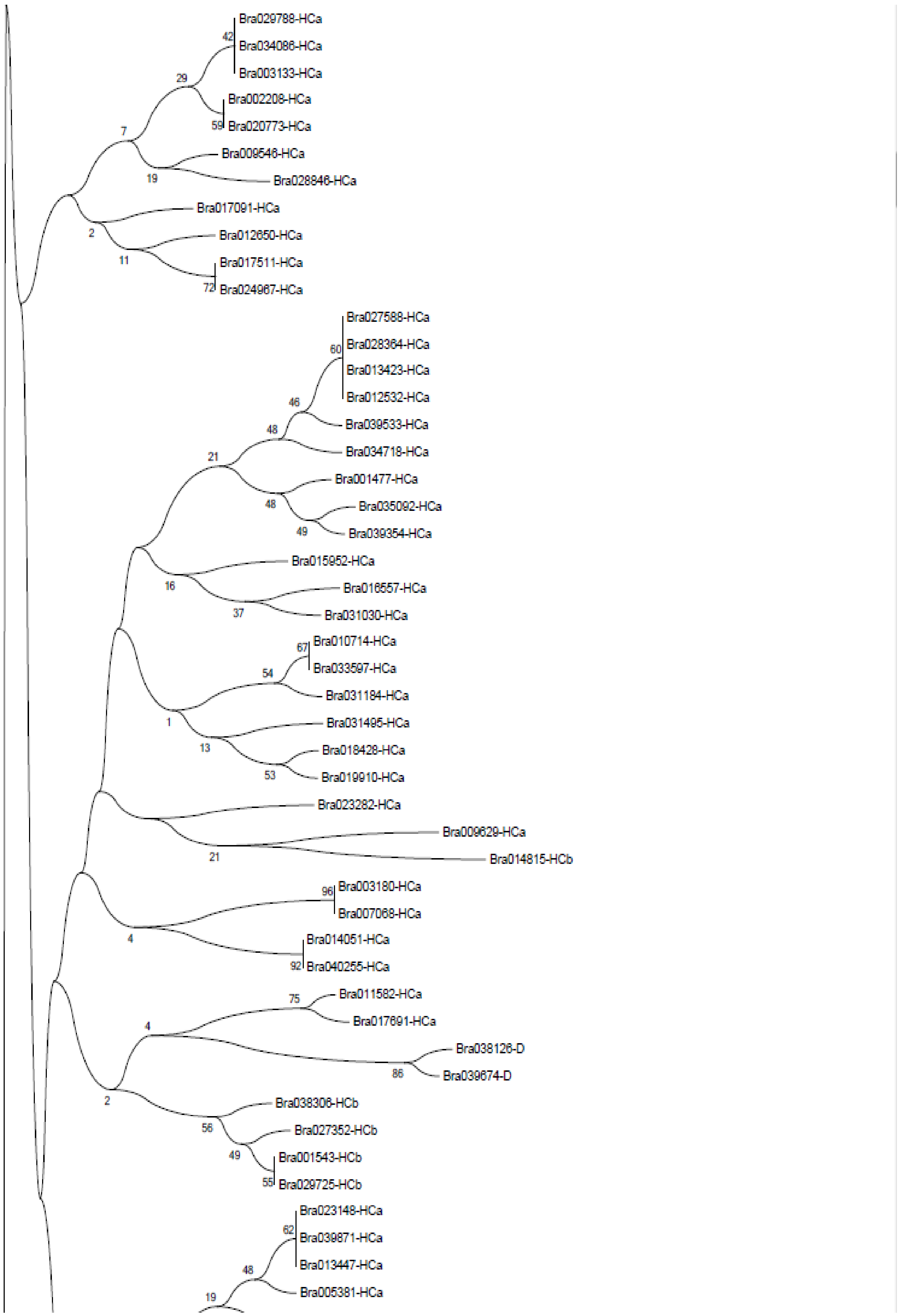
Bra029788-HCa
Bra034086-HCa
Bra003133-HCa
Bra002208-HCa
Bra020773-HCa
Bra009546-HCa
Bra028846-HCa
Bra017091-HCa
Bra012650-HCa
Bra017511-HCa
Bra024967-HCa
Bra027588-HCa
Bra028364-HCa
Bra013423-HCa
Bra012532-HCa
Bra039533-HCa
Bra034718-HCa
Bra001477-HCa
Bra035092-HCa
Bra039354-HCa
Bra015952-HCa
Bra016557-HCa
Bra031030-HCa
Bra010714-HCa
Bra033597-HCa
Bra031184-HCa
Bra031495-HCa
Bra018428-HCa
Bra019910-HCa
Bra023282-HCa
Bra009629-HCa
Bra014815-HCb
Bra003180-HCa
Bra007068-HCa
Bra014051-HCa
Bra040255-HCa
Bra011582-HCa
Bra017691-HCa
Bra038126-D
Bra039674-D
Bra038306-HCb
Bra027352-HCb
Bra001543-HCb
Bra029725-HCb
Bra023148-HCa
Bra039871-HCa
Bra013447-HCa
Bra005381-HCa
42
29
59
7
19
2
11
72
60
46
48
21
48
49
16
37
67
54
1
13
53
21
96
4
92
75
4
86
2
56
49
55
62
48
19

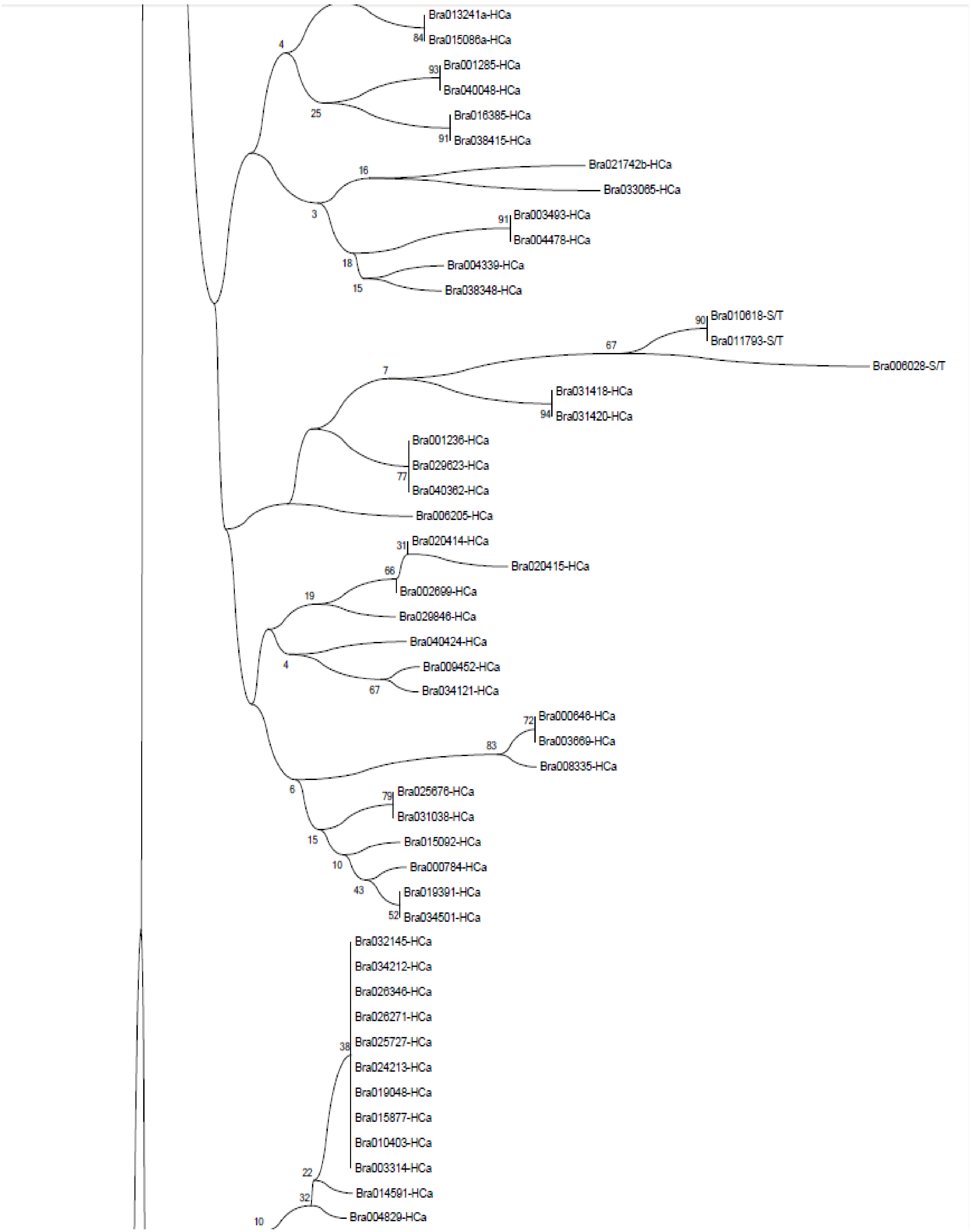
Bra013241a-HCa
Bra015086a-HCa
Bra001285-HCa
Bra040048-HCa
Bra016385-HCa
Bra038415-HCa
Bra021742b-HCa
Bra033065-HCa
Bra003493-HCa
Bra004478-HCa
Bra004339-HCa
Bra038348-HCa
Bra010618-S/T
Bra011793-S/T
Bra006028-S/T
Bra031418-HCa
Bra031420-HCa
Bra001236-HCa
Bra029623-HCa
Bra040362-HCa
Bra006205-HCa
Bra020414-HCa
Bra020415-HCa
Bra002699-HCa
Bra029846-HCa
Bra040424-HCa
Bra009452-HCa
Bra034121-HCa
Bra000646-HCa
Bra003669-HCa
Bra008335-HCa
Bra025676-HCa
Bra031038-HCa
Bra015092-HCa
Bra000784-HCa
Bra019391-HCa
Bra034501-HCa
Bra032145-HCa
Bra034212-HCa
Bra026346-HCa
Bra026271-HCa
Bra025727-HCa
Bra024213-HCa
Bra019048-HCa
Bra015877-HCa
Bra010403-HCa
Bra003314-HCa
Bra014591-HCa
Bra004829-HCa

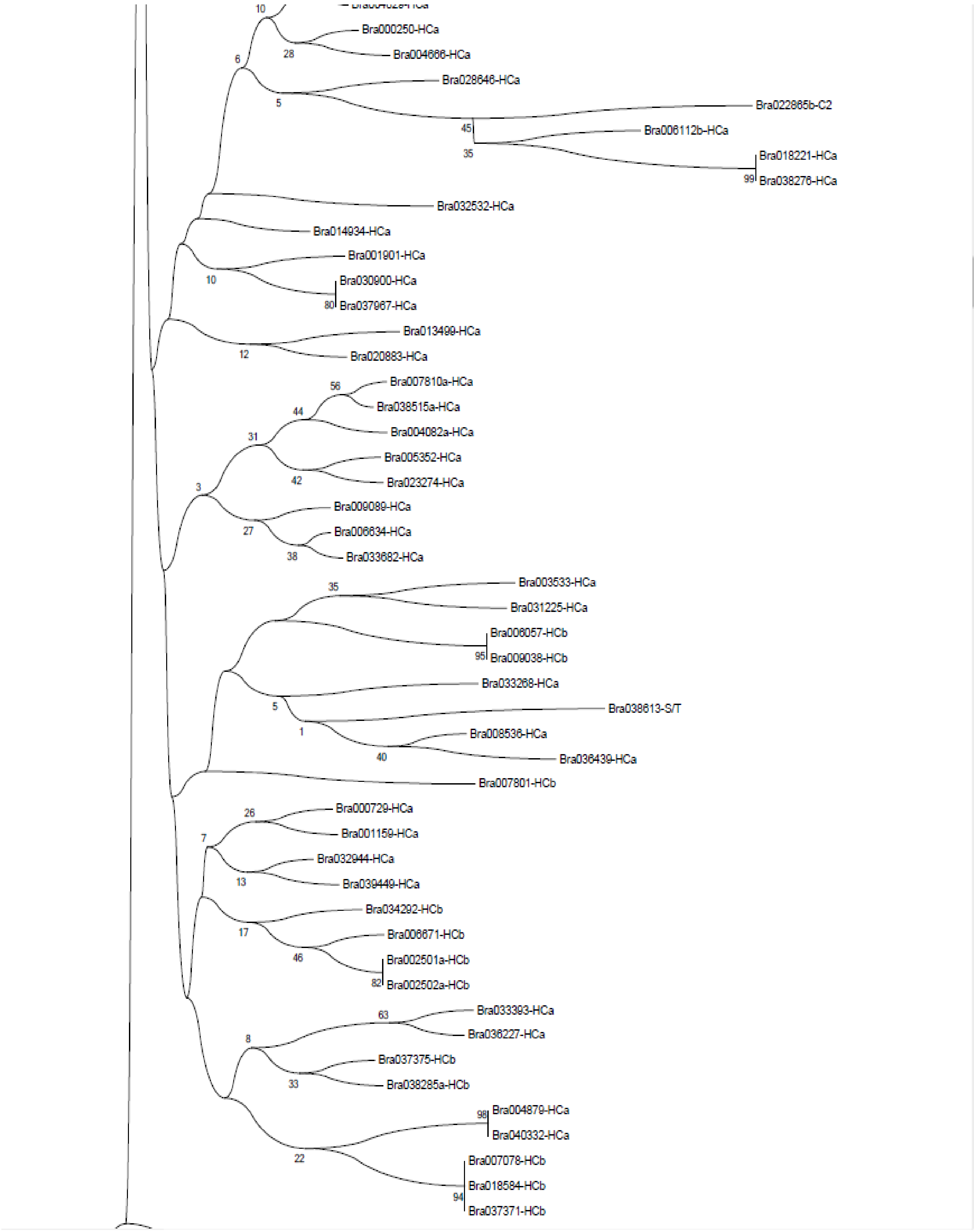

Bra000250-HCa
Bra004666-HCa
Bra028646-HCa
Bra022865b-C2
Bra006112b-HCa
Bra018221-HCa
Bra038276-HCa
Bra032532-HCa
Bra014934-HCa
Bra001901-HCa
Bra030900-HCa
Bra037967-HCa
Bra013499-HCa
Bra020883-HCa
Bra007810a-HCa
Bra038515a-HCa
Bra004082a-HCa
Bra005352-HCa
Bra023274-HCa
Bra009089-HCa
Bra006634-HCa
Bra033682-HCa
Bra003533-HCa
Bra031225-HCa
Bra006057-HCb
Bra009038-HCb
Bra033268-HCa
Bra038613-S/T
Bra008536-HCa
Bra036439-HCa
Bra007801-HCb
Bra000729-HCa
Bra001159-HCa
Bra032944-HCa
Bra039449-HCa
Bra034292-HCb
Bra006671-HCb
Bra002501a-HCb
Bra002502a-HCb
Bra033393-HCa
Bra036227-HCa
Bra037375-HCb
Bra038285a-HCb
Bra004879-HCa
Bra040332-HCa
Bra007078-HCb
Bra018584-HCb
Bra037371-HCb

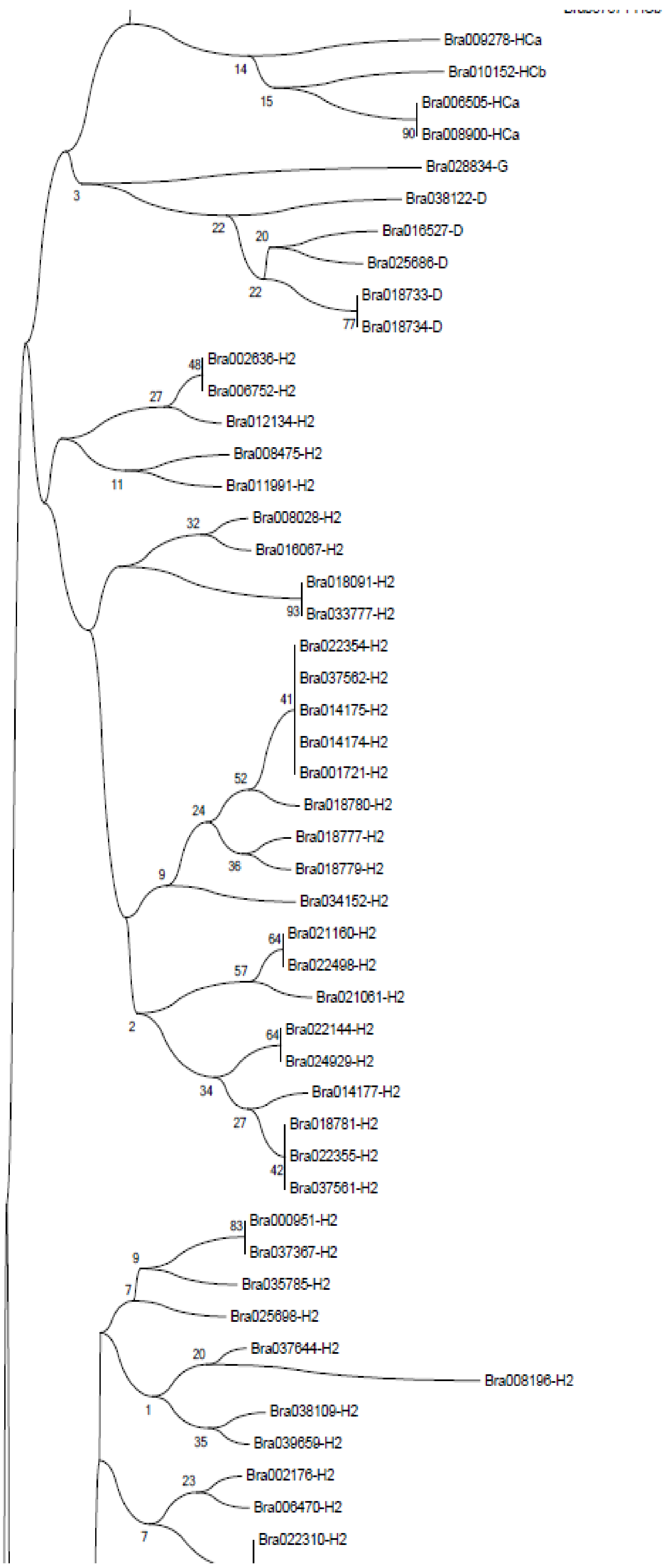
Bra009278-HCa
Bra010152-HCb
Bra006505-HCa
Bra008900-HCa
Bra028834-G
Bra038122-D
Bra016527-D
Bra025686-D
Bra018733-D
Bra018734-D
Bra002636-H2
Bra006752-H2
Bra012134-H2
Bra008475-H2
Bra011991-H2
Bra008028-H2
Bra016067-H2
Bra018091-H2
Bra033777-H2
Bra022354-H2
Bra037562-H2
Bra014175-H2
Bra014174-H2
Bra001721-H2
Bra018780-H2
Bra018777-H2
Bra018779-H2
Bra034152-H2
Bra021160-H2
Bra022498-H2
Bra021061-H2
Bra022144-H2
Bra024929-H2
Bra014177-H2
Bra018781-H2
Bra022355-H2
Bra037561-H2
Bra000951-H2
Bra037367-H2
Bra035785-H2
Bra025698-H2
Bra037644-H2
Bra008196-H2
Bra038109-H2
Bra039659-H2
Bra002176-H2
Bra006470-H2
Bra022310-H2

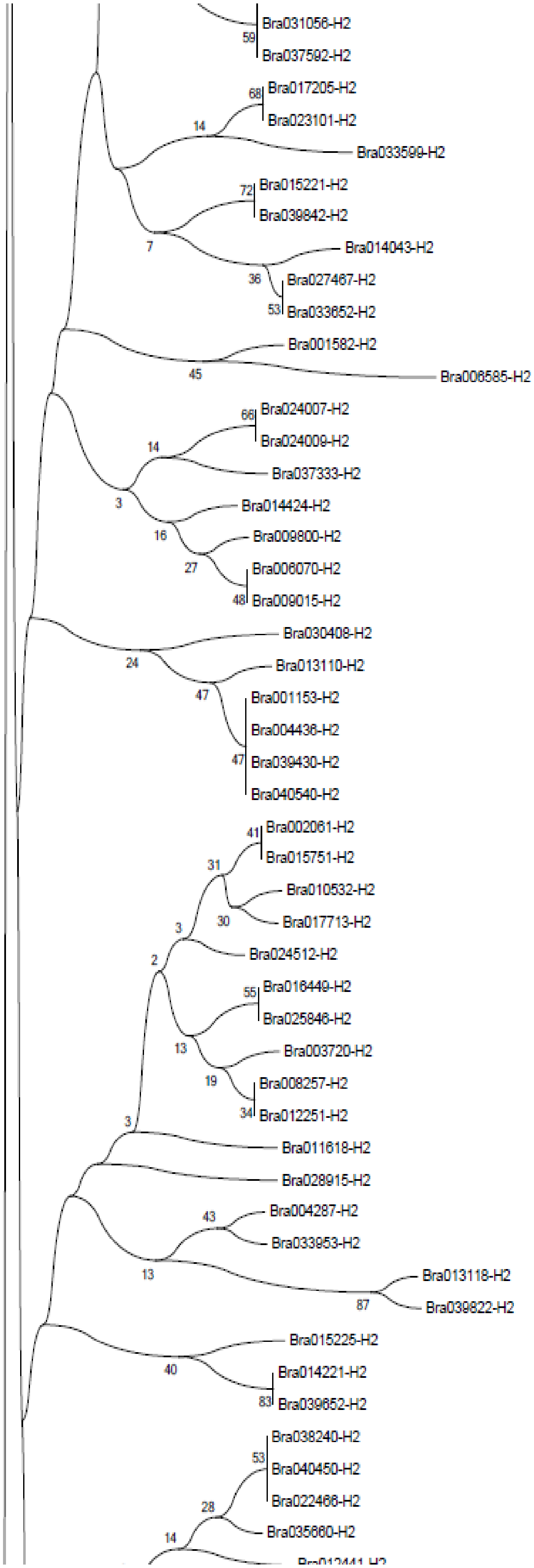
Bra031056-H2
Bra037592-H2
Bra017205-H2
Bra023101-H2
Bra033599-H2
Bra015221-H2
Bra039842-H2
Bra014043-H2
Bra027467-H2
Bra033652-H2
Bra001582-H2
Bra006585-H2
Bra024007-H2
Bra024009-H2
Bra037333-H2
Bra014424-H2
Bra009800-H2
Bra006070-H2
Bra009015-H2
Bra030408-H2
Bra013110-H2
Bra001153-H2
Bra004436-H2
Bra039430-H2
Bra040540-H2
Bra002061-H2
Bra015751-H2
Bra010532-H2
Bra017713-H2
Bra024512-H2
Bra016449-H2
Bra025846-H2
Bra003720-H2
Bra008257-H2
Bra012251-H2
Bra011618-H2
Bra028915-H2
Bra004287-H2
Bra033953-H2
Bra013118-H2
Bra039822-H2
Bra015225-H2
Bra014221-H2
Bra039652-H2
Bra038240-H2
Bra040450-H2
Bra022466-H2
Bra035660-H2

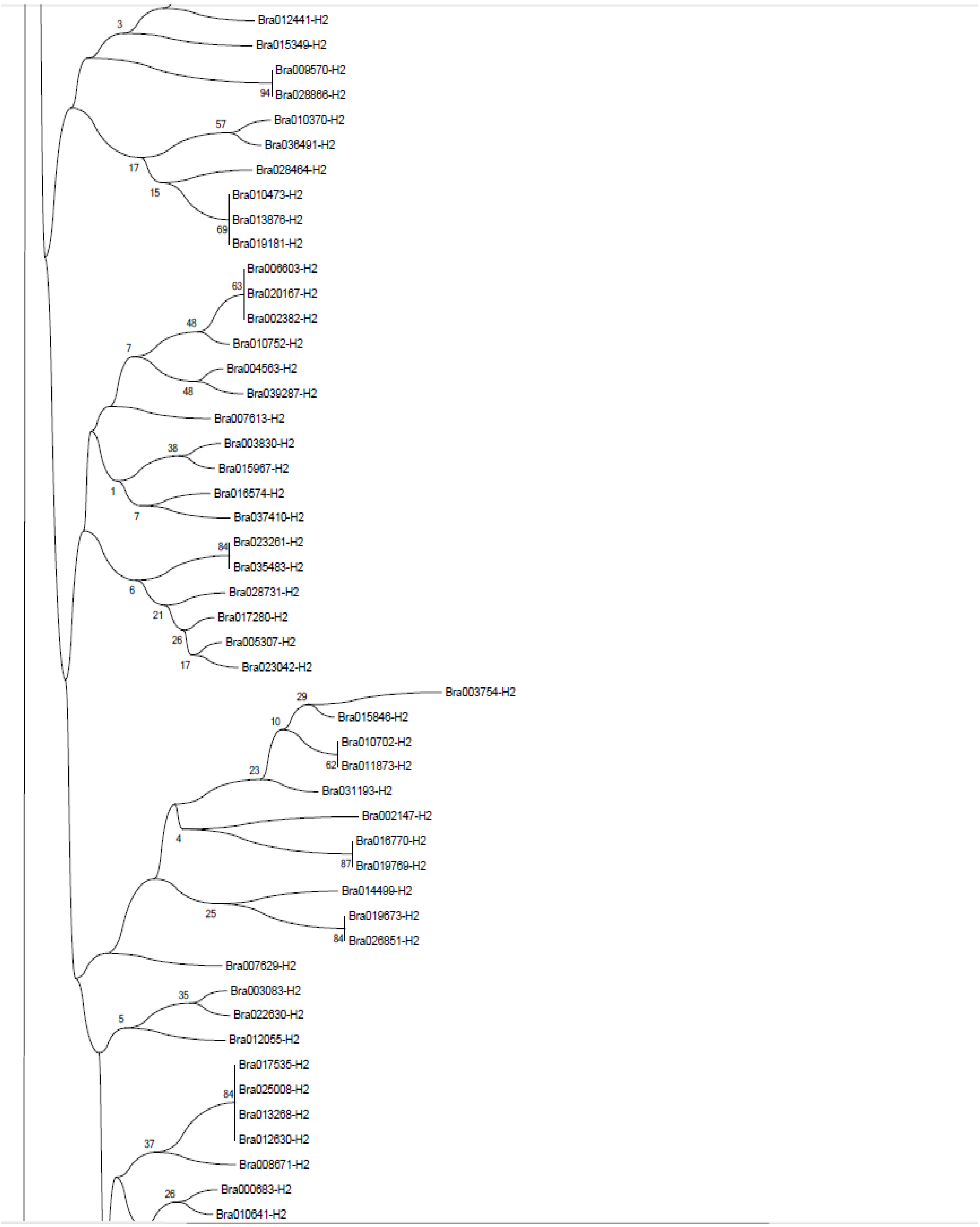
Bra012441-H2
Bra015349-H2
Bra009570-H2
Bra028866-H2
Bra010370-H2
Bra036491-H2
Bra028464-H2
Bra010473-H2
Bra013876-H2
Bra019181-H2
Bra006603-H2
Bra020167-H2
Bra002382-H2
Bra010752-H2
Bra004563-H2
Bra039287-H2
Bra007613-H2
Bra003830-H2
Bra015967-H2
Bra016574-H2
Bra037410-H2
Bra023261-H2
Bra035483-H2
Bra028731-H2
Bra017280-H2
Bra005307-H2
Bra023042-H2
Bra003754-H2
Bra015846-H2
Bra010702-H2
Bra011873-H2
Bra031193-H2
Bra002147-H2
Bra016770-H2
Bra019769-H2
Bra014499-H2
Bra019673-H2
Bra026851-H2
Bra007629-H2
Bra003083-H2
Bra022630-H2
Bra012055-H2
Bra017535-H2
Bra025008-H2
Bra013268-H2
Bra012630-H2
Bra008671-H2
Bra000683-H2
Bra010641-H2

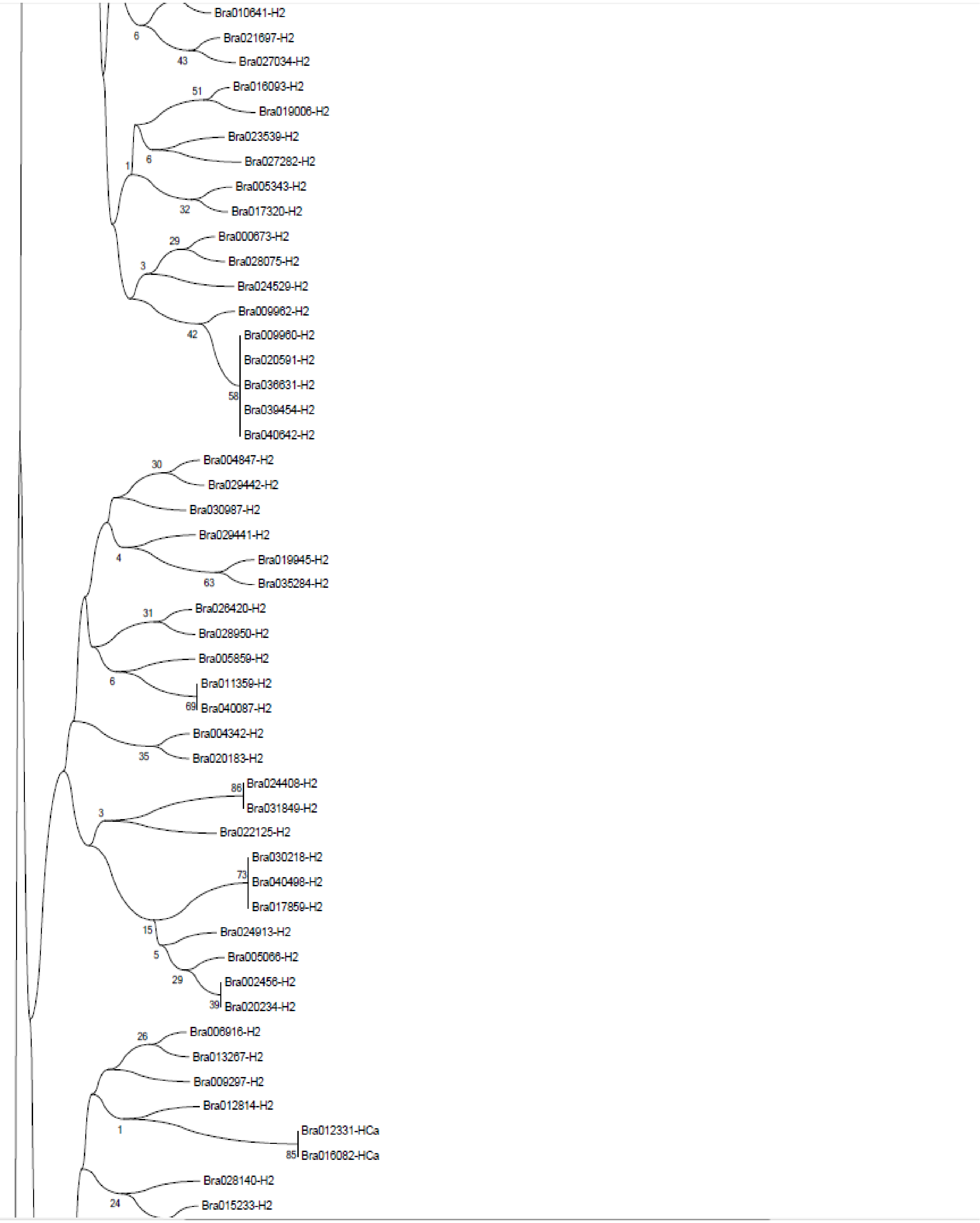
Bra010641-H2
Bra021697-H2
Bra027034-H2
Bra016093-H2
Bra019006-H2
Bra023539-H2
Bra027282-H2
Bra005343-H2
Bra017320-H2
Bra000673-H2
Bra028075-H2
Bra024529-H2
Bra009962-H2
Bra009960-H2
Bra020591-H2
Bra036631-H2
Bra039454-H2
Bra040642-H2
Bra004847-H2
Bra029442-H2
Bra030987-H2
Bra029441-H2
Bra019945-H2
Bra035284-H2
Bra026420-H2
Bra028950-H2
Bra005859-H2
Bra011359-H2
Bra040087-H2
Bra004342-H2
Bra020183-H2
Bra024408-H2
Bra031849-H2
Bra022125-H2
Bra030218-H2
Bra040498-H2
Bra017859-H2
Bra024913-H2
Bra005066-H2
Bra002456-H2
Bra020234-H2
Bra006916-H2
Bra013267-H2
Bra009297-H2
Bra012814-H2
Bra012331-HCa
Bra016082-HCa
Bra028140-H2
Bra015233-H2

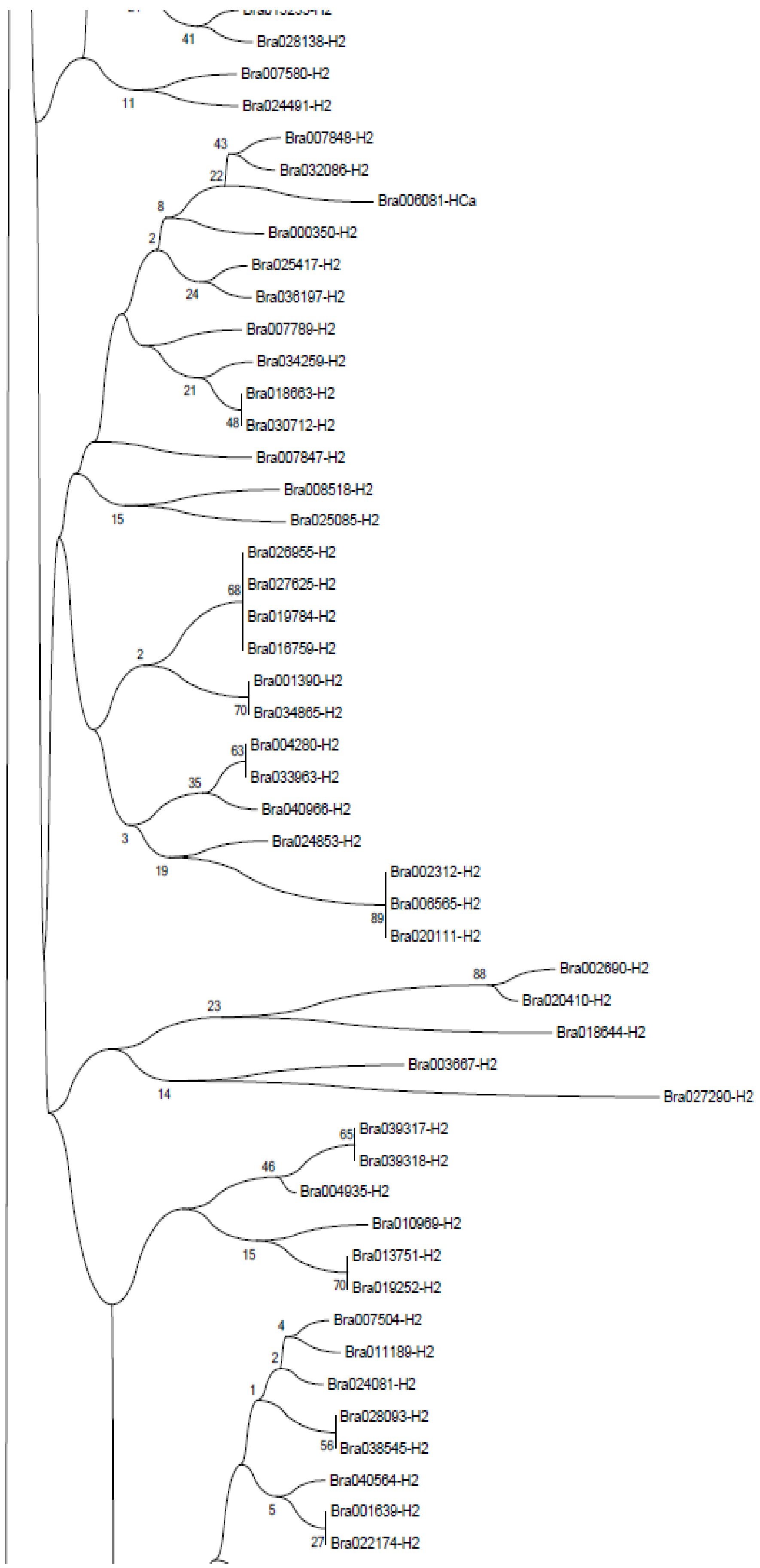
Bra028138-H2
Bra007580-H2
Bra024491-H2
Bra007848-H2
Bra032086-H2
Bra006081-HCa
Bra000350-H2
Bra025417-H2
Bra036197-H2
Bra007789-H2
Bra034259-H2
Bra018663-H2
Bra030712-H2
Bra007847-H2
Bra008518-H2
Bra025085-H2
Bra026955-H2
Bra027625-H2
Bra019784-H2
Bra016759-H2
Bra001390-H2
Bra034865-H2
Bra004280-H2
Bra033963-H2
Bra040966-H2
Bra024853-H2
Bra002312-H2
Bra006565-H2
Bra020111-H2
Bra002690-H2
Bra020410-H2
Bra018644-H2
Bra003667-H2
Bra027290-H2
Bra039317-H2
Bra039318-H2
Bra004935-H2
Bra010969-H2
Bra013751-H2
Bra019252-H2
Bra007504-H2
Bra011189-H2
Bra024081-H2
Bra028093-H2
Bra038545-H2
Bra040564-H2
Bra001639-H2
Bra022174-H2

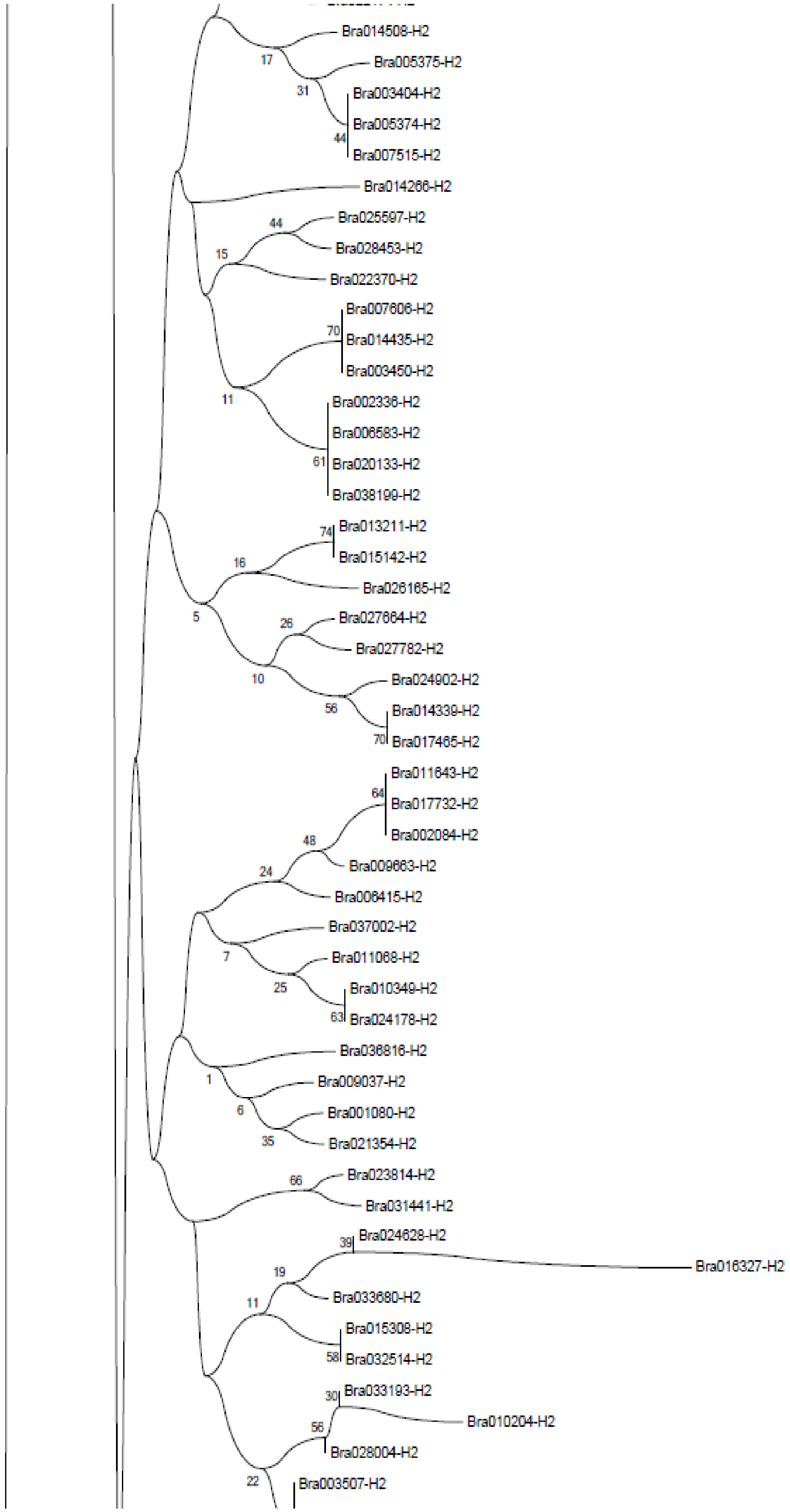
Bra014508-H2
Bra005375-H2
Bra003404-H2
Bra005374-H2
Bra007515-H2
Bra014266-H2
Bra025597-H2
Bra028453-H2
Bra022370-H2
Bra007606-H2
Bra014435-H2
Bra003450-H2
Bra002336-H2
Bra006583-H2
Bra020133-H2
Bra038199-H2
Bra013211-H2
Bra015142-H2
Bra026165-H2
Bra027664-H2
Bra027782-H2
Bra024902-H2
Bra014339-H2
Bra017465-H2
Bra011643-H2
Bra017732-H2
Bra002084-H2
Bra009663-H2
Bra006415-H2
Bra037002-H2
Bra011068-H2
Bra010349-H2
Bra024178-H2
Bra036816-H2
Bra009037-H2
Bra001080-H2
Bra021354-H2
Bra023814-H2
Bra031441-H2
Bra024628-H2
Bra016327-H2
Bra033680-H2
Bra015308-H2
Bra032514-H2
Bra033193-H2
Bra010204-H2
Bra028004-H2
Bra003507-H2

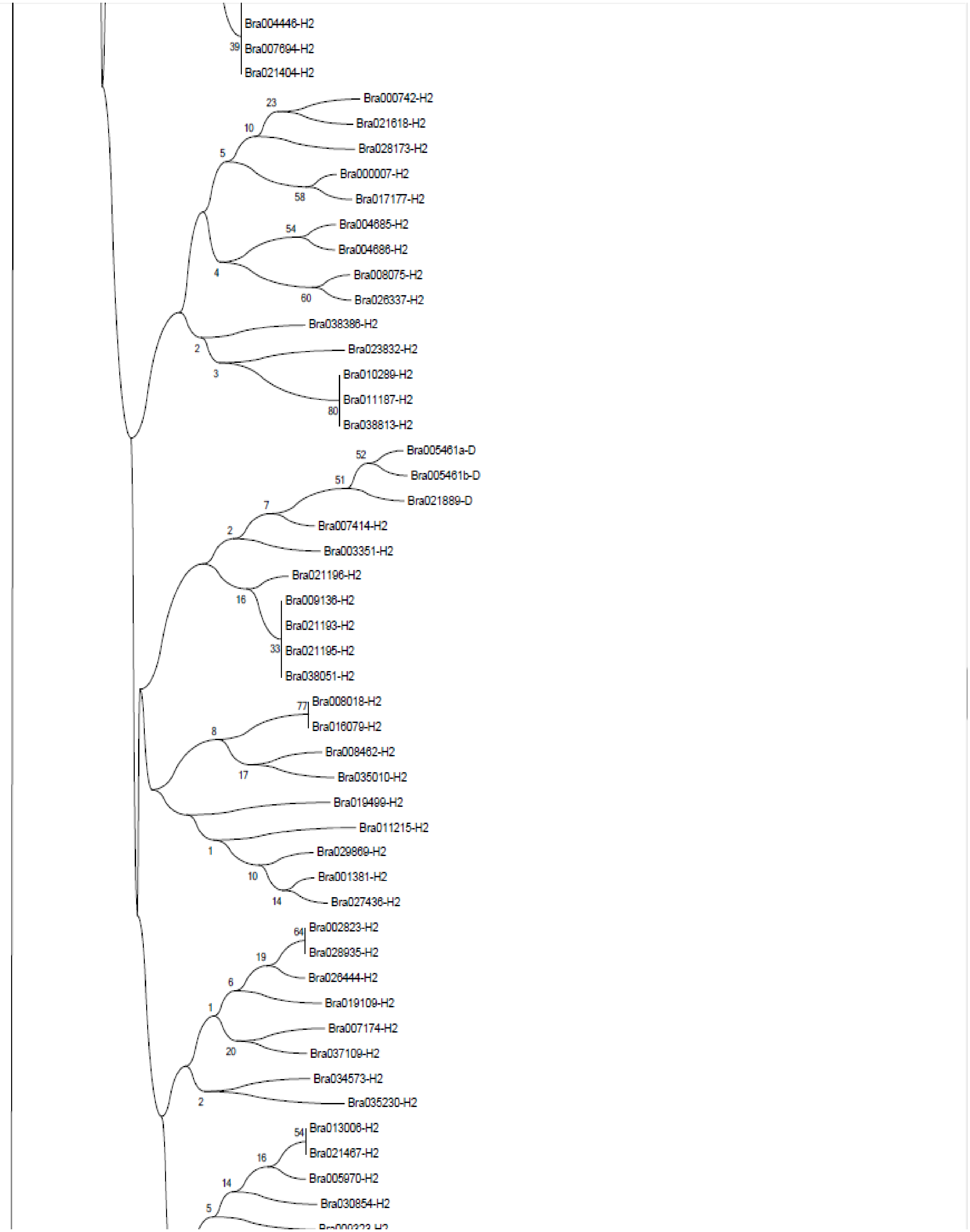
Bra004446-H2
Bra007694-H2
Bra021404-H2
Bra000742-H2
Bra021618-H2
Bra028173-H2
Bra000007-H2
Bra017177-H2
Bra004685-H2
Bra004686-H2
Bra008075-H2
Bra026337-H2
Bra038386-H2
Bra023832-H2
Bra010289-H2
Bra011187-H2
Bra038813-H2
Bra005461a-D
Bra005461b-D
Bra021889-D
Bra007414-H2
Bra003351-H2
Bra021196-H2
Bra009136-H2
Bra021193-H2
Bra021195-H2
Bra038051-H2
Bra008018-H2
Bra016079-H2
Bra008462-H2
Bra035010-H2
Bra019499-H2
Bra011215-H2
Bra029869-H2
Bra001381-H2
Bra027436-H2
Bra002823-H2
Bra028935-H2
Bra026444-H2
Bra019109-H2
Bra007174-H2
Bra037109-H2
Bra034573-H2
Bra035230-H2
Bra013006-H2
Bra021467-H2
Bra005970-H2
Bra030854-H2

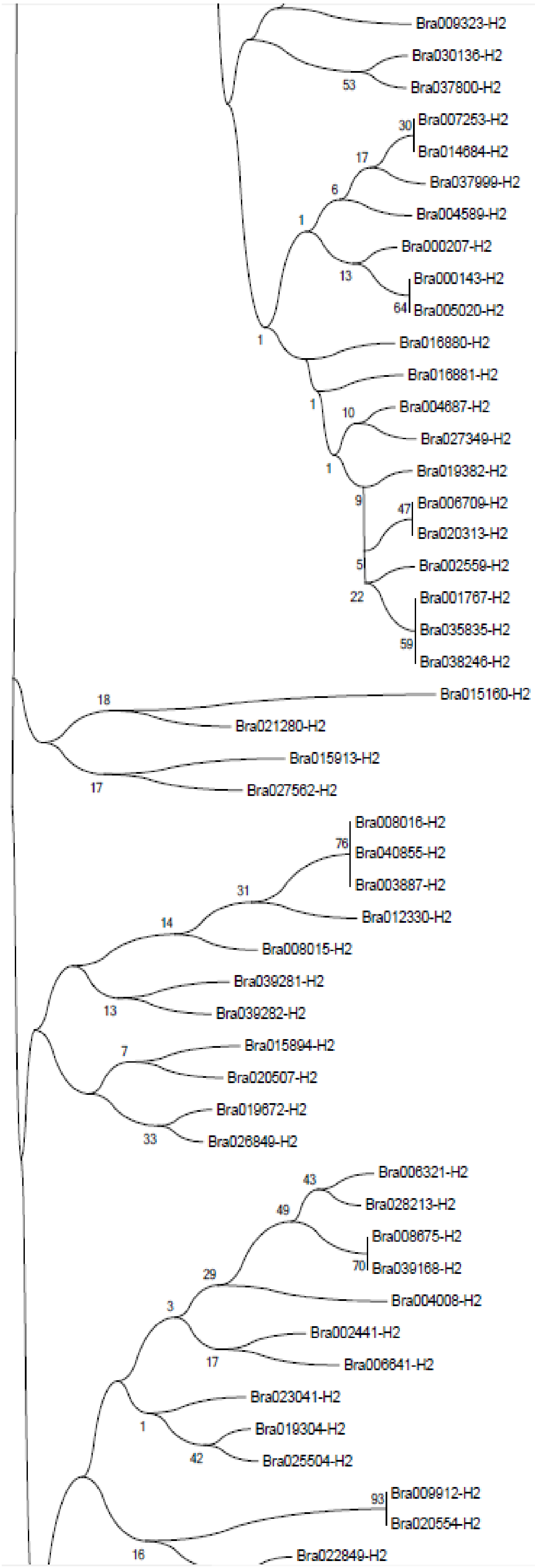

Bra009323-H2
Bra030136-H2
Bra037800-H2
Bra007253-H2
Bra014684-H2
Bra037999-H2
Bra004589-H2
Bra000207-H2
Bra000143-H2
Bra005020-H2
Bra016880-H2
Bra016881-H2
Bra004687-H2
Bra027349-H2
Bra019382-H2
Bra006709-H2
Bra020313-H2
Bra002559-H2
Bra001767-H2
Bra035835-H2
Bra038246-H2
Bra015160-H2
Bra021280-H2
Bra015913-H2
Bra027562-H2
Bra008016-H2
Bra040855-H2
Bra003887-H2
Bra012330-H2
Bra008015-H2
Bra039281-H2
Bra039282-H2
Bra015894-H2
Bra020507-H2
Bra019672-H2
Bra026849-H2
Bra006321-H2
Bra028213-H2
Bra008675-H2
Bra039168-H2
Bra004008-H2
Bra002441-H2
Bra006641-H2
Bra023041-H2
Bra019304-H2
Bra025504-H2
Bra009912-H2
Bra020554-H2
Bra022849-H2

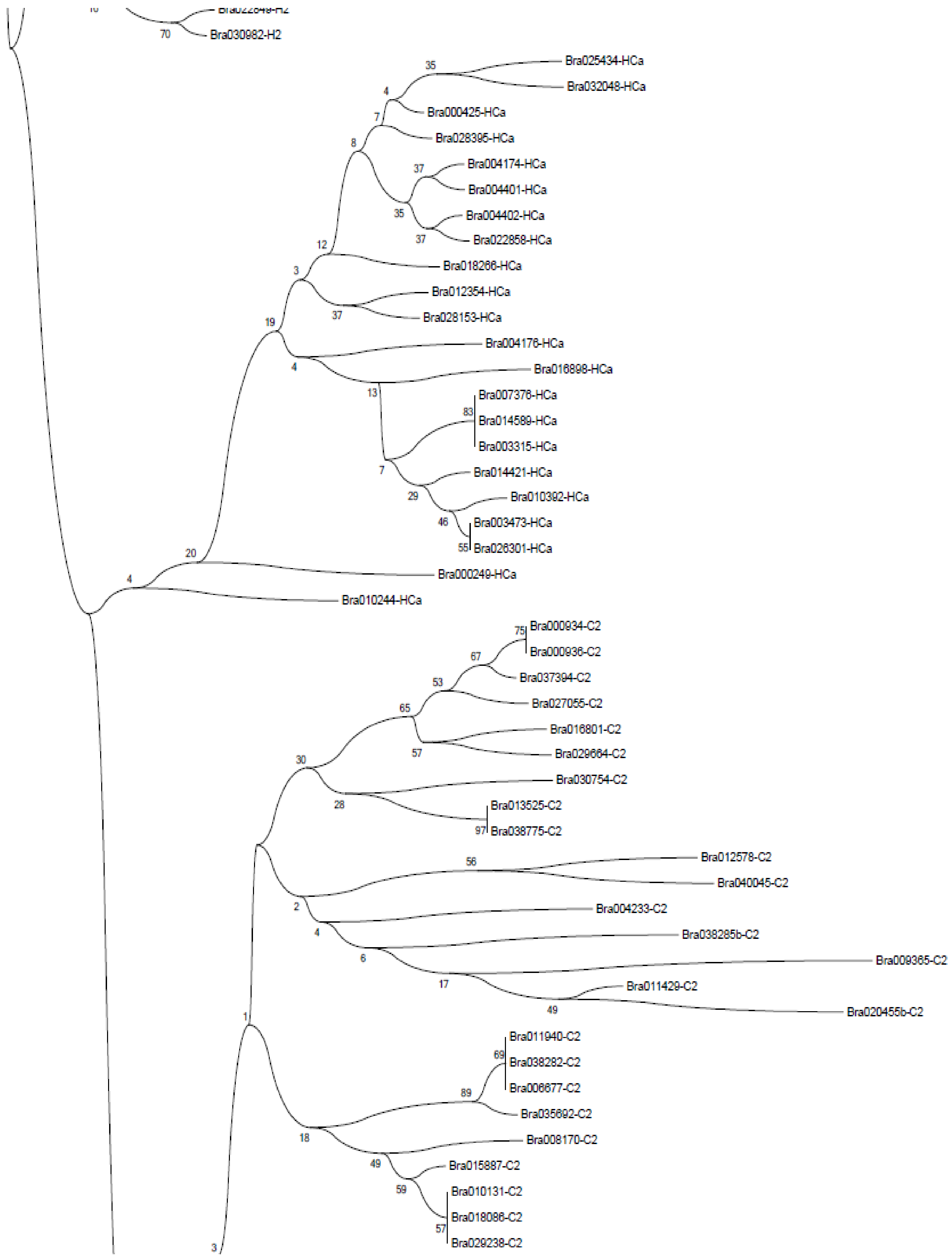

Bra030982-H2
Bra025434-HCa
Bra032048-HCa
Bra000425-HCa
Bra028395-HCa
Bra004174-HCa
Bra004401-HCa
Bra004402-HCa
Bra022858-HCa
Bra018266-HCa
Bra012354-HCa
Bra028153-HCa
Bra004176-HCa
Bra016898-HCa
Bra007376-HCa
Bra014589-HCa
Bra003315-HCa
Bra014421-HCa
Bra010392-HCa
Bra003473-HCa
Bra026301-HCa
Bra000249-HCa
Bra010244-HCa
Bra000934-C2
Bra000936-C2
Bra037394-C2
Bra027055-C2
Bra016801-C2
Bra029664-C2
Bra030754-C2
Bra013525-C2
Bra038775-C2
Bra012578-C2
Bra040045-C2
Bra004233-C2
Bra038285b-C2
Bra009365-C2
Bra011429-C2
Bra020455b-C2
Bra011940-C2
Bra038282-C2
Bra006677-C2
Bra035692-C2
Bra008170-C2
Bra015887-C2
Bra010131-C2
Bra018086-C2
Bra029238-C2

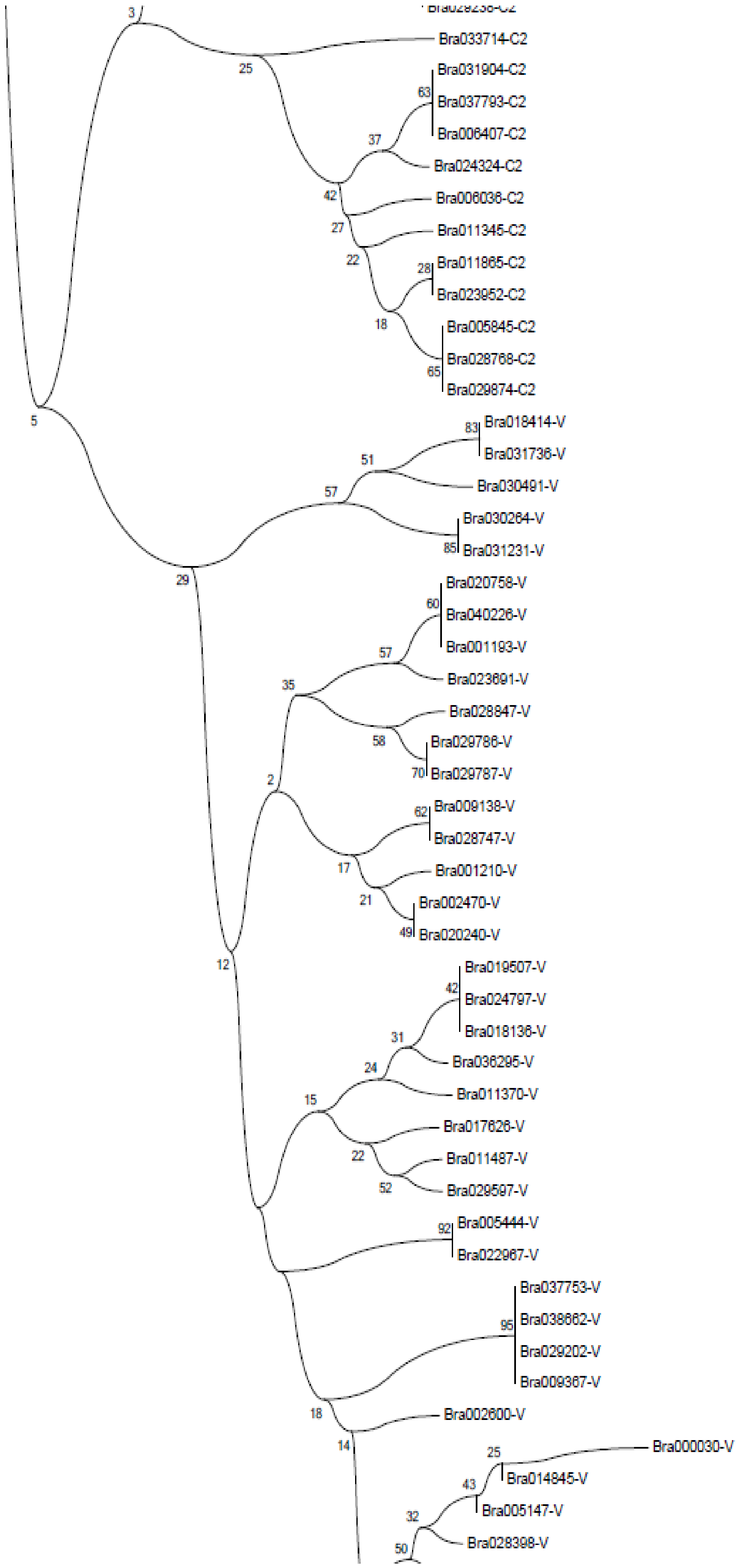
Bra033714-C2
Bra031904-C2
Bra037793-C2
Bra006407-C2
Bra024324-C2
Bra006036-C2
Bra011345-C2
Bra011865-C2
Bra023952-C2
Bra005845-C2
Bra028768-C2
Bra029874-C2
Bra018414-V
Bra031736-V
Bra030491-V
Bra030264-V
Bra031231-V
Bra020758-V
Bra040226-V
Bra001193-V
Bra023691-V
Bra028847-V
Bra029786-V
Bra029787-V
Bra009138-V
Bra028747-V
Bra001210-V
Bra002470-V
Bra020240-V
Bra019507-V
Bra024797-V
Bra018136-V
Bra036295-V
Bra011370-V
Bra017626-V
Bra011487-V
Bra029597-V
Bra005444-V
Bra022967-V
Bra037753-V
Bra038662-V
Bra029202-V
Bra009367-V
Bra002600-V
Bra000030-V
Bra014845-V
Bra005147-V
Bra028398-V

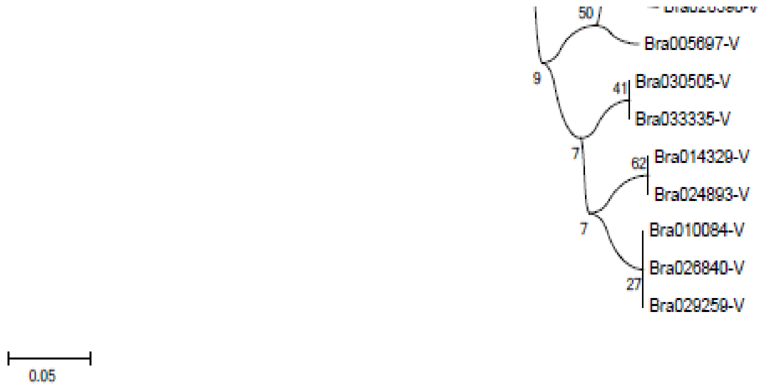

Figure S7 Expression profile of 667 RING finger protein genes in *B. rapa*, subdivided into groups (I-VII). The scale indicating the relative expression signal values is shown above.

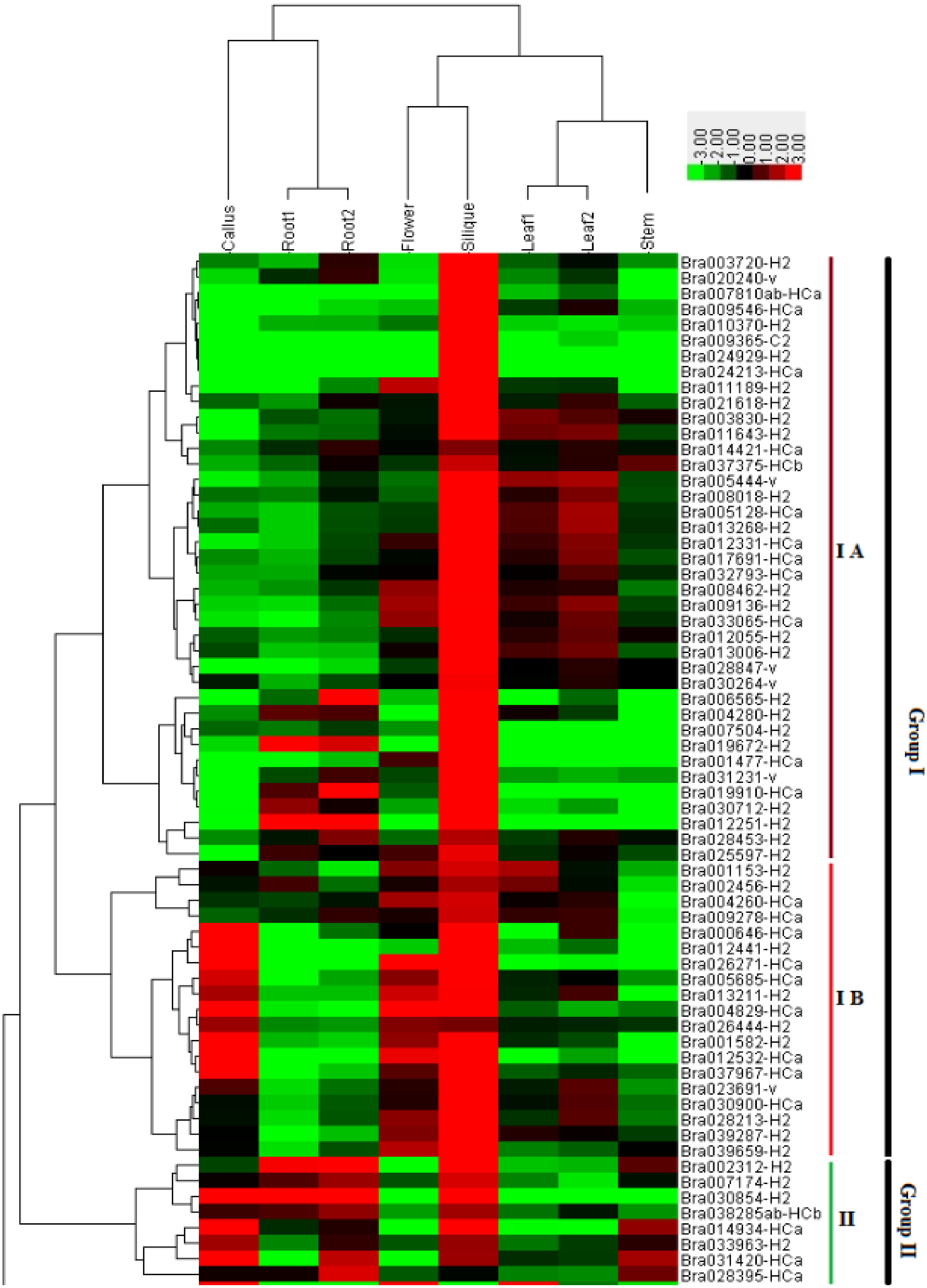
-3.00
-2.00
-1.00
0.00
1.00
2.00
3.00
Callus
Root1
Root2
Flower
Silique
Leaf1
Leaf2
Stem
Bra003720-H2
Bra020240-v
Bra007810ab-HCa
Bra009546-HCa
Bra010370-H2
Bra009365-C2
Bra024929-H2
Bra024213-HCa
Bra011189-H2
Bra021618-H2
Bra003830-H2
Bra011643-H2
Bra014421-HCa
Bra037375-HCb
Bra005444-v
Bra008018-H2
Bra005128-HCa
Bra013268-H2
Bra012331-HCa
Bra017691-HCa
Bra032793-HCa
Bra008462-H2
Bra009136-H2
Bra033065-HCa
Bra012055-H2
Bra013006-H2
Bra028847-v
Bra030264-v
Bra006565-H2
Bra004280-H2
Bra007504-H2
Bra019672-H2
Bra001477-HCa
Bra031231-v
Bra019910-HCa
Bra030712-H2
Bra012251-H2
Bra028453-H2
Bra025597-H2
Bra001153-H2
Bra002456-H2
Bra004260-HCa
Bra009278-HCa
Bra000646-HCa
Bra012441-H2
Bra026271-HCa
Bra005685-HCa
Bra013211-H2
Bra004829-HCa
Bra026444-H2
Bra001582-H2
Bra012532-HCa
Bra037967-HCa
Bra023691-v
Bra030900-HCa
Bra028213-H2
Bra039287-H2
Bra039659-H2
Bra002312-H2
Bra007174-H2
Bra030854-H2
Bra038285ab-HCb
Bra014934-HCa
Bra033963-H2
Bra031420-HCa
Bra028395-HCa
I A
I B
II
Group I
Group II

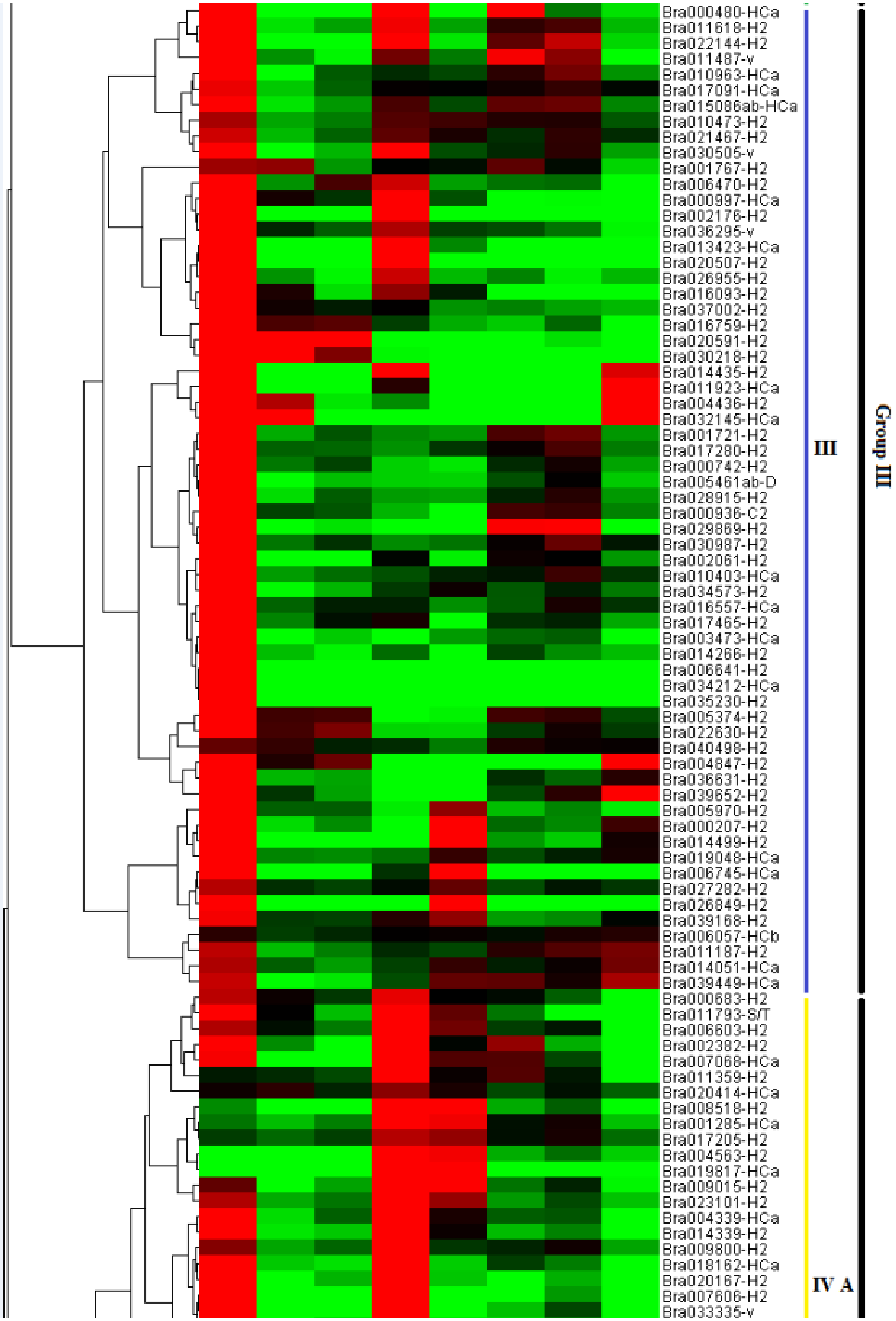
Bra000480-HCa
Bra011618-H2
Bra022144-H2
Bra011487-v
Bra010963-HCa
Bra017091-HCa
Bra015086ab-HCa
Bra010473-H2
Bra021467-H2
Bra030505-v
Bra001767-H2
Bra006470-H2
Bra000997-HCa
Bra002176-H2
Bra036295-v
Bra013423-HCa
Bra020507-H2
Bra026955-H2
Bra016093-H2
Bra037002-H2
Bra016759-H2
Bra020591-H2
Bra030218-H2
Bra014435-H2
Bra011923-HCa
Bra004436-H2
Bra032145-HCa
Bra001721-H2
Bra017280-H2
Bra000742-H2
Bra005461ab-D
Bra028915-H2
Bra000936-C2
Bra029869-H2
Bra030987-H2
Bra002061-H2
Bra010403-HCa
Bra034573-H2
Bra016557-HCa
Bra017465-H2
Bra003473-HCa
Bra014266-H2
Bra006641-H2
Bra034212-HCa
Bra035230-H2
Bra005374-H2
Bra022630-H2
Bra040498-H2
Bra004847-H2
Bra036631-H2
Bra039652-H2
Bra005970-H2
Bra000207-H2
Bra014499-H2
Bra019048-HCa
Bra006745-HCa
Bra027282-H2
Bra026849-H2
Bra039168-H2
Bra006057-HCb
Bra011187-H2
Bra014051-HCa
Bra039449-HCa
Bra000683-H2
Bra011793-S/T
Bra006603-H2
Bra002382-H2
Bra007068-HCa
Bra011359-H2
Bra020414-HCa
Bra008518-H2
Bra001285-HCa
Bra017205-H2
Bra004563-H2
Bra019817-HCa
Bra009015-H2
Bra023101-H2
Bra004339-HCa
Bra014339-H2
Bra009800-H2
Bra018162-HCa
Bra020167-H2
Bra007606-H2
Bra033335-v
III
IV A
Group III

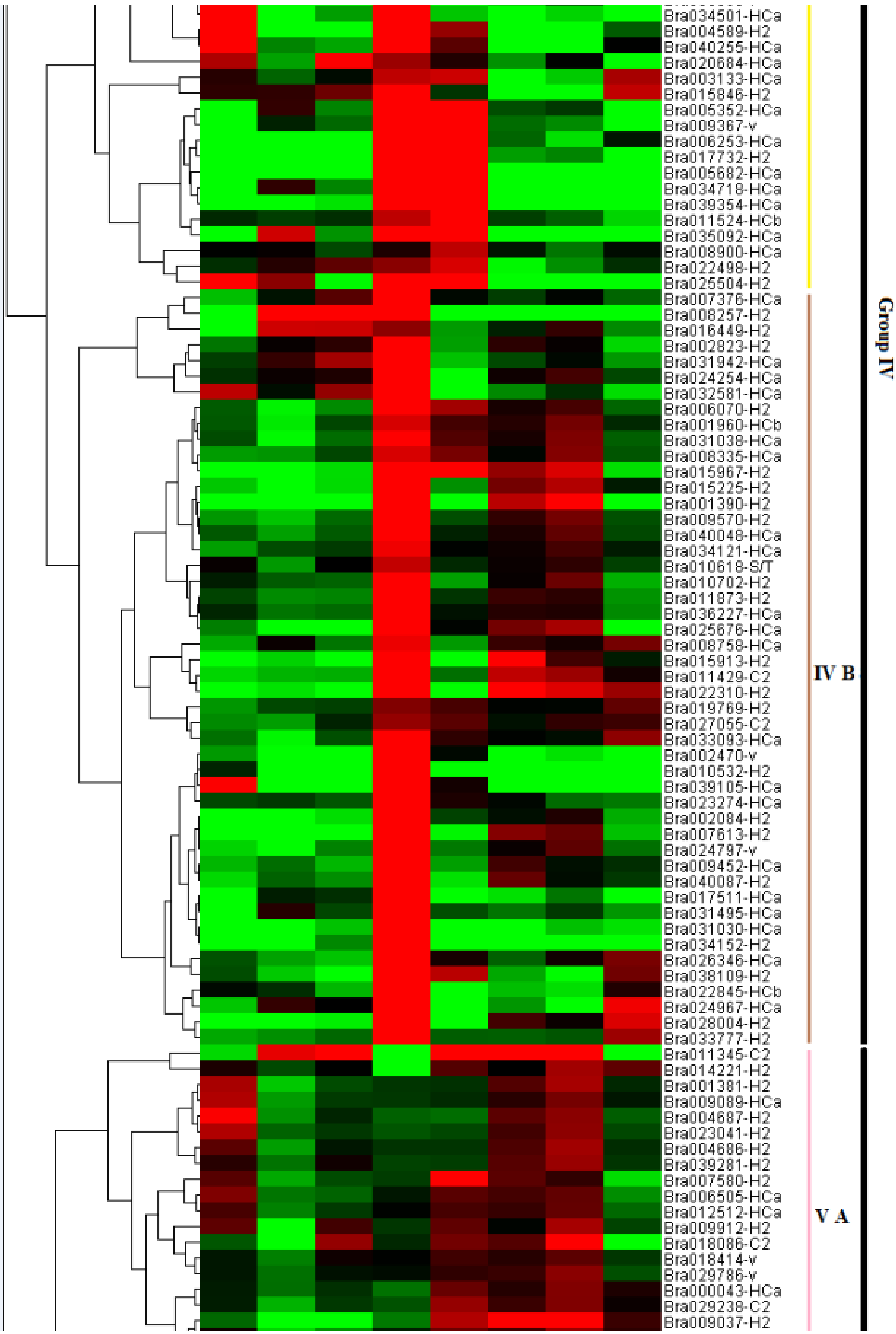
Bra034501-HCa
Bra004589-H2
Bra040255-HCa
Bra020684-HCa
Bra003133-HCa
Bra015846-H2
Bra005352-HCa
Bra009367-v
Bra006253-HCa
Bra017732-H2
Bra005682-HCa
Bra034718-HCa
Bra039354-HCa
Bra011524-HCb
Bra035092-HCa
Bra008900-HCa
Bra022498-H2
Bra025504-H2
Bra007376-HCa
Bra008257-H2
Bra016449-H2
Bra002823-H2
Bra031942-HCa
Bra024254-HCa
Bra032581-HCa
Bra006070-H2
Bra001960-HCb
Bra031038-HCa
Bra008335-HCa
Bra015967-H2
Bra015225-H2
Bra001390-H2
Bra009570-H2
Bra040048-HCa
Bra034121-HCa
Bra010618-S/T
Bra010702-H2
Bra011873-H2
Bra036227-HCa
Bra025676-HCa
Bra008758-HCa
Bra015913-H2
Bra011429-C2
Bra022310-H2
Bra019769-H2
Bra027055-C2
Bra033093-HCa
Bra002470-v
Bra010532-H2
Bra039105-HCa
Bra023274-HCa
Bra002084-H2
Bra007613-H2
Bra024797-v
Bra009452-HCa
Bra040087-H2
Bra017511-HCa
Bra031495-HCa
Bra031030-HCa
Bra034152-H2
Bra026346-HCa
Bra038109-H2
Bra022845-HCb
Bra024967-HCa
Bra028004-H2
Bra033777-H2
Bra011345-C2
Bra014221-H2
Bra001381-H2
Bra009089-HCa
Bra004687-H2
Bra023041-H2
Bra004686-H2
Bra039281-H2
Bra007580-H2
Bra006505-HCa
Bra012512-HCa
Bra009912-H2
Bra018086-C2
Bra018414-v
Bra029786-v
Bra000043-HCa
Bra029238-C2
Bra009037-H2
IV B
V A
Group IV

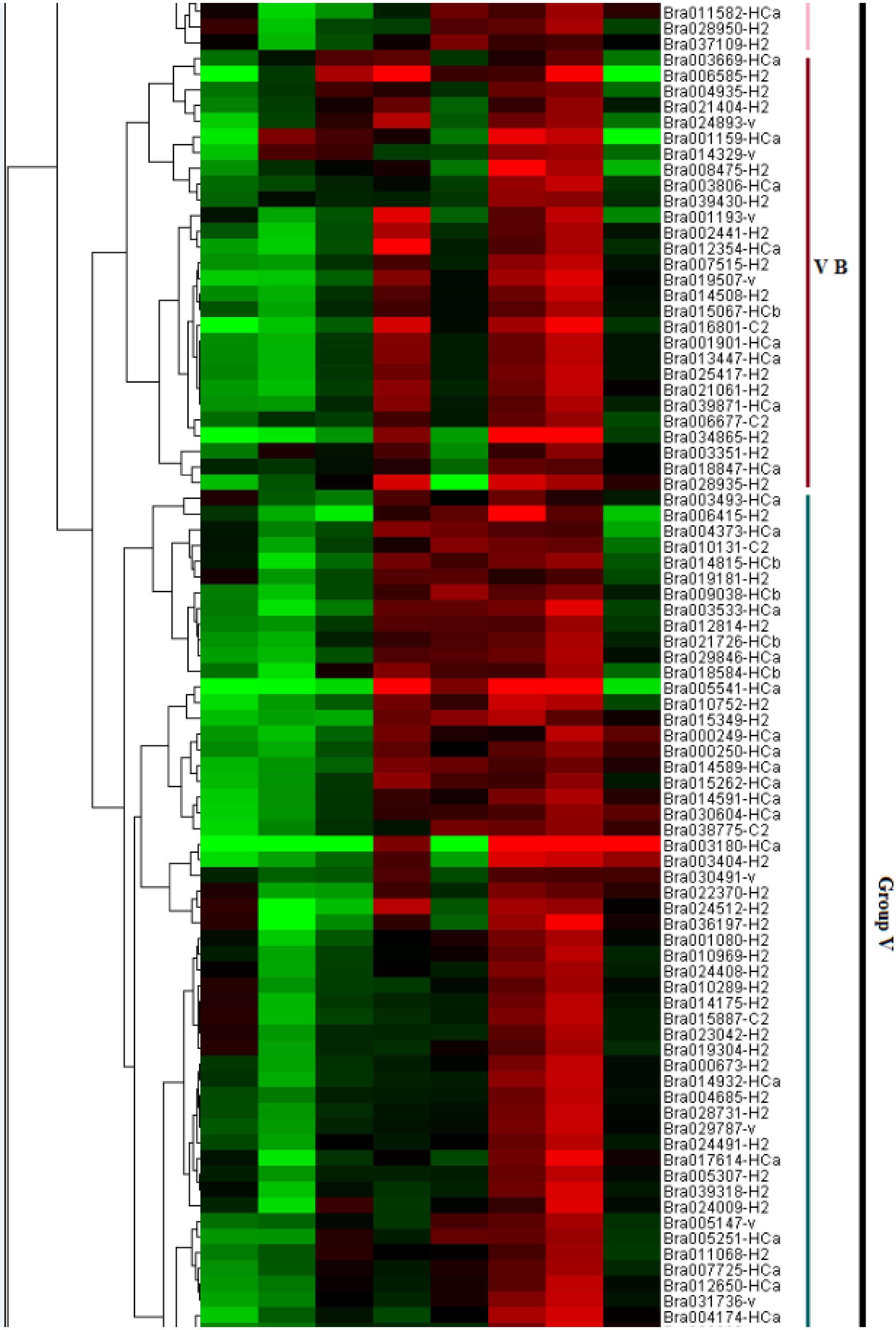
Bra011582-HCa
Bra028950-H2
Bra037109-H2
Bra003669-HCa
Bra006585-H2
Bra004935-H2
Bra021404-H2
Bra024893-v
Bra001159-HCa
Bra014329-v
Bra008475-H2
Bra003806-HCa
Bra039430-H2
Bra001193-v
Bra002441-H2
Bra012354-HCa
Bra007515-H2
Bra019507-v
Bra014508-H2
Bra015067-HCb
Bra016801-C2
Bra001901-HCa
Bra013447-HCa
Bra025417-H2
Bra021061-H2
Bra039871-HCa
Bra006677-C2
Bra034865-H2
Bra003351-H2
Bra018847-HCa
Bra028935-H2
Bra003493-HCa
Bra006415-H2
Bra004373-HCa
Bra010131-C2
Bra014815-HCb
Bra019181-H2
Bra009038-HCb
Bra003533-HCa
Bra012814-H2
Bra021726-HCb
Bra029846-HCa
Bra018584-HCb
Bra005541-HCa
Bra010752-H2
Bra015349-H2
Bra000249-HCa
Bra000250-HCa
Bra014589-HCa
Bra015262-HCa
Bra014591-HCa
Bra030604-HCa
Bra038775-C2
Bra003180-HCa
Bra003404-H2
Bra030491-v
Bra022370-H2
Bra024512-H2
Bra036197-H2
Bra001080-H2
Bra010969-H2
Bra024408-H2
Bra010289-H2
Bra014175-H2
Bra015887-C2
Bra023042-H2
Bra019304-H2
Bra000673-H2
Bra014932-HCa
Bra004685-H2
Bra028731-H2
Bra029787-v
Bra024491-H2
Bra017614-HCa
Bra005307-H2
Bra039318-H2
Bra024009-H2
Bra005147-v
Bra005251-HCa
Bra011068-H2
Bra007725-HCa
Bra012650-HCa
Bra031736-v
Bra004174-HCa
V B
Group V

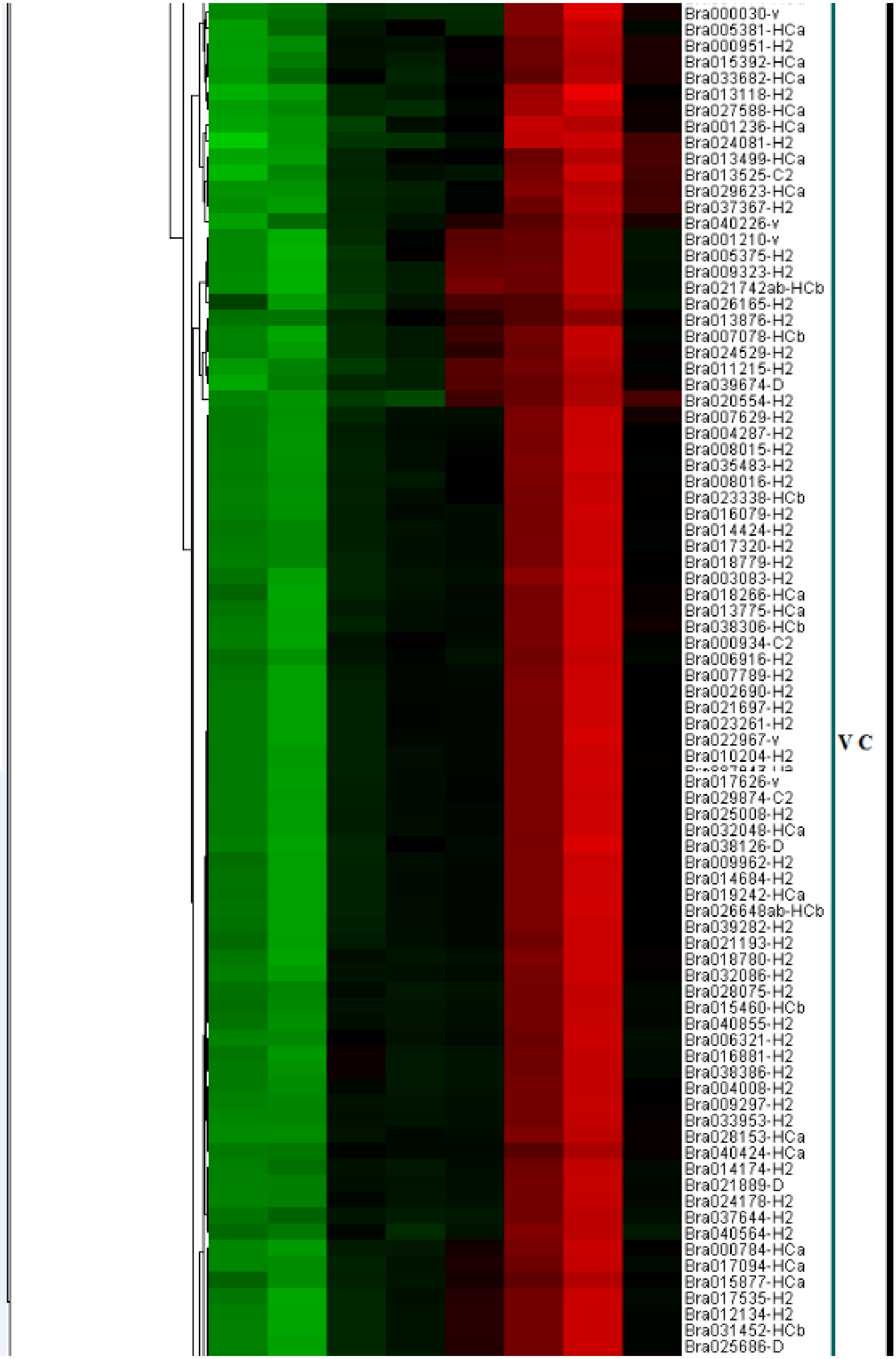
Bra000030-v
Bra005381-HCa
Bra000951-H2
Bra015392-HCa
Bra033682-HCa
Bra013118-H2
Bra027588-HCa
Bra001236-HCa
Bra024081-H2
Bra013499-HCa
Bra013525-C2
Bra029623-HCa
Bra037367-H2
Bra040226-v
Bra001210-v
Bra005375-H2
Bra009323-H2
Bra021742ab-HCb
Bra026165-H2
Bra013876-H2
Bra007078-HCb
Bra024529-H2
Bra011215-H2
Bra039674-D
Bra020554-H2
Bra007629-H2
Bra004287-H2
Bra008015-H2
Bra035483-H2
Bra008016-H2
Bra023338-HCb
Bra016079-H2
Bra014424-H2
Bra017320-H2
Bra018779-H2
Bra003083-H2
Bra018266-HCa
Bra013775-HCa
Bra038306-HCb
Bra000934-C2
Bra006916-H2
Bra007789-H2
Bra002690-H2
Bra021697-H2
Bra023261-H2
Bra022967-v
Bra010204-H2
Bra017626-v
Bra029874-C2
Bra025008-H2
Bra032048-HCa
Bra038126-D
Bra009962-H2
Bra014684-H2
Bra019242-HCa
Bra026648ab-HCb
Bra039282-H2
Bra021193-H2
Bra018780-H2
Bra032086-H2
Bra028075-H2
Bra015460-HCb
Bra040855-H2
Bra006321-H2
Bra016881-H2
Bra038386-H2
Bra004008-H2
Bra009297-H2
Bra033953-H2
Bra028153-HCa
Bra040424-HCa
Bra014174-H2
Bra021889-D
Bra024178-H2
Bra037644-H2
Bra040564-H2
Bra000784-HCa
Bra017094-HCa
Bra015877-HCa
Bra017535-H2
Bra012134-H2
Bra031452-HCb
Bra025686-D
V C

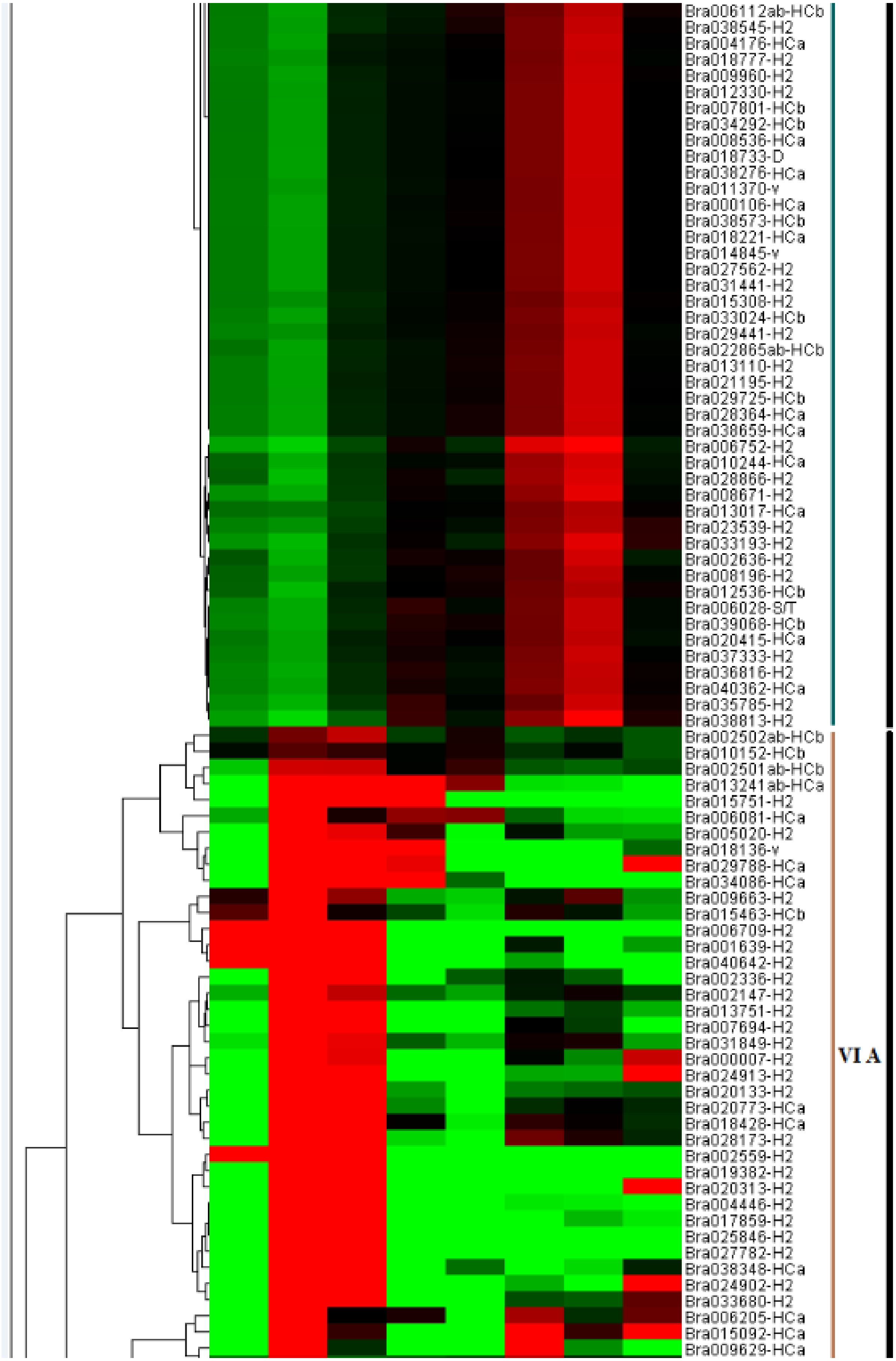
Bra006112ab-HCb
Bra038545-H2
Bra004176-HCa
Bra018777-H2
Bra009960-H2
Bra012330-H2
Bra007801-HCb
Bra034292-HCb
Bra008536-HCa
Bra018733-D
Bra038276-HCa
Bra011370-v
Bra000106-HCa
Bra038573-HCb
Bra018221-HCa
Bra014845-v
Bra027562-H2
Bra031441-H2
Bra015308-H2
Bra033024-HCb
Bra029441-H2
Bra022865ab-HCb
Bra013110-H2
Bra021195-H2
Bra029725-HCb
Bra028364-HCa
Bra038659-HCa
Bra006752-H2
Bra010244-HCa
Bra028866-H2
Bra008671-H2
Bra013017-HCa
Bra023539-H2
Bra033193-H2
Bra002636-H2
Bra008196-H2
Bra012536-HCb
Bra006028-S/T
Bra039068-HCb
Bra020415-HCa
Bra037333-H2
Bra036816-H2
Bra040362-HCa
Bra035785-H2
Bra038813-H2
Bra002502ab-HCb
Bra010152-HCb
Bra002501ab-HCb
Bra013241ab-HCa
Bra015751-H2
Bra006081-HCa
Bra005020-H2
Bra018136-v
Bra029788-HCa
Bra034086-HCa
Bra009663-H2
Bra015463-HCb
Bra006709-H2
Bra001639-H2
Bra040642-H2
Bra002336-H2
Bra002147-H2
Bra013751-H2
Bra007694-H2
Bra031849-H2
Bra000007-H2
Bra024913-H2
Bra020133-H2
Bra020773-HCa
Bra018428-HCa
Bra028173-H2
Bra002559-H2
Bra019382-H2
Bra020313-H2
Bra004446-H2
Bra017859-H2
Bra025846-H2
Bra027782-H2
Bra038348-HCa
Bra024902-H2
Bra033680-H2
Bra006205-HCa
Bra015092-HCa
Bra009629-HCa
VI A

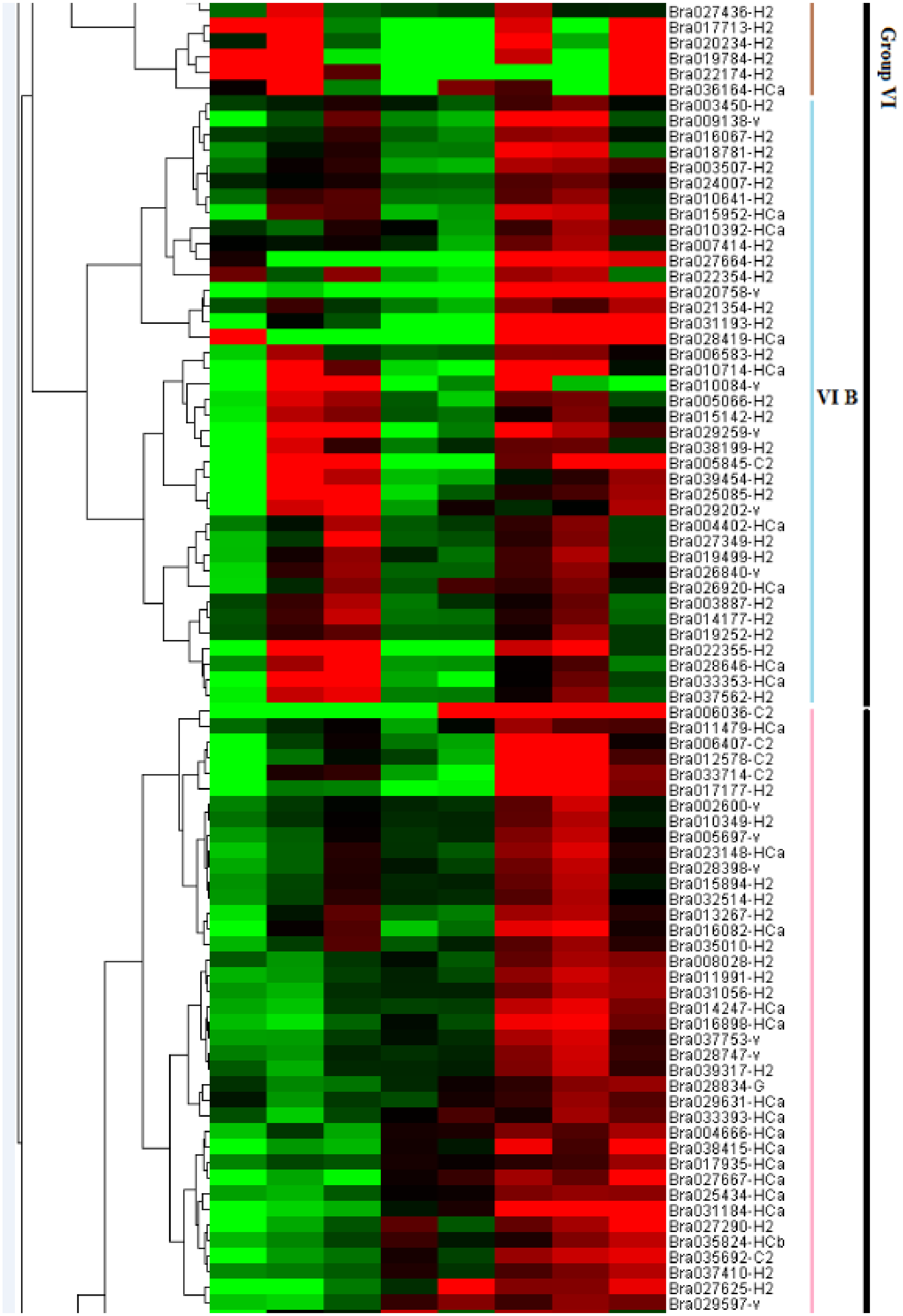
Bra027436-H2
Bra017713-H2
Bra020234-H2
Bra019784-H2
Bra022174-H2
Bra036164-HCa
Bra003450-H2
Bra009138-v
Bra016067-H2
Bra018781-H2
Bra003507-H2
Bra024007-H2
Bra010641-H2
Bra015952-HCa
Bra010392-HCa
Bra007414-H2
Bra027664-H2
Bra022354-H2
Bra020758-v
Bra021354-H2
Bra031193-H2
Bra028419-HCa
Bra006583-H2
Bra010714-HCa
Bra010084-v
Bra005066-H2
Bra015142-H2
Bra029259-v
Bra038199-H2
Bra005845-C2
Bra039454-H2
Bra025085-H2
Bra029202-v
Bra004402-HCa
Bra027349-H2
Bra019499-H2
Bra026840-v
Bra026920-HCa
Bra003887-H2
Bra014177-H2
Bra019252-H2
Bra022355-H2
Bra028646-HCa
Bra033353-HCa
Bra037562-H2
Bra006036-C2
Bra011479-HCa
Bra006407-C2
Bra012578-C2
Bra033714-C2
Bra017177-H2
Bra002600-v
Bra010349-H2
Bra005697-v
Bra023148-HCa
Bra028398-v
Bra015894-H2
Bra032514-H2
Bra013267-H2
Bra016082-HCa
Bra035010-H2
Bra008028-H2
Bra011991-H2
Bra031056-H2
Bra014247-HCa
Bra016898-HCa
Bra037753-v
Bra028747-v
Bra039317-H2
Bra028834-G
Bra029631-HCa
Bra033393-HCa
Bra004666-HCa
Bra038415-HCa
Bra017935-HCa
Bra027667-HCa
Bra025434-HCa
Bra031184-HCa
Bra027290-H2
Bra035824-HCb
Bra035692-C2
Bra037410-H2
Bra027625-H2
Bra029597-v
VI B
Group VI

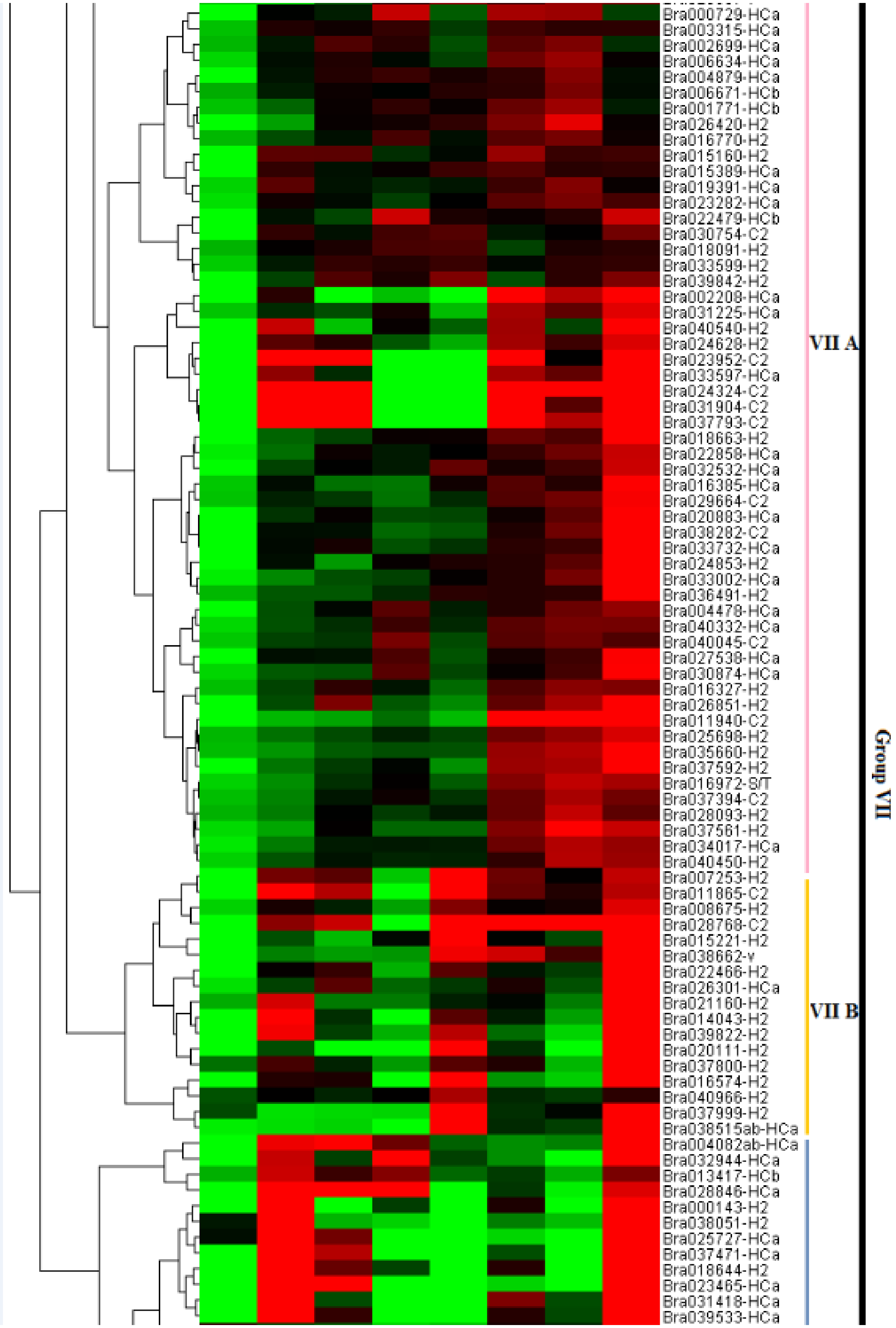
Bra000729-HCa
Bra003315-HCa
Bra002699-HCa
Bra006634-HCa
Bra004879-HCa
Bra006671-HCb
Bra001771-HCb
Bra026420-H2
Bra016770-H2
Bra015160-H2
Bra015389-HCa
Bra019391-HCa
Bra023282-HCa
Bra022479-HCb
Bra030754-C2
Bra018091-H2
Bra033599-H2
Bra039842-H2
Bra002208-HCa
Bra031225-HCa
Bra040540-H2
Bra024628-H2
Bra023952-C2
Bra033597-HCa
Bra024324-C2
Bra031904-C2
Bra037793-C2
Bra018663-H2
Bra022858-HCa
Bra032532-HCa
Bra016385-HCa
Bra029664-C2
Bra020883-HCa
Bra038282-C2
Bra033732-HCa
Bra024853-H2
Bra033002-HCa
Bra036491-H2
Bra004478-HCa
Bra040332-HCa
Bra040045-C2
Bra027538-HCa
Bra030874-HCa
Bra016327-H2
Bra026851-H2
Bra011940-C2
Bra025698-H2
Bra035660-H2
Bra037592-H2
Bra016972-S/T
Bra037394-C2
Bra028093-H2
Bra037561-H2
Bra034017-HCa
Bra040450-H2
Bra007253-H2
Bra011865-C2
Bra008675-H2
Bra028768-C2
Bra015221-H2
Bra038662-v
Bra022466-H2
Bra026301-HCa
Bra021160-H2
Bra014043-H2
Bra039822-H2
Bra020111-H2
Bra037800-H2
Bra016574-H2
Bra040966-H2
Bra037999-H2
Bra038515ab-HCa
Bra004082ab-HCa
Bra032944-HCa
Bra013417-HCb
Bra028846-HCa
Bra000143-H2
Bra038051-H2
Bra025727-HCa
Bra037471-HCa
Bra018644-H2
Bra023465-HCa
Bra031418-HCa
Bra039533-HCa
VII A
VII B
Group VII

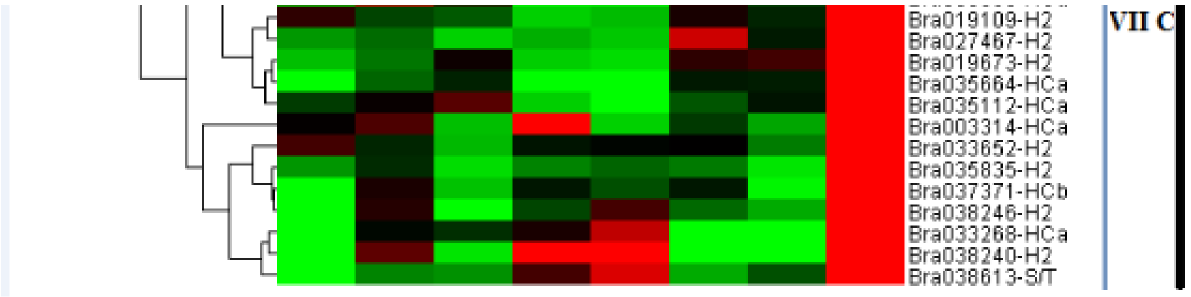

Bra019109-H2
Bra027467-H2
Bra019673-H2
Bra035664-HCa
Bra035112-HCa
Bra003314-HCa
Bra033652-H2
Bra035835-H2
Bra037371-HCb
Bra038246-H2
Bra033268-HCa
Bra038240-H2
Bra038613-S/T
VII C
